# Bicyclic anionic receptors for carboxylates in water†

**DOI:** 10.1039/d5sc04104j

**Published:** 2025-07-05

**Authors:** Xudong Ren, Anthony P. Davis

**Affiliations:** a University of Bristol, School of Chemistry Cantock's Close Bristol BS8 1TS UK anthony.davis@bristol.ac.uk

## Abstract

The selective recognition of carboxylates in water, the biological solvent, could have various applications in biology and medicine. Of particular interest is the design of antibiotics which mimic the glycopeptides such as vancomycin through binding C-terminal peptide units involved in bacterial cell wall synthesis. Here we report a general approach to carboxylate receptors with structures capable of encapsulating and interacting with all parts of their substrates. The synthesis involves elaboration of a diamino bridge unit into a bicyclic system incorporating a tetralactam anion binding site. Water-solubility can be achieved in a final step which introduces two dendrimeric nonacarboxylate units *via* Cu(i)-catalysed azide–alkyne cycloaddition. Three examples have been prepared and found to bind simple carboxylates and polar inorganic anions with *K*_a_ up to ∼400 M^−1^ in water at near-neutral pH, despite the presence of polycarboxyl solubilising groups. Selectivities are modest, probably because of the flexible bridge units employed, but the versatile synthesis should allow access to a wide range of variants including some with potential for medical applications.

## Abstract 2

A generalisable reaction sequence converts diamines into carboxylate receptors which can enclose their substrates and operate effectively in water.

## Graphical abstract

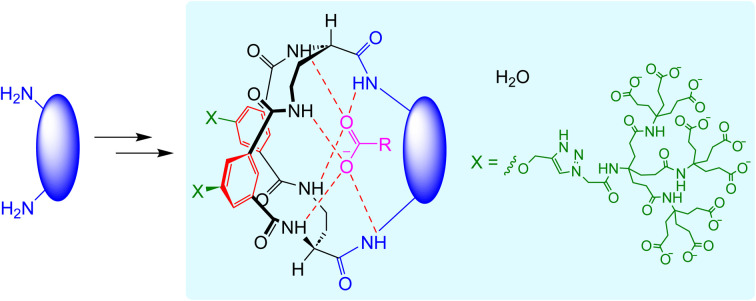

## Introduction

**Fig. 1:**
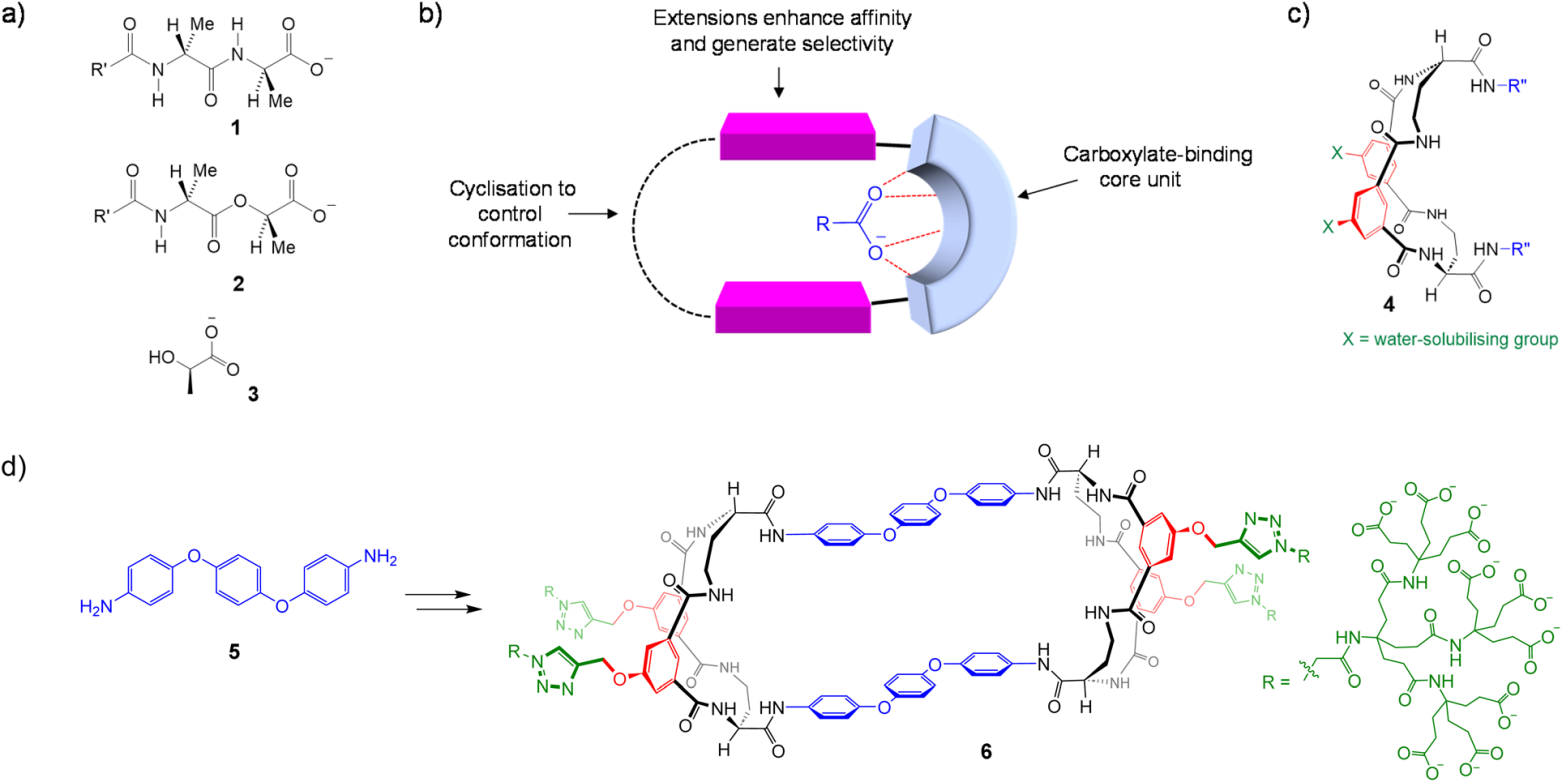
(a) Potential targets for carboxylate receptors. (b) Generalised design concept for selective carboxylate receptors. (c) The disubstituted tetralactam core unit employed in this work. (d) Diamine 5 and the derived divalent receptor 6, as previously reported. The solubilising groups are shown as polycarboxylates, but are not thought to be fully ionised at neutral pH.

The design of receptors for anions in water is a challenging task, especially if one wishes to avoid using Lewis acids or positive charges.^1–4^ Good solutions are available for the more hydrophobic anions,^5–8^ but charge-neutral receptors which target hydrophilic oxoanions are still very rare.^9,10^ Among anionic targets, the carboxylate group is especially prevalent in biology, appearing in proteins and peptides, oligosaccharides and many other biochemicals. Selective carboxylate receptors could therefore produce a variety of useful effects. Substrates of interest include the d-Ala-d-Ala C-terminal peptide 1, and the related d-Ala-d-Lac unit 2 (see Fig. 1a). Dipeptide 1 plays a central role in bacterial cell wall synthesis and is targeted by glycopeptide antibiotics such as vancomycin, while ester 2 is employed by bacteria which are resistant to the glycopeptides.^11,12^ Receptors for either (or ideally both) would have potential as novel antibiotics.^13^ Alternatively, receptors for smaller targets such as lactate 3 might find use in sensors.^14^ Despite these motivations, carboxylate recognition in water has received limited study.^3,15,16^ In particular, there has been very little work on carboxylate receptors with electroneutral binding sites, which may be less susceptible to high ionic strength and non-selective binding.^10^ Researchers have probably been discouraged by the high hydration energy of the RCO_2_^−^ unit,^17^ expected to curtail affinities, as well as practical issues such as receptor solubilisation.^1^

A selective receptor for carboxylate RCO_2_^−^ will presumably need a binding site for the anionic group with extensions that can interact with group R, increasing affinity and conferring selectivity (see Fig. 1b). As indicated, cyclisation to link the extensions is likely to reduce flexibility and help preorganisation. We considered that core binding sites with well-defined 3D architectures would be useful to preorganise the extensions for binding to R, and the disubstituted tetralactam unit 4 ^18^ (Fig. 1c) emerged as a favoured candidate. As described previously,^19^ versions of 4 with R′′ = aromatic groups did not perform well as receptors for simple carboxylates in water. We therefore decided to link the two R′′ groups to generate bicyclic structures, beginning with a bis(phenoxy)phenyl bridge derived from diamine 5 (Fig. 1d). Unexpectedly, the synthesis led to the tricyclic, dimeric structure 6 with two equivalent binding sites. Divalent receptor 6 was found to perform well as a receptor for carboxylates in water – the first example of a synthetic charge-neutral binding site with this capability.^19^ Notably, this was achieved despite the presence of multiple solubilising carboxylate groups.

**Fig. 2:**
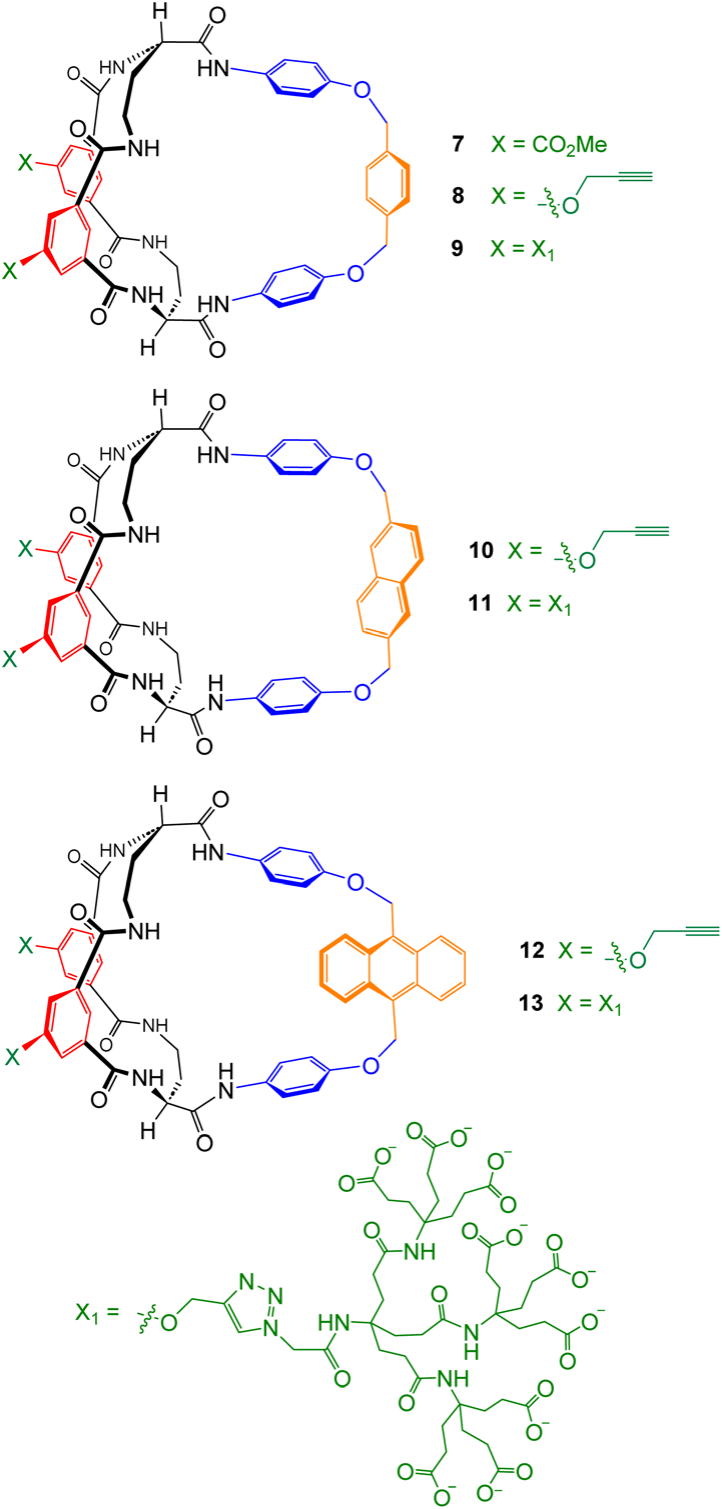
Carboxylate receptors prepared and studied in this work.

Although 6 was remarkably effective, the dimeric structure is too large and complex to serve as a starting point for receptor development. Moreover, calculations suggested that the structure would adopt a folded conformation in water, lacking an open hydrophobic cavity. We therefore wanted to show we could access structures which align with our original concept, with just one tetralactam binding site and cavities capable of enclosing carboxylate substrates. Here we describe the synthesis of a series of bicyclic receptors 7–13 (Fig. 2), which conform to Fig. 1b. We show that they are effective in both organic media and water, and provide evidence for the expected binding mode. We also show that the design can be adapted to provide fluorescence sensing of anions in water. The work serves as an important step towards selective carboxylate recognition under biological conditions, including mimicry of the glycopeptide antibiotics.

## Results and discussion

### Design and synthesis

**Scheme 1:**
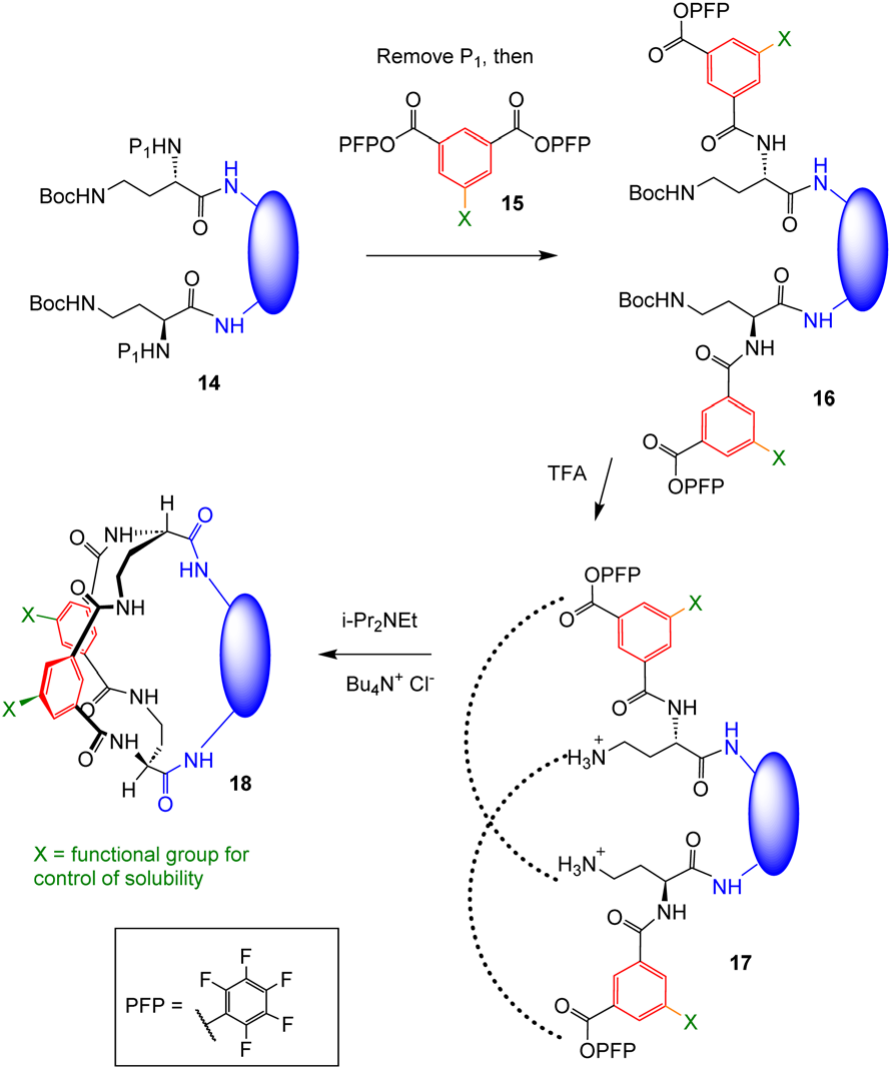
General synthetic scheme for bicyclic carboxylate receptors based on tetralactam core 4. P_1_ = Fmoc for 7–11 (remove with NaOH/MeOH) and Teoc for 12–13 (remove with TBAF).

Our synthetic approach to receptors such as 7–13 is shown in Scheme 1. An intermediate 14 is generated, formally derived from a diamine (blue) and differentially protected l-diaminobutyric acid (Dab). Protecting groups P_1_ are removed and the resulting diamine treated with an excess of an activated isophthalate ester 15 to give intermediate 16. Group X is included in the isophthalate to allow control of solubility. In the present work, X = CO_2_Me for 7, and O–CH_2_–C≡CH for 8, 10 and 12. The O–CH_2_–C≡CH groups can be converted into various solubilising groups at the end of the synthesis, in this case the dendrimeric nonacarboxylate X_1_ (see Fig. 2). Intermediate 16 is treated with acid to remove the Boc protection leading to diammonium diester 17. Finally addition of 17 to a tertiary amine at high dilution deprotonates the ammoniums and leads to bicyclic tetralactams 18. The final cyclisation is accelerated by the addition of chloride ions, presumed to act as templates. In the present work, it proceeded in up to 65% yield.

In principle the scheme is highly versatile. The only limitation on the blue diamine component is that the two amino nitrogens must be close enough together for the final cyclisation, although it is also desirable that the amide groups in target bicycle 18 should all be available for H-bond donation to a carboxylate. In particular, the diamine need not be symmetrical; the C2 symmetry of the core tetralactam unit ensures that only one product is possible. In the present work, we chose diamino bridges that might assist binding and selectivity through hydrophobic and/or steric interactions with the carboxylate R-group, as well as generating a fluorescence response for use in sensing.

**Scheme 2:**
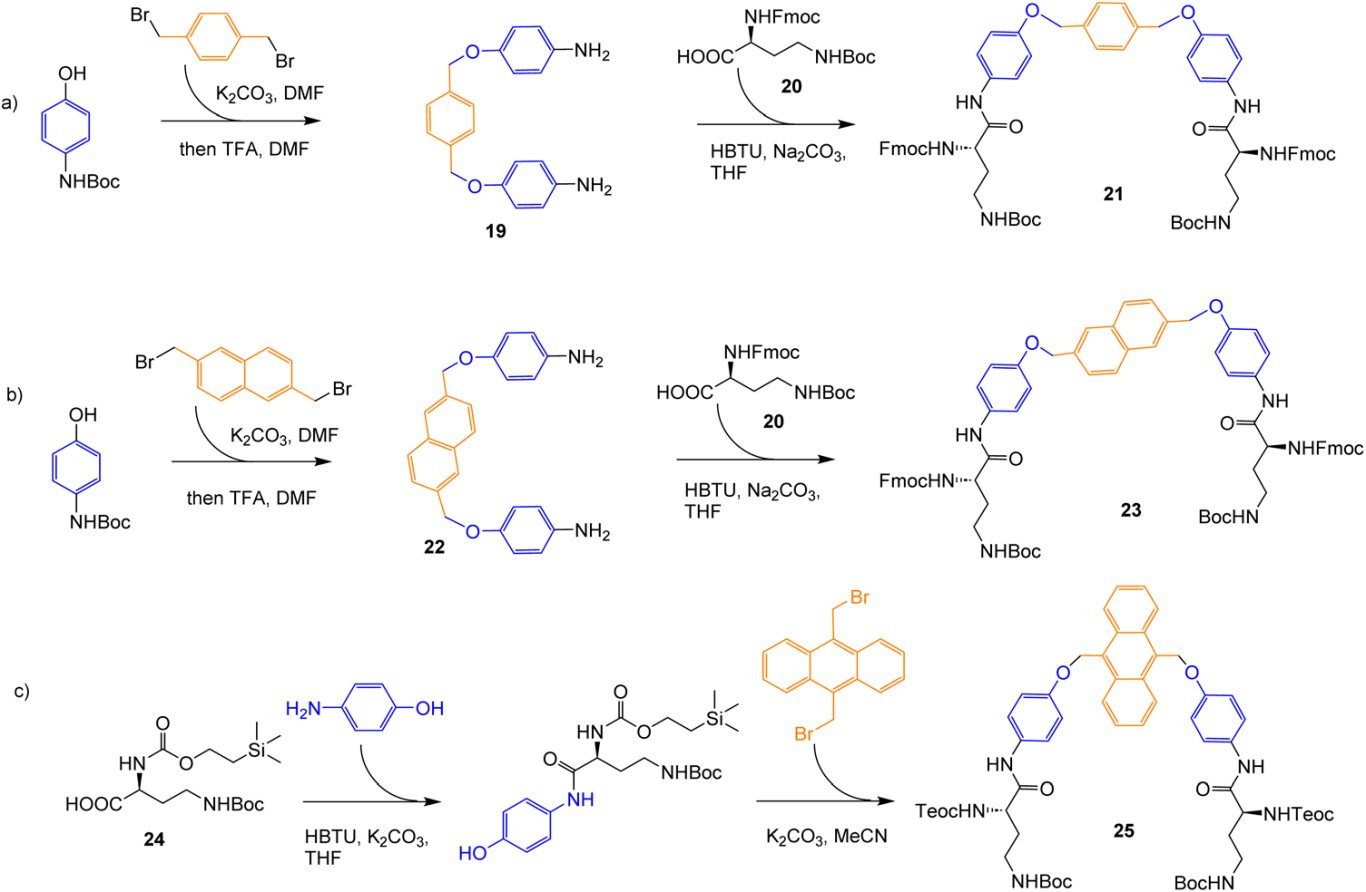
Preparation of acyclic intermediates required for the syntheses of 7–13.

For initial trials, we chose the phenyloxy-*p*-xylyl-oxyphenyl bridge in 7–9, derived from diamine 19. Monte Carlo molecular mechanics searches suggested that, unlike 6, these structures should possess persistent amphiphilic cavities in water, defined by the tetralactam ring and the bridge aromatic surfaces (Fig. S104†). The apolar cleft formed by the bridge could accommodate straight-chain carboxylates up to butanoate (see Fig. S105–S108†). Diamine 19 was prepared and converted into intermediate 21*via* reaction with commercially available 20, as shown in Scheme 2a. Intermediate 21 was then carried forward to bicycles 7 and 8, as indicated in Scheme 1. For studies in water, the dendrimeric solubilising group X_1_ (Fig. 2) was added to 8*via* Cu-catalysed azide alkyne cycloaddition, as described previously,^19^ to give 9.

To generate a second example, the benzene unit in the bridge was mutated into a naphthalene, as in 10 and 11. This enlarged the cavity slightly, adding apolar surface, and introduced fluorescence in the region 300–480 nm. The intermediate 23 required to prepare 10 and 11 was synthesised *via* diamine 22, as shown in Scheme 2b. Intermediate 23 was converted into organic-soluble receptor 10 as indicated in Scheme 1, and thence into water-soluble 11.

Finally, apolar surface and fluorescence emission were further increased by introducing an anthracene unit, as in 12 and 13. The synthesis of 12/13 was complicated by the sensitivity of the anthracenyl CH_2_–O unit to the acid conditions needed to remove *N*-Boc groups, as discussed below, as well as solubility issues. Intermediate 25 was therefore prepared *via* a route which did not involve strong acid, and employed the Teoc-protected starting material 24 to increase solubility (Scheme 2c).

**Scheme 3:**
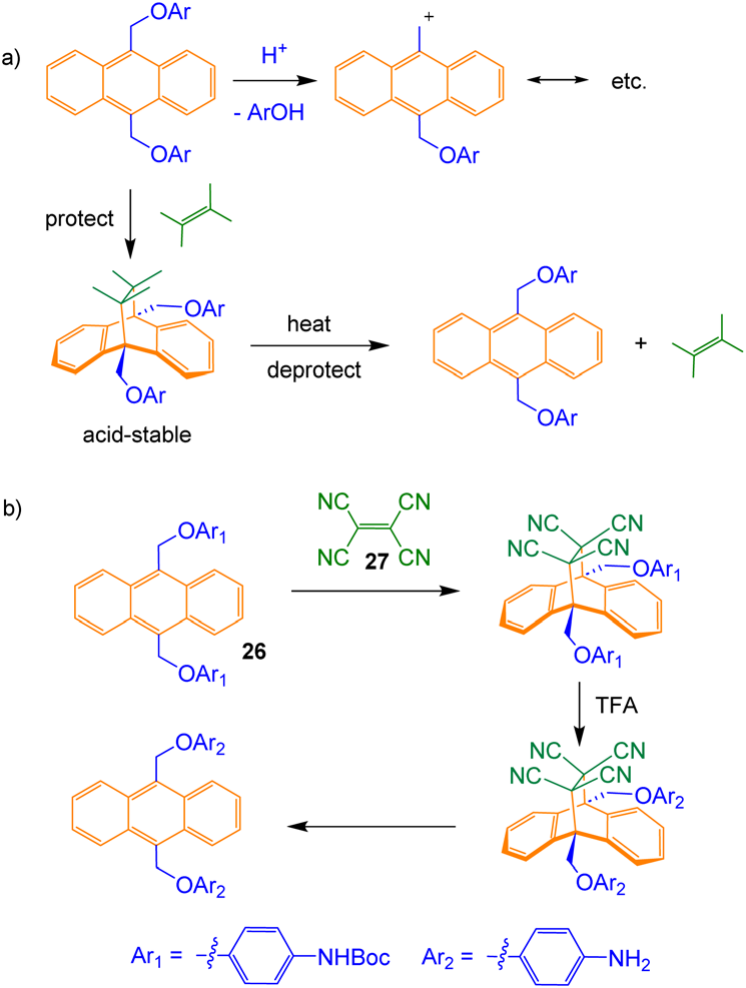
(a) Acid-catalysed decomposition of anthracenyl intermediates such as 25, and the application of a reversible Diels–Alder reaction as a protection strategy. (b) Trial reactions starting with 26 establish the practicality of the approach.

The acid-sensitivity of the anthryl CH_2_–O bond also raised issues later in the sequence, and these were resolved by an unusual stratagem. The source of the problem is the nature of the carbocation derived from CH_2_–O cleavage. As shown in Scheme 3a, acid-catalysed loss of ArOH from an intermediate such as 25 generates a cation stabilised by extended conjugation. Anthracenes are known to undergo Diels–Alder reactions across positions 9 and 10, and these can often be reversed by heating.^20,21^ The Diels–Alder adduct should not be acid-sensitive, and this could provide a straightforward way to protect the system from damage.

**Scheme 4:**
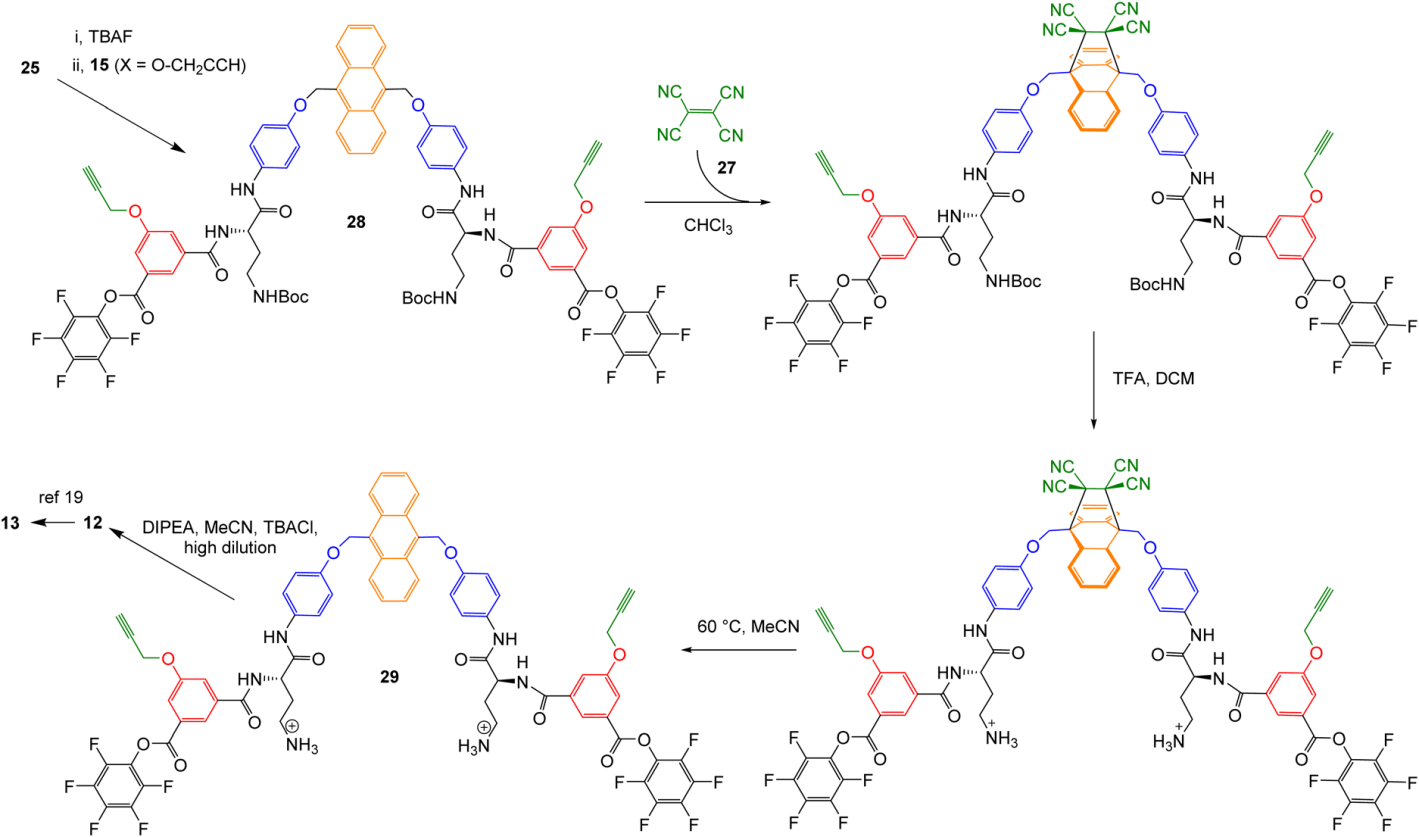
Conversion of intermediate 25 into receptors 12 and 13, employing TCNE 27 for anthracene protection.

The scheme proved quite easy to implement. Tetracyanoethene (TCNE, 27)^22^ and close relatives^23,24^ were known to react efficiently and reversibly with 9,10-disubstituted anthracenes. Trial experiments on model compound 26 showed that the Diels–Alder, acid-catalysed Boc deprotection and reverse Diels–Alder all worked well (Scheme 3b). Accordingly, intermediate 25 was converted to bis-PFP ester 28 and carried through to diprotonated diamine 29 as shown in Scheme 4. 29 was then cyclised to 12 through addition to a tertiary amine, following the pattern in Scheme 1. For studies in water, the dendrimeric solubilising group X_1_ (Fig. 2) was installed *via* Cu-catalysed azide alkyne cycloaddition to give 13.

Schemes showing each sequence in full are available in the ESI.†

### Binding and structural studies – organic media

**Fig. 3:**
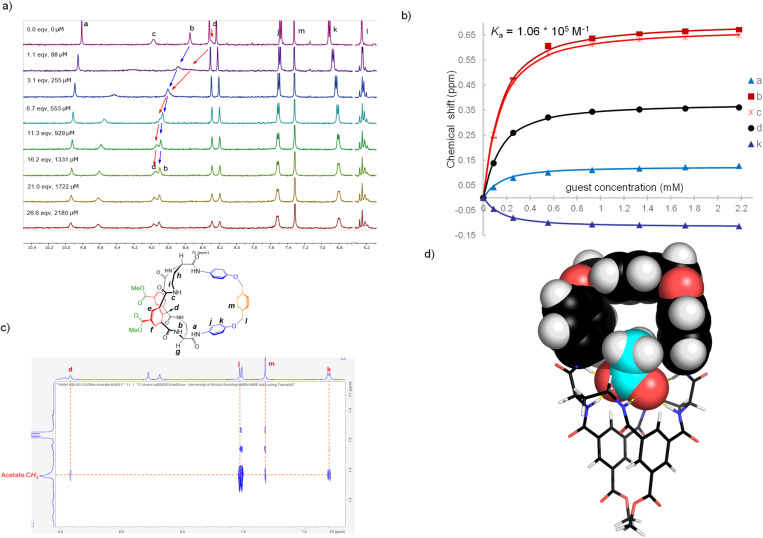
(a) ^1^H NMR spectra (500 MHz) from a titration of receptor 7 with TBA acetate in DMSO-d^6^. (b) Chemical shift measurements from the spectra in (a) fitted to a 1 : 1 binding model in Bindfit. (c) Portion of the 2D NOESY spectrum of receptor 7 (1.5 mM) + TBA acetate (5 mM) in DMSO-d^6^. (d) Energy-minimised structure of receptor 7 binding acetate anion *via* six NH⋯O^−^ hydrogen bonds in the expected binding geometry. Details of the calculations are given in the ESI.†

Bicycles 7, 8, 10 and 12 were suitable for study in organic media. Previous work^19^ had shown that the tetralactam core was capable of binding carboxylates with affinities in the range 10^3^–10^4^ M^−1^ in DMSO-d^6^, and this solvent was again used to allow comparisons. ^1^H NMR titrations were performed with tetrabutylammonium (TBA) salts of acetate, propionate, butyrate and benzoate as titrants. Substantial signal movements were observed in all cases, consistent with binding with fast exchange on the ^1^H NMR timescale. Spectra from the titration of 7 with TBA acetate are shown in Fig. 3a. The lactam NH protons b and c move downfield by >0.6 ppm, consistent with H-bond donation to the acetate substrate. The bridge NHa also moves downfield, but only by ∼0.1 ppm suggesting that its role in binding is less important. Inward-directed aromatic CHd moves downfield by ∼0.35 ppm while bridge CHk, which is relatively remote from the tetralactam binding site, moves upfield significantly. These movements were analysed using the programme Bindfit,^25^ and fit well to a 1 : 1 binding model with *K*_a_ = 10 600 M^−1^ (Fig. 3b). In addition, protons j move slightly downfield and protons l separate to form an AB quartet.

The movements of protons j and k seemed to suggest a direct interaction between the host bridge and the guest, as expected if the acetate is positioned in the cavity. The separation of protons l suggests a loss of conformational freedom in the bridge, also consistent with intracavity binding. To provide further evidence of this binding geometry, a NOESY spectrum was acquired for a 1 : 3 mixture of 7 and TBA acetate. As shown in Fig. 3c, a strong cross-peak was recorded between acetate CH_3_ and receptor proton j. The complex was modelled using molecular mechanics, confirming that these protons should be in close proximity (Fig. 3d and see also S106†). The results thus suggest that the substrate does indeed enter the cavity where, in principle, it can be affected by the choice of bridge.

The binding studies with TBA propionate and butyrate as substrates produced fairly similar results, the main difference being that the bridge NHa moved very little. The affinities are shown in Table 1, and were very similar to that for acetate. Again, NOESY spectra provided evidence that the anions entered the cavity of 7. For both anions, connections were revealed between α-CH_2_ and receptor protons j, and between the terminal CH_3_ and receptor protons m (Fig. S101 and S102†). For benzoate, protons b–d moved downfield, and k upfield, as for the other anions. However, in this case NHa moved upfield by ∼0.3 ppm, and bridge CHj also moved upfield significantly. The measured affinity was somewhat lower, at 3400 M^−1^. These differences are again consistent with the proposed mode of binding. Modelling suggests that benzoate is too large to enter the cavity but will clash with the bridge (Fig. S109†). The structure of the complex should therefore differ somewhat from those of the others. The lower binding constant could also be due to these steric interactions.

**Table 1:** ^1^H NMR titrations of organic-soluble receptors in DMSO-d^6^

Substrate^a^	Binding constants *K*_a_^b^ (M^−1^)			
	7	8	10	12
Acetate	10 600 (±2.6%)	11 500 (±3.4%)	10 400 (±4.0%)	10 800 (±2.1%)
Propionate	10 900 (±5.3%)	10 100 (±1.9%)		
*n*-Butyrate	10 100 (±4.4%)	9100 (±1.9%)		
Benzoate	3200 (±0.9%)	3400 (±1.3%)	3000 (±2.9%)	2400 (±2.6%)

a As tetrabutylammonium salts.

b Analysed using Bindfit.^25^

Receptor 8 was also studied with all four anions, giving very similar results to 7. Receptors 10 and 12 were tested with just acetate and benzoate, showing similar affinities to 7 and 8. The full set of data is listed in Table 1.

### Binding and structural studies – aqueous media

Bicycles 9, 11 and 13 were designed to operate in aqueous media at near-neutral pH. Samples for study were prepared by suspending the receptors in water, adding NaOH to adjust the pH to ∼7.4, then freeze-drying the resulting clear solutions. Redissolving in H_2_O or D_2_O gave solutions for characterisation and binding experiments. Given that the receptors contained carboxylate solubilising groups as well as a carboxylate binding site, it was necessary to check for self-association. ^1^H NMR dilution studies showed small signal movements consistent with intermolecular carboxylate–tetralactam interactions, but implied that 9, 11 and 13 should be monomeric below 90, 75 and 56 μM respectively. DOSY spectra on 9 supported this interpretation, yielding an estimated diameter of ∼2 nm, consistent with the monomeric structure (Fig. S24 and S25†). UV-visible and fluorescence spectroscopic dilution experiments on 11 and 13 showed linear concentration dependences up to 10 μM. Another consideration was intramolecular association between the binding sites and side-chain carboxylates. In principle this can be probed through NMR spectra at differing pH. The side-chain carboxylates are not expected to be fully ionised at neutral pH,^26^ so it is likely that binding site occupancy will increase with pH. The phenomenon had been observed with dimeric receptor 6, where increasing the pH from 7.7 to 9 resulted in significant ^1^H NMR signal movements. Similar experiments were performed here for 9, 11 and 13, with similar results. The data were consistent with intramolecular binding, but also implied that empty binding sites were available at near-neutral pH (otherwise little change would be expected). Fluorescence pH titrations on 11 and 13 also produced changes, but these were more difficult to interpret. The experiments confirmed the need to study binding at a constant, controlled pH.

**Fig. 4:**
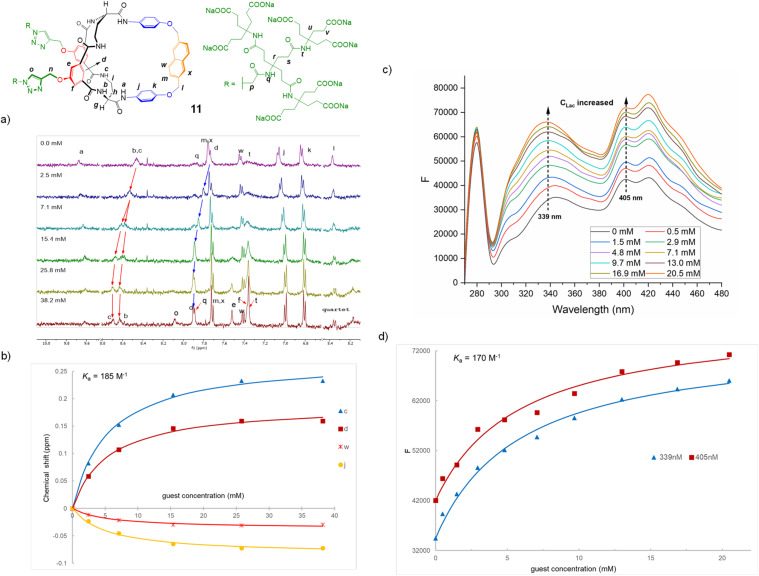
(a) ^1^H NMR spectra (500 MHz) from a titration of receptor 11 (16 μM) with sodium l-lactate in 1 : 9 D_2_O/H_2_O at constant near-neutral pH. (b) Chemical shift measurements from the spectra in (a) fitted to a 1 : 1 binding model in Bindfit. (c) Fluorescence spectra from a titration of receptor 11 (2 μM) titrated with sodium l-lactate in H_2_O at constant near-neutral pH. Excitation wavelength = 255 nm, slit width for both excitation and emission = 4 nm. (d) Emission intensities at two wavelengths from the spectra in (c) fitted to a 1 : 1 binding model in Bindfit.

^1^H NMR binding studies were performed in H_2_O/D_2_O 9 : 1 at near neutral pH (7.30–7.65), with a range of anionic substrates (see Fig. 4a and Table 2). The guest solutions were carefully adjusted to match the pH of the host. The host concentration was always below the threshold for aggregation, as determined by the dilution studies (see above). Signal movements were observed, consistent with 1 : 1 binding which is fast on the ^1^H NMR chemical shift timescale. Fig. 4a shows an example involving receptor 11 titrated against l-lactate. Protons b, c and d, expected to interact directly with the carboxylate, were observed to move downfield for all receptor–substrate combinations, although the movements for 13 were smaller than for the other two receptors. Proton a generally moved slightly upfield, as did protons j and, to a lesser extent, k. Protons l again changed from a singlet to an AB quartet suggesting a loss of conformational freedom in the complex. For each titration, several signals could be followed and were analysed using Bindfit^25^ based on the 1 : 1 binding model (see for example Fig. 4b).

**Table 2:** Binding constants (*K*_a_, M^−1^) to water-soluble receptors measured by titration methods in aqueous solution at near-neutral pH

Substrate	9		11			13	
	^1^H NMR^a^	ITC^b^	^1^H NMR^a^	Fluorescence^a^	ITC^b^	^1^H NMR^a^	Fluorescence^a^
Formate	70 (±4.5%)		93 (±2.9%)				
Acetate	79 (±2.5%)	33	123 (±3.6%)	102 (±3.0%)	54	154 (±4.5%)	
Propionate	100 (±1.7%)		154 (±6.6%)	144 (±4.6%)		182 (±4.1%)	
*n*-Butyrate	126 (±3.0%)	93	154 (±5.3%)		69	148 (±4.6%)	
Iso-butyrate	134 (±3.6%)		198 (±5.4%)			182 (±2.7%)	
Pivalate	146 (±3.1%)		174 (±6.4%)				
Benzoate	135 (±3.5%)		110 (±8.0%)			176 (±3.3%)	
l-Lactate	135 (±4.6%)		185 (±5.4%)	170 (±7.8%)		161 (±4.7%)	143 (±5.6%)
d-Lactate	140 (±3.9%)		191 (±8.0%)			181 (±3.3%)	
Chloride	96 (±1.7%)	54	192 (±4.8%)	207 (±10.3%)	77	239 (±7.8%)	
Bromide	137 (±3.0%)		203 (±5.4%)				
Iodide	112 (±3.1%)		215 (±4.6%)			205 (±4.9%)	
Sulphate	232 (±2.5%)					406 (±7.0%)	
Nitrate	100 (±1.6%)						

a Analysed using Bindfit.^25^

b Analysed using an Excel spreadsheet developed in-house, see ESI.

The resulting *K*_a_ values are listed in Table 2. The affinities are generally consistent with those measured for dimeric receptor 6, considering that the latter benefits from a statistical contribution due to the two identical binding sites.^27^ As in the case of 6, selectivities between different carboxylates are modest. This suggests that binding is dominated by polar interactions, while hydrophobic and steric interactions with the bridge play a relatively minor role. Inorganic anions are also bound, especially the well-hydrated sulphate, again suggesting that polar interactions predominate. Differences between the receptors are also quite small, but seem to be significant in some cases. In particular, 13 is generally slightly stronger than 9, both for carboxylates and inorganic anions.

As discussed earlier, the introduction of fluorescence signalling was a key objective of this work. The naphthalene unit in 11 generated useful levels of emission between 300 and 500 nm if excited between 250 and 300 nm. Excitation at 250 nm was preferred for binding studies as this minimised the Raman emission of the solvent. Addition of anions caused a roughly two-fold increase in emission intensity, as exemplified for 11 + l-lactate in Fig. 4c. The changes were again consistent with 1 : 1 binding, and analysis with Bindfit gave closely similar affinities to those measured by NMR (*e.g.*Fig. 4d). The anthracene unit in 13 generated peak emission at longer wavelengths than 11. However addition of l-lactate induced just a ∼1.3-fold increase in fluorescence. This could be analysed to obtain an affinity, but the low sensitivity discouraged further investigations. As further confirmation of binding, we also performed ITC measurements on 9 and 11 with acetate, isobutyrate and chloride. The results are listed in Table 2. Sensitivity limitations necessitated host concentrations above the aggregation threshold, so it is unsurprising that the values are slightly lower than those obtained by the other methods.

Finally, we sought direct evidence that the substrates enter the cavities of these water-soluble macrocycles, as already shown for 7 + acetate, propionate and butyrate in DMSO. NOESY spectroscopy was complicated by the low binding constants and the intense signals from the solubilising groups, especially as these tended to overlap with substrate protons of interest. However a NOESY spectrum of 9 + propionate showed cross-peaks which, despite overlaps, could be assigned with reasonable confidence to connections between propionate α-CH_2_ and receptor protons d and j, as expected for enclosed substrate. Further details are given in the ESI (Fig. S103).†

## Conclusions

The most important conclusion derived from this work is that carboxylate receptors capable of encapsulating their substrates can be prepared *via* a sequence of reactions which occur in reasonable yields and, importantly, would seem to be quite general. There should be a very wide range of diamines to which the method could be applied, generating a variety of potential binding sites. Versatile solubilisation is built into the methodology – the alkynyl groups introduced with reagent 15 can be variously modified in a straightforward final step.

A second conclusion is that the tetralactam binding site 4, when constrained by a polycyclic architecture, is confirmed to be effective at binding carboxylates and other hydrophilic anions in water even in the presence of 18 solubilising carboxyl groups. The fluorescence results for 11 and 13 are especially helpful in this respect, providing complementary data that was not available for 6.

Thirdly, NOESY data support the proposed binding mode for carboxylates, in which the substrate enters the cavity as indicated in Fig. 1b. The shifts in bridge proton signals during ^1^H NMR titrations and the emission changes during fluorescence titrations are also supportive of intracavity binding. It might seem surprising that the bridge NHa NMR signals do not move consistently downfield on binding, but both DMSO and water are good H-bond acceptors which must be displaced by the carboxylates. Depending on the strength of the H-bonds formed on binding, substantial downfield movements are not necessarily expected. Similar behaviour was observed for the corresponding protons in 6.^19^

Fourthly, simple linearly connected bridges as in 7–13 are relatively ineffective in controlling selectivity, even in water where hydrophobic interactions can contribute to binding. These tris-aromatic straps are quite flexible and it seems that they cannot provide effective, well-defined hydrophobic pockets. However, given the versatility of the synthesis it should be possible to generate analogues with more structured cavities, capable of both polar and hydrophobic interactions. Improved discrimination should thus be possible, perhaps leading to selectivity for C-terminal peptides and ultimately to mimics of the glycopeptide antibiotics.

## Experimental

For detailed experimental procedures see the ESI.†

## Author contributions

All experimental work including synthesis, spectroscopic studies and data analysis was performed by XR, who was also responsible for the specific designs. The project was conceived and supervised by APD. XR and APD wrote the paper.

## Conflicts of interest

There are no conflicts to declare.

## Supplementary Material

SC-OLF-D5SC04104J-s001

## Data Availability

The data that support the findings of this study are available in the ESI.†

## References

[cit1] Langton M. J., Serpell C. J., Beer P. D. (2016). Anion Recognition in Water: Recent Advances from a Supramolecular and Macromolecular Perspective. Angew. Chem., Int. Ed..

[cit2] Kubik S. (2017). Anion Recognition in Aqueous Media by Cyclopeptides and Other Synthetic Receptors. Acc. Chem. Res..

[cit3] Kubik S. (2010). Anion recognition in water. Chem. Soc. Rev..

[cit4] Li J. H., Catal O., Marques I., McNaughton D. A., Maklad R. M., Ryder W. G., Hill M. J. S., Seddon A., Lewis W., Adams D. J., Félix V., Wu X., Gale P. A. (2025). Trapping Anions within Stacks of Tetra-Urea Macrocycles. J. Am. Chem. Soc..

[cit5] Borissov A., Marques I., Lim J. Y. C., Felix V., Smith M. D., Beer P. D. (2019). Anion Recognition in Water by Charge-Neutral Halogen and Chalcogen Bonding Foldamer Receptors. J. Am. Chem. Soc..

[cit6] Yawer M. A., Havel V., Sindelar V. (2015). A Bambusuril Macrocycle that Binds Anions in Water with High Affinity and Selectivity. Angew. Chem., Int. Ed..

[cit7] Lizal T., Sindelar V. (2018). Bambusuril Anion Receptors. Isr. J. Chem..

[cit8] Lisbjerg M., Nielsen B. E., Milhoj B. O., Sauer S. P. A., Pittelkow M. (2014). Anion binding by biotin 6 uril in water. Org. Biomol. Chem..

[cit9] He M., Yao Y., Yang Z., Li B., Wang J., Wang Y., Kong Y., Zhou Z., Zhao W., Yang X.-J., Tang J., Wu B. (2024). Biomimetic Charge-Neutral Anion Receptors for Reversible Binding and Release of Highly Hydrated Phosphate in Water. Angew. Chem., Int. Ed..

[cit10] Jing L., Deplazes E., Clegg J. K., Wu X. (2024). A charge-neutral organic cage selectively binds strongly hydrated sulfate anions in water. Nat. Chem..

[cit11] Williams D. H., Bardsley B. (1999). The vancomycin group of antibiotics and the fight against resistant bacteria. Angew. Chem., Int. Ed..

[cit12] Wu Z.-C., Boger D. L. (2020). Maxamycins: Durable Antibiotics Derived by Rational Redesign of Vancomycin. Acc. Chem. Res..

[cit13] Flint A. J., Davis A. P. (2022). Vancomycin mimicry: towards new supramolecular antibiotics. Org. Biomol. Chem..

[cit14] Didier P., Minami T. (2020). Non-enzymatic lactate detection by an extended-gate type organic field effect transistor. Semicond. Sci. Technol..

[cit15] Hatai J., Schmuck C. (2019). Diverse Properties of Guanidiniocarbonyl Pyrrole-Based Molecules: Artificial Analogues of Arginine. Acc. Chem. Res..

[cit16] Giese M., Niemeyer J., Voskuhl J. (2020). Guanidiniocarbonyl-Pyrroles (GCP)-20 Years of the Schmuck Binding Motif. ChemPlusChem.

[cit17] Marcus Y. , in Ions in Solution and Their Solvation, John Wiley & Sons, 2015, pp. 107–155

[cit18] Chmielewski M. J., Zielinski T., Jurczak J. (2007). Synthesis, structure, and complexing properties of macrocyclic receptors for anions. Pure Appl. Chem..

[cit19] Ren X. D., Flint A. J., Austin D., Davis A. P. (2024). Polyanionic Receptors for Carboxylates in Water. Angew. Chem., Int. Ed..

[cit20] Chung Y. S., Duerr B. F., McKelvey T. A., Nanjappan P., Czarnik A. W. (1989). Structural effects controlling the rate of the retro-Diels-Alder reaction in anthracene cycloadducts. J. Org. Chem..

[cit21] Sanyal A., Yuan Q., Snyder J. K. (2005). A new, chiral aminoanthracene for the Diels-Alder/retro-Diels-Alder sequence in lactam and butenolide synthesis. Tetrahedron Lett..

[cit22] Masci B., Pasquale S., Thuéry P. (2008). Supramolecular Control of a Fast and Reversible Diels-Alder Reaction. Org. Lett..

[cit23] Reutenauer P., Boul P. J., Lehn J. M. (2009). Dynamic Diels-Alder Reactions of 9,10-Dimethylanthracene: Reversible Adduct Formation, Dynamic Exchange Processes and Thermal Fluorescence Modulation. Eur. J. Org Chem..

[cit24] Bruckner R., Huisgen R. (1994). Diels-Alder reactions with 2,2-bis(trifluoromethyl)ethylene-1,1-dicarbonitrile as dienophile. Tetrahedron Lett..

[cit25] http://supramolecular.org

[cit26] Destecroix H., Renney C. M., Mooibroek T. J., Carter T. S., Stewart P. F. N., Crump M. P., Davis A. P. (2015). Affinity enhancement by dendritic side chains in synthetic carbohydrate receptors. Angew. Chem., Int. Ed..

[cit27] Hibbert D. B., Thordarson P. (2016). The death of the Job plot, transparency, open science and online tools, uncertainty estimation methods and other developments in supramolecular chemistry data analysis. Chem. Commun..

