# Supplementary material for: Bicyclic anionic receptors for carboxylates in water
Source: Chem Sci. 2025 Jul 5;16(31):14314–22. doi: 10.1039/d5sc04104j (PMC12243157; doi:10.1039/d5sc04104j)
Supplement: SC-016-D5SC04104J-s001 [file SC-016-D5SC04104J-s001.pdf]

Supplementary Information (SI) for Chemical Science.
This journal is © The Royal Society of Chemistry 2025

Bicyclic anionic receptors for carboxylates in water

Xudong Ren and Anthony P. Davis[1]

[1]School of Chemistry, University of Bristol, Cantock's Close, Bristol, BS8 1TS, UK.

Table of Contents

1. Synthesis and Characterisation 4

1.1 General 4

1.2 Synthesis of receptor 7 5

1.3 Synthesis of receptors 8 and 9 10

1.4 Synthesis of receptors 10 and 11 14

1.5 Synthesis of receptors 12 and 13 19

Trial Experiments for Diels-Alder and retro Diels-Alder reactions 20

1.6 Deprotonation of carboxylic acids on receptors 29

1.7 Characterisation of receptors in their operating environments 30

Receptor **7** 30

Receptor **8** 33

Receptor **10** 35

Receptor **12** 37

Receptor **9** 39

Receptor **11** 41

Receptor **13** 43

1.8 Dilution studies 45

1.9.1 NMR dilution studies 45

1.9.2 UV-Vis and fluorescence dilution studies 49

1.9 pH titrations of receptors 53

1.10.1 NMR pH titrations 53

1.10.2 Fluorescence pH titrations 55

2. Binding studies 57

2.1 Nuclear Magnetic Resonance (NMR) titrations 57

2.1.1 Receptor **7** in DMSO-d^6 58

Acetate 58
Propionate 60
n-Butyrate 61
Benzoate 62
2.1.2 Receptor **8** in DMSO-*d*[6] 63
Acetate 63
Propionate 65
n-Butyrate 66
Benzoate 67
2.1.3 Receptor **10** in DMSO-*d*[6] 68
Acetate 68
Benzoate 70
2.1.4 Receptor **12** in DMSO-*d*[6] 71
Acetate 71
Benzoate 73
2.1.7 Receptor **9** in H_2O/D_2O 74
Formate 74
Acetate 76
Propionate 77
n-Butyrate 78
iso-Butyrate 79
Pivalate 80
Benzoate-d[5] 81
D-Lactate 82
L-Lactate 83
Chloride 84
Bromide 85
Iodide 86
Sulphate 87
Nitrate 88
2.1.8 Receptor **12** in H_2O/D_2O 89
Formate 89

Acetate ... 91
Propionate ... 92
n-Butyrate ... 93
iso-Butyrate ... 94
Pivalate ... 95
Benzoate-d^5 ... 96
D-Lactate ... 97
L-Lactate ... 98
Chloride ... 99
Bromide ... 100
Iodide ... 101
2.1.9 Receptor **13** in H_2O/D_2O ... 102
Acetate ... 102
Propionate ... 104
n-butyrate ... 105
iso-butyrate ... 106
L-Lactate ... 107
D-Lactate ... 108
Benzoate-d^5 ... 109
Chloride ... 110
Iodide ... 111
Sulphate ... 112
2.2 Fluorescence titrations ... 113
2.2.1 Photophysical Properties of receptors ... 114
Receptor **11** ... 114
Receptor **13** ... 116
2.2.2 Fluorescence titrations ... 117
Receptor **11** + Acetate ... 117
Receptor **11** + Propionate ... 118
Receptor **11** + L-Lactate ... 119
Receptor **11** + Chloride ... 120
Receptor **13** + L-Lactate ... 121

2.3 ITC titrations ... 122

Receptor **9** + Chloride ... 123

Receptor **9** + Acetate ... 124

Receptor **9** + n-Butyrate ... 125

Receptor **11** + Chloride ... 126

Receptor **11** + Acetate ... 127

Receptor **11** + n-Butyrate ... 128

2.4 NMR Structural Studies of Hosts and Complexes ... 129

3. Molecular Modelling ... 132

1. Synthesis and Characterisation

1.1 General

Commercial reagents were purchased from Sigma–Aldrich, Alfa Aesar or Fluorochem and were used without further purification unless otherwise specified. All air and moisture sensitive manipulations were carried out using standard vacuum line and Schlenk techniques. Solvents for air and moisture sensitive manipulations were obtained from an Anhydrous Engineering Solvent Purification System, distilled and dried over activated molecular sieves, or purchased from Sigma–Aldrich.

Flash column chromatography was performed on a Biotage® Selekt System using silica (Biotage® Sfär Silica D - 60 µm) or C18 (Biotage® Sfär C18 D - Duo 100 Å 30 µm) columns and a suitable eluent. TLC was performed using aluminium backed TLC plates (Merck-Keiselgel 60 F254) and visualised using UV fluorescence (254 or 365 nm) and/or developed using ninhydrin, potassium permanganate or bromocresol green. Prep-LCMS was performed on a system with the Waters 600 Controller, the Waters SQ Detector 2 and a XSelect CSH Prep C18 column

NMR spectra were recorded on Varian VNMR 400 MHz, Bruker 400 MHz, Varian VNMRS 500 MHz, Bruker Advance III HD Cryo 500 MHz, Bruker Neo Cryo 600 MHz and Bruker Cryo 700 MHz spectrometers. All spectra were obtained at 298 K. All ^{1}H and ^{13}C NMR chemical shifts are reported relative to the ^{1}H and ^{13}C chemical shifts of the solvent as standard. LRMS (low resolution mass spectrometry) was performed on a Waters 600 Controller with a Waters SQ Detector 2. HRMS (high resolution mass spectrometry) was performed on a Thermo Scientific Orbitrap Elite or a Waters Synapt G2S.

1.2 Synthesis of receptor 7

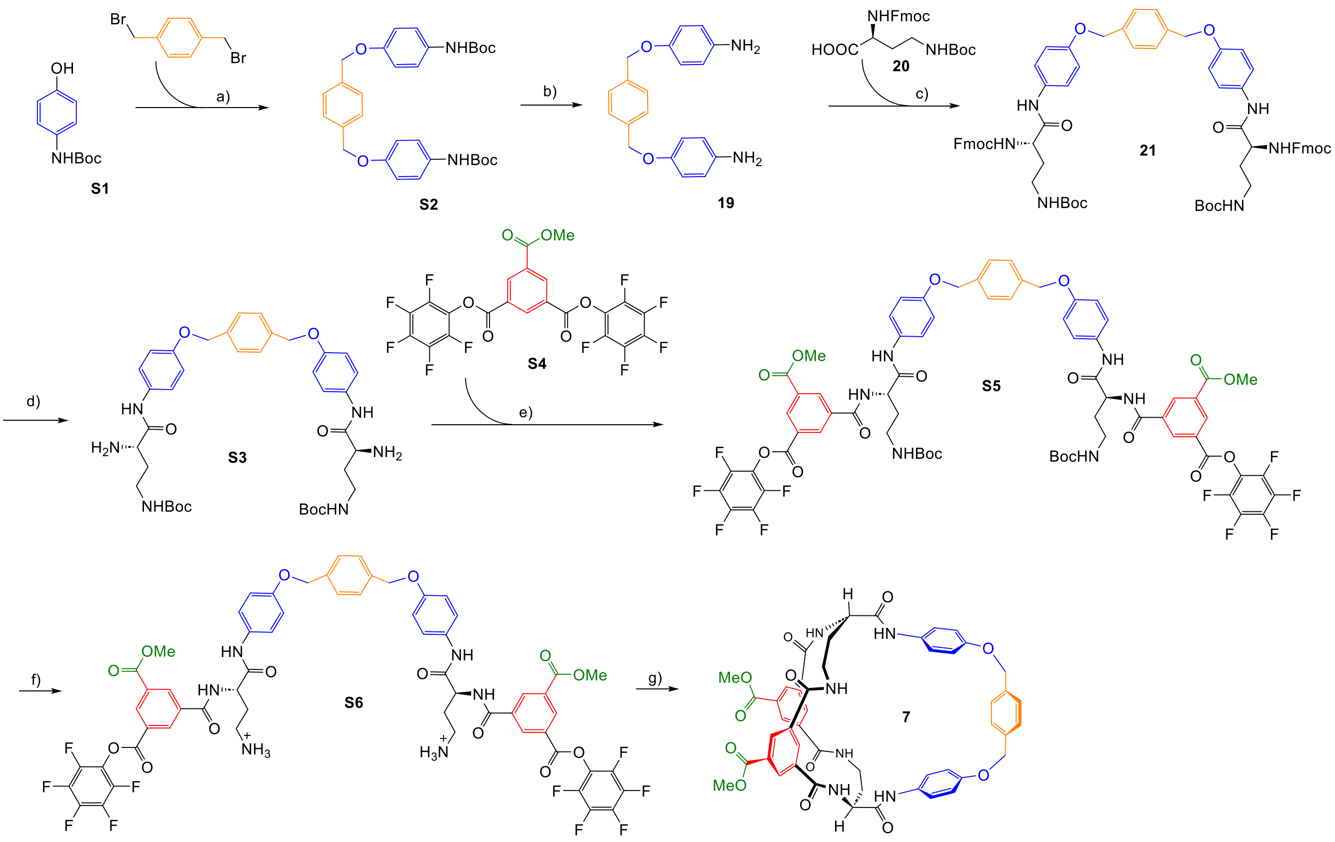

Scheme S1. a) K_2CO_3, DMF; b) TFA, DCM; c) HBTU, Na_2CO_3, THF; d) NaOH, MeOH; e) DIPEA, THF; f) TFA, DCM; g) DIPEA, TBACl, THF.

1-methyl 3,5-bis(perfluorophenyl) benzene-1,3,5-tricarboxylate S4

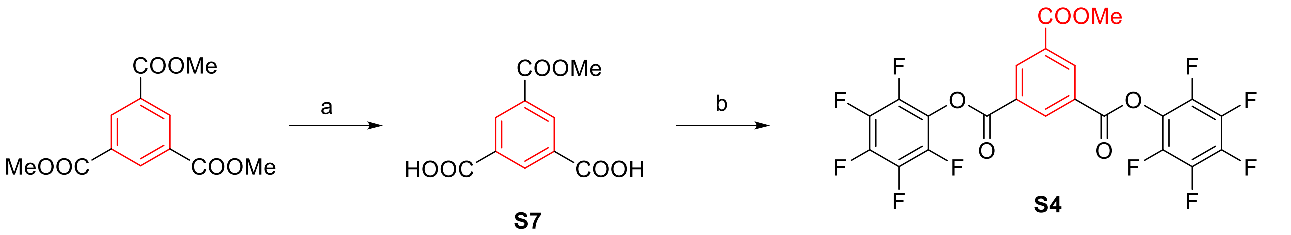

To a solution of trimethyl benzene-1,3,5-tricarboxylate (2.57 g, 10.2 mmol, 1.0 eqv) in MeOH (30 ml) was added NaOH (815 mg, 20.4 mmol, 2.0 eqv). The reaction mixture was stirred at room temperature for overnight before the mono-ester intermediate **S7** was precipitated by changing the pH to 1 using HCl aq. After filtration, the intermediate **S7** was obtained as white solid and was used in the next step without further purification. To a solution of **S7** in THF (50 ml) was added PFPOH (4.12 g, 22.4 mmol, 2.2 eqv) and DCC (5.25 g, 25.4 mmol, 2.5 eqv). The reaction mixture was stirred at room temperature for overnight before the solvent was removed *in vacuo*. The residue was dissolved in DCM (20 ml) and insoluble solid removed by filtration. After concentration *in vacuo*, the solid was recrystallized from hot hexane to give title compound **S4** (3.62 g, 6.5 mmol, 64 %) as a white solid.

^{1}**H NMR** (400 MHz, $CDCl_3$) δ 9.16 – 9.15 (m, 1H, C2H), 9.15 – 9.13 (m, 2H, C1H), 4.05 (s, 3H, C6H). ^{19}F (376 MHz, $CDCl_3$) δ -152.03 (d, *J* = 17.1 Hz, C3F), -156.47 (d, *J* = 21.8 Hz, C5F), -161.37 (dd, *J* = 21.7, 17.2 Hz, C4F). The data in accordance with the literature.[1]

4,4'-((1,4-phenylenebis(methylene))bis(oxy))dianiline 19

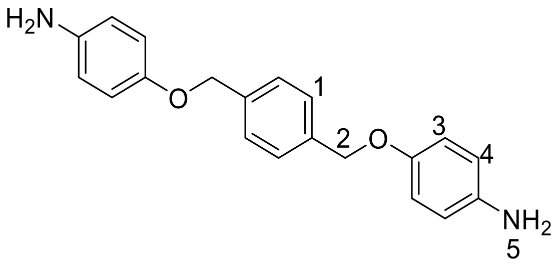

To a solution of 4-(Boc-amino)phenol **S1** (1.0 g, 4.78 mmol, 2.02 eqv) in DMF (10 ml) was added 1,4-bis(bromomethyl)benzene (625 mg, 2.37 mmol, 1.0 eqv) and K_2CO_3 (817 mg, 5.91 mmol. 2.5 eqv). The reaction mixture was stirred at 60 °C overnight. The reaction mixture was poured into water (50 ml) to give a precipitate, and a white solid was obtained after filtration. The solid was recrystallised from hot EtOAc to give the intermediate **S2** (751 mg, 1.44 mmol, 61 %) as a white solid. To a solution of intermediate **S2** (700 mg, 1.34 mmol) in dry DCM (6 ml) was added TFA (2 ml). The reaction was stirred at 0 °C overnight before volatiles were removed using a flow of N_2. The residue was dissolved in DCM (20 ml) and washed with *Sat.* $NaHCO_3$ aq solution (3 x 10 ml). The organic fraction was washed with brine (10 ml) and then dried over $MgSO_4$. The solvent was removed to give the title compound **19** (383 mg, 1.20 mmol, 89 %) as a colourless oil.

^{1}**H NMR** (400 MHz, DMSO-*d*$_6$) δ 7.39 (s, 4H, C1H), 6.75 – 6.65 (m, 4H, C3H), 6.53 – 6.45 (m, 4H, C4H), 4.93 (s, 4H, C2H), 4.61 (s, 4H, N5H). Data in accordance with the literature.[2]

Bridge diamine S3

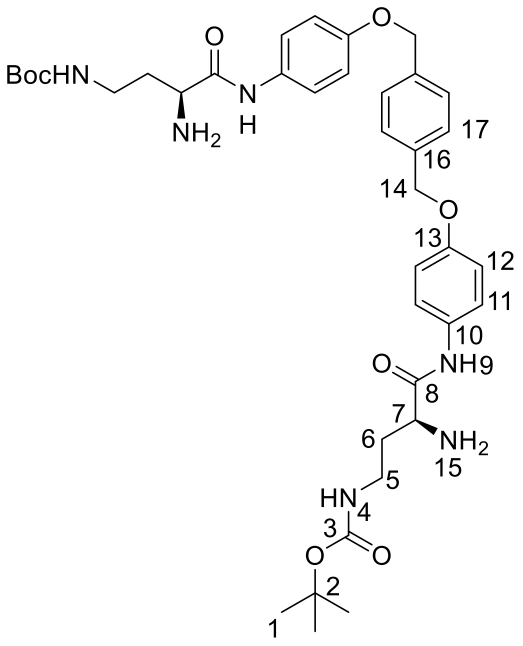

To a solution of commercially available Fmoc-Dab(Boc)-OH **20** (1.07 g, 2.44 mmol, 2.-5 eqv) in dry THF (50 ml) was added HBTU (992mg, 2.62 mmol, 2.2 eqv), Na_2CO_3 (822 mg, 5.95 mmol, 5.0 eqv) and 4,4'-((1,4-phenylenebis(methylene))bis(oxy))dianiline **19** (381 mg, 1.19 mmol, 1.0 eqv). The reaction mixture was stirred at 60 °C for 12 hours before the solvent was removed *in vacuo.* The residue was suspended in water (50 ml) and then filtered. The filtered cake was collected and dried in the oven to give intermediate compound **21** (1.22 g, 1.05 mmol, 88 %). Intermediate **21** was then suspended in MeOH (50 ml) and 1 M NaOH aq solution (20 ml) was added. The mixture was then refluxed for 2 hours before MeOH was removed *in vacuo*. The remaining aqueous phase was then filtered, and the cake was washed with water (3 x 20 ml) and hexane (3 x 20 ml). The filtered cake was collected and dried to give the title compound **S3** (498 mg, 691 µmol, 66 %) as a colourless oil.

^{1}H NMR (600 MHz, DMSO) δ 9.64 (s, 2H, N9H), 9.18 (s, 4H, N15H), 7.39 (d, *J* = 8.8 Hz, 4H, C11H), 7.27 (s, 4H, C17H), 6.77 (t, *J* = 5.6 Hz, 2H, N4H), 6.70 (d, *J* = 8.7 Hz, 4H, C12H), 3.69 (d, *J* = 13.4 Hz, 2H, C14H), 3.52 (d, *J* = 13.5 Hz, 2H, C14H), 3.11 (s, 2H, C7H), 3.04 (q, *J* = 7.3 Hz, 4H, C5H), 1.74 – 1.59 (m, 4H, C6H), 1.36 (s, 18H, C1H).**^{13}C NMR** (151 MHz, DMSO) δ 172.2 (C8), 155.5 (C3), 153.4 (C13), 138.8 (C16), 130.4 (C10), 127.8 (C17), 121.1 (C11), 115.0 (C12), 77.5 (C2), 59.9 (C7), 50.8 (C14), 37.4 (C5), 33.5 (C6), 28.3 (C1). **HRMS** for $C_{38}H_{53}N_6O_8^+$ $[M+H]^+$ Calculated m/z = 721.3919 Found m/z = 721.3913.

Bridge di-PFP ester S5

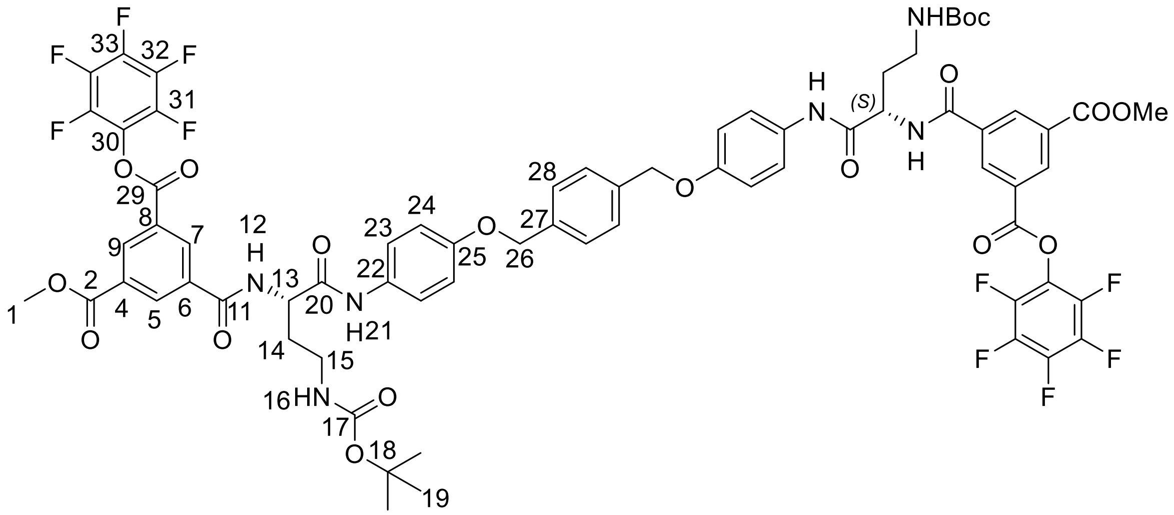

To a solution of bis(pentafluorophenyl) 5-(carboxymethyl)isophthalate **S4** (926 mg, 1.66 mmol, 4 eqv) in THF (30 ml) was added DIPEA (725 μL, 4.16 mmol, 10.0 eqv). A solution of bridge diamine **S3** (300 mg, 416 μmol, 1.0 eqv) in THF (10 ml) was added to the above solution over 1 hour by a syringe pump. The reaction was allowed to stir at room temperature for 4 hours after the completion of the addition. The solvent was removed *in vacuo*, and the residue was then purified by reverse phase column chromatography (Hexane : EtOAc = 15 : 85) to give the title compound **S5** (378 mg, 258 μmol, 62 %) as a white solid.

^{1}H NMR (500 MHz, $CDCl_3$) δ 9.93 (s, 2H, N21H), 8.93 (t, *J* = 1.6 Hz, 2H, C5H), 8.86 (t, *J* = 1.7 Hz, 2H, C9H), 8.76 (t, *J* = 1.5 Hz, 2H, C7H), 8.00 (d, *J* = 6.7 Hz, 2H, N12H), 7.54 (d, *J* = 9.0 Hz, 4H, C23H), 7.41 (s, 4H, C28H), 6.89 (d, *J* = 9.0 Hz, 4H, C24H), 5.32 – 5.16 (m, 2H, N16H), 5.05 (s, 6H, C1H), 4.89 – 4.81 (m, 2H, C13H), 3.61 (t, *J* = 11.5 Hz, 2H, C15H), 3.13 (dt, *J* = 14.9, 4.2 Hz, 2H, C15H), 2.16 – 2.09 (m, 2H, C14H), 2.07 – 2.00 (m, 2H, C14H), 1.48 (s, 18H, C19H). **^{13}C NMR** (126 MHz, $CDCl_3$) δ 169.0 (C20), 165.1 (C11), 164.5 (C2), 161.3 (C29), 158.1 (C17), 155.6 (C25), 136.9 (C27), 135.4 (C6), 134.6 (C9), 133.8 (C7), 133.4 (C5), 131.9 (C8), 131.5 (C4), 128.2 (C22), 127.8 (C28), 121.5 (C23), 115.4 (C24), 80.8 (C18), 70.1 (C26), 53.0 (C1), 51.7 (C13), 37.2 (C15), 35.1 (C14), 28.5 (C19). **HRMS** for $C_{70}H_{63}N_6O_{18}^+$ $[M+H]^+$ Calculated m/z = 1465.4039 Found m/z = 1465.4061.

Receptor 7

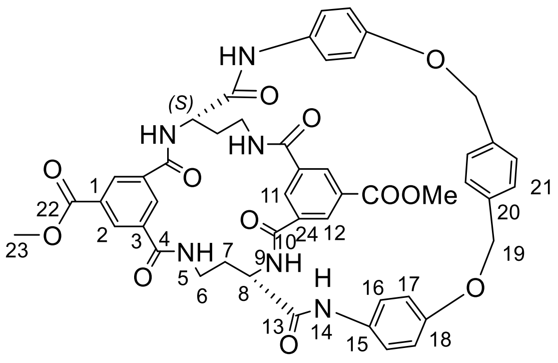

To a solution of di-PFP ester **S5** (375 mg, 256 μmol, 1.0 eqv) in DCM (6 ml) was added TFA (2 ml). The reaction was stirred at room temperature for 2 hours before the solvent was removed using a flow of N_2 to give bis-ammonium **S6** as the TFA salt. This material was dissolved in THF (10 ml) and placed in a syringe. A solution of DIPEA (223 μL, 1.28 mmol, 5.0 eqv) and TBACl (97 mg, 350 μmol, 5.0 eqv) in THF (250 ml) was prepared, and the solution in the syringe was added at room temperature over 24 hours. The solution was stirred under the same condition for a further 4 hours before the solvent was removed *in vacuo*. The residue was then purified by reverse (H_2O : Acetone = 40 : 60) and normal (DCM : MeOH = 75 : 25) phase column chromatography to give receptor **7** (90 mg, 100 μmol, 39 %) as a white solid.

^{1}H NMR (500 MHz, DMSO) δ 9.80 (s, 2H, N14H), 8.97 (t, *J* = 5.8 Hz, 2H, N5H), 8.52 (s, 2H, C11H), 8.32 (t, *J* = 1.5 Hz, 2H, C2H), 8.27 (d, *J* = 8.9 Hz, 2H, N9H), 8.24 (s, 2H, C12H), 7.48 (d, *J* = 9.0 Hz, 4H, C16H), 7.32 (s, 4H, C21H), 6.92 (d, *J* = 9.1 Hz, 4H, C17H), 5.25 (s, 4H, C19H), 4.87 (dt, *J* = 8.4, 4.0 Hz, 2H, C8H), 3.85 (s, 6H, C23H), 3.79 – 3.69 (m, 2H, C6H), 3.27 – 3.21 (m, 2H, C6H), 2.34 – 2.26 (m, 2H, C7H), 2.13 – 2.01 (m, 2H, C7H). **^{13}C NMR** (126 MHz, DMSO) δ 168.4 (C13), 164.9 (C22), 164.5 (C4), 164.5 (C10), 153.5 (C18), 136.7 (C20), 134.4 (C3), 133.6 (C24), 131.8 (C1), 131.3 (C2), 129.9 (C15), 129.6 (C11), 129.1 (C12), 126.8 (C21), 122.1 (C16), 115.0 (C17), 67.4 (C19), 52.4 (C23), 52.1 (C8), 36.4 (C6), 28.5 (C7). **HRMS** for $C_{48}H_{44}N_6O_{12}^+$ $[M+H]^+$ Calculated m/z = 897.3090 Found m/z = 897.3084.

1.3 Synthesis of receptors 8 and 9

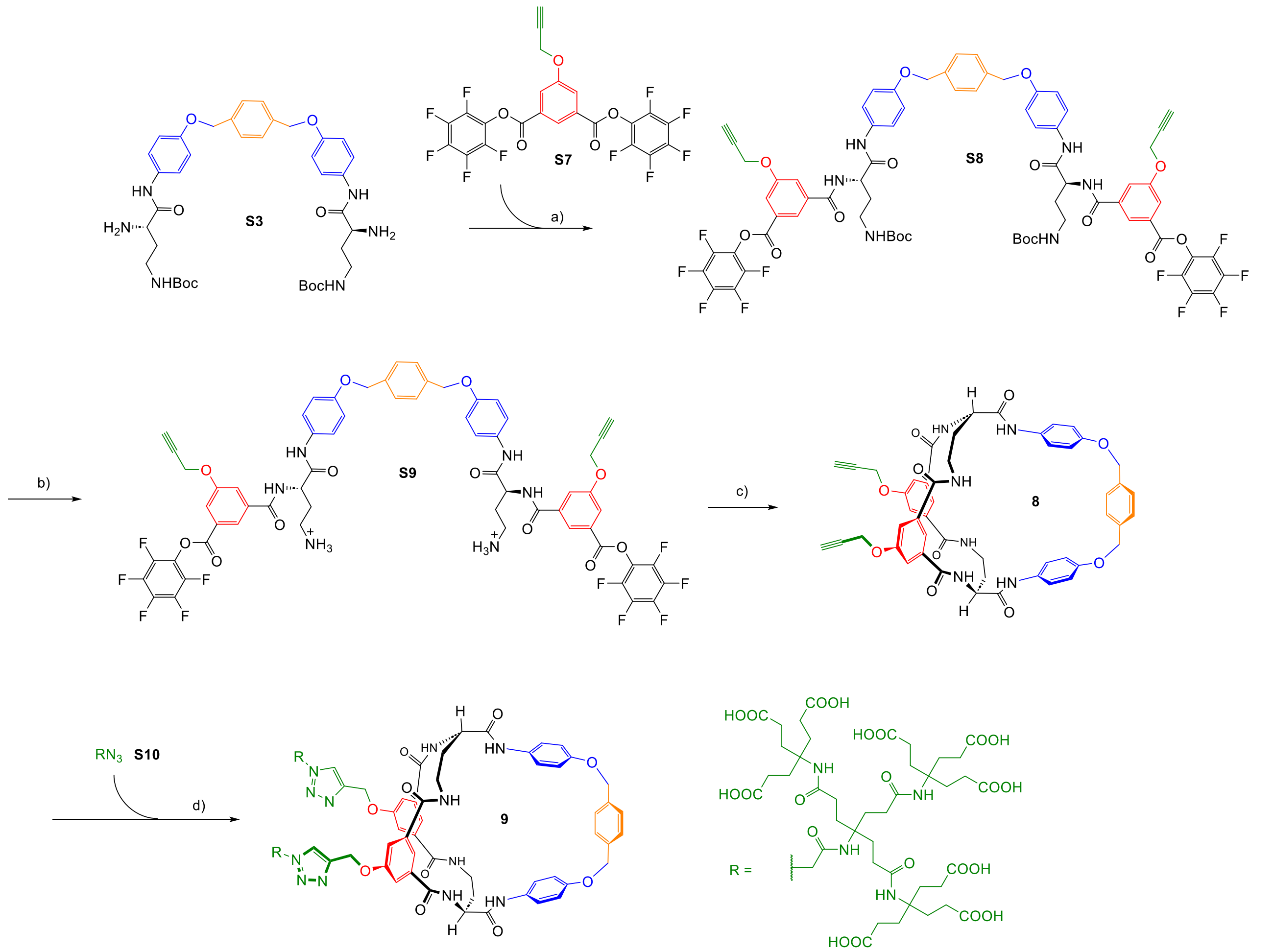

Scheme S2. a) K_2CO_3, DMF; b) TFA, DCM; c) HBTU, Na_2CO_3, THF; d) NaOH, MeOH; a) DIPEA, THF; b) TFA, DCM; c) TBACl, DIPEA, THF; d) G_2-N_3 $[Cu(CH_3CN)_4]PF_6$, 2,6-luditine, MeCN.

Bridge di-PFP ester S8

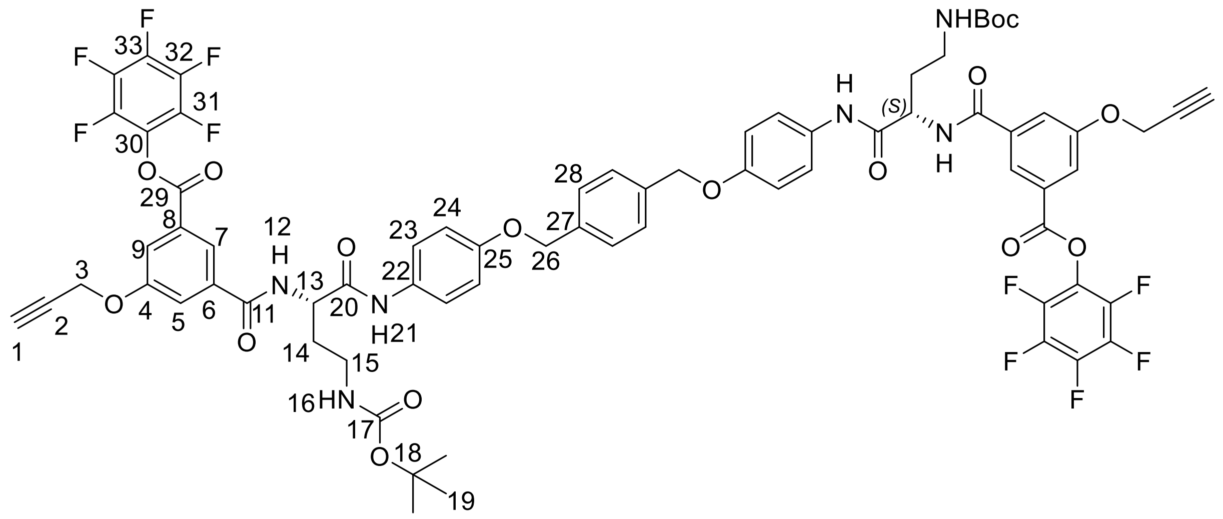

To a solution of bis(pentafluorophenyl)-5-(propargyloxy)isophthalate **S7**[3] (575 mg, 1.0 mmol, 5.0 eqv) in THF (30 ml) was added DIPEA (362 μL, 2.1 mmol, 10.0 eqv). A solution of bis(Boc-Dab-phenoxyphenyl) diamide **S3** (150 mg, 208 μmol, 1.0 eqv) in THF (10 ml) was added over 1 hour by a syringe pump. The reaction was stirred at room temperature for 4 hours after the completion of the addition. The solvent was removed *in vacuo*, and the residue was then purified by column chromatography (hexane:EtOAc = 2:98) to give the title compound **S8** (252 mg, 173 μmol, 83 %) as a white solid.

1H NMR (600 MHz, CDCl3) δ 8.64 (s, 2H, C7H), 8.11 – 8.07 (m, 2H, C9H), 8.04 – 8.00 (m, 2H, C5H), 7.71 (d, J = 8.4 Hz, 4H, C24H), 7.35 (d, J = 4.6 Hz, 4H, C28H), 7.20 (d, J = 8.5 Hz, 4H, C23H), 4.86 (d, J = 2.4 Hz, 4H, C3H), 3.80 (d, J = 7.9 Hz, 4H, C26H), 3.35 (s, 2H, C13H), 3.31 – 3.23 (m, 4H, C15H), 2.60 (t, J = 2.4 Hz, 2H, C1H), 1.94 (s, 4H, C14H), 1.42 (s, 18H, C19H). **13C NMR** (151 MHz, CDCl3) δ 163.9 (C20), 161.7 (C11), 158.1 (C29), 157.0 (C17), 146.8 (C4), 142.3 (C30), 140.6 (C32), 139.0 (C31), 137.3 (C33), 136.1 (C25), 132.1 (C27), 130.5 (C6), 129.0 (C28), 128.0 (C8), 125.3 (C7), 124.6 (C22), 122.6 (C5), 122.1 (C23), 121.9 (C9), 120.6 (C24), 80.0 (C18), 77.3 (C2), 77.0 (C1), 60.4 (C13), 56.6 (C3), 52.4 (C26), 37.2 (C15), 34.2 (C14), 28.5 (C19). **HRMS** $C_{72}H_{63}F_{10}N_6O_{16}^+$ $[M+H]^+$ Calculated m/z = 1457.4141 Found m/z = 1457.4164.

Receptor 8

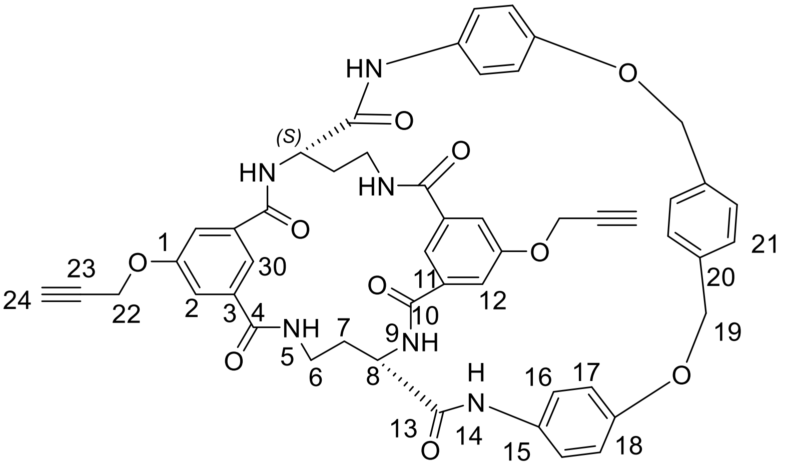

To a solution of bis-PFP ester **S8** (100 mg, 69 µmol, 1.0 eqv) in DCM (3 ml) was added TFA (1 ml). The reaction was stirred at room temperature for 2 hours before the solvent was removed using a flow of N_2 to give bis-ammonium **S9** as the TFA salt. This material was dissolved in THF (10 ml) and placed in a syringe. A solution of DIPEA (299 µL, 1.7 mmol, 25.0 eqv) and TBACl (95 mg, 343 µmol, 5.0 eqv) in THF (140 ml) was prepared, and the solution in the syringe was added at room temperature over 24 hours. The solution was then stirred at room temperature for a further 4 hours before the solvent was removed *in vacuo*. The residue was then purified by reverse (H_2O:acetone = 43:57) and normal (DCM:MeOH = 70:30) phase column chromatography to give receptor **8** (29 mg, 32 µmol, 47 %) as a white solid.

^{1}H NMR (600 MHz, DMSO) δ 9.72 (s, 2H, N14H), 8.72 (t, *J* = 5.7 Hz, 2H, N5H), 8.37 (d, *J* = 8.8 Hz, 2H, N9H), 8.02 (s, 2H, C30H), 7.50 – 7.46 (m, 2H, C2H), 7.45 – 7.41 (m, 6H, C12,16H), 7.28 (s, 4H, C21H), 6.86 (d, *J* = 9.1 Hz, 4H, C17H), 5.20 (s, 4H, C19H), 4.88 (dt, *J* = 8.6, 4.1 Hz, 2H, C8H), 4.75 (d, *J* = 2.3 Hz, 4H, C22H), 3.69 – 3.63 (m, 2H, C6H), 3.51 (t, *J* = 2.3 Hz, 2H, C24H), 3.27 – 3.22 (m, 2H, C6H), 2.37 – 2.30 (m, 2H, C7H), 1.89 – 1.82 (m, 2H, C7H). **^{13}C NMR** (151 MHz, DMSO) 168.7 (C13), 165.4 (C4), 164.5 (C10), 156.8 (C1), 153.5 (C18), 136.7 (C20), 135.0 (C3), 134.3 (C11), 131.8 (C15), 126.7 (C21), 122.2 (C16), 118.7 (C30), 116.8 (C2), 116.3 (C12), 115.0 (C17), 78.7 (C23), 78.6 (C24), 67.5 (C19), 55.8 (C22), 51.7 (C8), 36.5 (C6), 30.0 (C7). **HRMS** for $C_{50}H_{45}N_6O_{10}^+$ [M+H]$^+$ Calculated m/z = 889.3192 Found m/z = 889.3188

Receptor 9

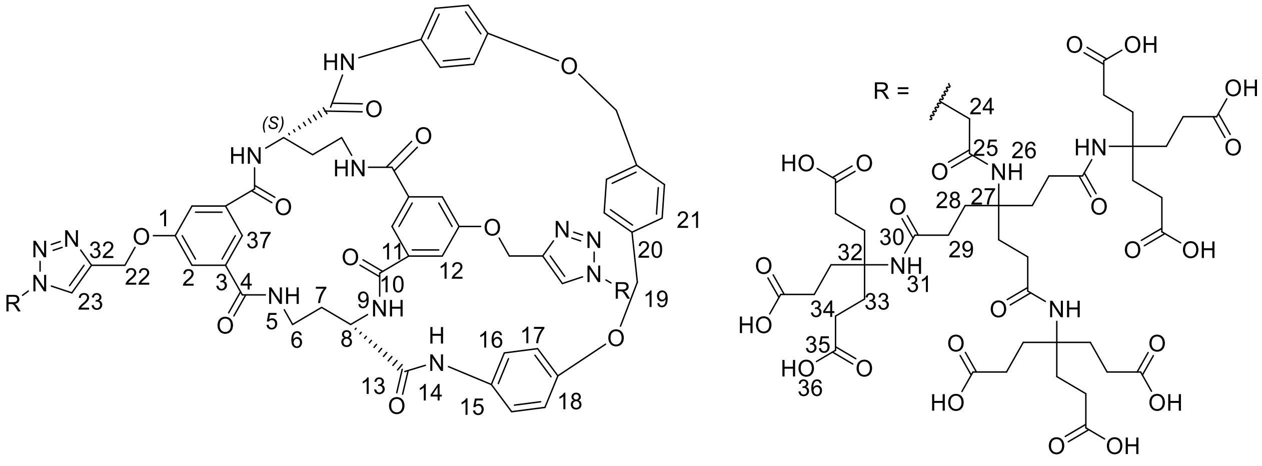

To a solution of bicycle **8** (20.0 mg, 22.5 μmol, 1.0 eqv) in de-gassed MeCN and DMF mixture (2 ml + 2 ml) was added azido nona-acid **S10**[3] (55.7 mg, 56.3 μmol, 2.5 eqv) and 2,6-lutidine (12.1 mg, 112.5 μmol, 5.0 eqv). The solution was stirred at room temperature for 15 minutes before tetrakis(acetonitrile)copper(I) hexafluorophosphate (16.8 mg, 45.0 μmol, 2.0 eqv) was added. The reaction mixture was stirred at 60 °C overnight before the solvent was removed in vacuo. The residue was then purified by reverse phase column chromatography (0.01 M aqueous HCl:MeCN = 45:55) to give receptor **9** (34.8 mg, 11.9 μmol, 53 %) as a colourless oil.

^{1}H NMR (600 MHz, DMSO) δ 12.03 (s, 18H, O36H), 9.74 (s, 2H, N14H), 8.88 (s, 2H, N9H), 8.68 – 8.53 (m, 4H, C37H, N5H), 8.11 (s, 2H, C23H), 7.79 (s, 2H, N26H), 7.59 (s, 2H, C12H), 7.52 (d, *J* = 8.5 Hz, 4H, C17H), 7.48 (s, 2H, C2H), 7.26 (s, 4H, C21H), 7.20 (s, 6H, N31H), 6.72 (d, *J* = 8.4 Hz, 4H, C16H), 5.26 – 5.18 (m, 4H, C19H), 5.16 (s, 4H, C22H), 5.08 (s, 4H, C24H), 4.92 (s, 2H, C8H), 2.40 – 2.33 (m, 2H, C7H), 2.26 – 2.22 (m, 2H, C7H), 2.15 – 2.00 (m, 48H, C29,34H), 1.90 – 1.72 (m, 48H, C28,33H). **^{13}C NMR** (151 MHz, DMSO) δ 174.5 (C35), 172.0 (C30), 169.2 (C13), 165.2 (C25), 164.7 (C4), 164.4 (C10), 158.1 (C1), 153.2 (C18), 142.0 (C32), 136.6 (C3), 136.3 (C11), 134.6 (C15), 132.0 (C20), 127.0 (C21), 126.1 (C23), 121.1 (C17), 117.0 (C2), 116.7 (C12), 116.0 (C16), 68.6 (C19), 61.3 (C22), 57.6 (C27), 56.3 (C32), 54.4 (C8), 51.8 (C24), 37.0 (C6), 30.9 (C28), 30.7 (C7), 30.1 (C29), 29.0 (C33), 28.1 (C34). **HRMS** for $C_{134}H_{172}N_{20}O_{54}^{2+}$ [M+2H]$^{2+}$ Calculated m/z = 1463.0680 Found m/z = 1463.0654

1.4 Synthesis of receptors 10 and 11

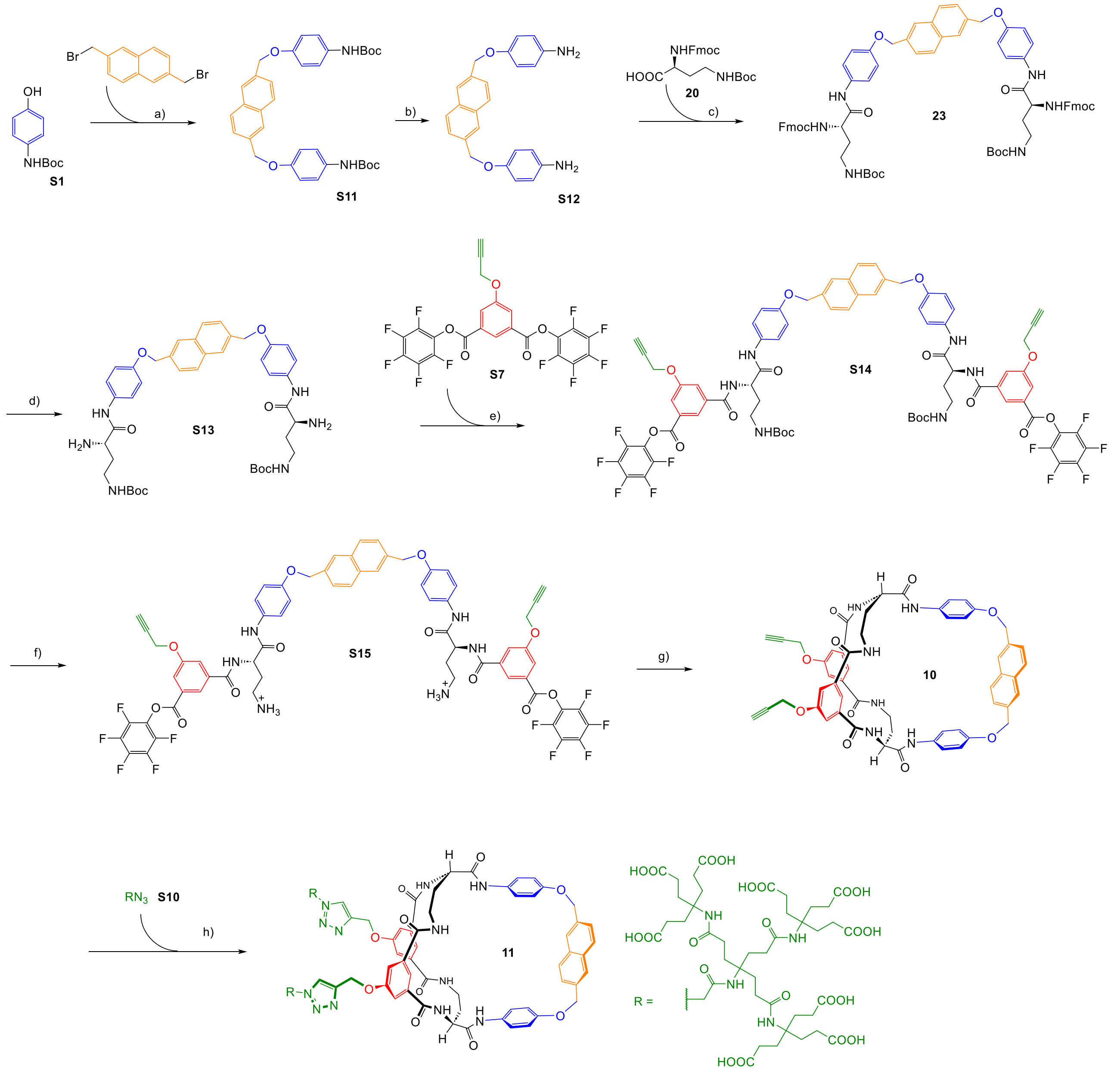

Scheme S3. a) K_2CO_3, DMF; b) TFA, DCM; c) HBTU, Na_2CO_3, THF; d) NaOH, MeOH; e) DIPEA, THF; f) TFA, DCM; g) TBACl, DIPEA, MeCN; h) **S10**, $[Cu(CH_3CN)_4]PF_6$, 2,6-luditine, MeCN.

Aryloxynaphthalene S11

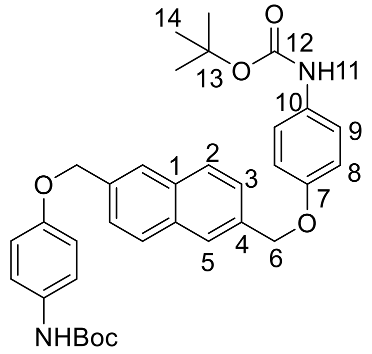

To a solution of 2,6-bis(bromomethyl)naphthalene (500 mg, 1.59 mmol, 1.0 eqv) in dry DMF (15 ml) was added *N*-Boc-4-hydroxyaniline **S1** (683 mg, 3.26 mmol, 2.1 eqv) and K_2CO_3 (880mg, 6.37 mmol, 4.0 eqv). The mixture was stirred at 100 °C for 12 hours. The solution was evaporated under reduced pressure, the resultant solid was suspended in water (50 ml), then filtered and washed with water (100 ml). The filtration cake was collected and dried in the oven to give the title compound **S11** (736 mg, 1.29 mmol, 81 %) as a brown solid.

^{1}H NMR (400 MHz, DMSO) δ 9.13 (s, 2H, N11H), 7.99 – 7.90 (m, 4H, C2,5H), 7.57 (d, *J* = 8.1 Hz, 2H, C3H), 7.34 (d, *J* = 7.9 Hz, 4H, C9H), 6.96 (d, *J* = 7.9 Hz, 4H, C8H), 5.21 (s, 4H, C6H), 1.45 (s, 18H, C14H). **^{13}C NMR** (101 MHz, DMSO) δ 153.5 (C12), 152.9 (C7), 135.1 (C4), 132.9 (C1), 132.3 (C10), 128.0 (C2), 126.0 (C5, C3), 119.6 (C8), 114.9 (C9), 78.7 (C13), 69.5 (C6), 28.2 (C14). **HRMS** for $C_{34}H_{38}N_2O_6Na^+$ $[M+Na]^+$ Calculated m/z = 593.2622 Found m/z = 593.2623.

Diamine S12

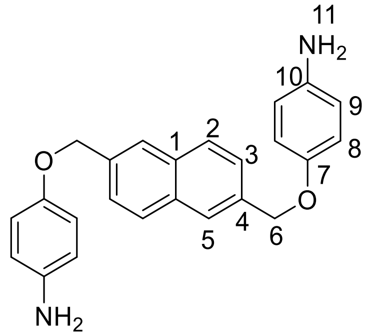

To a solution of 4M HCl in dioxane (10 ml) was added **S11** (500 mg, 0.88 mM), the reaction was stirred at 60 °C for 12 hours. The solution was evaporated under reduced pressure, the resultant solid was dissolved in EtOAc (20 ml) washed with sat. aq. $NaHCO_3$ (3 x 10 ml), and then brine. The organic phase was evaporated to give diamine **S12** (308 mg, 0.83 mM, 95 %) as white solid.

^{1}H NMR (400 MHz, DMSO) δ 7.95 – 7.88 (m, 4H, C2,5H), 7.55 (d, J = 9.3 Hz, 2H, C3H), 6.76 (d, J = 8.8 Hz, 4H, C8H), 6.50 (d, J = 8.8 Hz, 4H, C9H), 5.11 (s, 4H, C6H), 4.62 (s, 4H, N11H). **^{13}C NMR** (126 MHz, DMSO) δ 149.7 (C7), 142.7 (C10), 135.5 (C4), 132.2 (C1), 127.9 (C2), 126.0 (C5), 125.8 (C3), 115.8 (C8), 114.9 (C9), 70.0 (C6). **HRMS** for $C_{24}H_{22}N_2O_2Na^+$ $[M+Na]^+$ Calculated m/z = 393.1579 Found m/z = 353.1584

Bis-pentafluorophenyl ester S14

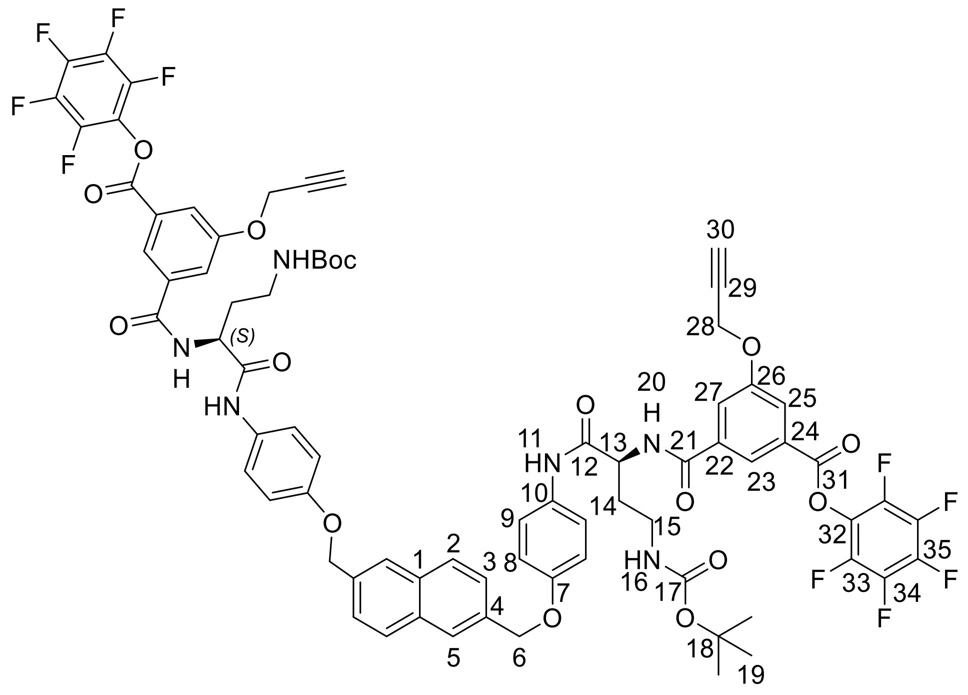

To a solution of commercially available Fmoc-Dab(Boc)-OH **20** (829 mg, 1.88 mmol, 2.1 eqv) in dry MeCN (30 ml) was added HBTU (870 mg, 2.29 mmol, 2.5 eqv), K_2CO_3 (507 mg, 3.67 mmol, 4.0 eqv) and diamine **S12** (340 mg, 0.92 mmol, 1.0 eqv). The reaction mixture was stirred at 60 °C for 12 hours before the solvent was removed *in vacuo.* The residue was suspended in water (50 ml), then filtered and washed with water (100 ml). The filtration cake was collected and dried in the oven to give intermediate **23** (1.0 g, 0.84 mmol, 92 %) as a brown solid.

Intermediate **23** (1.0 g, 0.84 mmol, 1.0 eqv) was suspended in MeOH (30 ml) before 1 M NaOH aq solution (10 ml) was then added. The mixture was refluxed for 2 hours then concentrated in vacuo. The remaining aqueous phase was then filtered, and the cake was washed with water (3 x 20 ml) and then hexane (3 x 20 ml). The filtration cake was collected and purified by reverse phase column chromatography (H_2O:MeCN = 30 : 70) to give diamine **S13** (320 mg, 0.42 mmol, 45 %) as a colourless gum.

To a solution of bis(pentafluorophenyl)-5-(propargyloxy)isophthalate **S7** (1.15 g, 2.1 mmol, 5.0 eqv) and diamine **S13** (320 mg, 0.42 mmol, 1.0 eqv) in MeCN and DMF mixture (20 ml + 5 ml) was added DIPEA (362 μL, 2.1 mmol, 5.0 eqv). The reaction was stirred at room temperature for 4 hours before the solvent was removed *in vacuo*. The residue was then purified by reverse phase (Water:MeCN = 5:95) and normal phase column chromatography (hexane:EtOAc = 10:90) to give the title compound **S14** (407 mg, 270 μmol, 65 %) as a white solid.

^{1}H NMR (500 MHz, $CDCl_3$) δ 9.89 (s, 2H, N11H), 8.28 (s, 2H, C23H), 7.99 (s, 2H, N20H), 7.90 – 7.76 (m, 8H, C2,5,8,27H), 7.54 – 7.45 (m, 6H, C3,25H), 6.93 (d, *J* = 8.3 Hz, 4H, C9H), 5.33 (s, 2H, N16H), 5.16 (s, 4H, C6H), 4.86 (s, 2H, C13H), 4.77 (s, 4H, C28H), 3.63 – 3.50 (m, 2H, C15H), 3.15 (s, 2H, C15H), 2.55 (s, 2H, C30H), 2.18 – 2.07 (m, 4H, C14H), 1.43 (s, 18H, C19H). **^{13}C NMR** (126 MHz, $CDCl_3$) δ 171.3 (C12), 169.3 (C21), 161.8 (C31), 158.1 (C26), 157.9 (C7), 155.7 (C17), 142.3 (C32), 140.3 (C34), 139.0 (C35), 137.0 (C33), 136.2 (C4),

134.8 (C24), 133.0 (C22), 131.5 (C1), 128.7 (C10), 128.5 (C5), 126.2 (C2), 125.7 (C3), 122.1 (C23), 121.6 (C8), 120.3 (C25), 120.1 (C27), 115.3 (C9), 80.5 (C18), 76.8 (C30), 70.5 (C6), 56.5 (C28), 51.8 (C13), 37.2 (C15), 34.7 (C14), 28.5 (C19). **HRMS** for $C_{76}H_{67}N_6O_{16}F_{10}^{2+}$ $[M+2H]^{2+}$ Calculated m/z = 1507.4297 Found m/z = 1507.4292

Receptor 10

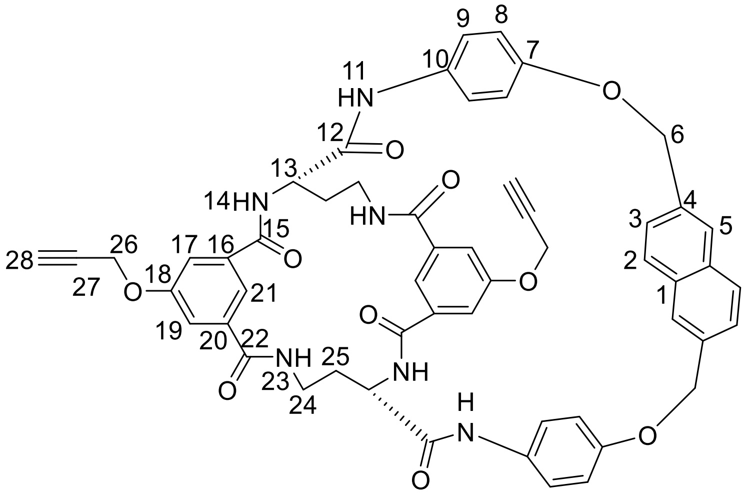

To a solution of **S14** (100 mg, 70 μmol, 1.0 eqv) in MeCN (3 ml) was added 4N HCl in dioxane (3 ml). The reaction was stirred at room temperature overnight before the solvent was removed i*n vacuo* to give bis-ammonium **S15** as the chloride salt. This material was dissolved in MeCN (10 ml) and placed in a syringe. Separately, a solution of DIPEA (289 μL, 1.66 mmol, 25.0 eqv) and TBACl (92 mg, 332 μmol, 5.0 eqv) in MeCN (140 ml) was prepared in a round bottomed flask. The solution in the syringe was added slowly to the round bottomed flask at room temperature over 24 hours. The solution was then stirred at room temperature for 4 hours before the solvent was removed *in vacuo*. The residue was then purified by reverse (H_2O:acetone = 23:77) and normal (DCM:MeOH = 75:25) phase column chromatography to give receptor **10** (30 mg, 18 μmol, 50%) as a white solid.

^{1}H NMR (500 MHz, DMSO) δ 9.67 (s, 2H, N11H), 8.70 (t, *J* = 5.5 Hz, 2H, N23H), 8.49 (d, *J* = 8.4 Hz, 2H, N14H), 8.11 (s, 2H, C21H), 7.82 (s, 2H, C5H), 7.80 (d, *J* = 8.4 Hz, 2H, C2H), 7.51 – 7.43 (m, 6H, C3,17,19H), 7.34 (d, *J* = 9.1 Hz, 4H, C8H), 6.86 (d, *J* = 9.1 Hz, 4H, C9H), 5.44 – 5.36 (m, 4H, C6H), 4.87 – 4.80 (m, 2H, C13H), 4.78 (d, *J* = 2.3 Hz, 4H, C26H), 3.57 – 3.51 (m, 4H, C18,24H), 3.30 – 3.26 (m, 2H, C24H), 2.39 – 2.32 (m, 2H, C25H), 2.02 – 1.93 (m, 2H, C25H). **^{13}C NMR** (126 MHz, DMSO) δ 168.9 (C12), 165.2 (C15), 164.4 (C22), 156.9 (C18), 153.4 (C7), 135.2 (C16), 134.9 (C20), 134.4 (C4), 132.0 (C1), 131.5 (C10), 128.0 (C2), 125.8 (C5), 125.2 (C3), 122.1 (C8), 118.8 (C21), 116.7 (C17), 116.3 (C19), 115.7 (C9), 78.7 (C27), 78.6 (C28), 68.6 (C6), 55.8 (C26), 52.3 (C13), 36.6 (C24), 30.3 (C25). **HRMS** for $C_{54}H_{48}N_6O_{10}^{+}$ $[M+H]^{+}$ Calculated m/z = 939.3354 Found m/z = 939.3367

Receptor 11

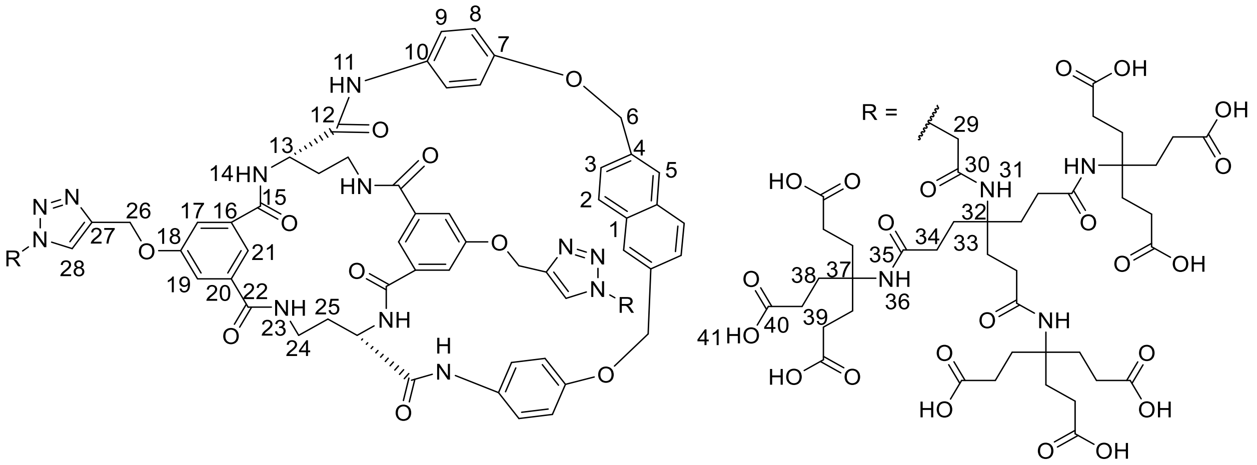

To a solution of bicycle **10** (21.0 mg, 22.5 μmol, 1.0 eqv) in de-gassed MeCN and DMF mixture (2 ml + 2 ml) was added azido nona-acid **S10**[3] (55.7 mg, 56.3 μmol, 2.5 eqv) and 2,6-lutidine (12.1 mg, 112.5 μmol, 5.0 eqv). The solution was stirred at room temperature for 15 minutes before tetrakis(acetonitrile)copper(I) hexafluorophosphate (16.8 mg, 45.0 μmol, 2.0 eqv) was added. The reaction mixture was stirred at 60 °C overnight before the solvent was removed in vacuo. The residue was then purified by reverse phase column chromatography (0.01 M aqueous HCl:MeCN = 40:60) to give receptor **11** (37.4 mg, 12.6 μmol, 59 %) as a colourless oil.

^{1}H NMR (600 MHz, DMSO) δ 9.68 (s, 2H, N11H), 8.85 (s, 2H, N14H), 8.76 – 8.66 (m, 4H, N23H, C21H), 8.10 (s, 2H, C28H), 7.83 – 7.77 (m, 6H, C2,5H, N31H), 7.57 (s, 2H, C17H), 7.54 (s, 2H, C19H), 7.44 (d, *J* = 8.6 Hz, 2H, C3H), 7.36 (d, *J* = 8.9 Hz, 4H, C8H), 7.21 (s, 6H, N36H), 6.83 (d, *J* = 9.0 Hz, 4H, C9H), 5.45 – 5.35 (m, 4H, C6H), 5.15 (s, 4H, C26H), 5.07 (s, 4H, C29H), 4.95 – 4.87 (m, 2H, C13H), 2.37 – 2.28 (m, 2H, C25H), 2.15 – 1.99 (m, 48H, C29,34H), 1.82 (s, 48H, C28,33H). **^{13}C NMR** (151 MHz, DMSO) δ 174.5 (C40), 172.0, 168.9, 164.8, 164.4, 158.2, 153.2, 142.0, 135.2, 134.7, 132.0, 131.6, 128.2 (C2), 126.1 (C28), 126.0 (C5), 125.3 (C3), 121.6 (C8), 117.0 (C17), 116.8 (C19), 116.0 (C9), 68.8 (C6), 61.4 (C26), 57.6, 56.3, 53.6 (C13), 51.8 (C29), 40.4, 37.3 (C24), 30.9 (C28), 30.7 (C25), 30.1 (C29), 29.0 (C33), 28.1 (C34). **HRMS** $C_{138}H_{174}N_{20}O_{54}^{2+}$ $[M+2H]^{2+}$ Calculated m/z = 1488.0758 Found m/z = 1488.0757.

1.5 Synthesis of receptors 12 and 13

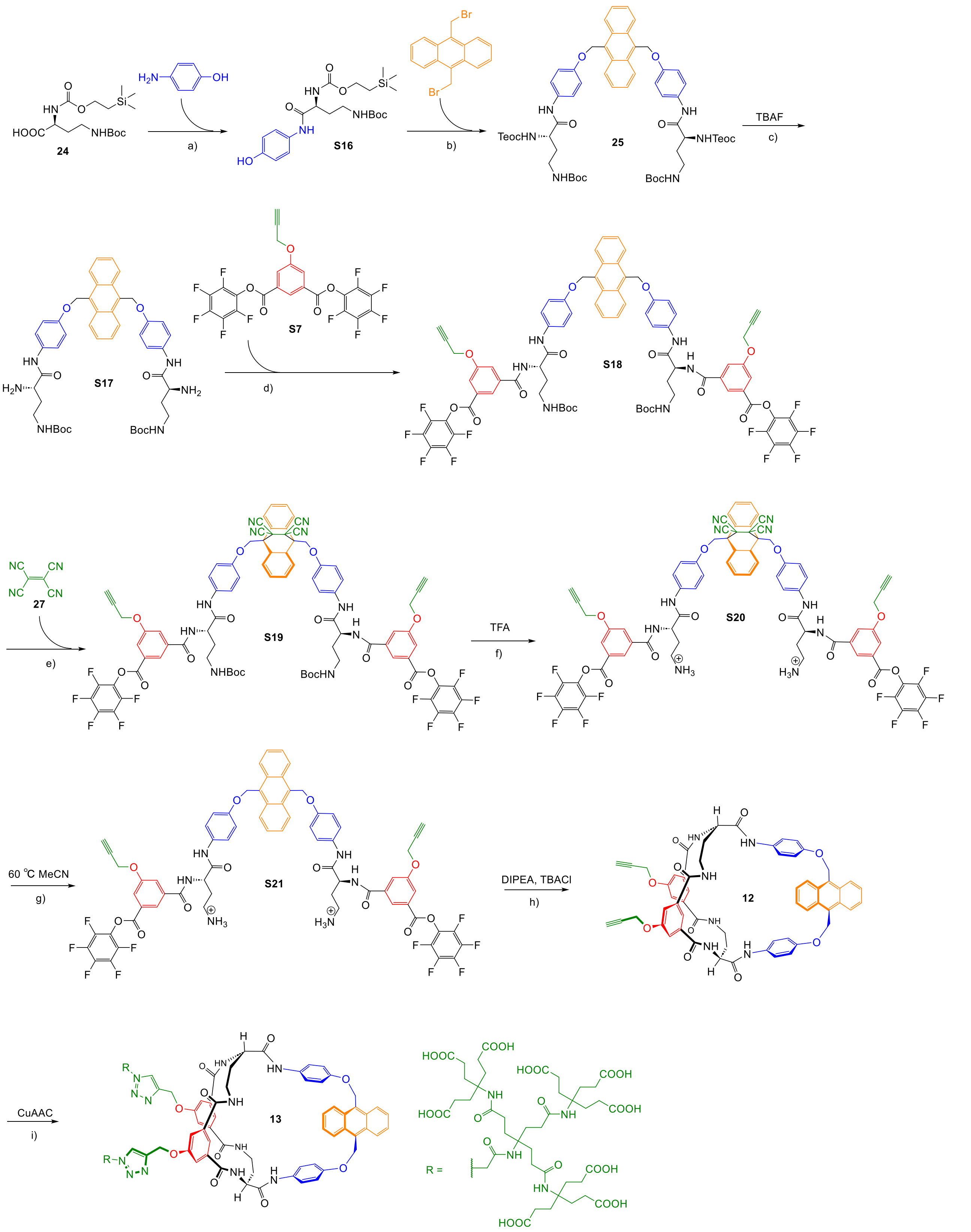

Scheme S4. a) HBTU, K_2CO_3, THF; b) K_2CO_3, MeCN; c) TBAF; d) DIPEA, THF; e) $CHCl_3$; f) TFA, DCM; g) MeCN; h) TBACl, DIPEA, MeCN; i) G_2-N_3, $[Cu(CH_3CN)_4]PF_6$, 2,6-luditine, MeCN.

Trial Experiments for Diels-Alder and retro Diels-Alder reactions

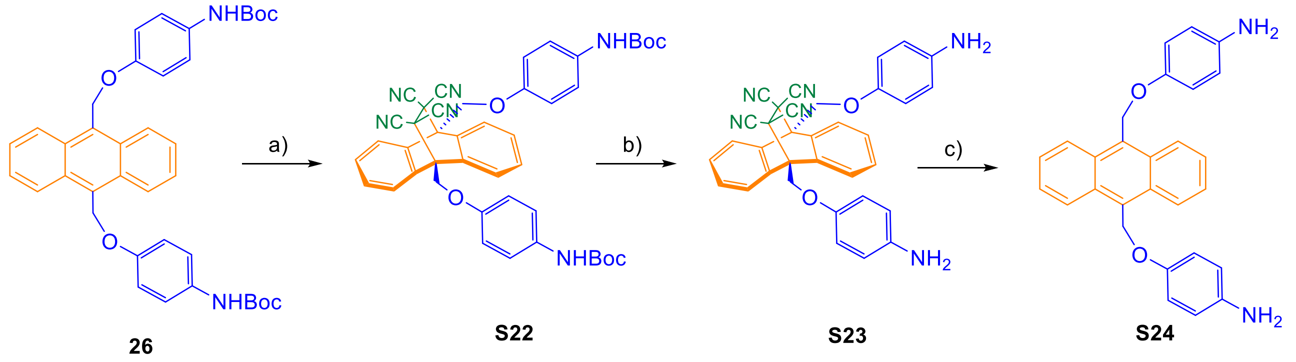

Scheme S5. a) TCNE, DCM; b) TFA, DCM; c) DMSO-*d*[6].

Bis-aryloxymethylanthracene 26

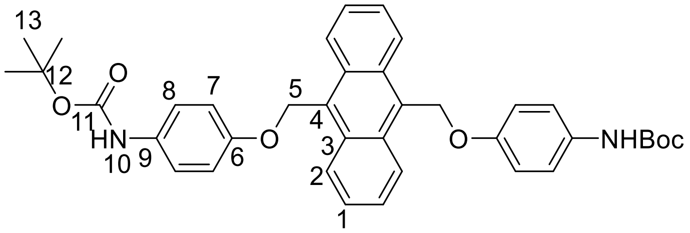

To a solution of 9,10-Bis(bromomethyl)anthrathene (500 mg, 1.37 mmol, 1.0 eqv) in MeCN (30 ml) was added *N*-Boc-4-hydroxyaniline (578 mg, 2.76 mmol, 2.01 eqv) and K_2CO_3 (759 mg, 5.49 mmol, 4.0 eqv). The mixture was stirred at 60 °C for 12 hours. The solution was evaporated under reduced pressure, the resultant solid was suspended in water (50 ml), then filtered and washed with water (100 ml). The filtration cake was collected and dried in the oven to give anthracene **26** (767 mg, 1.24 mmol, 90 %) as a bright yellow solid.

^{1}H NMR (500 MHz, DMSO) δ 9.17 (s, 2H, N10H), 8.41 (dd, *J* = 6.9, 3.3 Hz, 4H, C2H), 7.62 (dd, *J* = 6.9, 3.1 Hz, 4H, C1H), 7.40 (d, *J* = 8.0 Hz, 4H, C8H), 7.09 (d, *J* = 9.0 Hz, 4H, C7H), 6.02 (s, 4H, C5H), 1.48 (s, 18H, C13H). **^{13}C NMR** (126 MHz, DMSO) δ 154.0 (C11), 153.0 (C6), 133.1 (C4), 130.2 (C9), 129.6 (C3), 126.3 (C1), 125.3, 124.9 (C2), 119.7 (C8), 115.1 (C7), 78.7 (C12), 62.6 (C5), 28.2 (C13). **HRMS** for $C_{38}H_{40}N_2O_6$ $[M+Na]^+$ Calculated m/z = 643.2779 Found m/z = 643.2778.

TCNE adduct S22

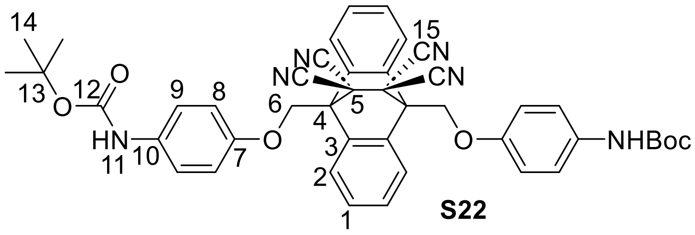

To a solution of **26** (Ar = Ar$_1$) (670 mg, 1.08 mmol, 1.0 eqv) in DCM (15 ml) was added tetracyanoethylene **27** (207 mg, 1.61 mmol, 1.5 eqv). Upon addition of the dienophile, the colour of the reaction changed from bright

yellow to dark green, suggesting a charge transfer complex forming between tetracyanoethylene and the electron-rich aromatics.[4] The mixture was stirred at room temperature for 1 hours before the solvent was removed *in vacuo*. The residue was purified by column chromatography (DCM : MeOH = 70 : 30) to give adduct **S22** as a colourless solid (694 mg, 0.93 mmol, 75 %).

^{1}H NMR (500 MHz, DMSO) δ 9.27 (s, 2H, N11H), 7.76 (dd, *J* = 5.6, 3.3 Hz, 4H, C2H), 7.54 (dd, *J* = 5.8, 3.2 Hz, 4H, C1H), 7.50 (d, *J* = 8.6 Hz, 4H, C9H), 7.26 (d, *J* = 9.1 Hz, 4H, C8H), 5.44 (s, 4H, C6H), 1.49 (s, 18H, C14H). **^{13}C NMR** (126 MHz, DMSO) δ 153.0 (C12), 152.7 (C7), 135.3 (C3), 134.1 (C10), 129.2 (C1), 125.5 (C2), 119.8 (C9), 115.0 (C8), 110.8 (C15), 78.9 (C13), 65.7 (C6), 52.9 (C4), 50.6 (C5), 28.2 (C14). **HRMS** for $C_{44}H_{40}N_6O_6$ $[M+Na]^+$ Calculated m/z = 771.2902 Found m/z = 711.2909.

Diamino TCNE adduct S23

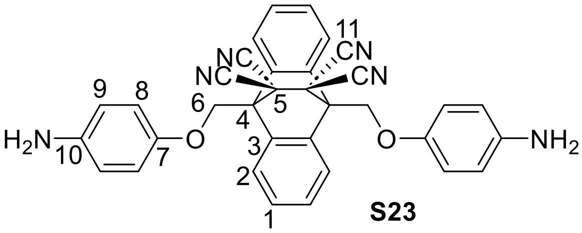

S23

To a solution of TCNE adduct **S22** (690 mg, 0.92 mmol, 1.0 eqv) in DCM (3 ml) was added TFA (1 ml). The reaction was stirred at 0 °C for 30 mins before the solvent was removed by N_2. The residue was dissolved in EtOAc (30 ml) and washed with Sat. $NaHCO_3$ (3 * 20ml) and then brine (10 ml). The organic phase was concentrated to give diamine **S23** (550 mg, 0.85 mmol, 92 %) as a brown oil.

^{1}H NMR (400 MHz, DMSO) δ 7.85 – 7.76 (m, 4H, C2H), 7.62 – 7.53 (m, 4H, C1H), 7.46 (d, *J* = 9.0 Hz, 4H, C9H), 7.41 (d, *J* = 8.9 Hz, 4H, C8H), 5.55 (s, 4H, C6H). **^{13}C NMR** (126 MHz, DMSO) δ 156.4 (C7), 135.2 (C3), 129.3 (C1), 125.5 (C2), 124.3 (C10), 124.1 (C8), 116.0 (C9), 110.9 (C15), 65.9 (C6), 52.9 (C4), 50.7 (C5).

Diamine S24

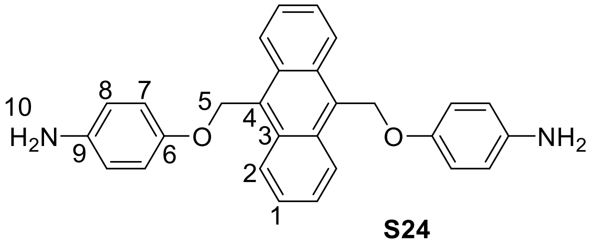

S24

In an NMR tube, compound **S23** (25 mg) was dissolved in DMSO-*d*[6] (0.5 ml). The tube was heated to 60 °C for 4 hours, before the colour of the solution changed from colourless to light yellow. ^{1}H NMR of the sample was acquired, indicating the completion of the retro Diels-Alder reaction with a yield of ~ 90 % (see Fig. S1).

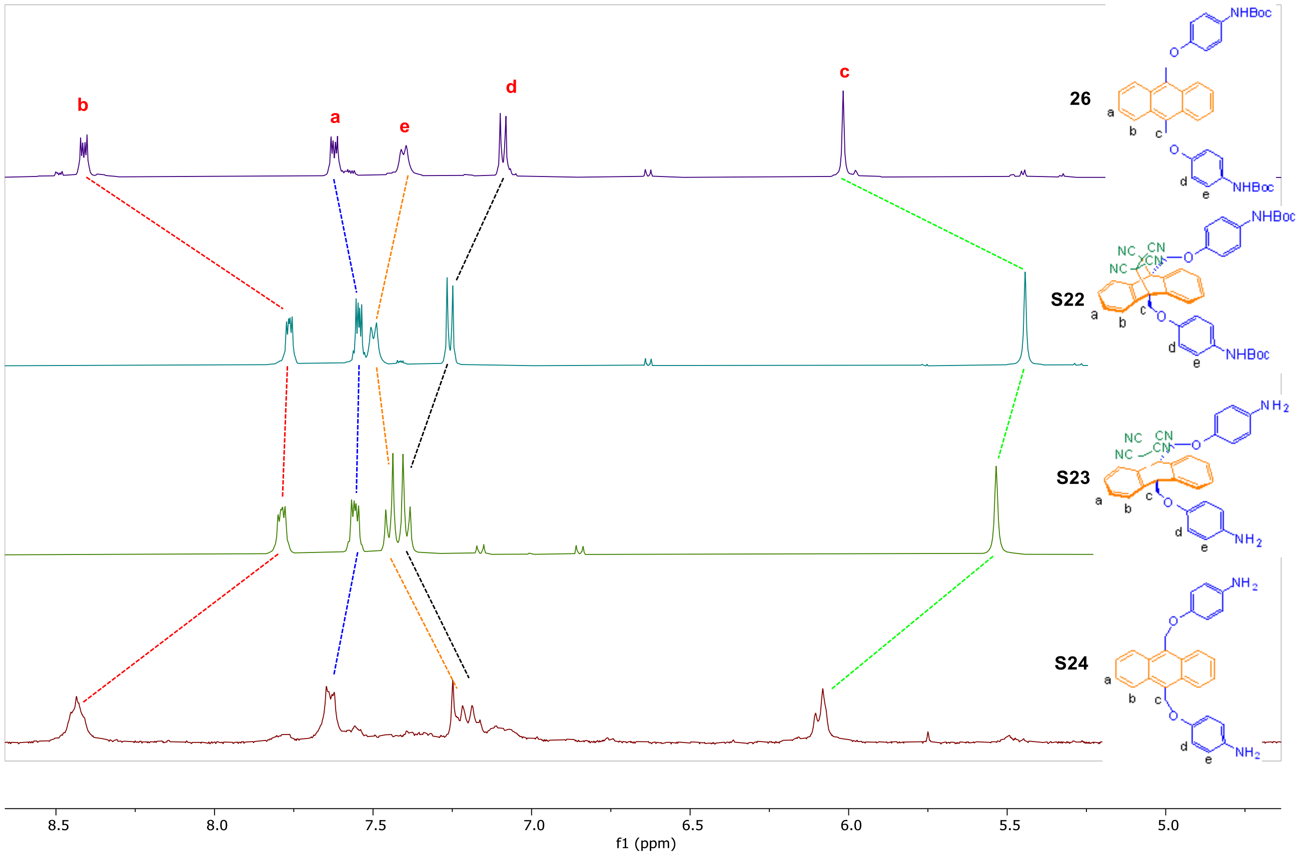

Figure S1. Overlapping of ^{1}H NMR (400 MHz and 500 MHz, DMSO-*d*[6]) for Diels-Alder and retro Diels-Alder reactions.

Hydroxyanilide S16

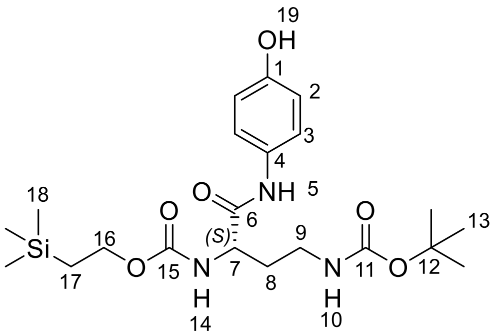

To a solution of Teoc-Dab(Boc)-OH **24** (411 mg, 1.13 mmol, 1.0 eqv) in THF (30 ml) was added 4-aminophenol (148 mg, 1.36 mmol, 1.2 eqv), HBTU (516 mg, 1.36 mmol, 1.2 eqv) and K_2CO_3 (143 mg, 1.72 mmol, 1.5 eqv). The reaction mixture was stirred at 60 °C overnight before the solvent was removed *in vacuo*. The residue was dissolved in DCM (20 ml), washed with water (3 x 10 ml) and dried over $MgSO_4$. The residue was then purified by column chromatography (Hexane: EtOAc = 45 : 55) to give anilide **S16** (360 mg, 0.19 mmol, 75 %) as a white solid.

^{1}H NMR (500 MHz, DMSO) δ 9.66 (s, 1H, N5H), 9.18 (s, 1H, O19H), 7.35 (d, *J* = 8.8 Hz, 2H, C3H), 7.25 (d, *J* = 8.0 Hz, 1H, N14H), 6.72 (t, *J* = 5.6 Hz, 1H, N10H), 6.68 (d, *J* = 8.8 Hz, 2H, C2H), 4.12 – 3.98 (m, 3H, C7,16H), 3.01 – 2.93 (m, 2H, C9H), 1.83 – 1.73 (m, 1H, C8H), 1.73 – 1.62 (m, 1H, C8H), 1.37 (s, 9H, C13H), 0.92 (d, *J* = 8.4 Hz, 2H, C17H), 0.02 (d, *J* = 2.8 Hz, 9H, C18). **^{13}C NMR** (126 MHz, DMSO) δ 170.0 (C6), 156.2 (C15), 155.5 (C11), 153.4 (C1), 130.5 (C4), 120.9 (C3), 115.0 (C2), 77.6 (C12), 61.8 (C16), 53.1 (C7), 37.0 (C9), 32.1 (C8), 28.2 (C13), 17.4 (C17), -1.4 (C18). **HRMS** for $C_{21}H_{35}N_3O_6Si$ $[M+H]^+$ Calculated m/z = 454.2373 Found m/z = 454.2377.

Intermediate 25

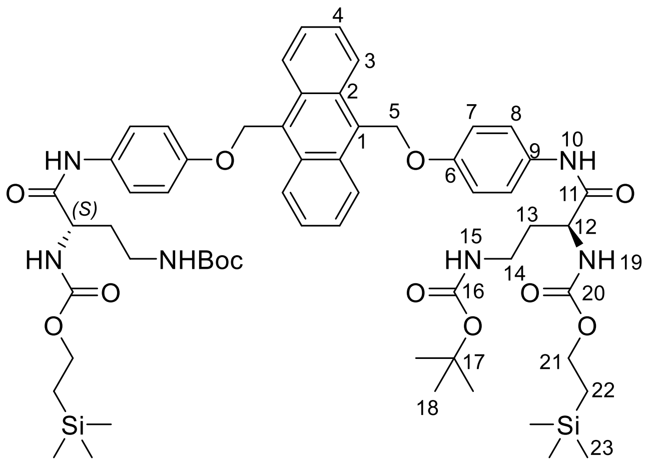

To a solution of **S16** (200 mg, 0.44 mmol, 2.0 eqv) in MeCN (25 ml) was added K_2CO_3 (91 mg, 0.66 mmol, 3.0 eqv). The mixture was stirred at 65 °C for 30 min before 9,10-bis(bromomethyl)anthracene (80 mg, 0.22 mmol, 1.0 eqv) was added. The reaction was stirred at 85 °C for 1 hour before the solvent was removed *in vacuo*. The residue was washed with water (3 x 10 ml) to give **25** (191 mg, 0.17 mmol, 78 %) as a yellow solid.

^{1}H NMR (500 MHz, DMSO) δ 9.88 (s, 2H, N10H), 8.40 (dd, *J* = 6.8, 3.2 Hz, 4H, C3H), 7.65 – 7.55 (m, 8H, C4H, C8H), 7.31 (d, *J* = 7.7 Hz, 2H, N19H), 7.15 (d, *J* = 8.8 Hz, 4H, C7H), 6.75 (s, N15H), 6.02 (s, 4H, C5H), 4.18 – 4.12 (m, 2H, C12H), 4.09 – 4.01 (m, 4H, C21H), 3.06 – 2.98 (m, 4H, C14), 1.90 – 1.67 (m, 4H, C13H), 1.38 (s, 18H, C18H), 0.98 – 0.91 (m, 4H, C22H), 0.03 (s, 18H, C23H). **^{13}C NMR** (126 MHz, DMSO) δ 170.7 (C11), 156.7 (C16), 156.0 (C20), 155.4 (C6), 132.9 (C9), 130.7 (C1), 129.9 (C2), 126.7 (C4), 125.3 (C3), 121.2 (C8), 115.5 (C7), 78.1 (C17), 63.0 (C5), 62.3 (C21), 53.5 (C12), 37.5 (C14), 32.5 (C13), 28.7 (C18), 17.9 (C22), -1.0 (C23). **HRMS** for $C_{58}H_{80}N_6O_{12}Si_2$ $[M+H]^+$ Calculated m/z = 1109.5446 Found m/z = 1109.5453.

Diamine S17

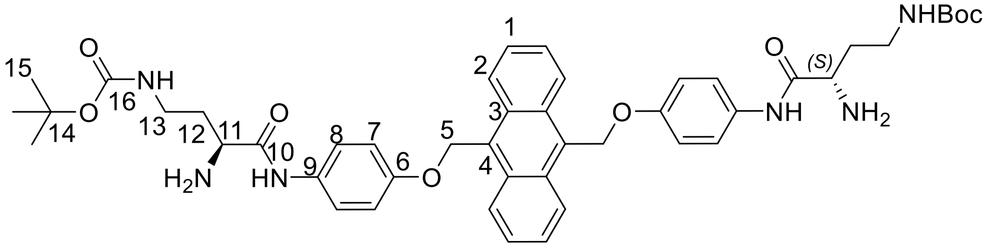

To a solution of **25** (140 mg, 126 µmol, 1.0 eqv) in THF (5 ml) was added 1M TBAF THF solution (265 µL, 265 µmol, 2.1 eqv). The reaction mixture was stirred at 60 °C for 3 hours before the solvent was removed *in vacuo*. The residue was purified by normal phase column chromatography (EtOAc : MeOH = 75 : 25) to give the diamine **S17** (90 mg, 115 µmol, 94 mg) as a yellow solid.

^{1}H NMR (500 MHz, MeOD) δ 8.40 (dd, *J* = 6.9, 3.2 Hz, 4H, C2H), 7.60 – 7.54 (m, 8H, C1, C8H), 7.15 (d, *J* = 8.9 Hz, 4H, C7H), 6.05 (s, 4H, C5H), 3.50 – 3.44 (m, 2H, C11H), 3.23 – 3.23 (m, 4H, C13H), 2.00 – 1.90 (m, 2H, C12H), 1.80 – 1.70 (m, 2H, C12H), 1.44 (s, 18H, C15H). **^{13}C NMR** (126 MHz, MeOD) δ 175.3 (C10), 158.7 (C16), 157.5 (C6), 133.1 (C9), 132.1 (C4), 130.8 (C3), 127.2 (C1), 125.9 (C2), 123.1 (C8), 116.3 (C7), 80.2 (C14), 64.3 (C5), 54.5 (C11), 49.6, 38.1 (C13), 36.8 (C12), 30.9, 28.8 (C15). **HRMS** for $C_{46}H_{56}N_6O_8$ $[M+H]^+$ Calculated m/z = 821.4232 Found m/z = 821.4237.

Bis-pentafluorophenyl ester S18

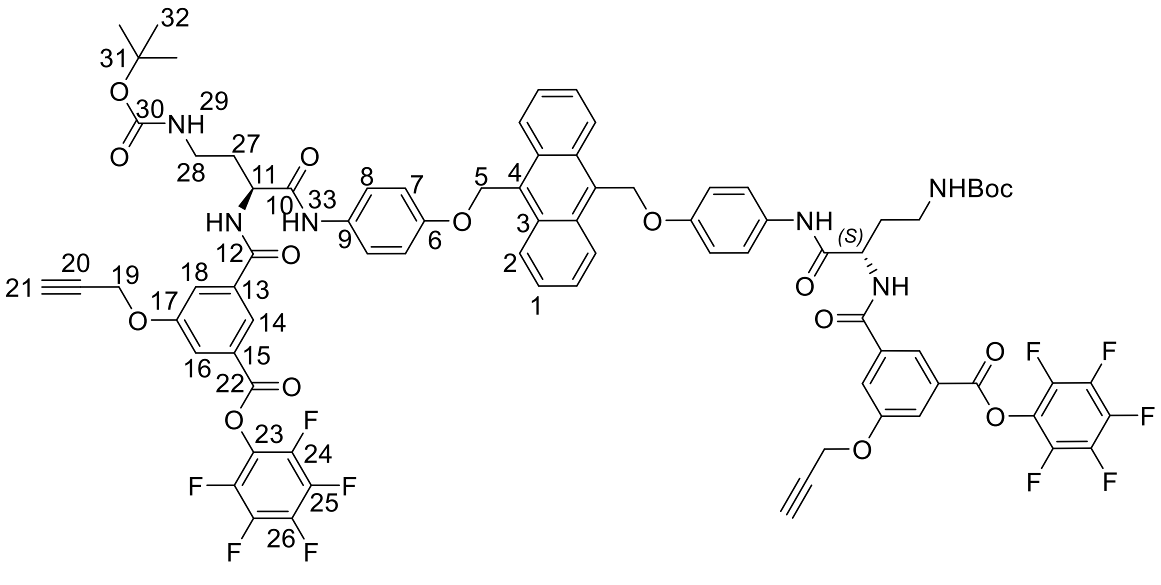

To a solution of bis(pentafluorophenyl)-5-(propargyloxy)isophthalate **S7** (336 mg, 0.61 mmol, 5.0 eqv) and compound **S17** (100 mg, 0.12 mmol, 1.0 eqv) in MeCN (10 ml) was added DIPEA (180 µL, 1.2 mmol, 10.0 eqv). The reaction was stirred at room temperature for 4 hours before the solvent was removed *in vacuo*. The residue

was then purified by normal (Hexane: EtOAc = 22 : 78) and reverse phase (Water : MeCN = 0 : 100) column chromatography to give **S18** (85 mg, 55 μmol, 45 %) as a yellow solid.

^{1}H NMR (500 MHz, $CDCl_3$) δ 9.67 (s, 2H, N33H), 8.65 (s, 2H, C16H), 8.50 – 8.44 (m, 4H, C2H), 8.10 (s, 2H, C14H), 8.04 – 8.01 (s, 2H, C18H), 7.63 – 7.55 (m, 8H, C1H, C8H), 7.20 (d, *J* = 8.6 Hz, 4H, C7H), 4.87 (d, *J* = 2.2 Hz, 4H, C19H), 4.81 (s, 4H, C5H), 3.57 (s, 2H, C11H)3.43 – 3.21 (m, 4H, C28H), 2.61 (d, *J* = 2.6 Hz, 2H, C21H), 1.99 – 1.84 (m, 4H, C27H), 1.44 (s, 18H, C32H). **^{13}C NMR** (126 MHz, $CDCl_3$) δ 163.9 (C12), 161.7 (C22), 158.1 (C17), 156.7 (C10), 146.7 (C6), 142.4 (C25), 140.4 (C23), 139.1 (C26), 137.1 (C24), 136.0 (C9), 132.0 (C13), 130.3 (C4), 128.9 (C15), 126.5 (C1), 125.3 (C16), 124.8 (C2), 122.6 (C14), 122.0 (C18), 121.9 (C7), 120.3 (C8), 79.8 (C31), 77.3 (C21), 77.0 (C20), 61.4 (C11), 56.6 (C19), 44.9 (C5), 37.4 (C28), 34.6 (C27), 28.5 (C32). **HRMS** for $C_{80}H_{66}F_{10}N_6O_{16}$ $[M+H]^+$ Calculated m/z = 1557.4454 Found m/z = 1557.4464.

TCNE adduct S19

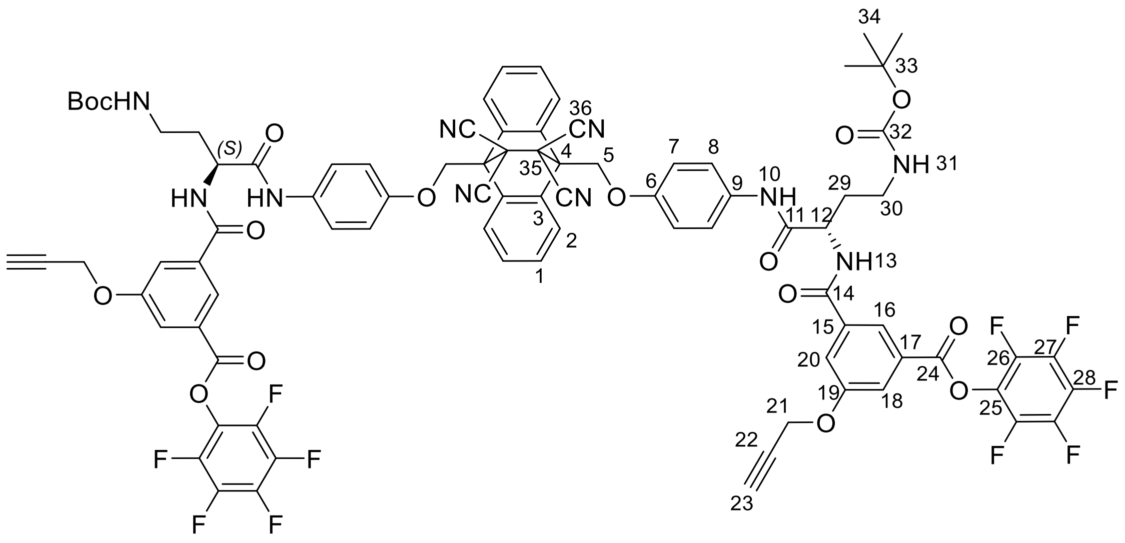

To a solution of **S18** (60 mg, 39 μmol, 1.0 eqv) in $CHCl_3$ (2 ml) was added tetracyanoethylene **27** (7.4 mg, 58 μmol, 1.5 eqv). Upon addition of the dienophile, the colour of the reaction changed from bright yellow to dark green,[5] suggesting a charge transfer complex forming between tetracyanoethylene and the electron-rich aromatics.[4] The reaction mixture was stirred at rt for 30 mins before the solvent was removed *in vacuo*. The residue was purified by column chromatography (Hexane : EtOAc = 20 :80) to give adduct **S19** (62 mg, 37 μmol, 95 %) as a yellow solid.

^{1}H NMR (500 MHz, $CDCl_3$) δ 10.15 (s, 2H, N10H), 8.34 (s, 2H, C18H), 7.96 (s, 2H, C16H), 7.90 (s, 2H, C20H), 7.78 (d, *J* = 9.0 Hz, 4H, C8H), 7.75 (d, *J* = 6.7Hz, 2H, N13H), 7.70 – 7.64 (m, 4H, C2H), 7.55 – 7.49 (m, 4H, C1H), 7.21 (d, *J* = 8.9 Hz, 4H, C7H), 5.40 (s, 4H, C5H), 5.21 – 5.13 (m, 2H, N31H), 4.89 – 4.81 (m, 6H, C21,12H), 3.75 – 3.65 (m, 2H, C30H), 3.22 – 3.14 (m, 2H, C30H), 2.62 (t, *J* = 2.3 Hz, 2H, C23H), 2.26 – 2.18 (m, 2H, C29H), 2.07 – 1.98 (m, 2H, C29H), 1.54 (s, 18H, C34H). **^{13}C NMR** (126 MHz, $CDCl_3$) δ 169.1 (C11),

165.1 (C14), 161.7 (C24), 158.1 (C19), 154.0 (C32), 152.1 (C6), 142.3 (C27), 140.3 (C25), 139.0 (C28), 137.9 (C3), 137.0 (C26), 136.2 (C15), 134.8 (C17), 133.1 (C9), 129.7 (C1), 128.7, 125.3 (C2), 122.0 (C16), 121.9 (C8), 120.3 (C20), 120.0 (C18), 115.1 (C7), 110.1 (C36), 80.8 (C33), 78.4 (C22), 76.9 (C23), 65.5 (C5), 60.4 (C4), 56.4 (C12), 51.5 (C21), 50.8 (C35), 37.1 (C30), 35.0 (C29), 28.4 (C34). **HRMS** for $C_{86}H_{66}F_{10}N_{10}O_{16}$ $[M+H]^+$ Calculated m/z = 1685.4577 Found m/z = 1685.4578

Bis-ammonium cation S20

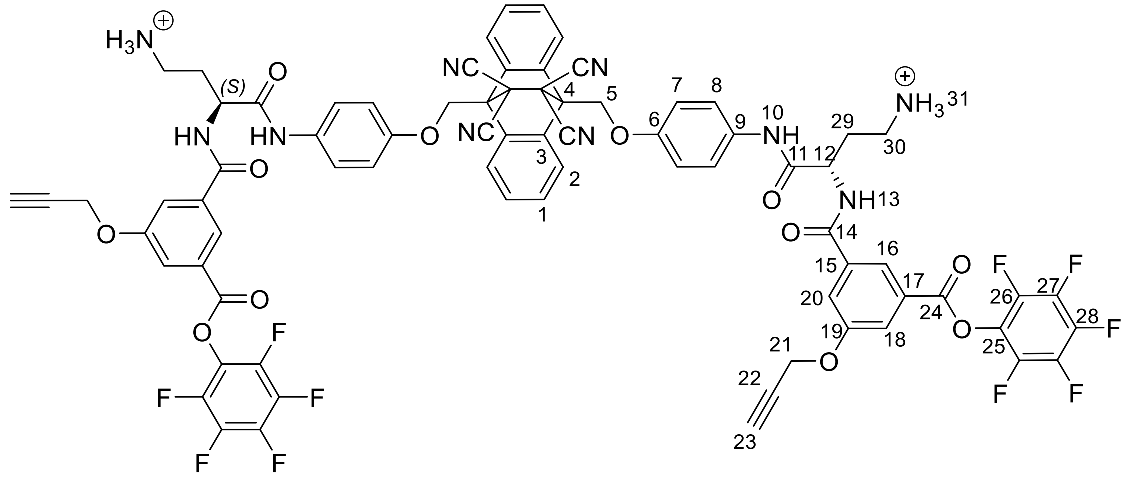

To a solution of **S19** (62 mg, 37 µmol) in cold DCM (3 ml) was added TFA (1 ml). The reaction was stirred at 0°C for 30 mins before the solvent was removed *in vacuo* to give bis-ammonium **S20** as the TFA salt, a colourless solid. The formation of the intermediate was confirmed by ^{1}H NMR and LC-MS.

^{1}H NMR (500 MHz, DMSO) δ 10.17 (s, 2H, , N10H), 9.19 (d, *J* = 7.2 Hz, 2H, N13H), 8.40 (s, 2H, C18H), 8.00 (s, 2H, C16H), 7.94 (s, 2H, C20H), 7.80 – 7.76 (m, 4H, C1H), 7.76 – 7.72 (m, 6H, N31H), 7.69 (d, *J* = 8.9 Hz, 4H, C8H), 7.59 – 7.53 (m, 4H, C2H), 7.35 (d, *J* = 8.7 Hz, 4H, C7H), 5.49 (s, 4H, C5H), 5.06 (d, *J* = 2.3 Hz, 4H, C21H), 4.79 – 4.72 (m, 2H, C12H), 3.70 (t, *J* = 2.4 Hz, 2H, C23H), 3.01 – 2.89 (m, 4H, C30H), 2.30 – 2.16 (m, 2H, C29H), 2.16 – 2.09 (m, 2H, C29H). **LCMS** for $C_{76}H_{52}F_{10}N_{10}O_{12}^{2+}$ $[M-H]^+$ Calculated m/z = 1486.3 Found m/z = 1486.3

Bis-ammonium cation S21

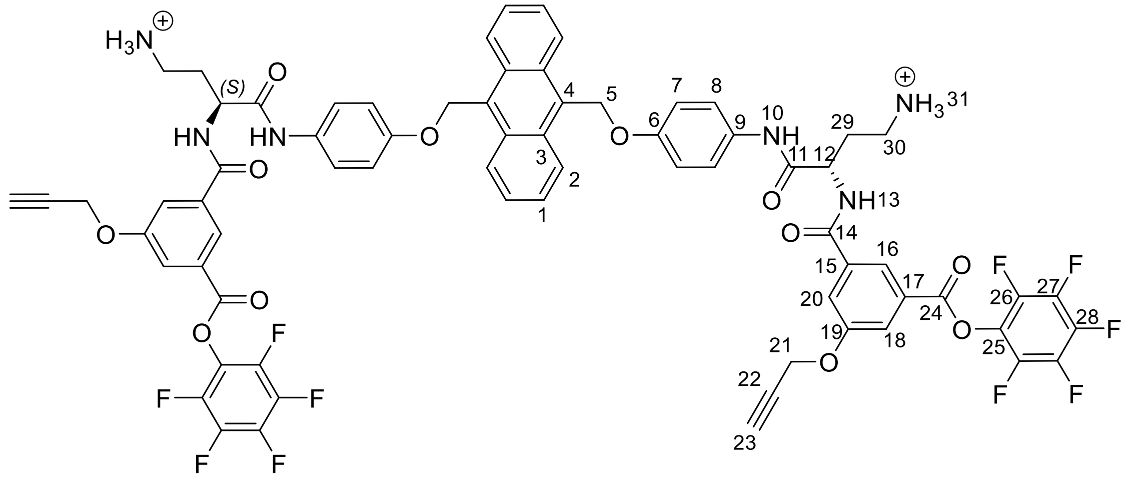

Bis-ammonium **20** TFA salt was dissolved in MeCN (10 ml). The reaction mixture was heated at 60 °C for 3 hours, and the colour of the solution changed from colourless to light yellow. The solvent was then removed *in vacuo* to give **21** TFA salt as a light yellow solid. The formation of **21** was checked by ^{1}H NMR and LC-MS.

^{1}H NMR (500 MHz, DMSO) δ10.06 (s, 2H, N10H), 9.04 (d, *J* = 7.6 Hz, 2H, N13H), 8.45 – 8.38 (m, 4H, C1H), 8.18 (s, 2H, C18H), 7.78 (s, 2H, C16H), 7.75 – 7.68 (m, 8H, N31H, C20H), 7.67 – 7.62 (m, 4H, C2H), 7.60 (d, *J* = 9.0 Hz, 4H, C8H), 7.18 (d, *J* = 8.8 Hz, 4H, C7H), 6.06 (s, 4H, C5H), 4.96 (d, *J* = 2.3 Hz, 4H, C21H), 4.75 – 4.68 (m, 2H, C12H), 3.65 (t, *J* = 2.3 Hz, 2H, C23H), 2.97 – 2.89 (m, 4H, C30H), 2.25 – 2.15 (m, 2H, C29H), 2.15 – 2.04 (m, 2H, C29H). **LCMS** for $C_{70}H_{52}F_{10}N_{16}O_{12}^{2+}$ $[M-H]^{+}$ Calculated m/z = 1358.3 Found m/z = 1358.3

Receptor 12

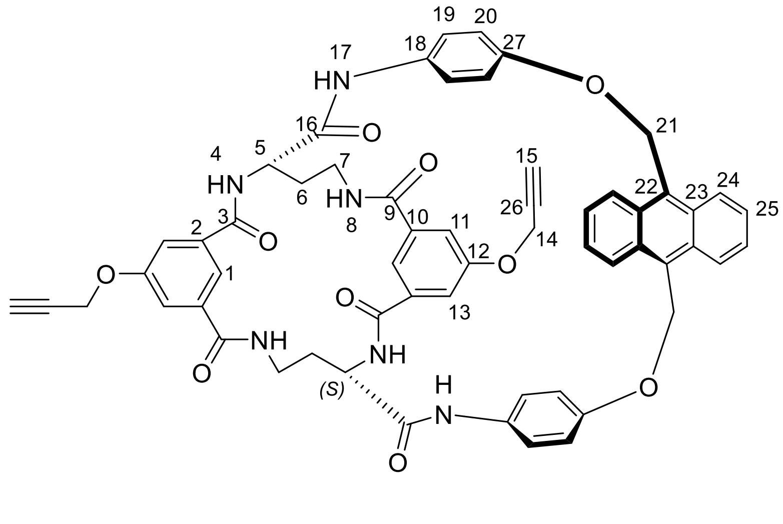

Bis-ammonium **S21** TFA salt (50 mg, 37 µmol, 1.0 eqv) was dissolved in MeCN (10 ml) and placed in a syringe. Separately, a solution of, DIPEA (128 µL, 736 µmol, 20 eqv) and TBACl (51 mg, 184 µmol, 5.0 eqv) in MeCN (65 ml) was prepared in a round bottomed flask. The solution in the syringe was added slowly to the round bottomed flask at room temperature over 24 hours. The solution was then stirred at room temperature for 4

hours before the solvent was removed *in vacuo*. The residue was then purified by Prep-LCMS on a C18 column with a gradient of H_2O (0.1% FA) : MeCN (0.1% FA) = 11 : 89 to give receptor **12** (23.7 mg, 24 µmol, 65 %) as a pale yellow solid.

^{1}H NMR (500 MHz, DMSO) δ 9.76 (s, 2H, N17H), 8.83 (d, *J* = 8.5 Hz, 2H, N4H), 8.77 – 8.71 (m, 4H, C24H), 8.46 – 8.30 (m, 4H, C1H, N8H), 7.66 – 7.60 (m, 4H, C25H), 7.44 (s, 2H, C11H), 7.35 (s, 2H, C13H), 7.23 (d, *J* = 8.9 Hz, 4H, C19H), 6.66 (d, *J* = 8.4Hz, 4H, C20H), 6.36 – 6.22 (m, 4H, C21H), 4.84 – 4.78 (m, 2H, C5H), 4.77 (d, *J* = 2.0 Hz, 4H, C14H), 3.53 (t, *J* = 2.2 Hz, 2H, C15H), 2.23 – 2.04 (m, 4H, C6H). **^{13}C NMR** (126 MHz, DMSO) δ 168.8 (C16), 164.7 (C3), 164.6 (C9), 157.0 (C12), 153.6 (C27), 135.3 (C10), 134.8 (C2), 132.2 (C18), 130.2 (C22), 129.7 (C23), 126.0 (C25), 125.2 (C24), 121.4 (C19), 117.4 (C1), 116.9 (C11), 116.8 (C13), 116.4 (C20), 78.8 (C15), 78.6 (C26), 64.1 (C21), 55.6 (C14), 53.8 (C5), 40.4, 36.7 (C7), 30.2 (C6). **HRMS** for $C_{58}H_{48}N_6O_{10}$ $[M+Na]^+$ Calculated m/z = 1011.3314 Found m/z = 1011.3341

Receptor 13

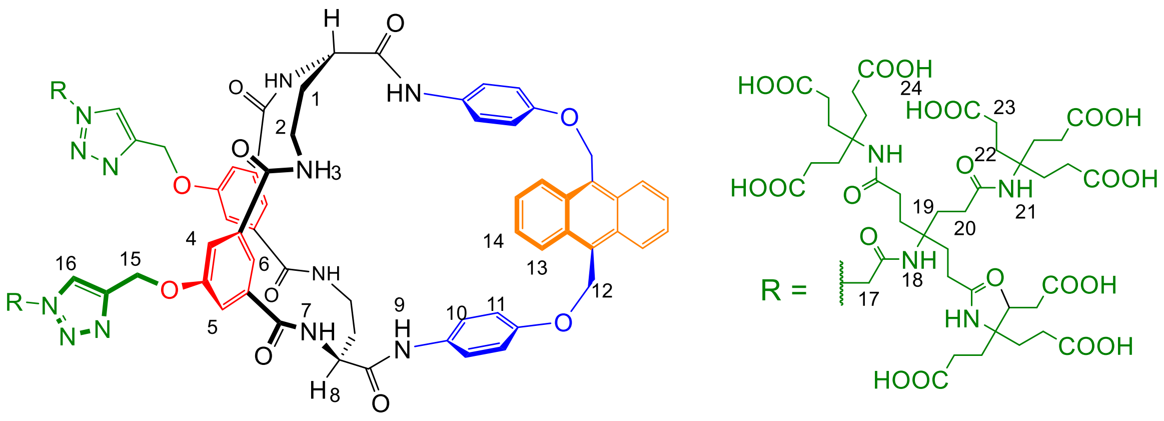

To a solution of bicycle **12** (21.0 mg, 21.1 µmol, 1.0 eqv) in de-gassed MeCN and DMF mixture (2 ml + 2 ml) was added azido nona-acid **S10**[3] (54.0 mg, 53.1 µmol, 2.5 eqv) and 2,6-lutidine (68.3 mg, 637 µmol, 30.0 eqv). The solution was stirred at room temperature for 15 minutes before tetrakis(acetonitrile)copper(I) hexafluorophosphate (15.8 mg, 42.5 µmol, 2.0 eqv) was added. The reaction mixture was stirred at 60 °C for overnight before the solvent was removed in vacuo. The residue was then purified by Prep-LCMS on a C18 column with a gradient of H_2O (0.1% FA) : MeCN (0.1% FA) = 32 : 68 to give receptor **13** (18.6 mg, 6.2 µmol, 29 %) as a brown solid.

^{1}H NMR (600 MHz, DMSO) δ 12.05 (s, 18H, C24H), 9.65 (s, 2H, N9H), 8.80 – 8.55 (m, 8H, N3,7H, C13H), 8.18 (s, 2H, C6H), 8.11 (s, 2H, C16H), 7.79 (s, 2H, N18H), 7.62 (s, 4H, C14H), 7.56 (s, 4H, C5H), 7.51 (s, 4H, C4H), 7.36 – 7.21 (m, 10H, N21H, C10H), 6.86 – 6.67 (m, 4H, C11H), 6.28 (s, 4H, C12H), 5.13 (s, 4H, C15H), 5.07 (s, 4H, C17H), 4.90 – 4.81 (m, 2H, C8H), 3.86 (s, 2H, C2H), 2.35 – 2.26 (m, 2H, C1H), 2.21 – 2.17 (m, 2H, C1H), 2.15 – 2.00 (m, 48H, C20,23H), 1.89 – 1.69 (m, 48H, C19,22H). **HRMS** for $C_{142}H_{174}N_{20}O_{54}$ $[M-2H]^{2-}$ Calculated m/z = 1511.0675 Found m/z = 1151.0639

^{13}C NMR could not be acquired due to aggregation of the receptor.

1.6 Deprotonation of carboxylic acids on receptors

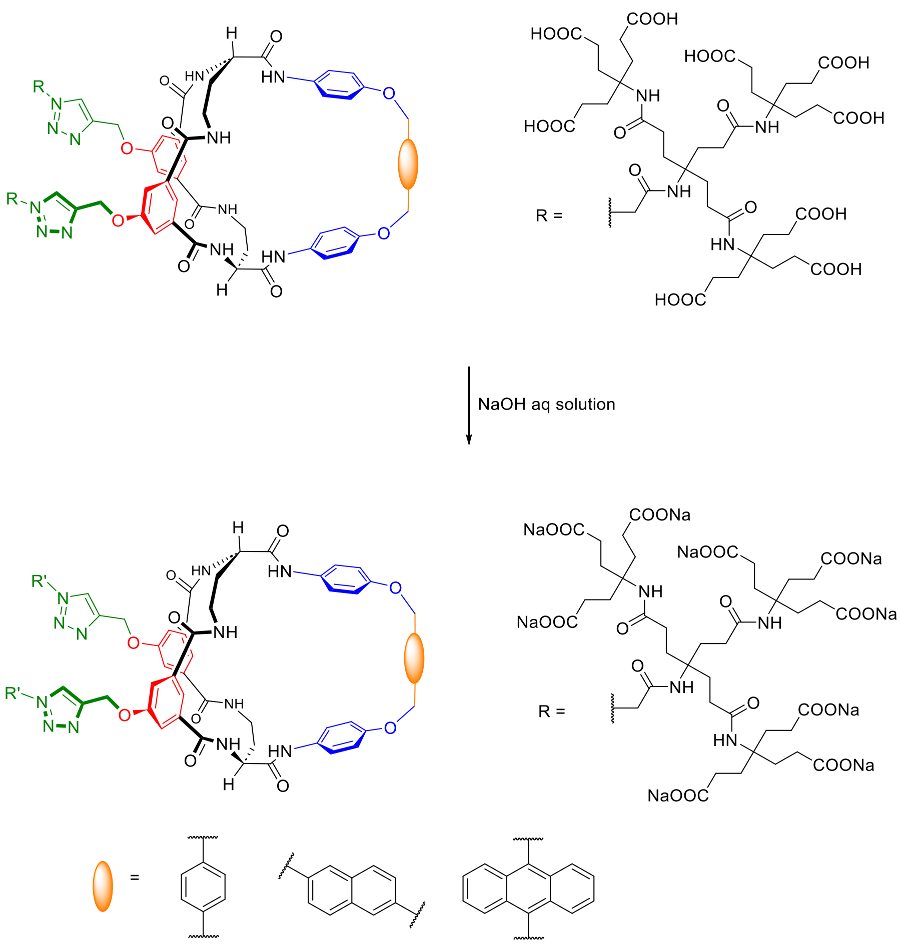

Scheme S6

To a suspension of bicyclic octadeca-acid in water (10 ml) was carefully added 10 mM NaOH aq solution to adjust the pH to 7.4. The resulting clear colourless solution was freeze-dried to yield the water soluble macrocycles (quant yield) in their sodium form as white solids. Samples for characterisation and further studies were prepared by dissolution in D_2O or 9:1 H_2O/D_2O. The process of freeze-drying and dissolution was found to have no effect on the pH.

1.7 Characterisation of receptors in their operating environments

Each receptor was characterised by NMR in the environment employed for binding studies, i.e. DMSO-*d*[6] for **7**, **8**, **10** and **12**, and 9:1 H_2O/D_2O at pH~7.4 for **9**, **11** and **13**.

Receptor 7

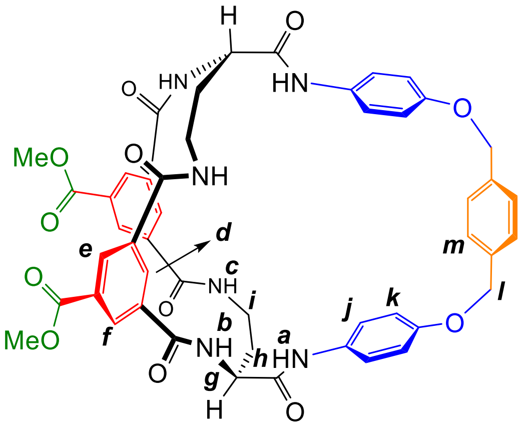

The assignment of the ^{1}H NMR spectrum in was made with the help of 2D COSY, HSQC and NOESY (see Supplementary Figures below).

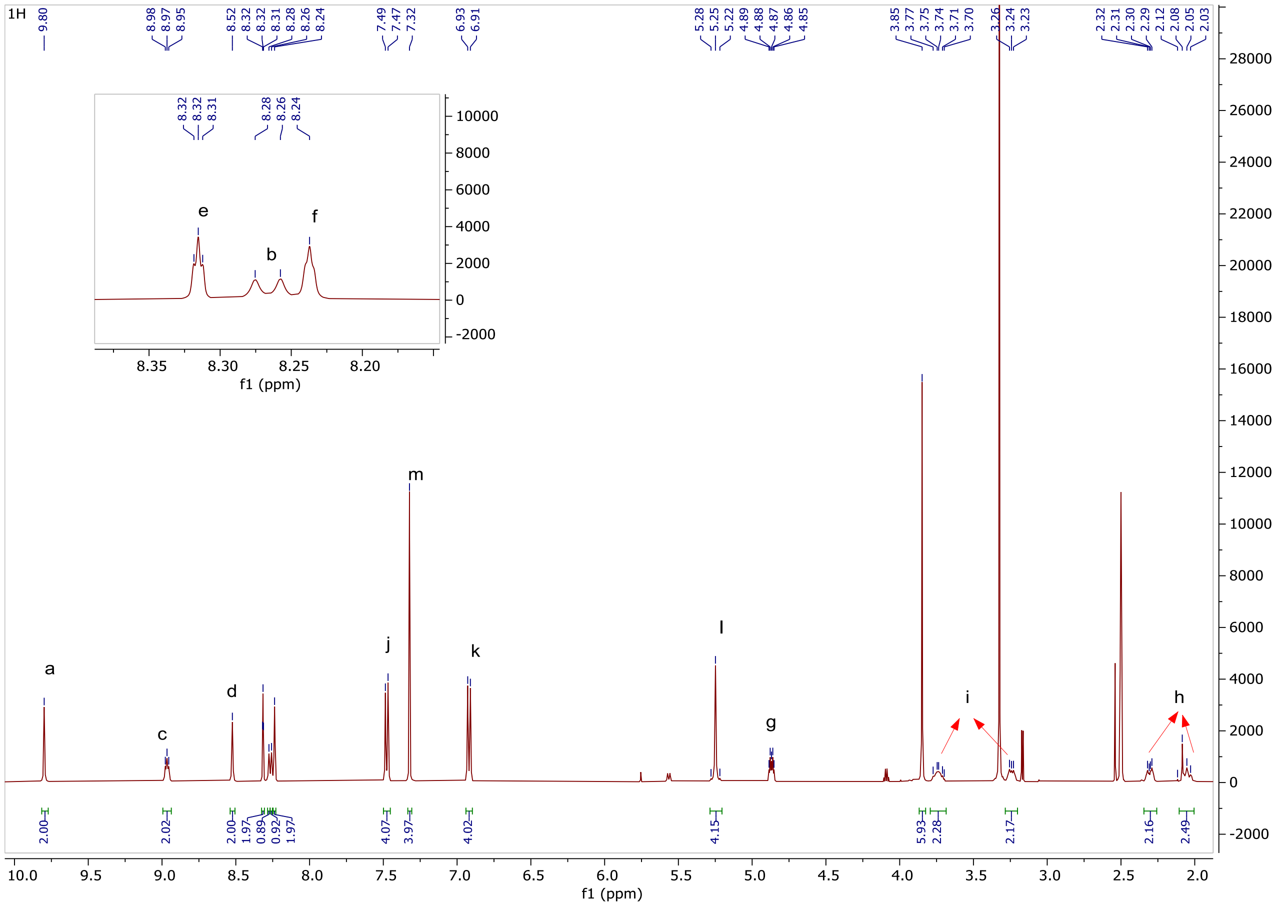

Figure S2. ^{1}H NMR spectrum (500 MHz) of receptor **7** in DMSO-*d*[6].

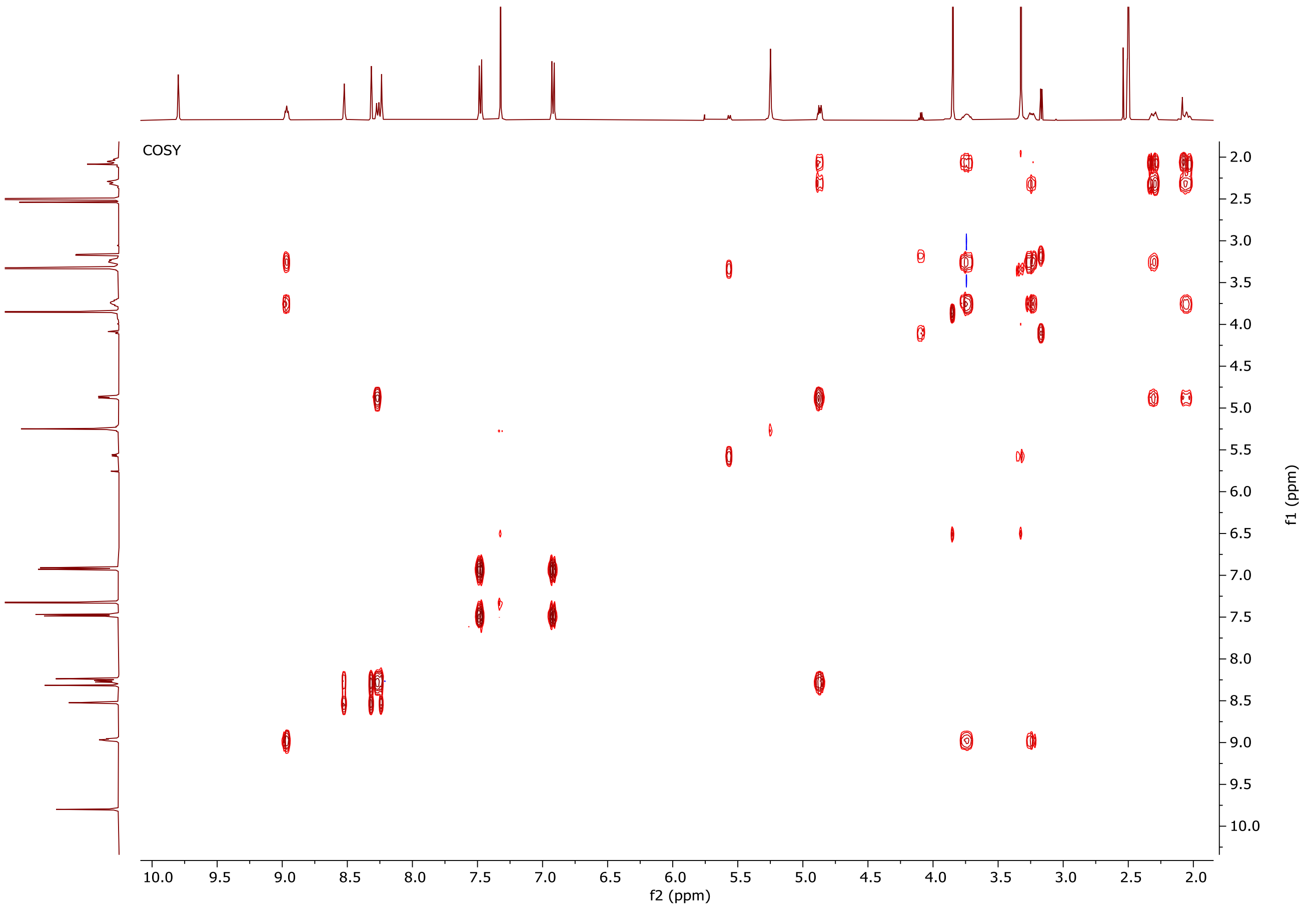

Figure S3. 2D COSY NMR spectrum (500 MHz) of receptor **7** in DMSO-d^6.

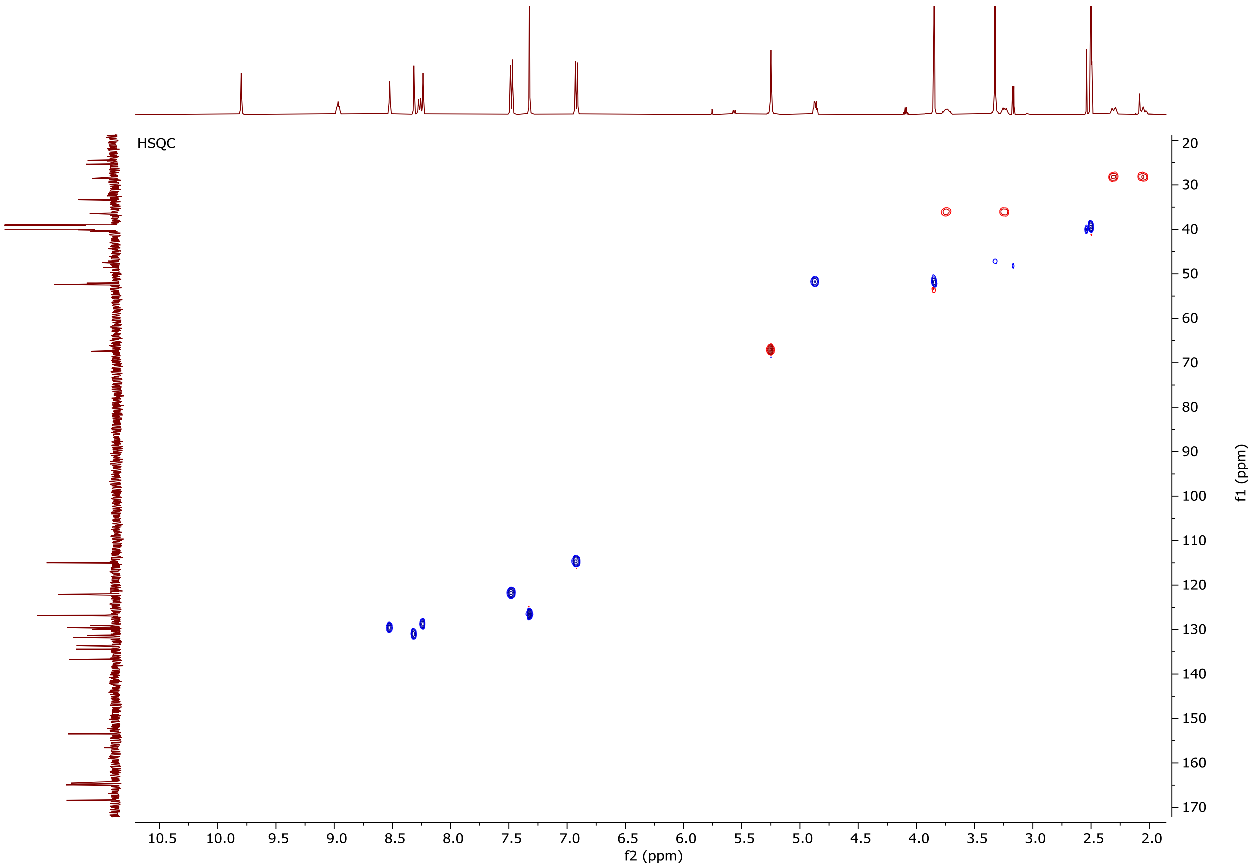

Figure S4. 2D HSQC NMR spectrum (500 MHz) of receptor **7** in DMSO-d^6.

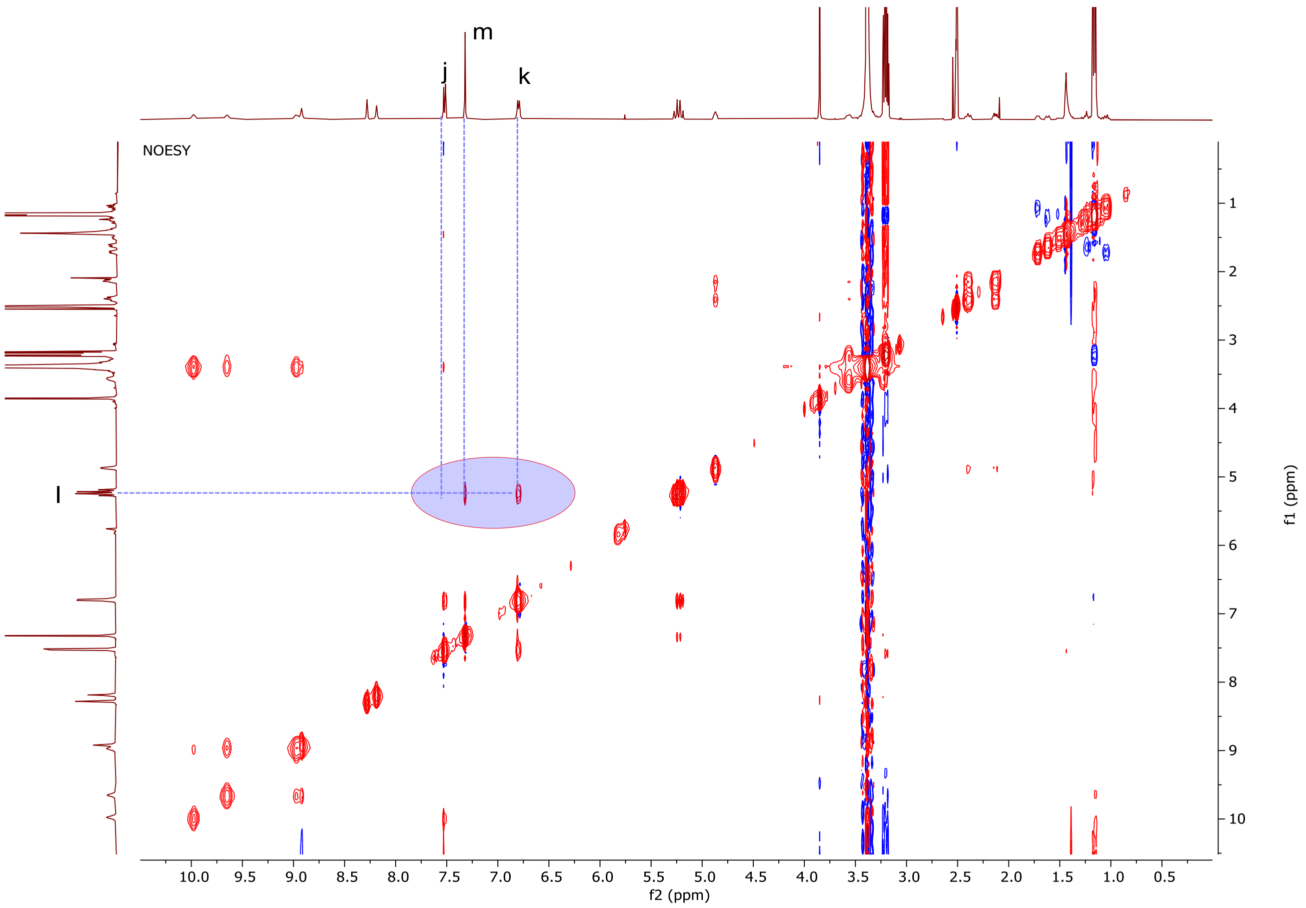

Figure S5. 2D NOESY NMR spectrum (600 MHz, 350 ms mixing time) of **7** (1.5 mM) and TBA Acetate (5 mM) complex in DMSO-d^6. Proton *k* is distinguished from *j* through cross-peak with *l*.

Receptor 8

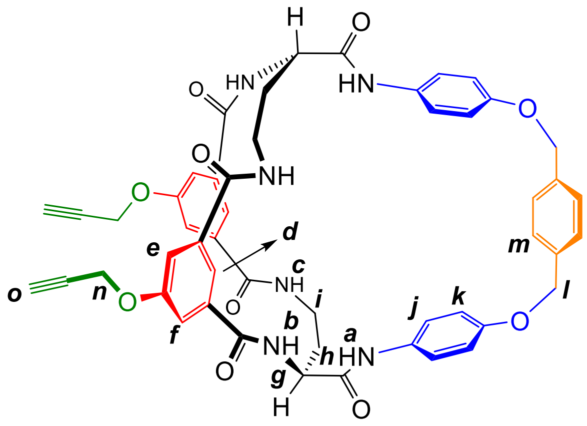

The assignment of the NMR spectrum in DMSO-d^6 was made with the help of 2D COSY and HSQC (see Supplementary Figures below).

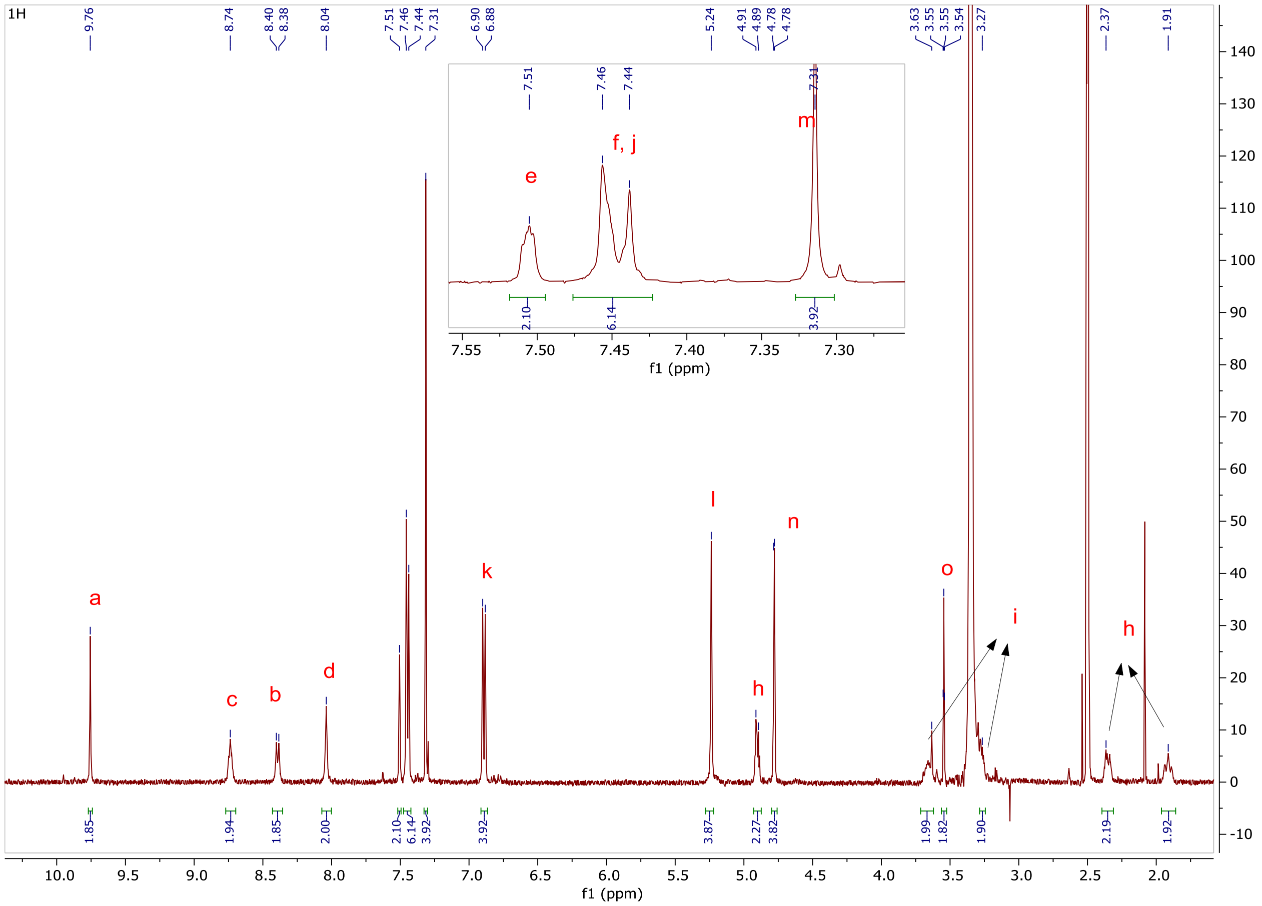

Figure S6. ^{1}H NMR spectrum (500 MHz) of receptor **8** in DMSO-d^6.

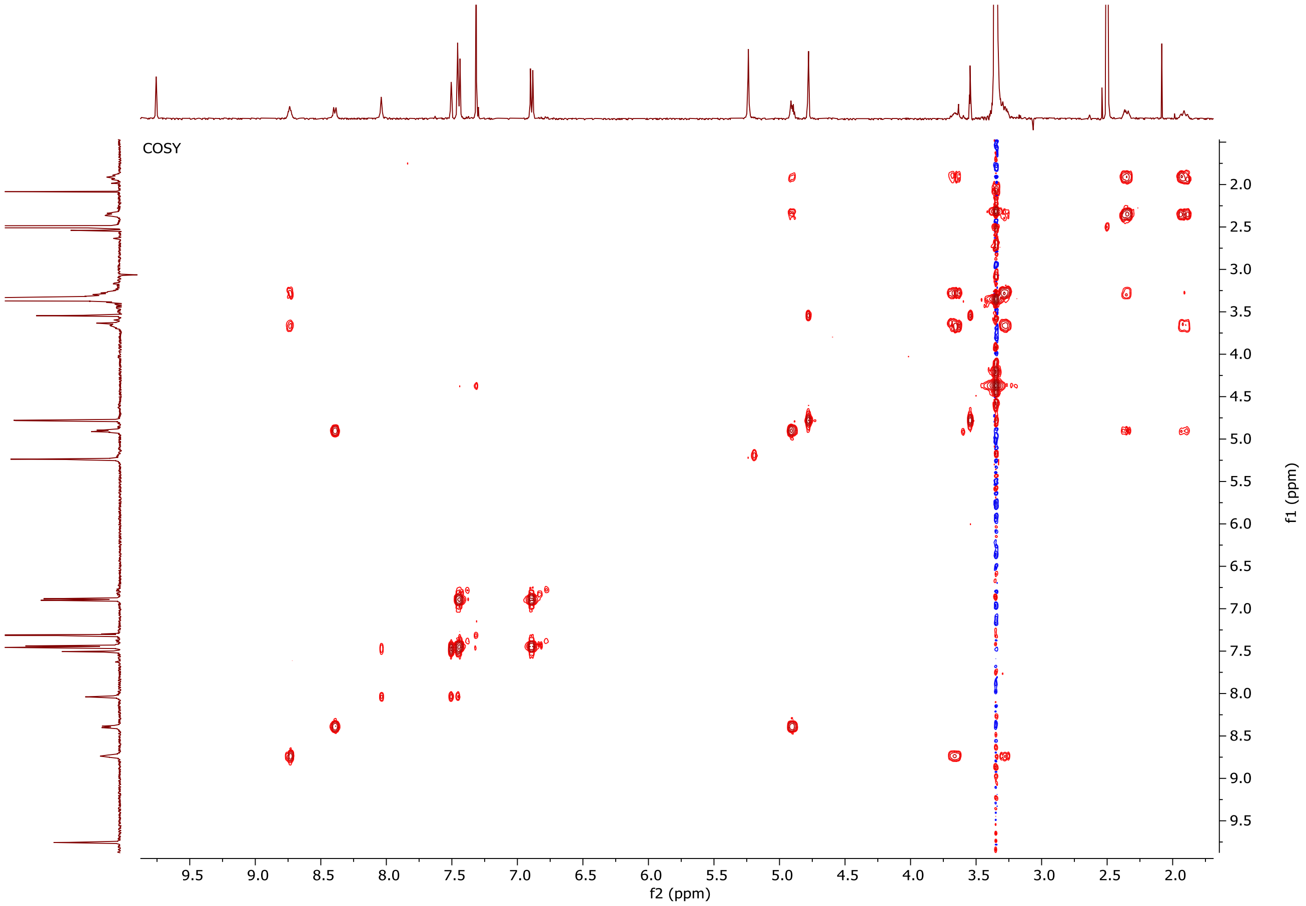

Figure S7. 2D COSY NMR spectrum (500 MHz) of receptor **8** in DMSO-*d*[6].

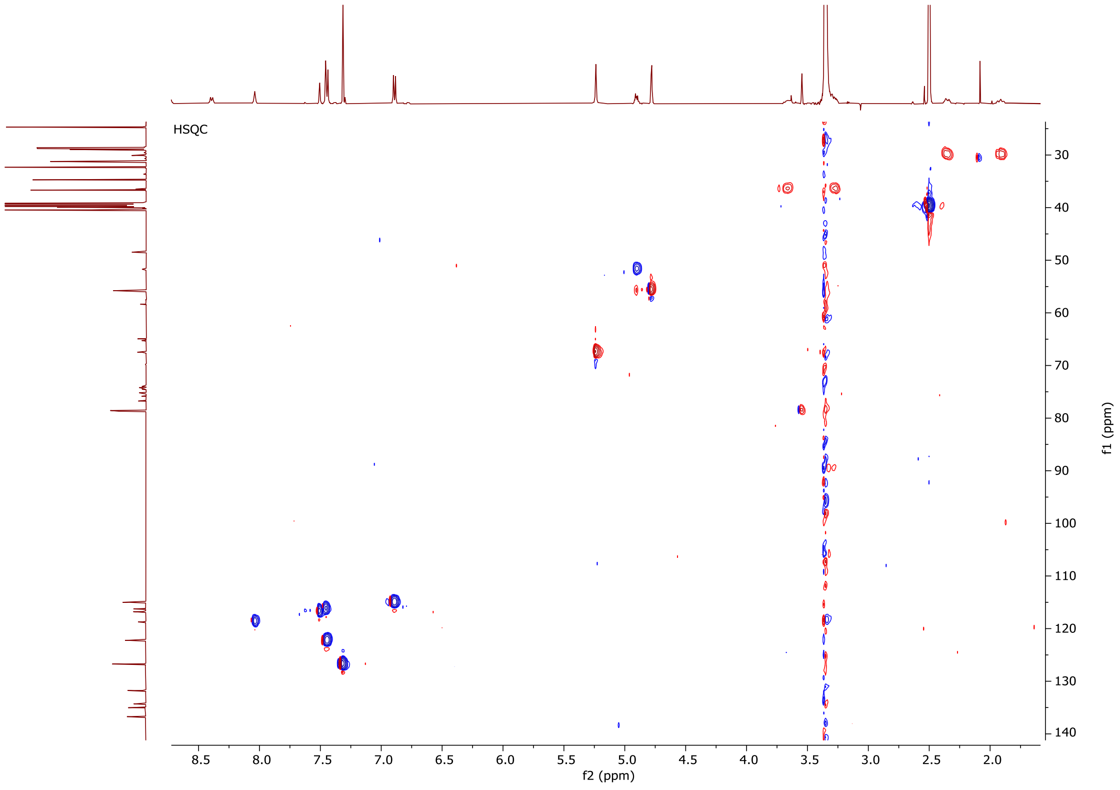

Figure S8. 2D HSQC NMR spectrum (500 MHz) of receptor **8** in DMSO-*d*[6]

Receptor 10

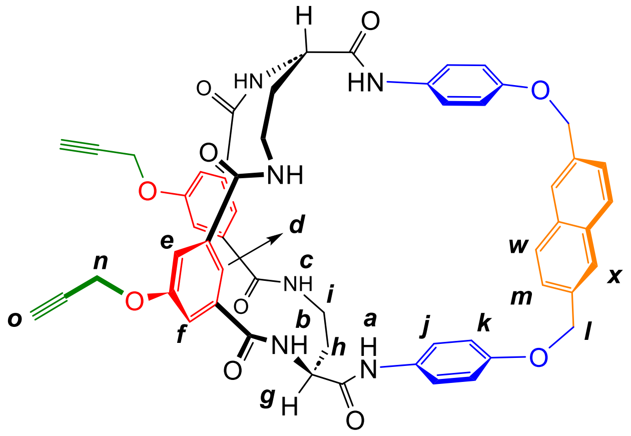

The assignment of the NMR spectrum in DMSO-d^6 was made with the help of 2D COSY and HSQC (see Supplementary Figures below).

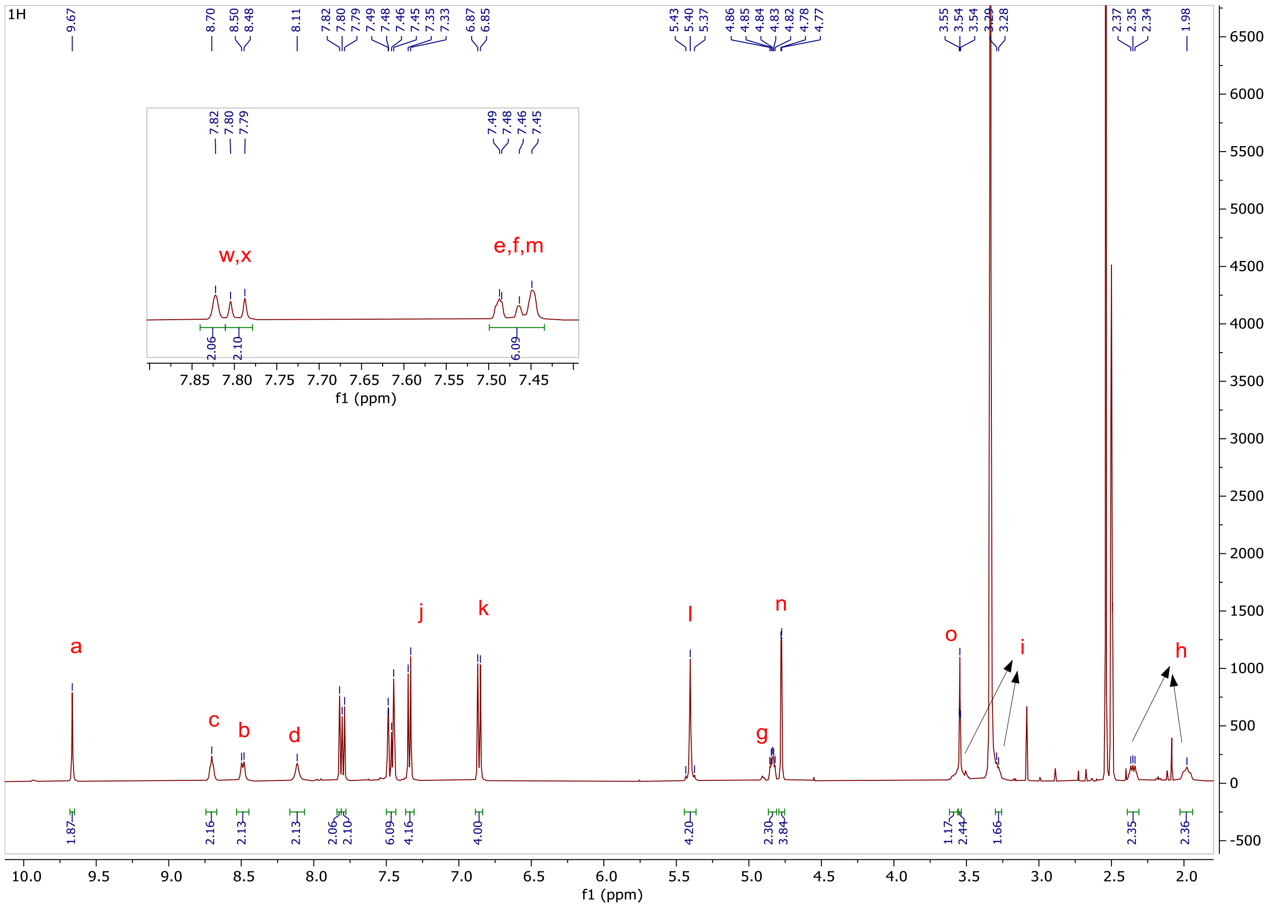

Figure S9. ^{1}H NMR spectrum (500 MHz) of receptor **10** in DMSO-d^6.

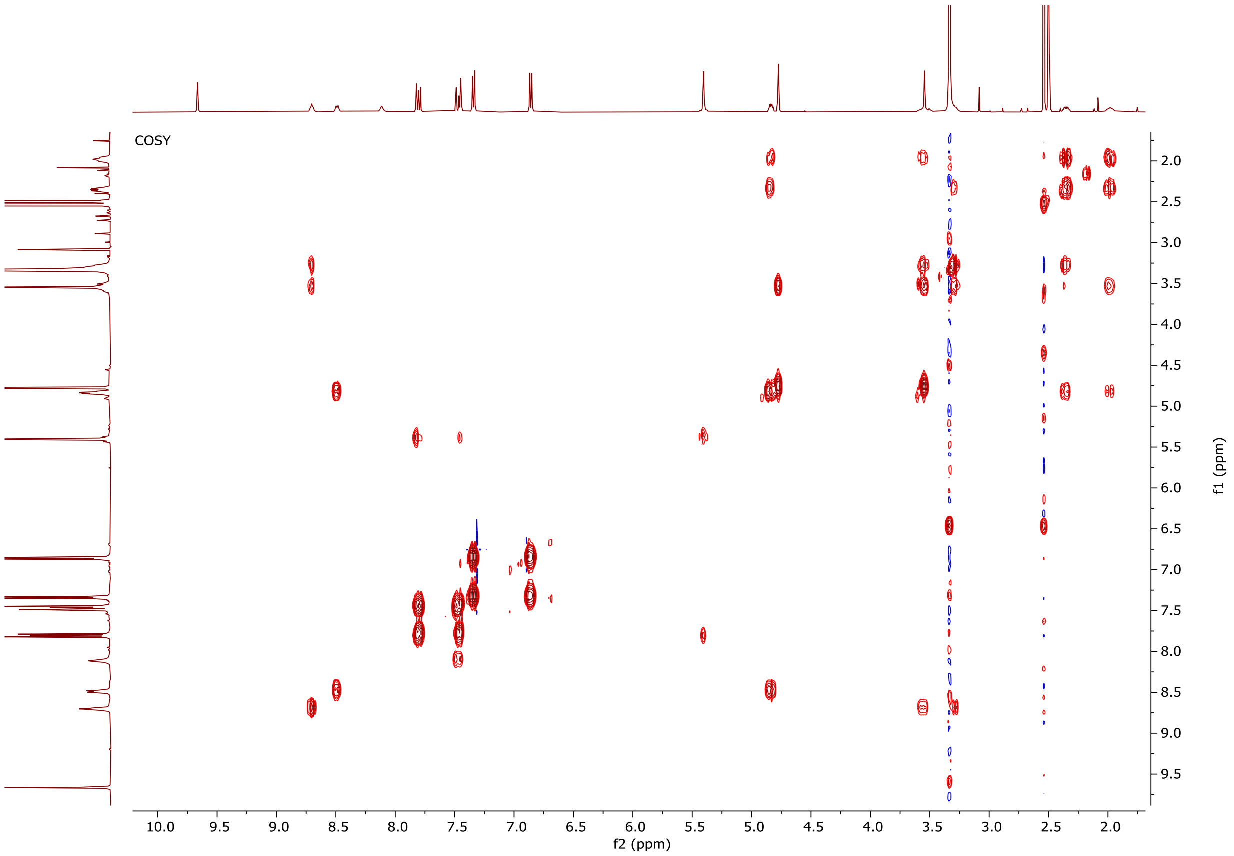

Figure S10. 2D COSY NMR spectrum (500 MHz) of receptor **10** in 9:1 H_2O/D_2O.

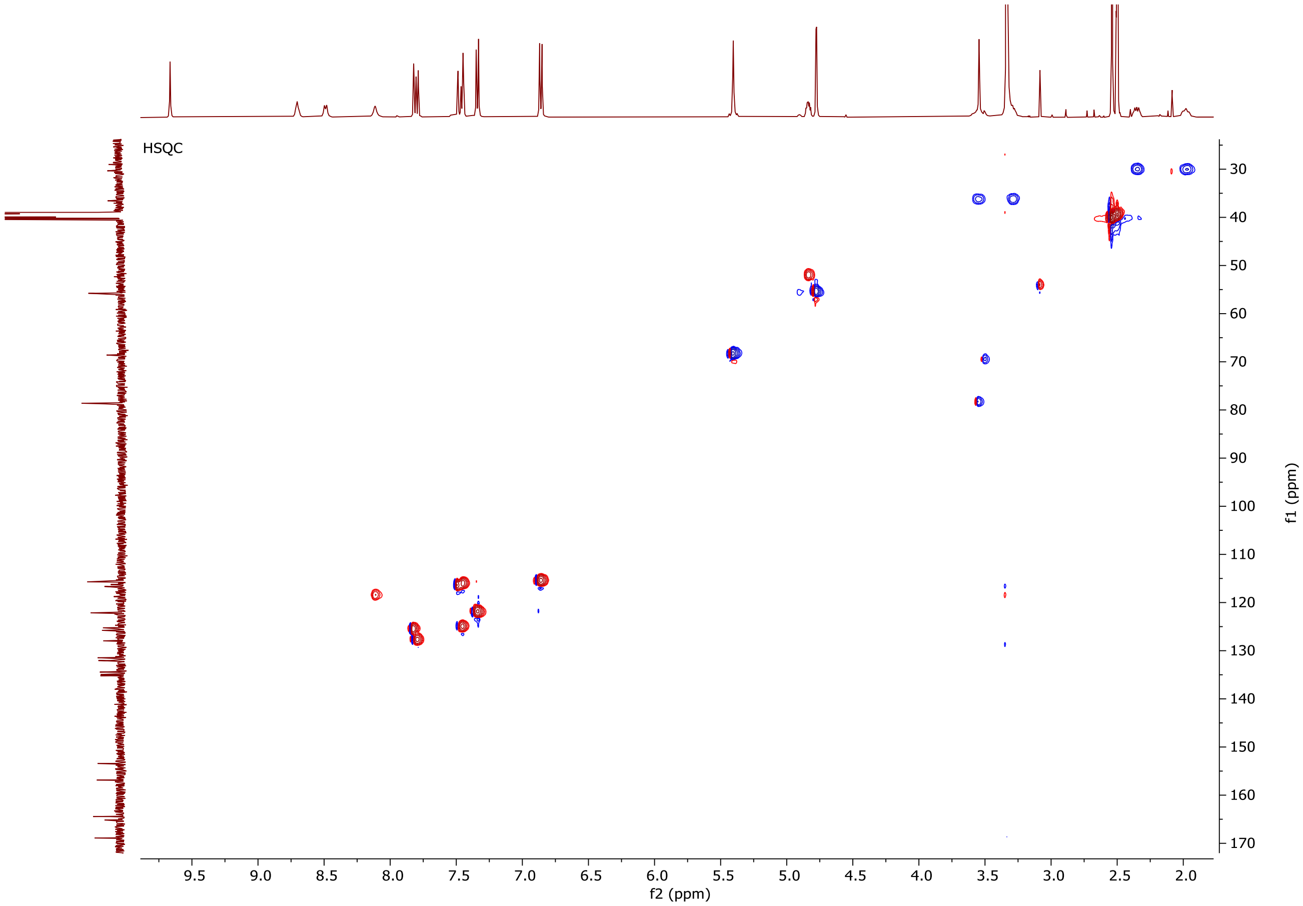

Figure S11. 2D HSQC NMR spectrum (500 MHz) of receptor **10** in DMSO-d^6

Receptor 12

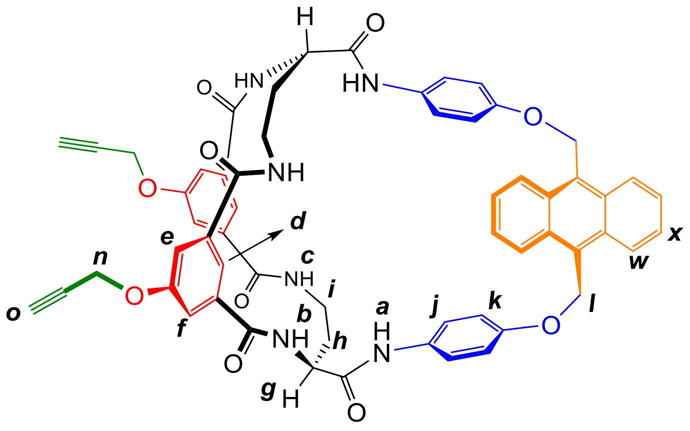

The assignment of the NMR spectrum in DMSO-d^6 was made with the help of 2D COSY and HSQC (see Supplementary Figures below).

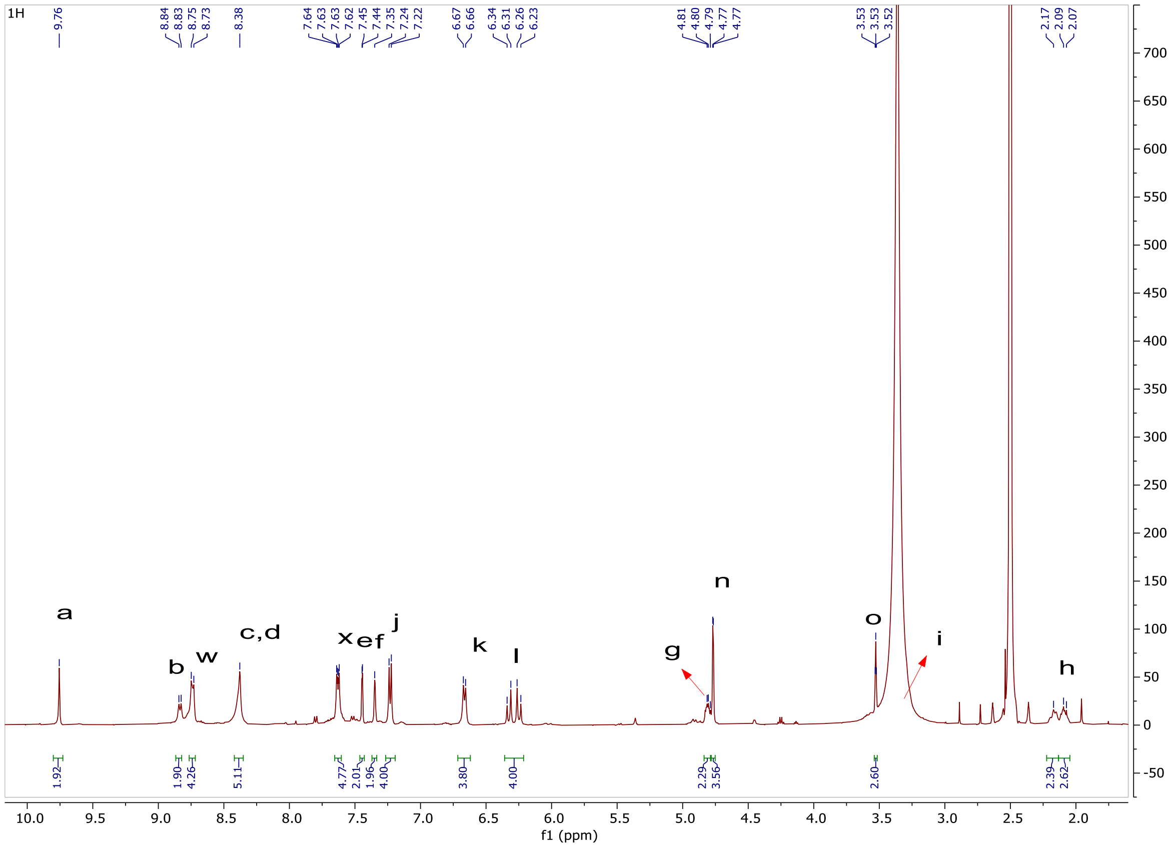

Figure S12. ^{1}H NMR spectrum (500 MHz) of receptor **12** in DMSO-d^6.

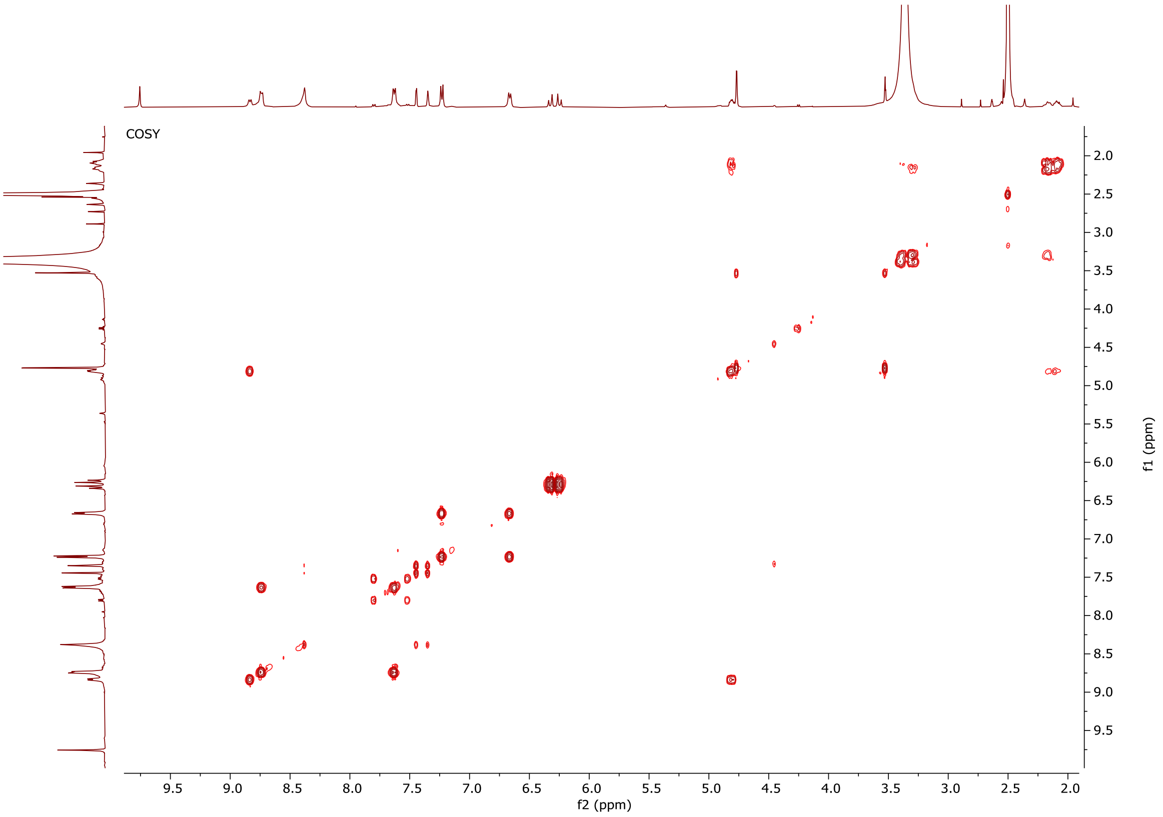

Figure S13. 2D COSY NMR spectrum (500 MHz) of receptor **12** in DMSO-d^6

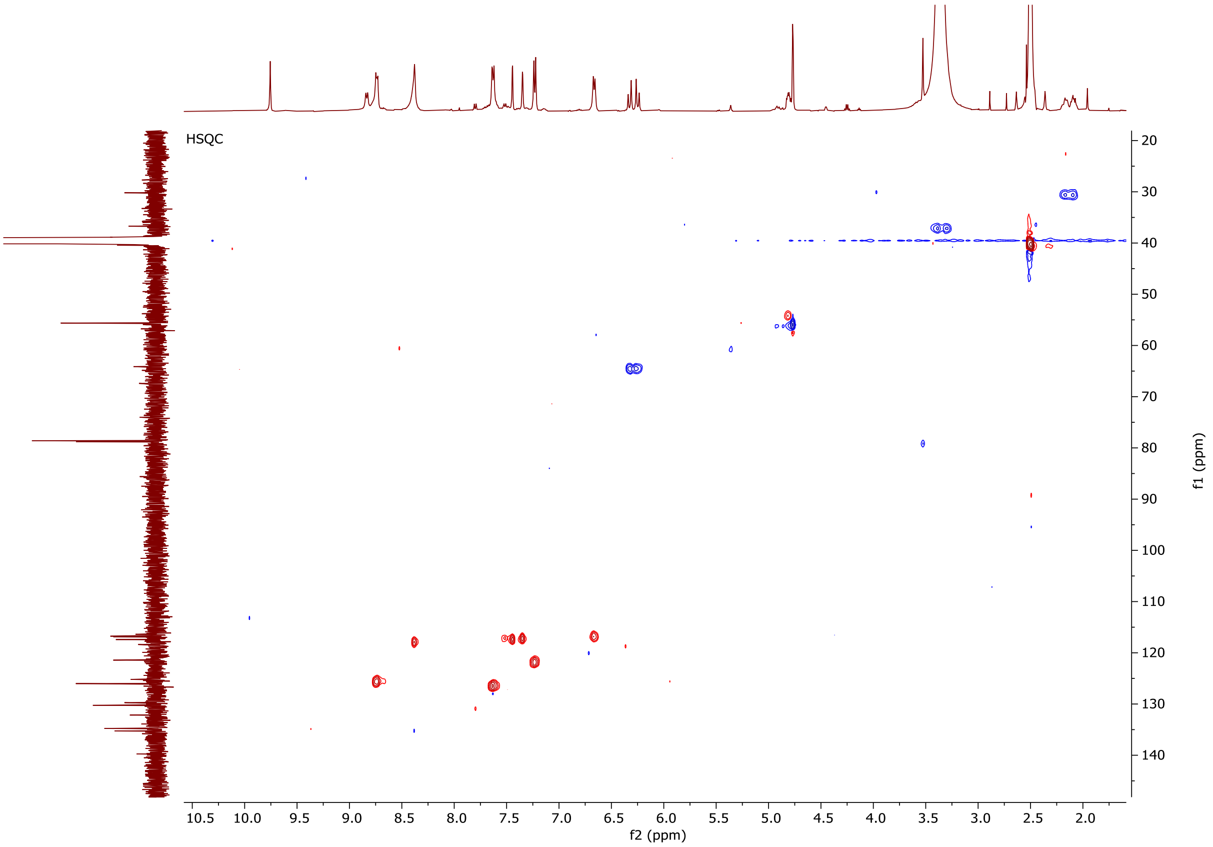

Figure S14. 2D COSY NMR spectrum (500 MHz) of receptor **12** in DMSO-d^6

Receptor 9

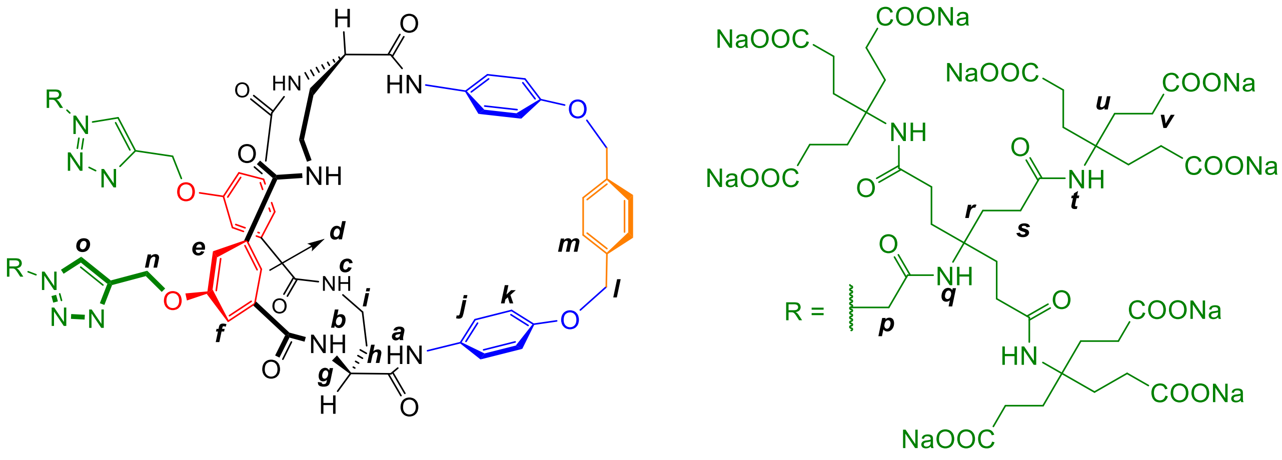

The assignment of the NMR spectrum in 9:1 H_2O/D_2O. was made with the help of 2D TOCSY and HSQC (see Supplementary Figures below). The side-chain carboxyls are shown as sodium salts but are expected to be only partially deprotonated.

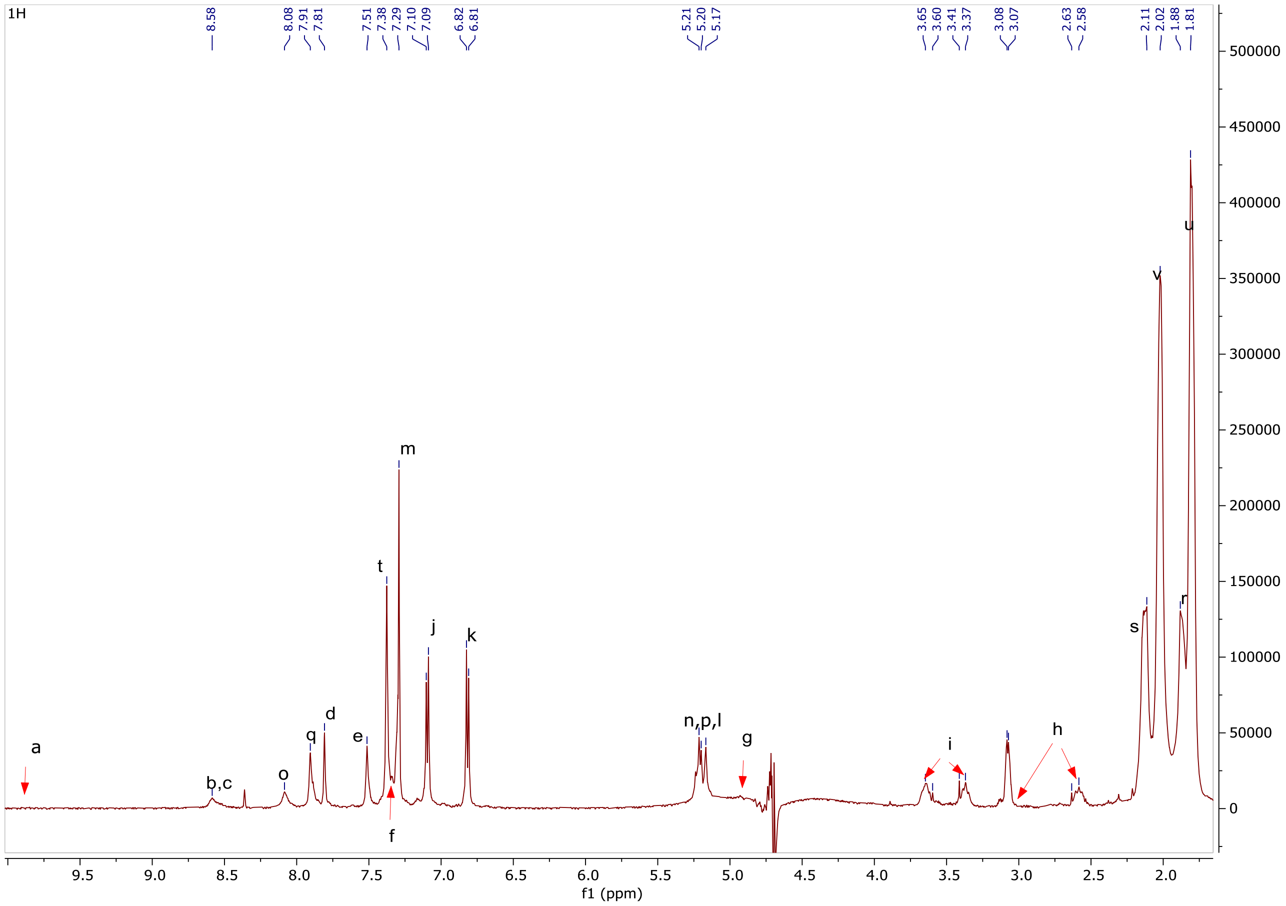

Figure S15. ^{1}H NMR spectrum (600 MHz) of receptor **9** in 9:1 H_2O/D_2O

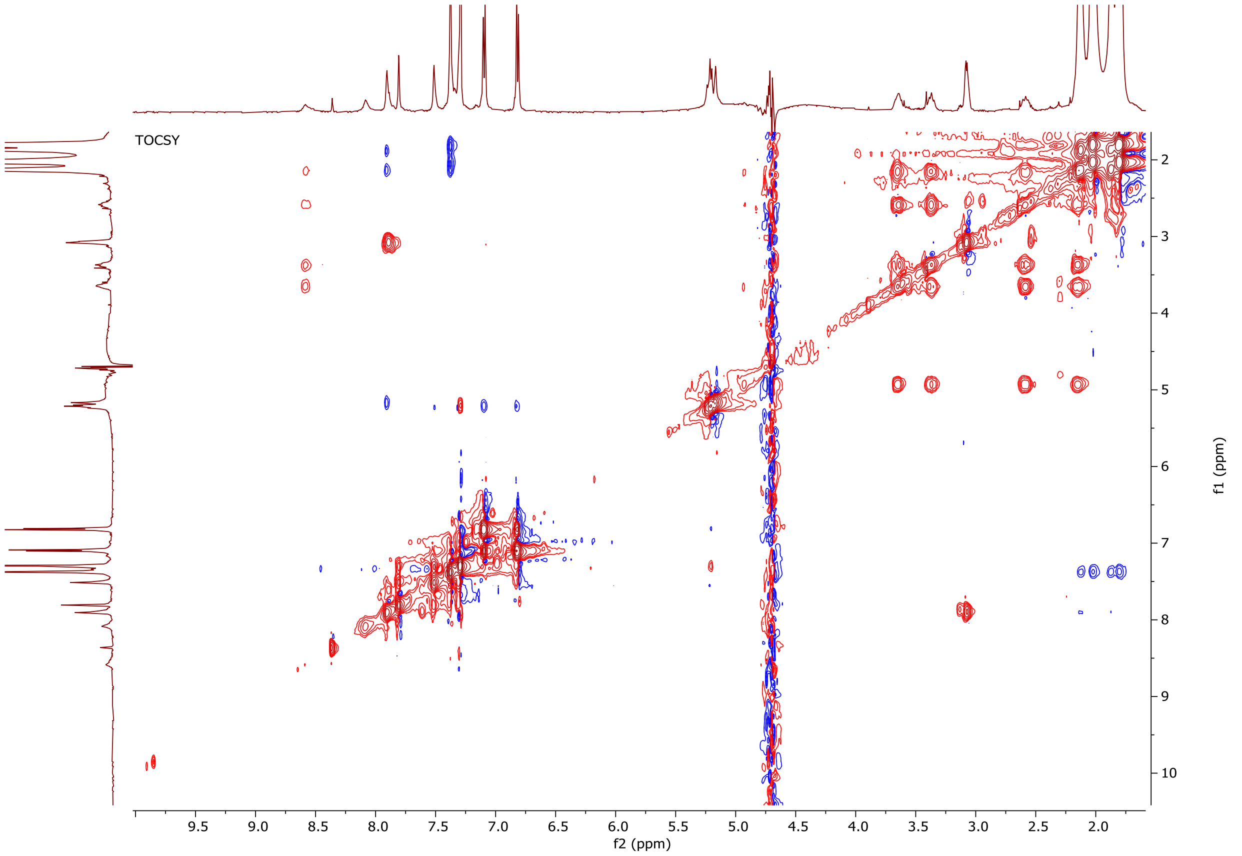

Figure S16. 2D TOCSY NMR spectrum (600 MHz) of receptor **9** in 9:1 H_2O/D_2O.

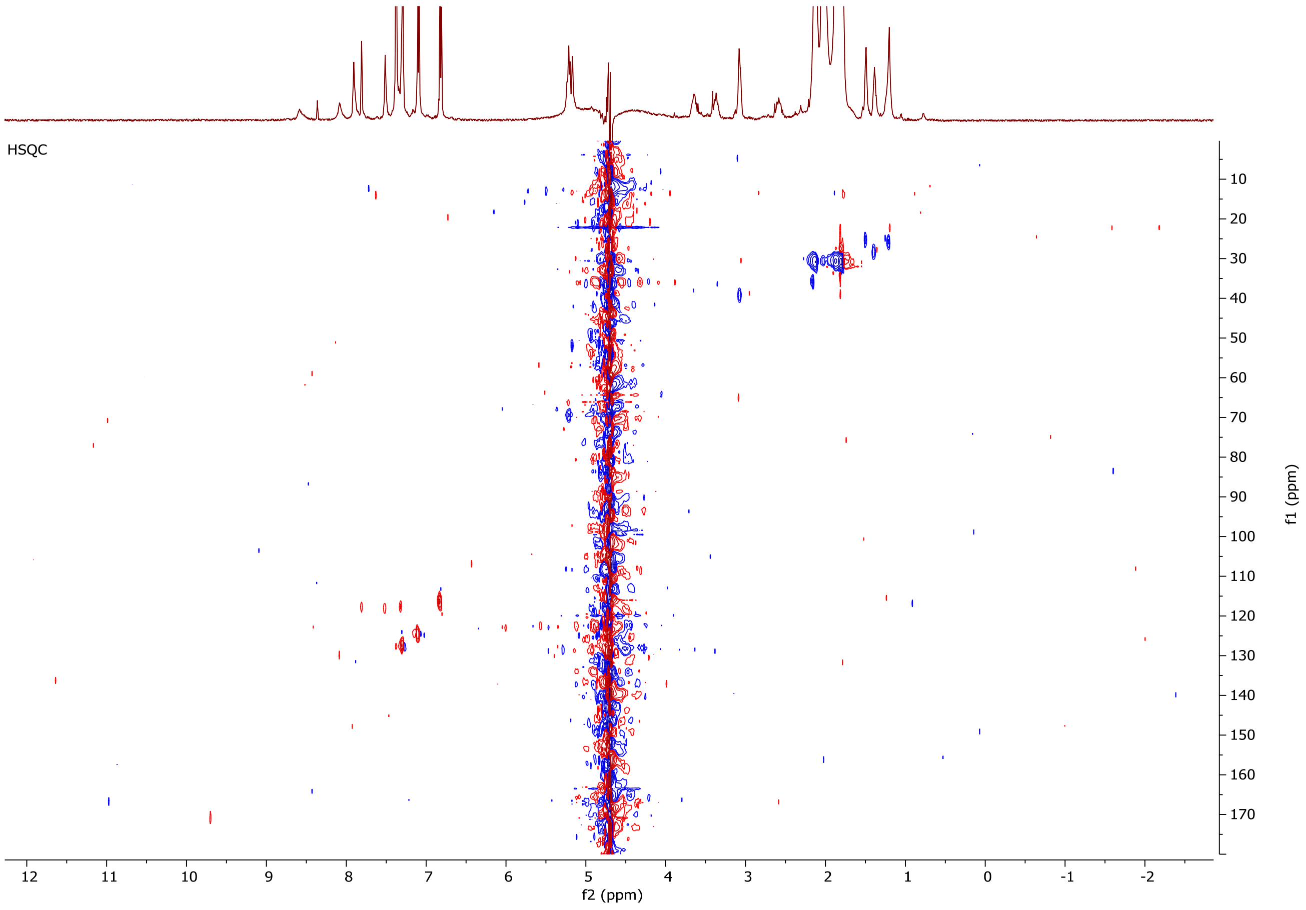

Figure S17. 2D HSQC NMR spectrum (600 MHz) of receptor **9** in 9:1 H_2O/D_2O.

Receptor 11

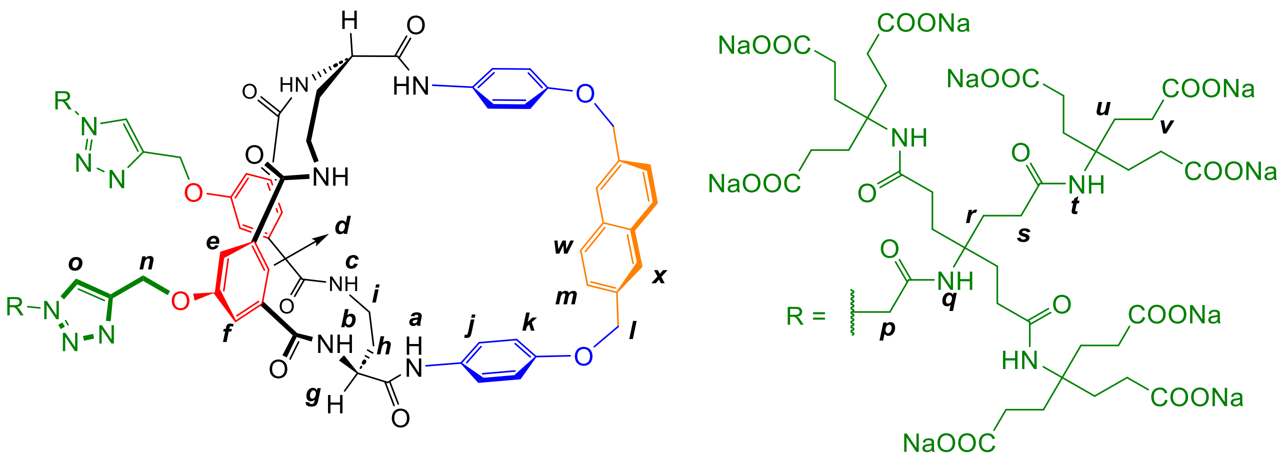

The assignment of the NMR spectrum in 9:1 H_2O/D_2O. was made with the help of 2D TOCSY and HSQC (see Supplementary Figures below). The side-chain carboxyls are shown as sodium salts but are expected to be only partially deprotonated.

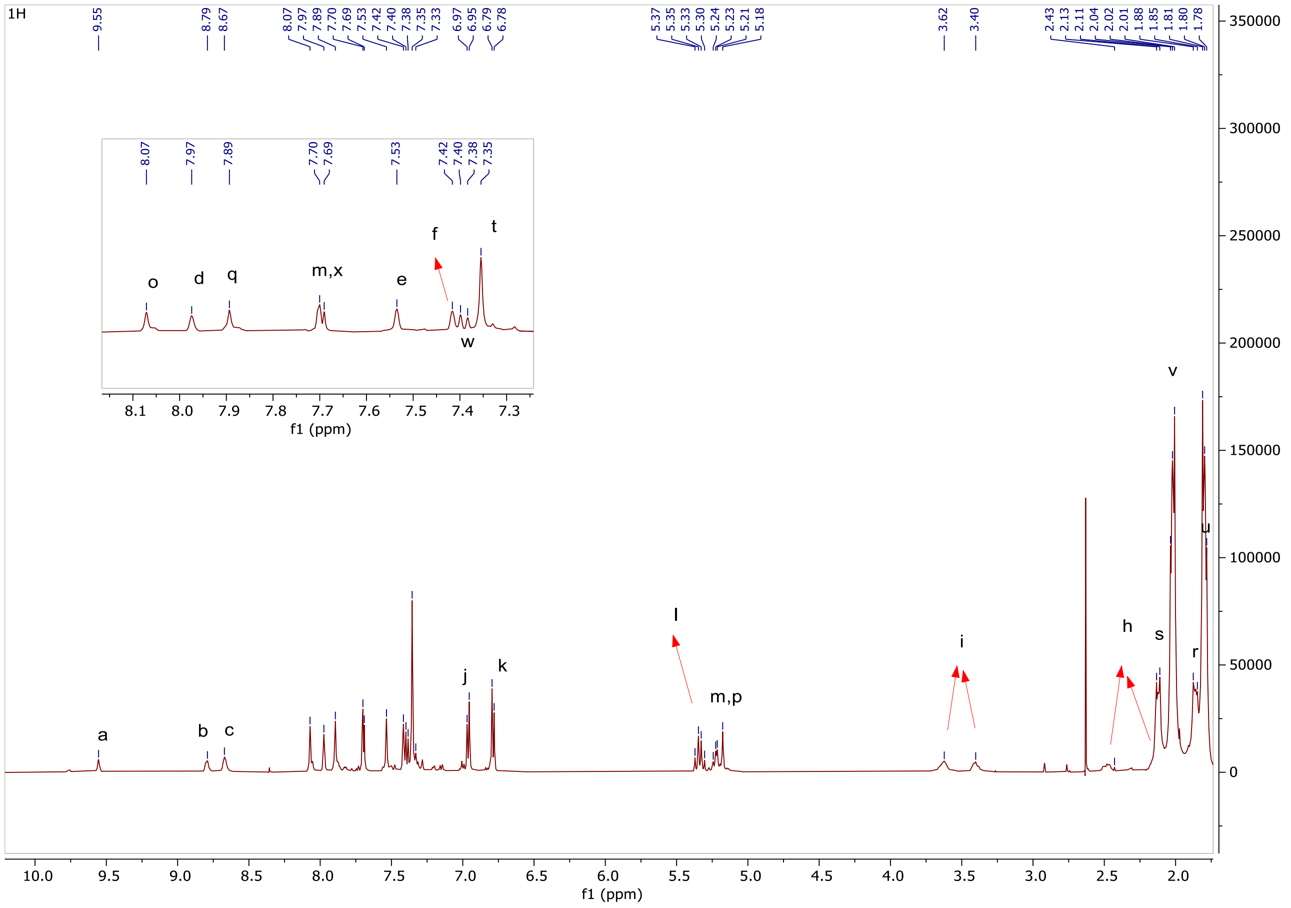

Figure S18. ^{1}H NMR spectrum (600 MHz) of receptor **11** in 9:1 H_2O/D_2O.

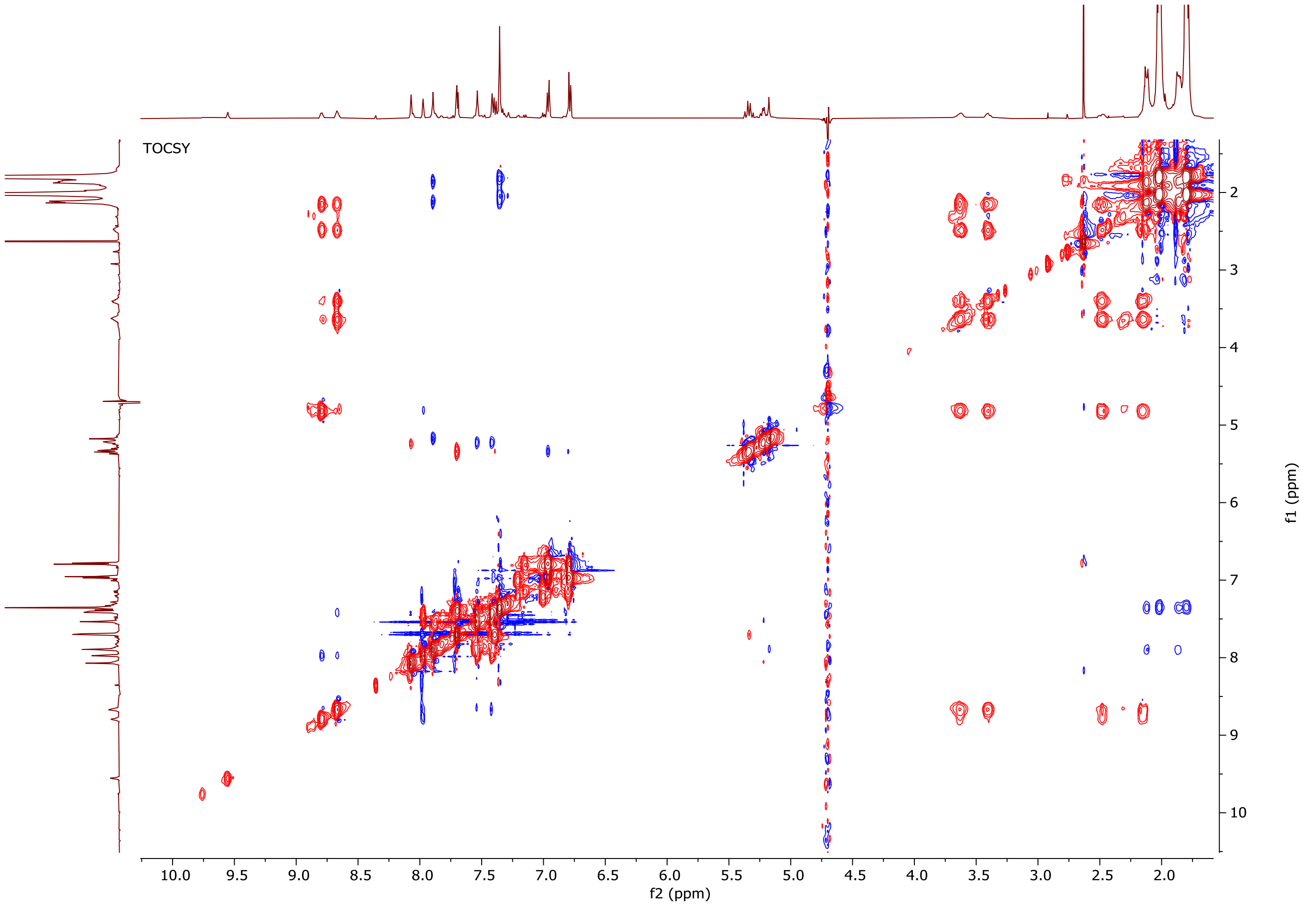

Figure S19. 2D TOCSY NMR spectrum (600 MHz) of receptor **11** in 9:1 H_2O/D_2O.

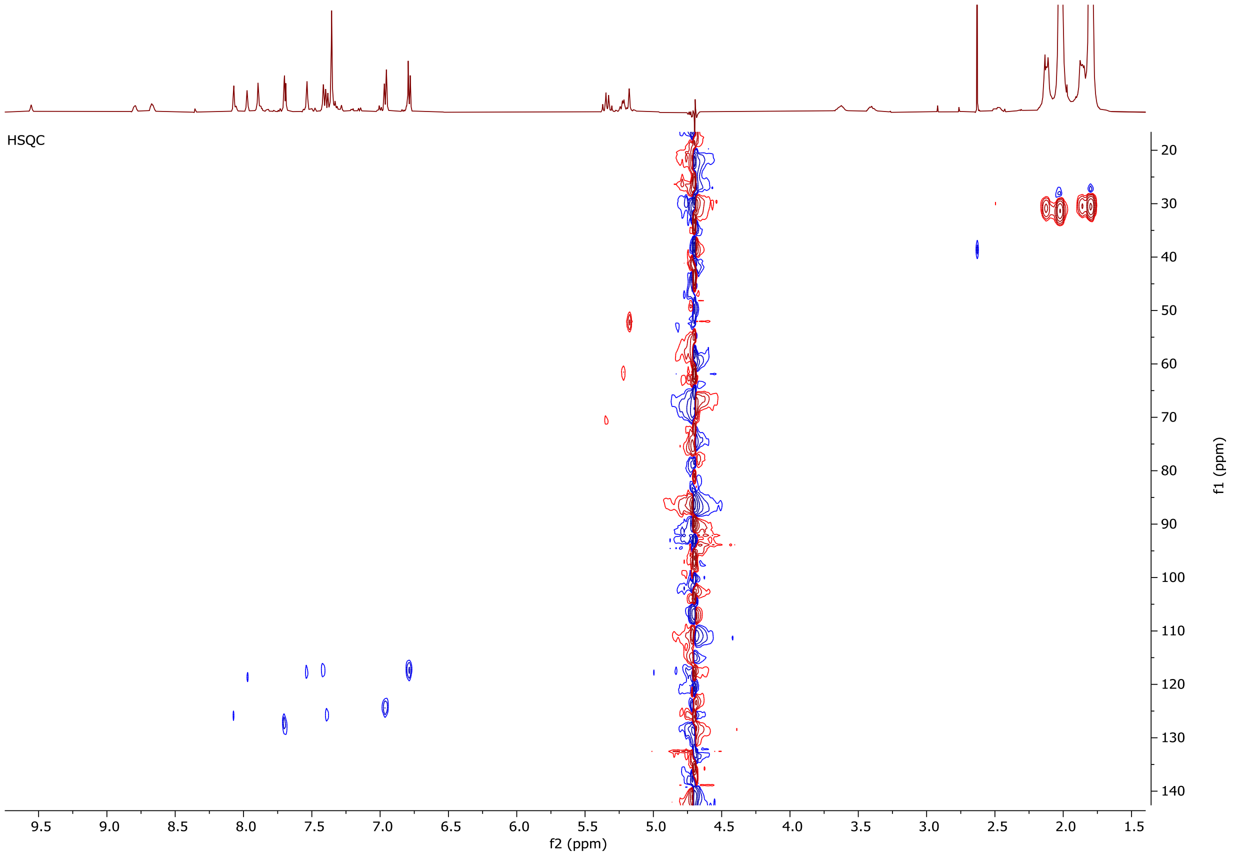

Figure S20. 2D HSQC NMR spectrum (600 MHz) of receptor **11** in 9:1 H_2O/D_2O.

Receptor 13

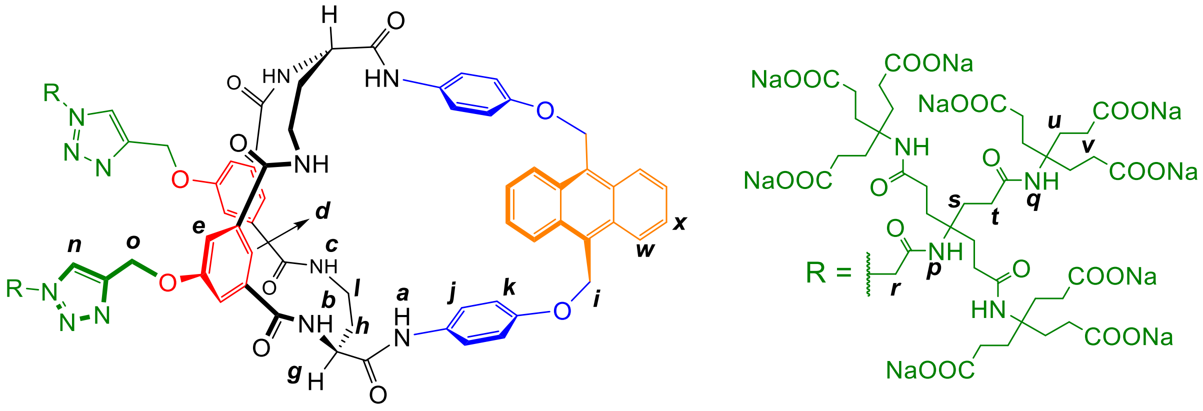

The assignment of the NMR spectrum in 9:1 H_2O/D_2O. was made with the help of the proton exchange experiment and 2D TOCSY(see Supplementary Figures below). The side-chain carboxyls are shown as sodium salts but are expected to be only partially deprotonated.

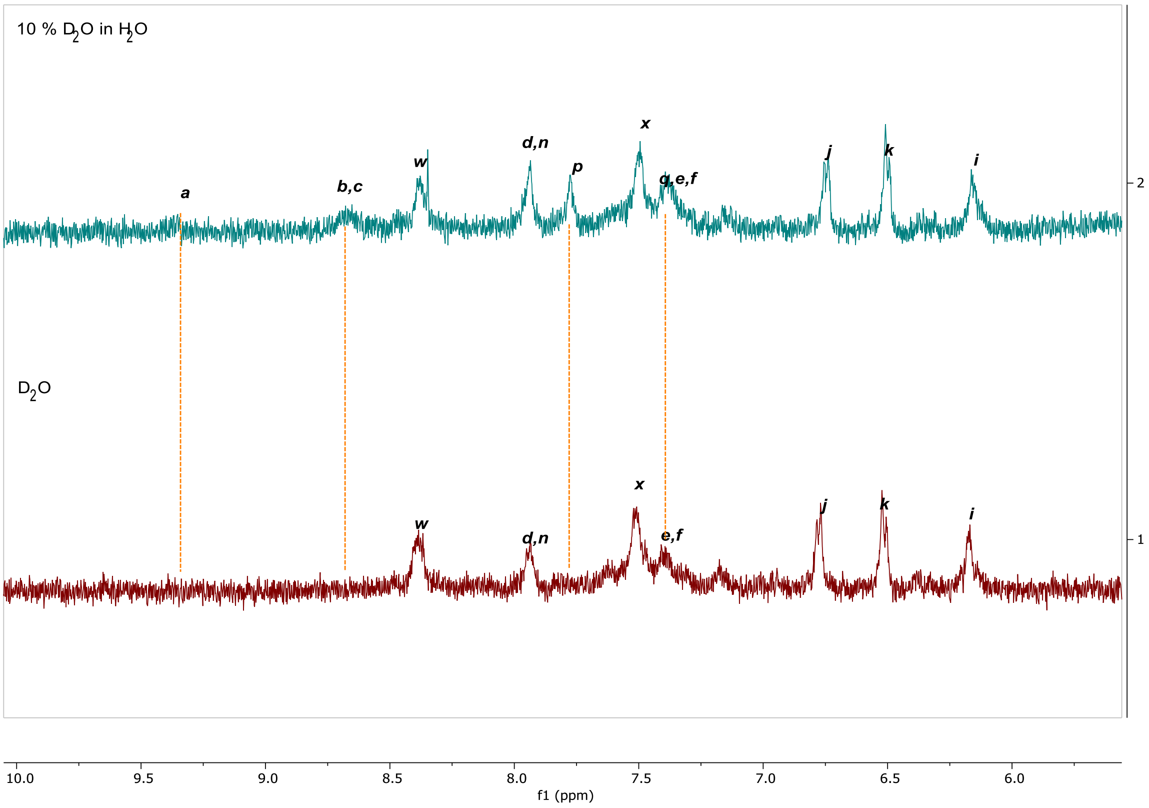

Figure S21. ^{1}H NMR spectrum of receptor (30 μM) under different aqueous conditions (10 % D_2O in H_2O and D_2O). Exchangeable protons were determined (protons *a*, *b*, *c*, *p* and *q*).

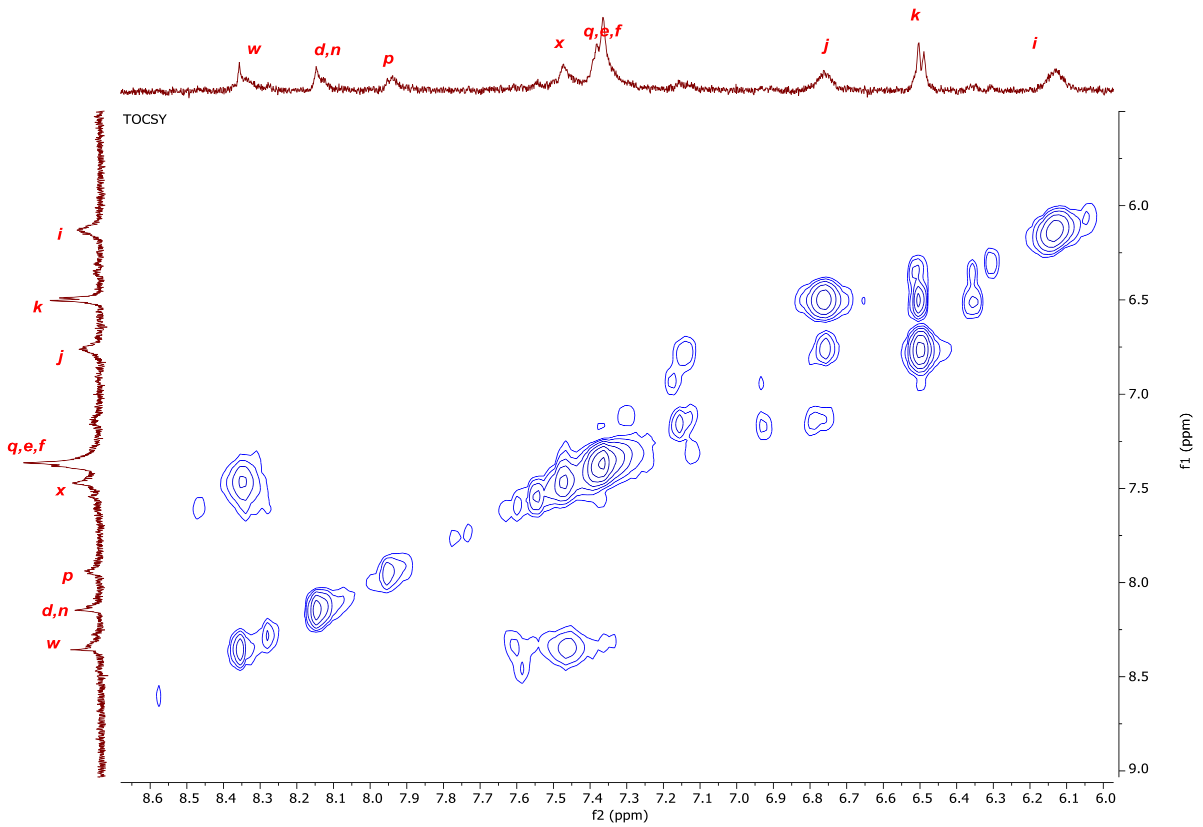

Figure S22. 2D TOCSY NMR spectrum (600 MHz) of receptor **13** in 1:9 D_2O/H_2O.

1.8 Aggregation studies

1.9.1 NMR dilution and DOSY studies

The solid receptors were dissolved in 9:1 H_2O/D_2O to make solutions with specific starting concentrations for each dilution study. 500 µL of the above solution was transferred into an NMR tube and the ^1H NMR spectrum (298 K) was acquired. The receptor solution was then diluted in the NMR tube by adding the same solvent, and a series of ^1H NMR spectra was obtained.

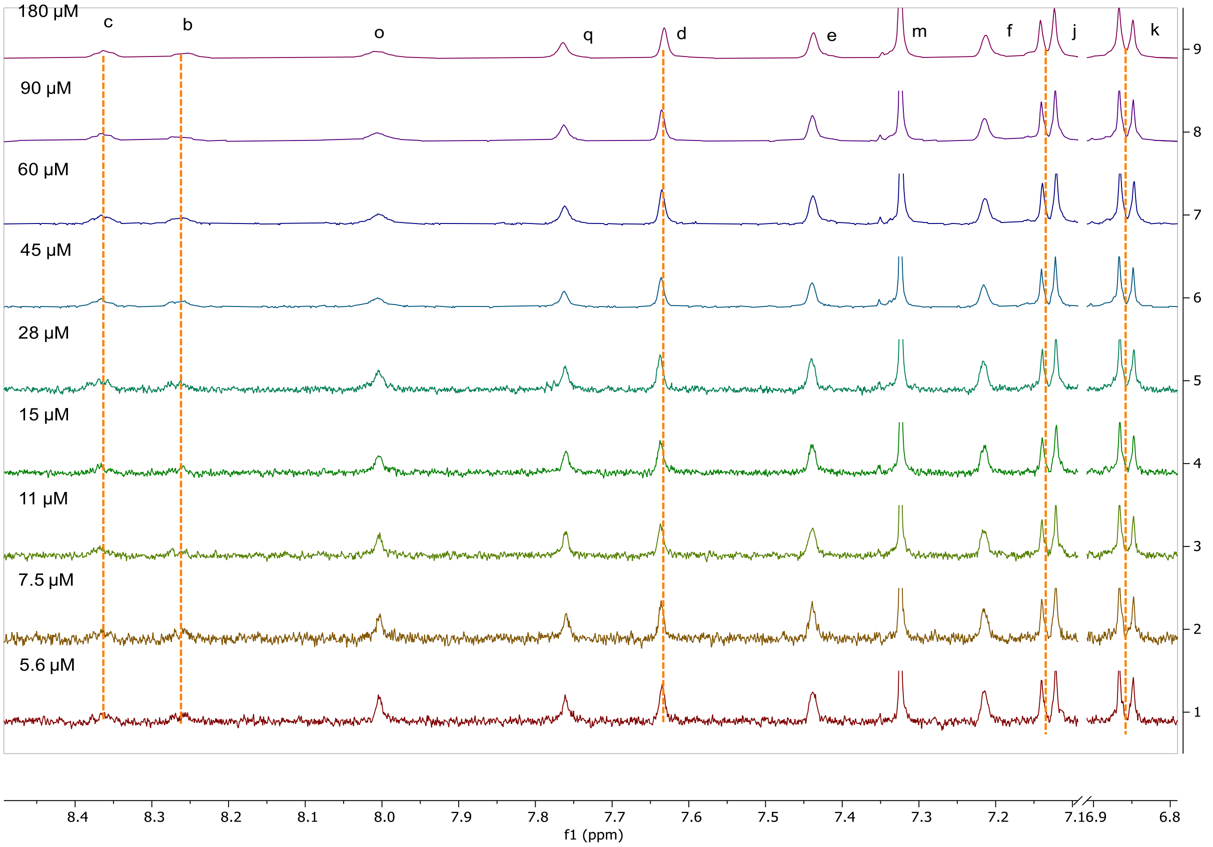

Figure S23. ^1H NMR spectra showing receptor **9** at various concentrations in 9:1 H_2O/D_2O (Yellow reference line has been put on the spectrum). The receptor is taken to be monomeric below 90 µM, and all binding studies were conducted below this concentration.

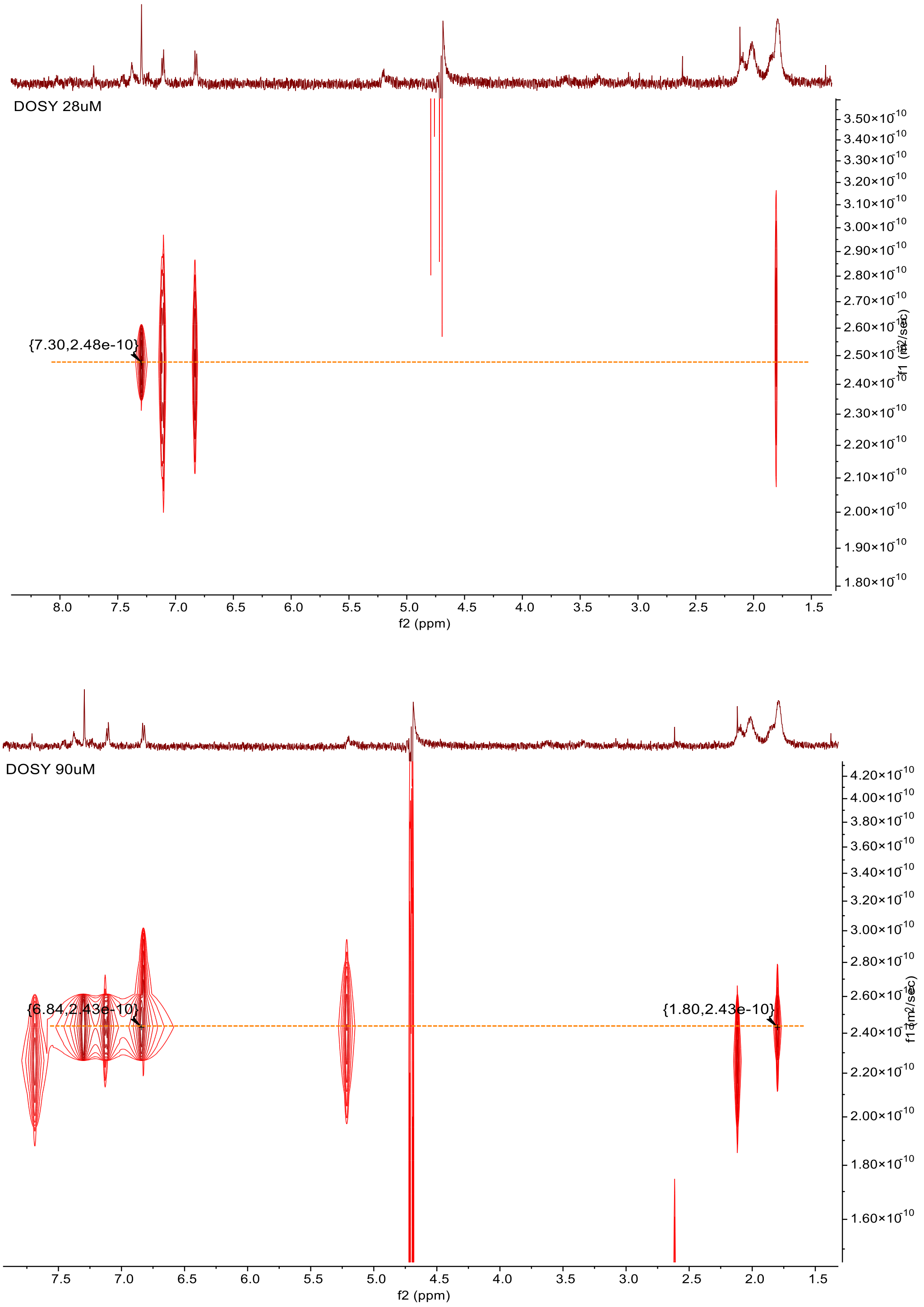

Figure S24. 2D DOSY NMR spectra (600 MHz, 9:1 H_2O/D_2O) of receptor **9** at 298 K. Diffusion coefficients of 2.48 x 10^{-10} and 2.43 x 10^{-10} m^2s^{-1} were measured for concentrations of 28 μM (top) and 90 μM (bottom). Using the Stokes-Einstein equation these values can be converted to the hydrodynamic radius, giving estimated diameters of 1.98 nm and 2.02 nm respectively.

Stokes-Einstein equation: $R_H = kT/6\pi\eta D$

Where D = diffusion coefficient (from DOSY in m^2s^{-1}*), k = Boltzmann's constant (1.38 x* 10^{-23} $m^2kg\ s^{-2}\ K^{-1}$*), T = temperature (298 K), η = solvent viscosity (0.891 x* 10^{-3} $kg\ m^{-1}\ s^{-1}$ *for* H_2O*),* R_H *= hydrodynamic Radius (m).*

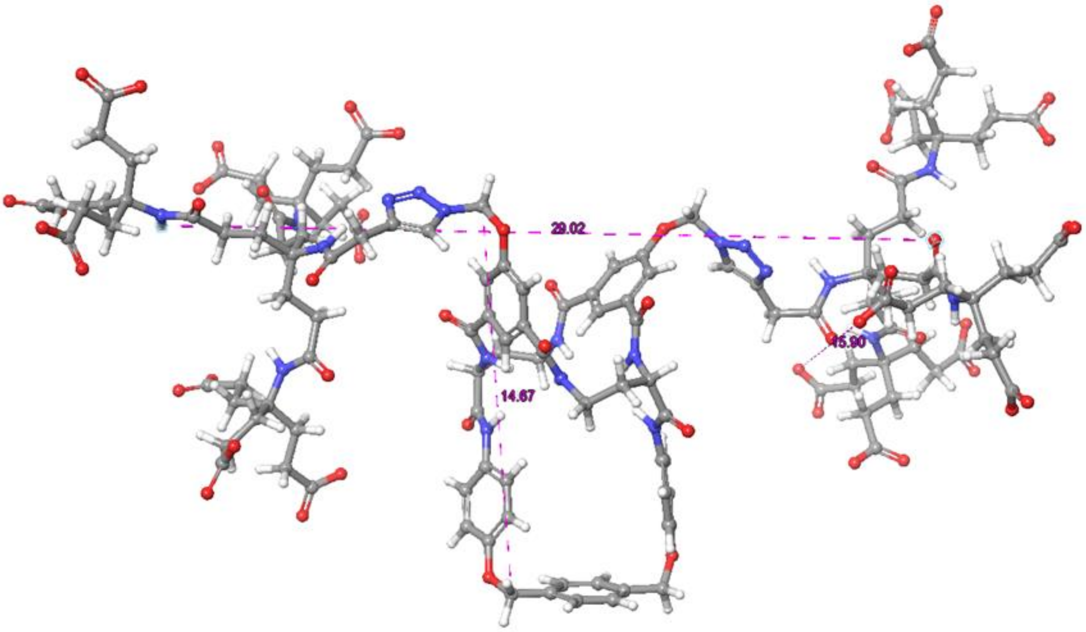

Figure S25. Dimensions of receptor **9** as judged from a molecular model (1.47 nm * 2.90 nm * 1.59 nm).

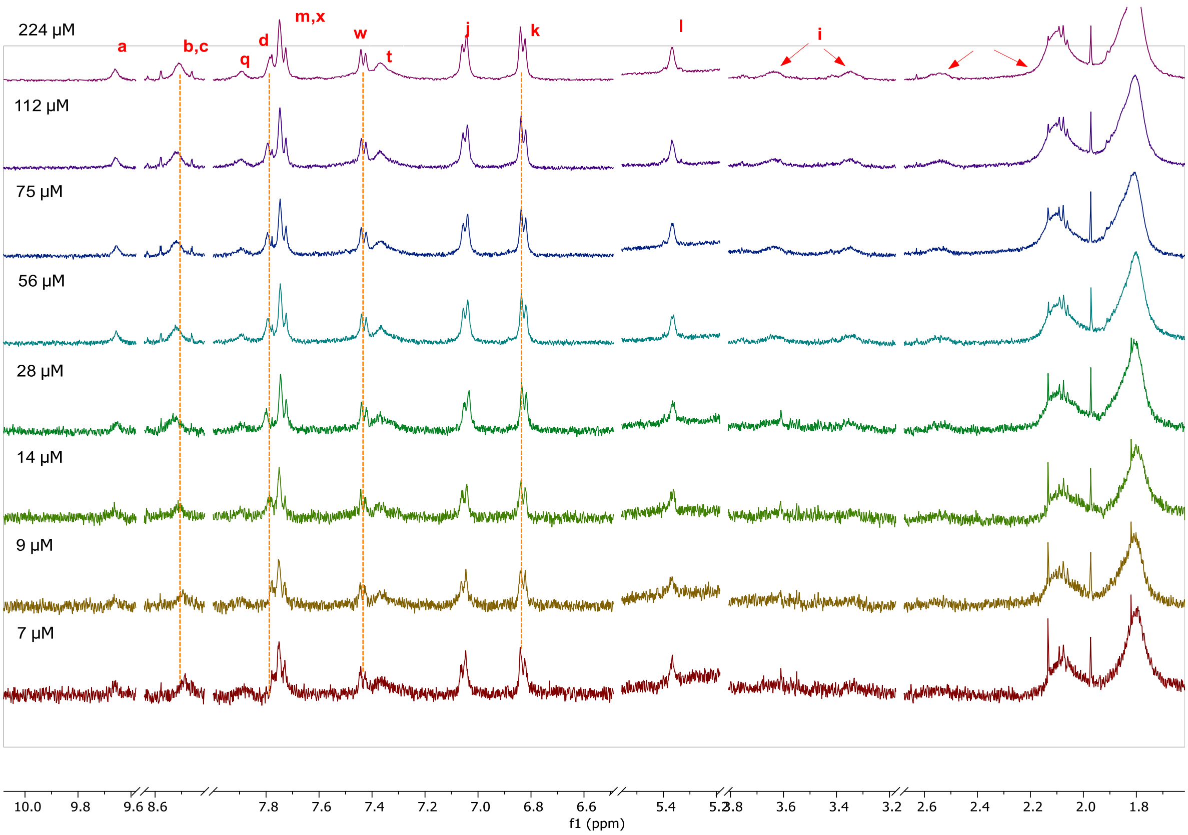

Figure S26. ^1H NMR spectra showing receptor **11** at various concentrations in 9:1 H_2O/D_2O. The receptor is taken to be monomeric below 75 μM, and all binding studies were conducted below this concentration.

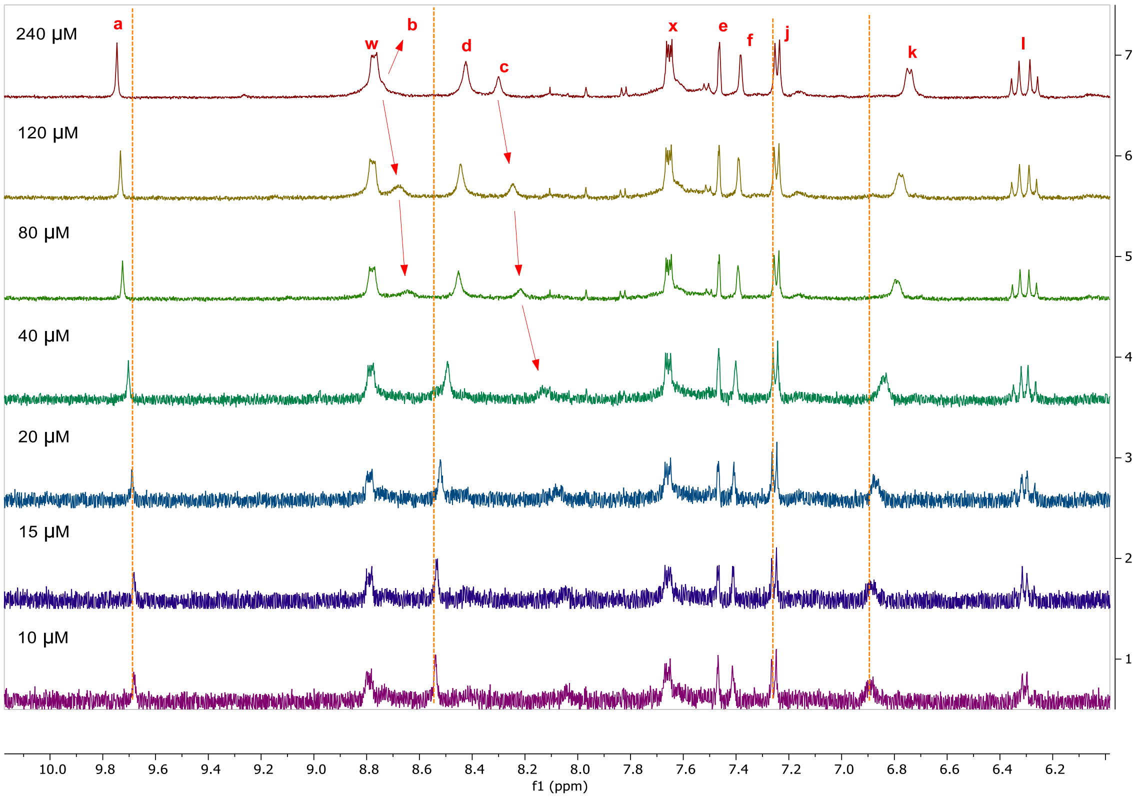

Figure S27. ^1H NMR spectra showing receptor **12** at various concentrations in DMSO-d6. The receptor is taken to be monomeric below 15 μM, and all binding studies were conducted below this concentration.

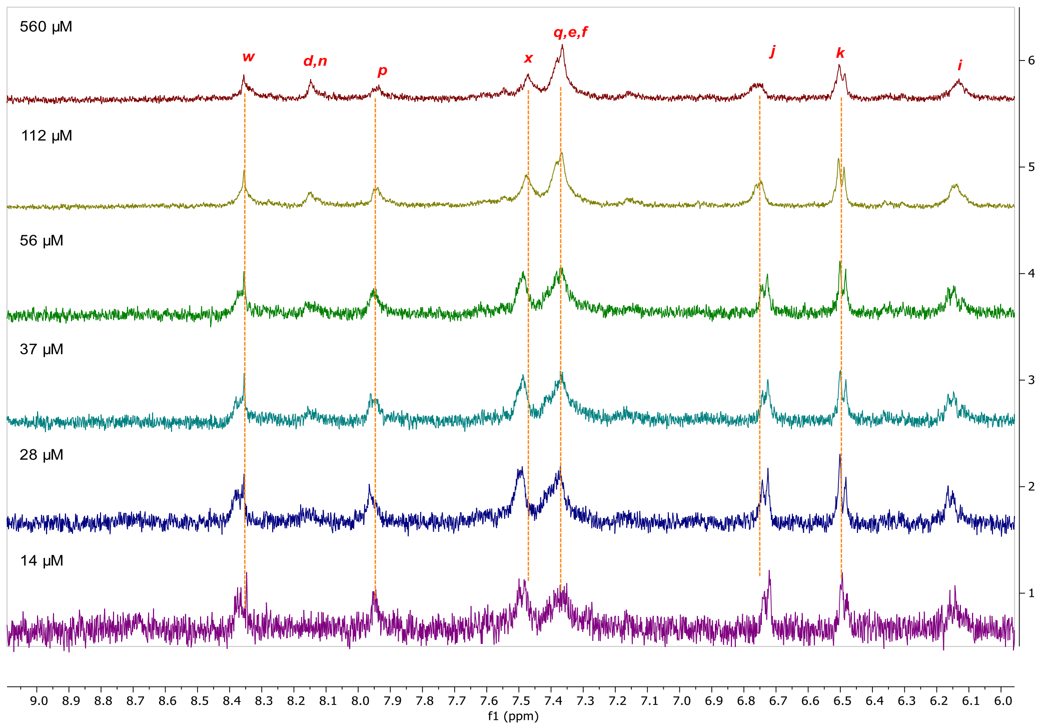

Figure S28. ^1H NMR spectra showing receptor **13** at various concentrations in 9:1 H_2O/D_2O. The receptor is taken to be monomeric below 56 μM, and all binding studies were conducted below this concentration.

1.9.2 UV-Vis and fluorescence dilution studies

UV-Vis and fluorescence dilution studies were carried out on an Agilent Cary 300 spectrometer and a Horiba Fluoromax spectrofluorometer in quartz cuvettes (3 mL, 10 mm path length). All dilutions were performed in H_2O solution at 298K.

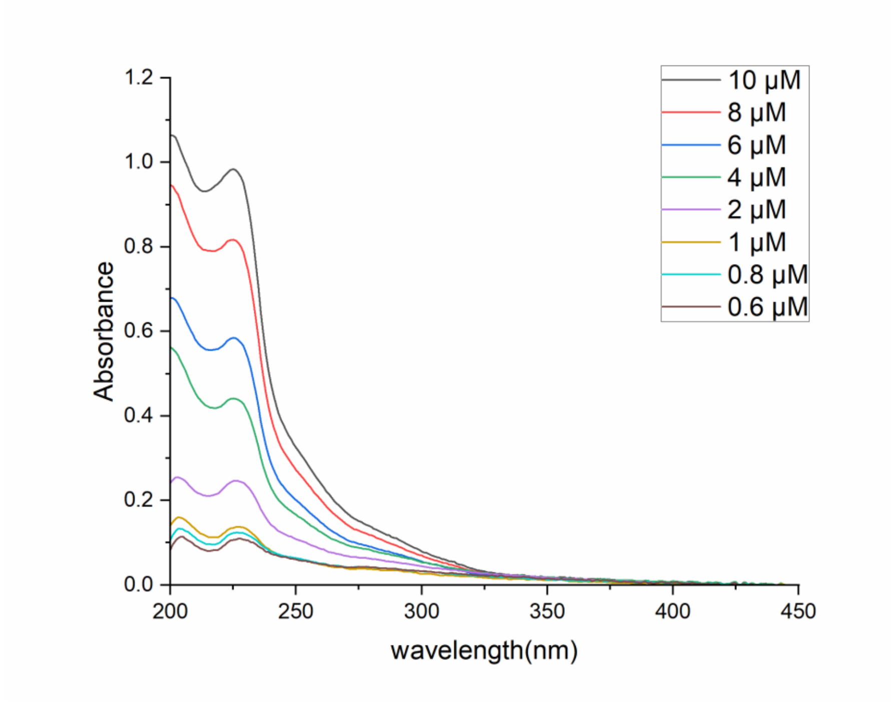

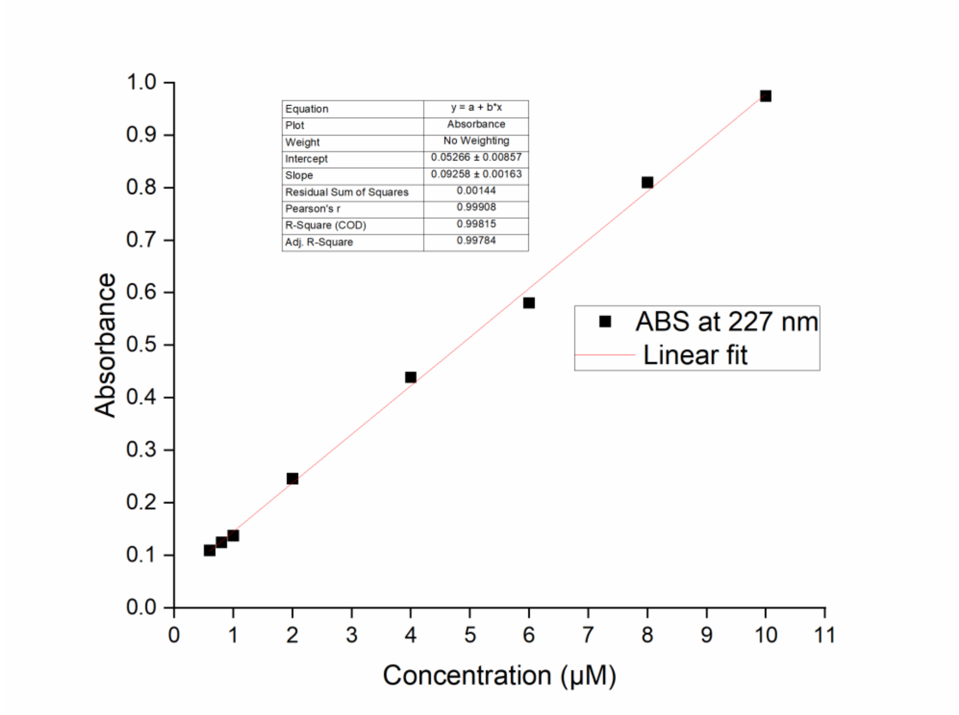

Figure S29. Top). UV-Vis spectrum of receptor **11** at various concentrations in H_2O. Bottom). The UV absorption at 227 nm for receptor **11**, plotted against increasing receptor concentration. A linear relationship is observed below 10 μM.

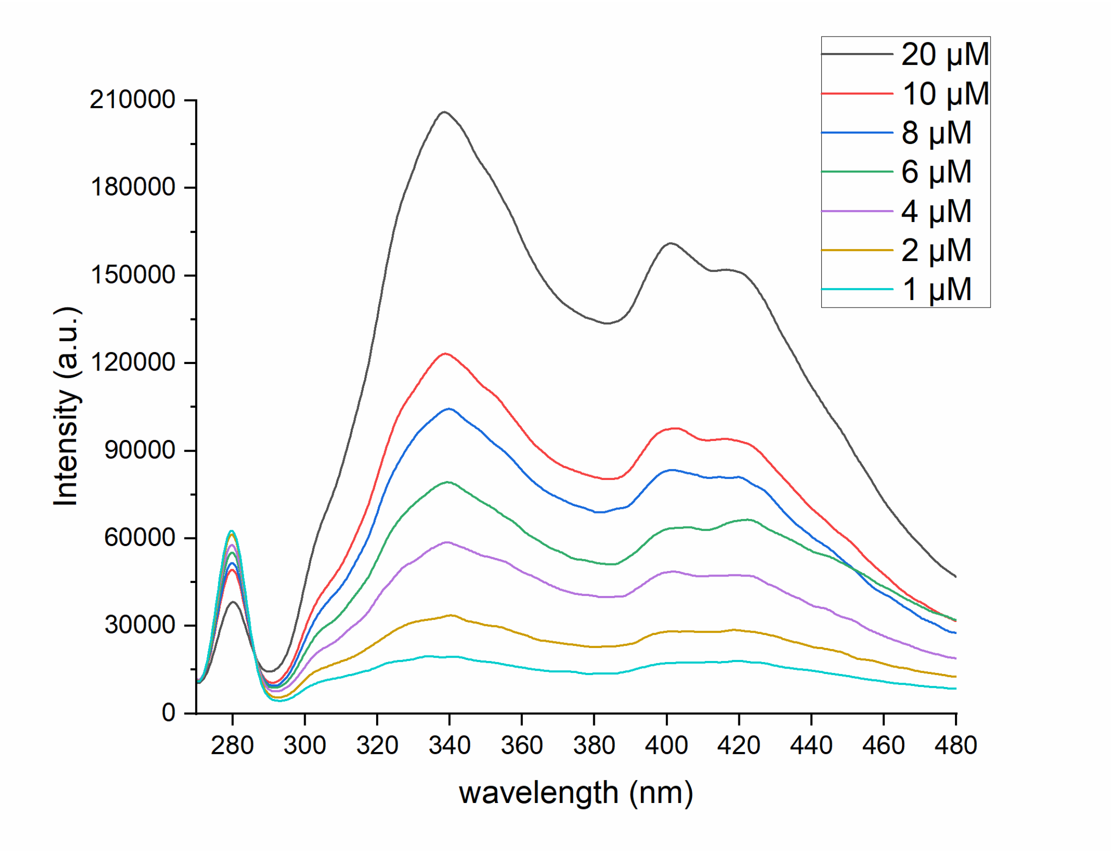

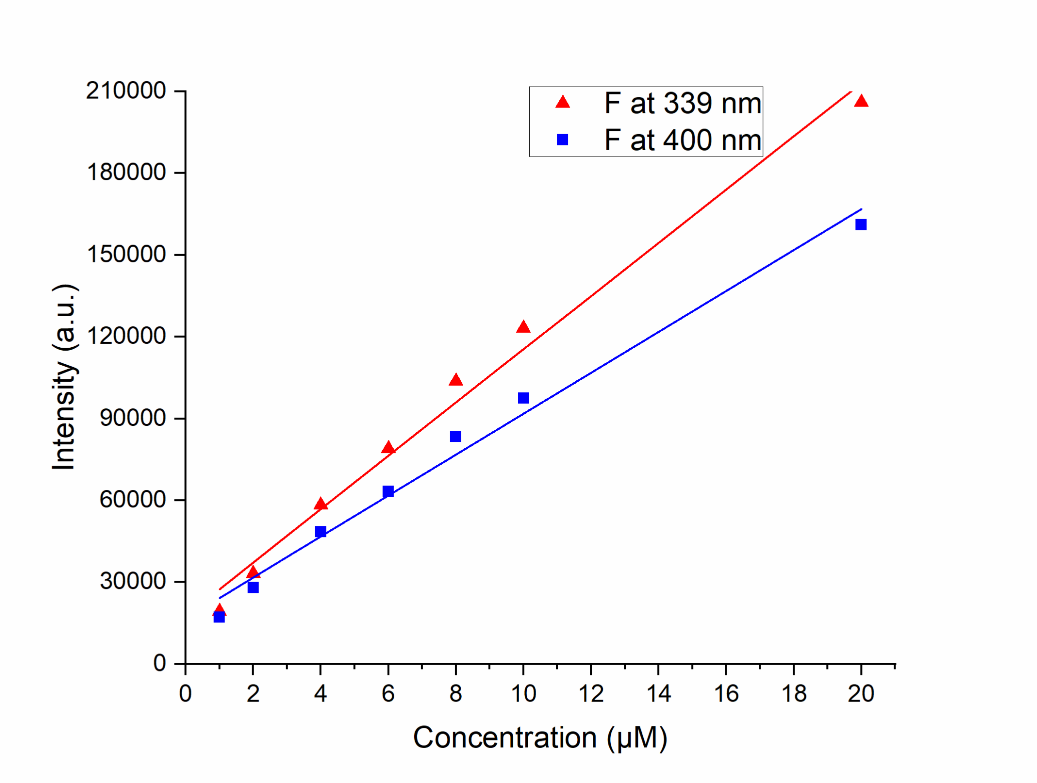

Figure S 30. Top). Fluorescence spectrum of receptor **11** at various concentrations in H_2O. Bottom). Fluorescence emission intensity at 339 nm and 400 nm for receptor **11**, plotted against increasing receptor concentration. A linear relationship is observed below 20 μM.

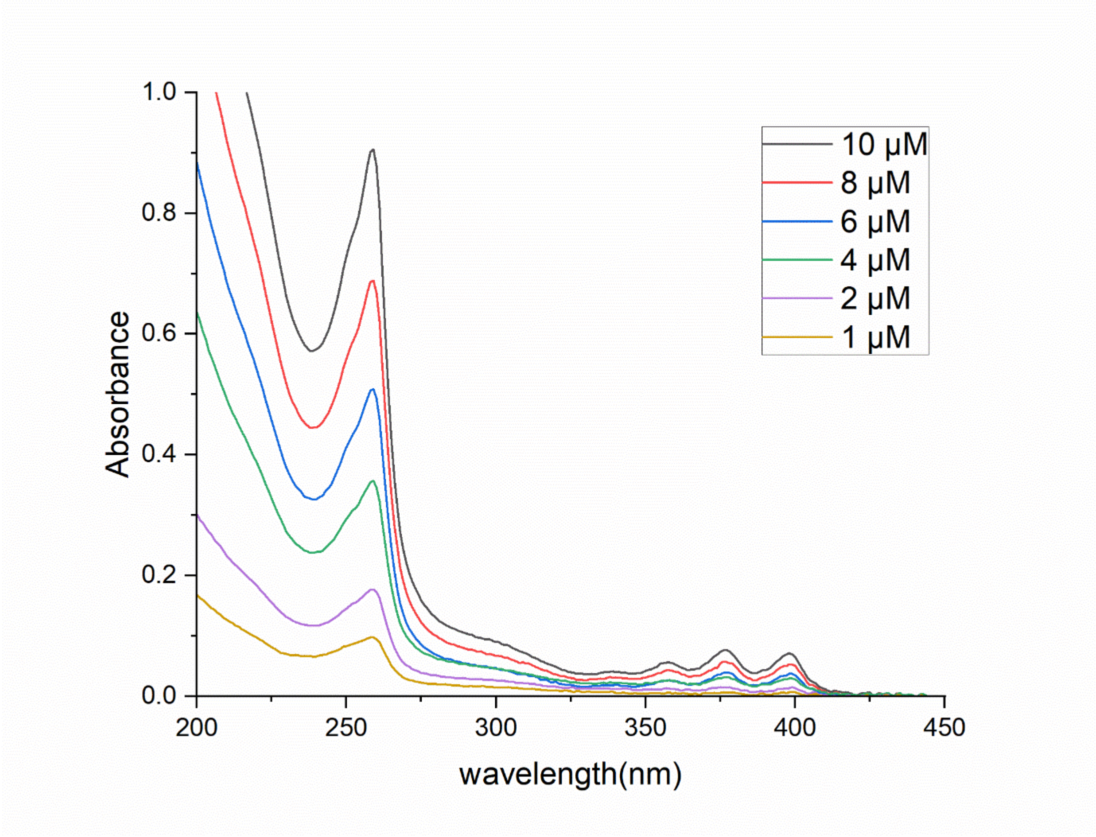

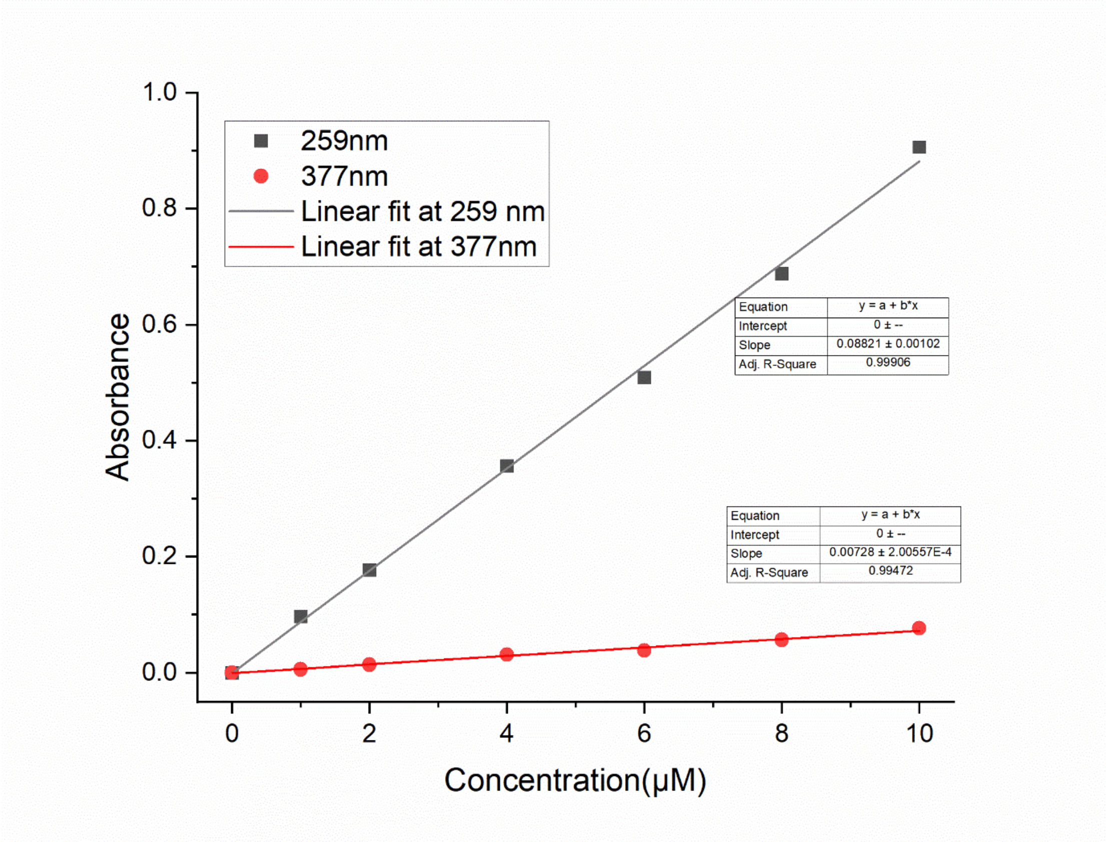

Figure S31. Top). UV-Vis spectrum of receptor **13** at various concentrations in H_2O. Bottom). The UV absorption at 259 nm and 377 nm for receptor **13**, plotted against increasing receptor concentration. A linear relationship is observed below 10 μM.

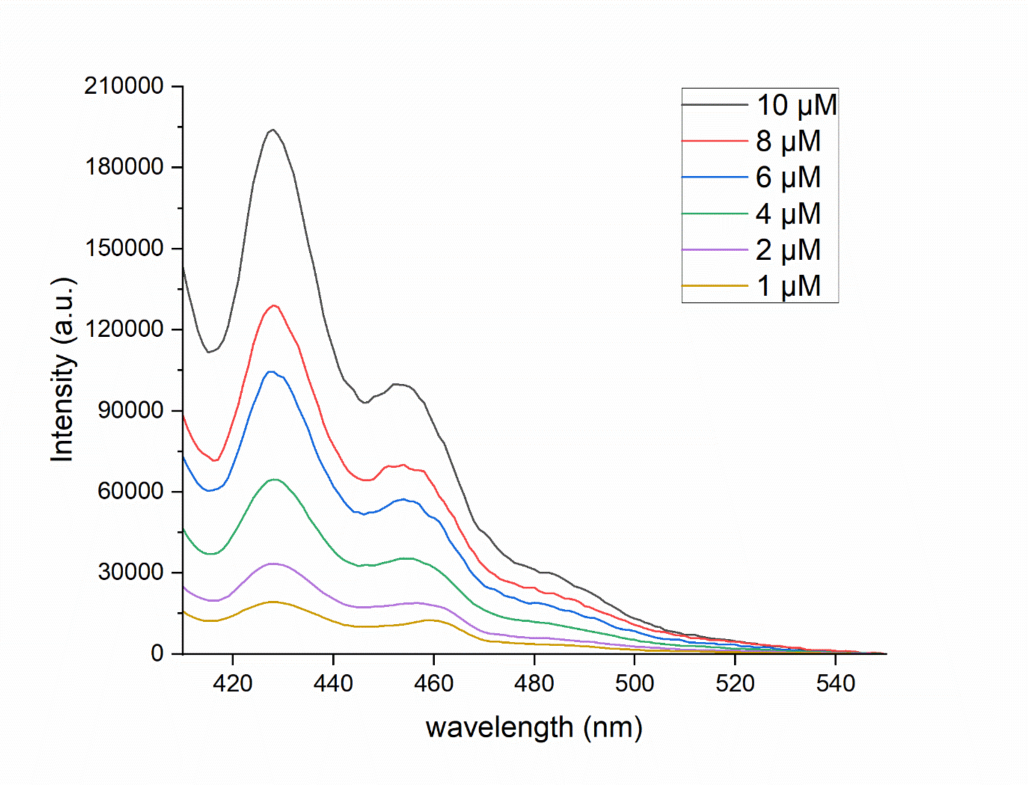

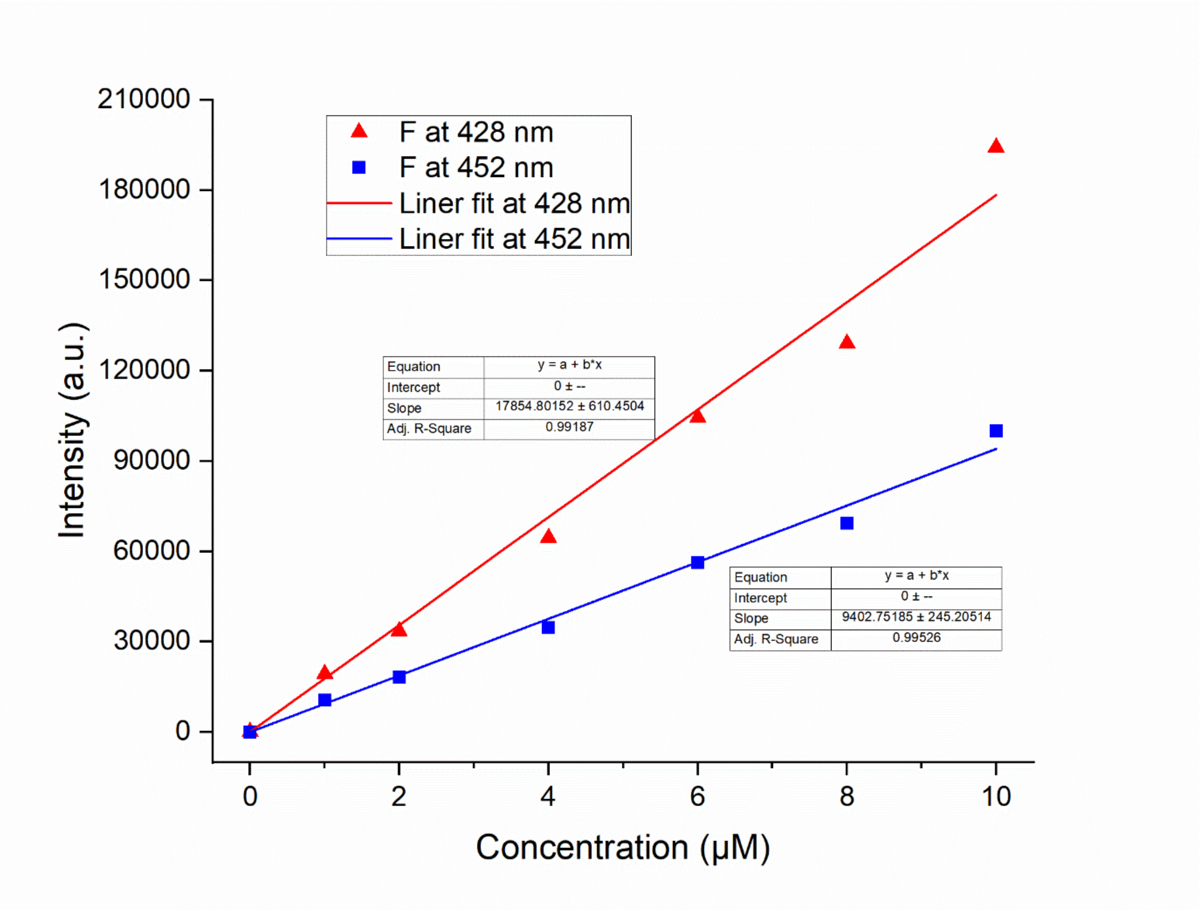

Figure S 32. Top). Fluorescence spectrum of receptor **13** at various concentrations in H_2O. Bottom). Fluorescence emission intensity at 428 nm and 452 nm for receptor **13**, plotted against increasing receptor concentration. A linear relationship is observed below 10 μM.

1.9 pH titrations of receptors

1.10.1 NMR pH titrations

pH near-neutral solutions of receptors were made by dissolving the solid receptors into a specific amount to in 9:1 H_2O/D_2O (typically 1000 µL). Solid NaOH was then dissolved in the above receptor solutions to make 10 mM, 100 mM and 1 M NaOH aqueous solution (100 µL each). The pH titration started by transferring the neutral receptor solution (500 µL) into an NMR tube and adding an aliquot of the prepared NaOH solutions. The NMR tube was shaken after the addition and then the ^1H NMR spectrum was acquired at 298 K. After the acquisition, the titration solution was transferred to a vial for pH measurement before putting it back into the same NMR tube for the next addition.

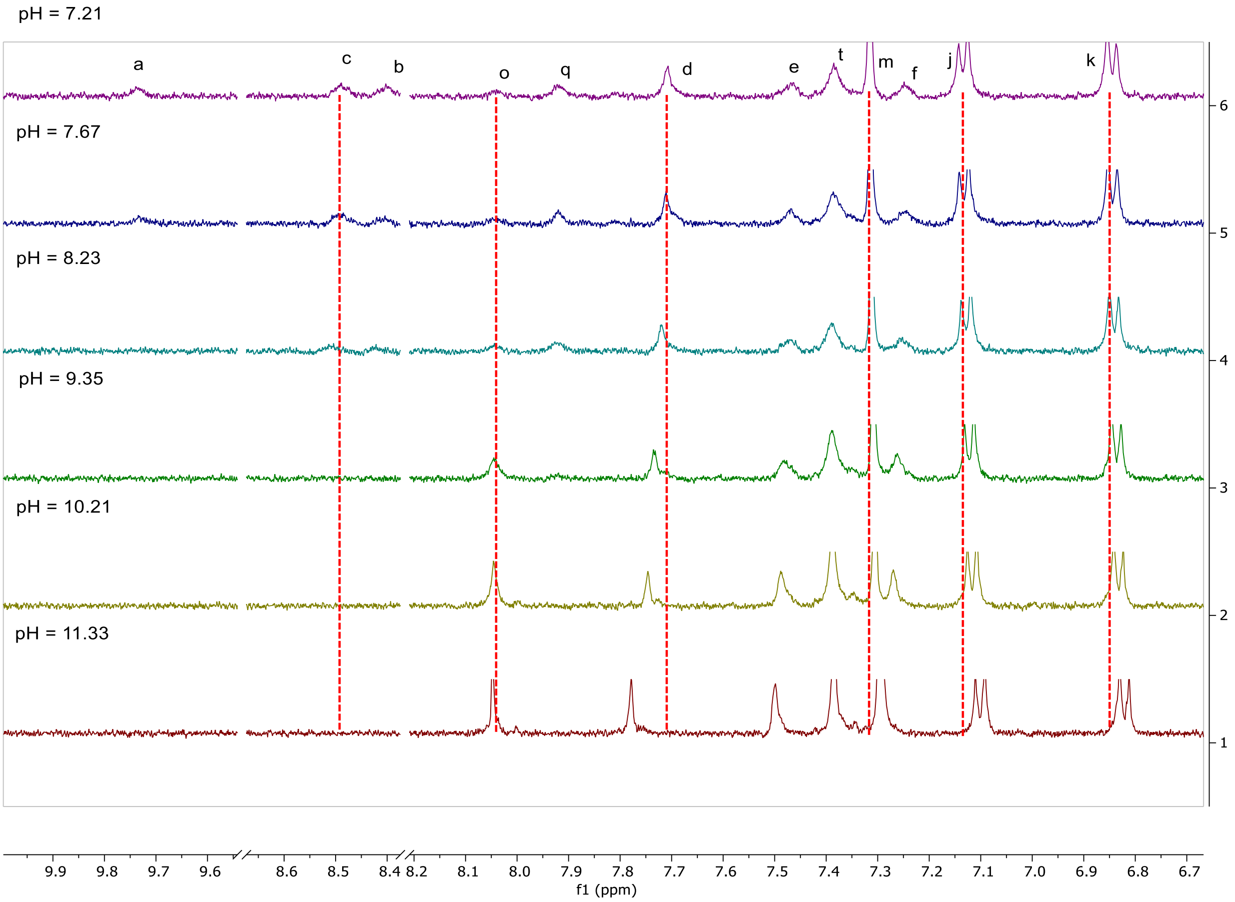

Figure S33. ^1H NMR spectra showing receptor **9** (16 µM) at various pH in 9:1 H_2O/D_2O.

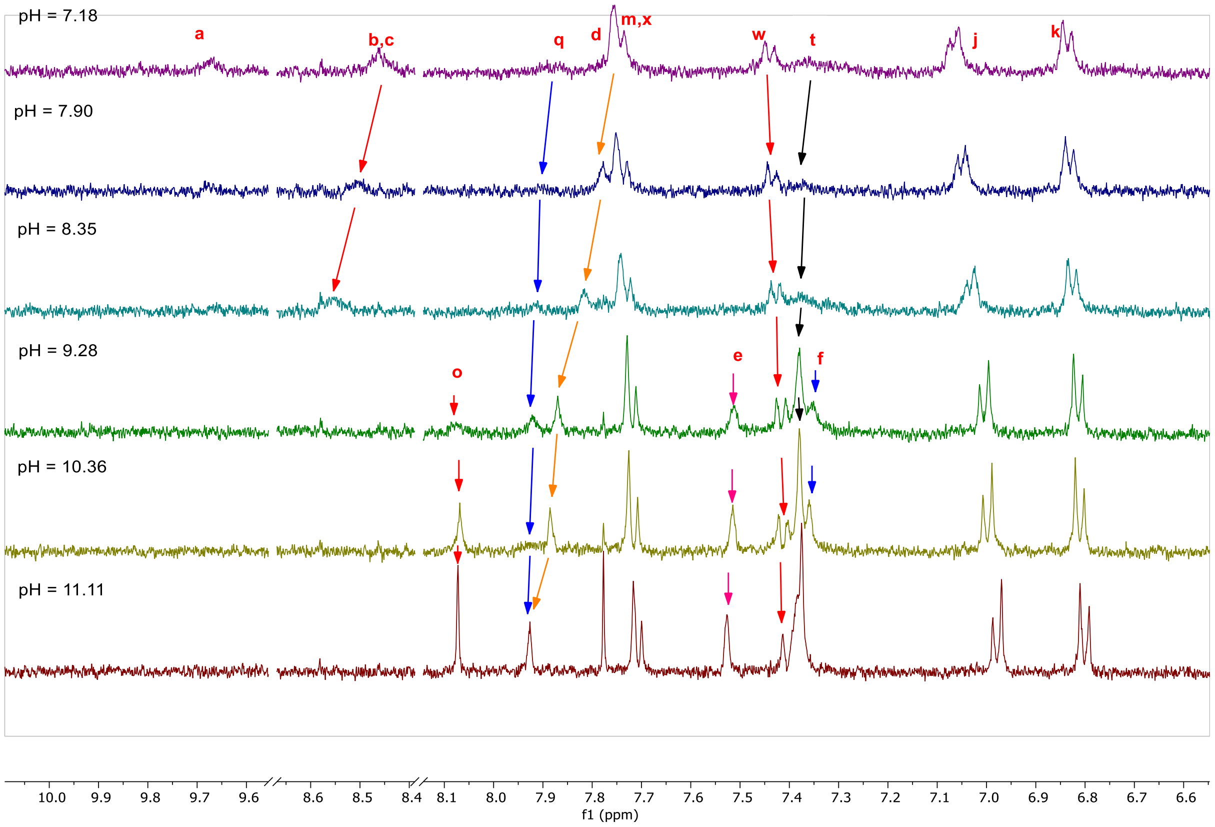

Figure S34. ^{1}H NMR spectra showing receptor **11** (16 μM) at various pH in 9:1 H_2O/D_2O.

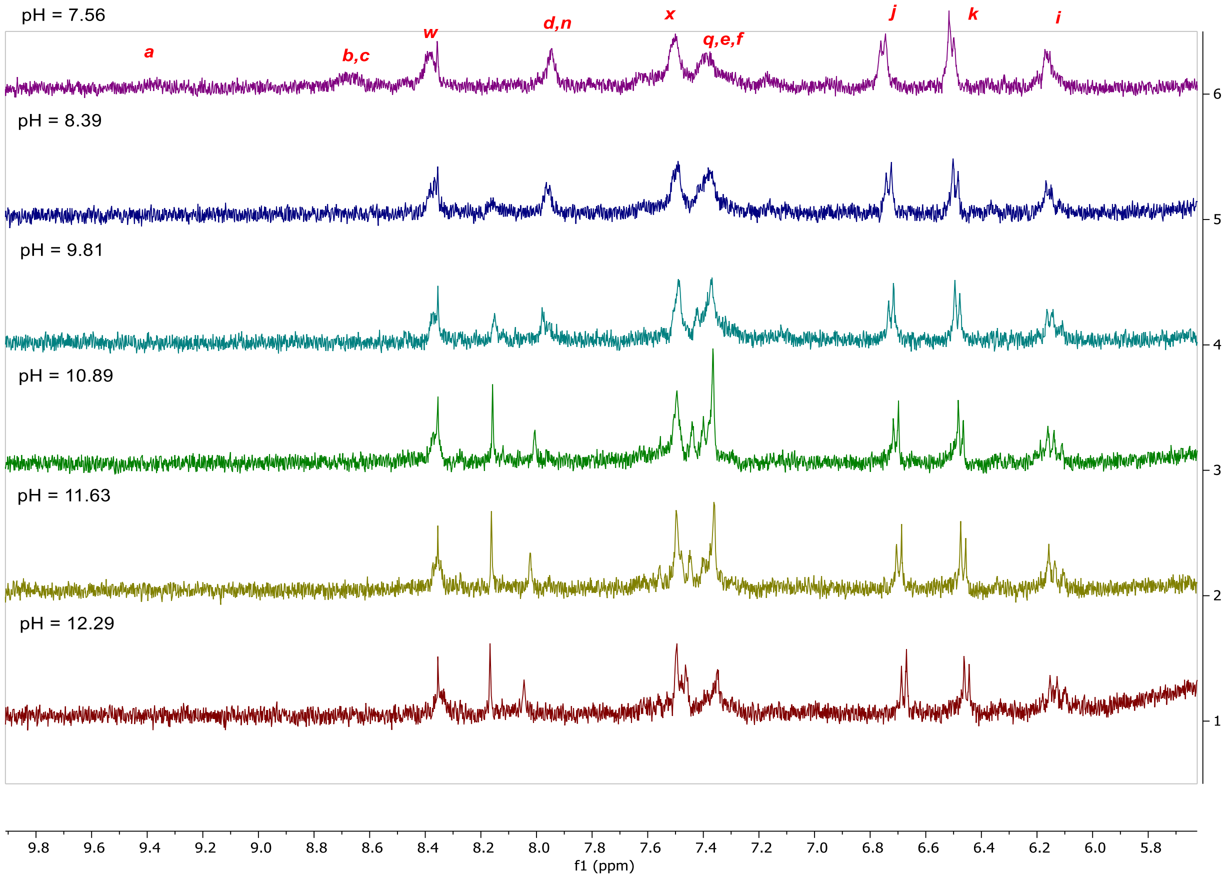

Figure S35. ^{1}H NMR spectra showing receptor **13** (25 μM) at various pH in 9:1 H_2O/D_2O.

1.10.2 Fluorescence pH titrations

pH near-neutral solutions of receptors were made by dissolving the solid receptors into H_2O. Solid NaOH was then dissolved in the above receptor solutions to make 10 mM, 100 mM and 1 M NaOH aqueous solution (100 μL each). The pH titration started by transferring the neutral receptor solution (2500 μL) into a quartz cuvette (3 mL, 10 mm path length) and adding an aliquot of the prepared NaOH solutions. The mixture was stirred for 3 mins and then the fluorescence spectrum was acquired at 298 K. After the acquisition, the titration solution was transferred to a vial for pH measurement before putting it back into the same cuvette for the next addition.

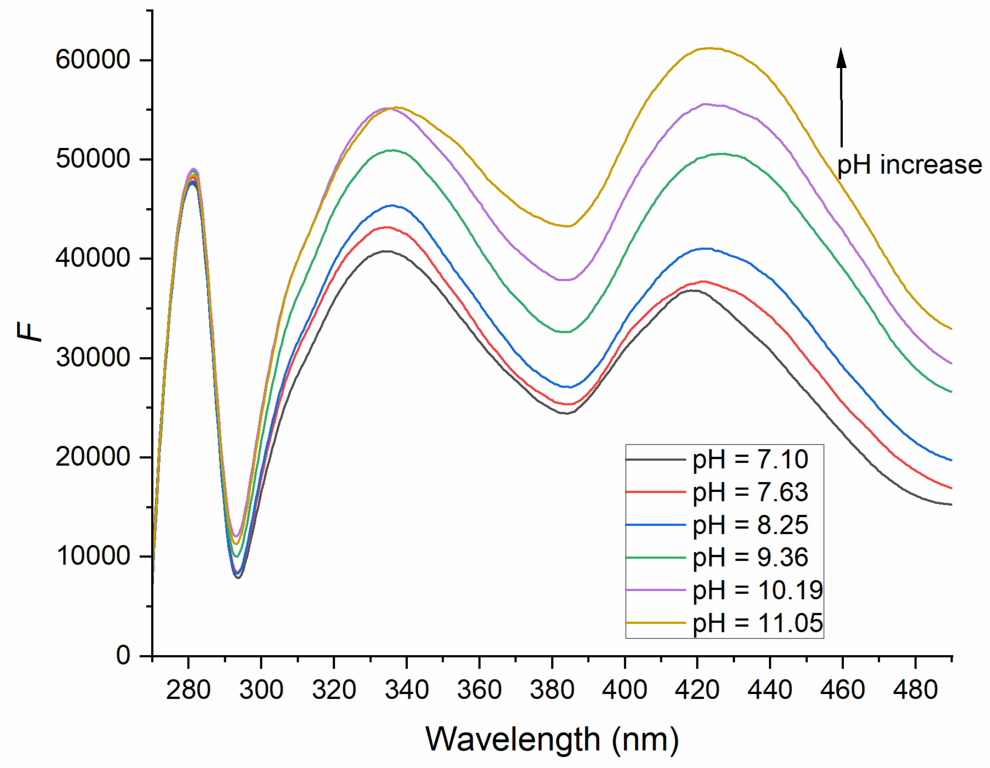

Figure S36. Fluorescence spectra showing receptor **11** (2 μM) at various pH in H_2O.

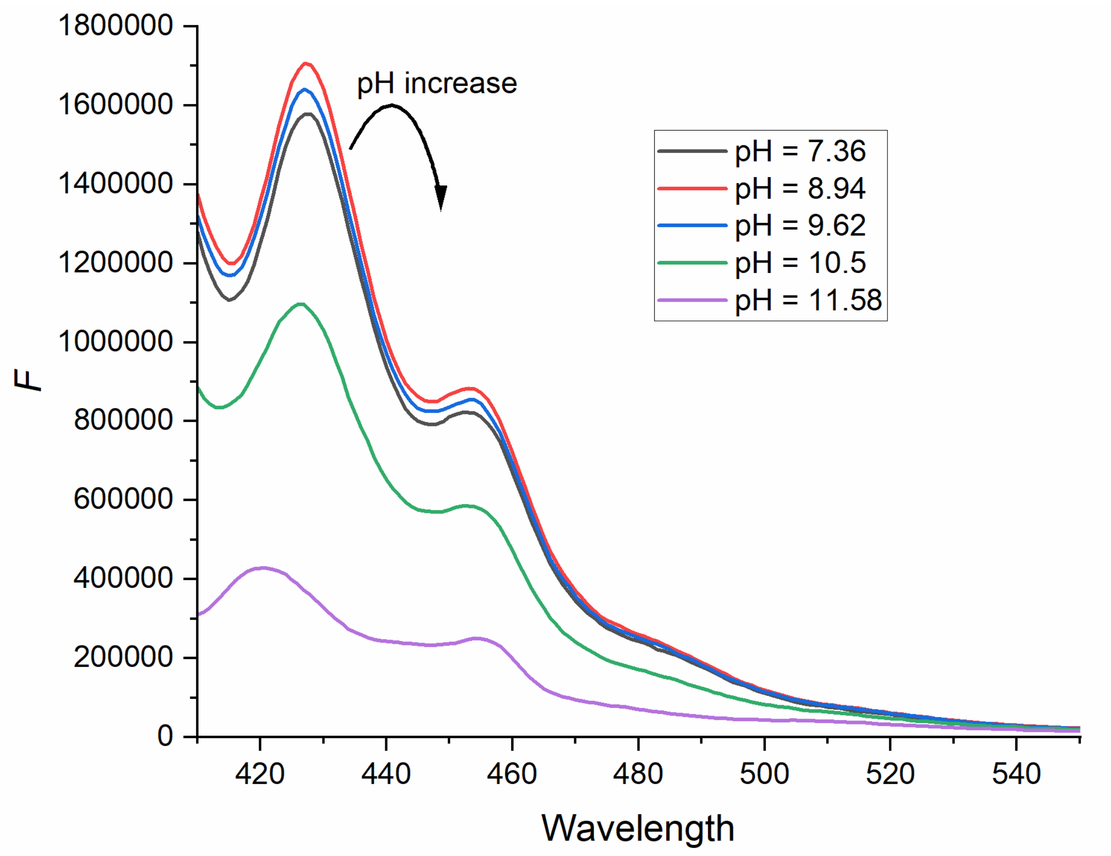

Figure S37. Fluorescence spectra showing receptor **13** (2 μM) at various pH in H_2O.

2. Binding studies

Titrations in aqueous medium were conducted at a constant near-neutral pH in H_2O or 9:1 H_2O/D_2O. The host stock solution was first prepared by dissolving a weighed amount of host solid in H_2O or 9:1 H_2O/D_2O and the pH was checked to be near-neutral (the pH ranged from 7.35 to 7.50 for different hosts). For carboxylate guests, the guest stock solution was then prepared by dissolving sodium carboxylate salt in H_2O or 9:1 H_2O/D_2O and adjusting pH to match the host solution as closely as possible ($pH_{guest} = pH_{host} \pm 0.15$) by adding the conjugate acid. Other guest (NaCl, NaBr, NaI, Na_2SO_4 and $NaNO_3$) stock solutions were prepared by dissolving the compound in H_2O or 9:1 H_2O/D_2O and confirming the neutrality of pH.

2.1 Nuclear Magnetic Resonance (NMR) titrations

^1H NMR titrations were performed on Bruker Advance III HD Cryo 500 MHz or Bruker Neo Cryo 600 MHz spectrometers.

For titrations in organic solvent (DMSO-d^6), solutions of receptor at known concentrations were prepared in an NMR tube. Aliquots of a solution of receptor at the same concentration and guest were added. The receptor concentration was therefore held constant while the guest concentration was increased. The NMR tube was shaken after each addition and the ^1H NMR spectra acquired at 298 K.

For aqueous titrations in 9:1 H_2O/D_2O, before each binding experiment, two solutions for titration were prepared using the near-neutral stock solutions. The first consisted of 500 μL of host solution at a concentration below the association threshold. The second solution consisted of 500 μL of host, at the same concentration as the first solution, mixed with guest. Both solutions were made in 1.5 mL vials, and the near-neutral pH was confirmed. During the titration, the host solution (500 μL) was transferred to an NMR tube and aliquots of host-guest mixture were added precisely using Gilson pipettes. This method holds the receptor concentration constant while the guest concentration was increased, with the pH staying near-neutral. The NMR tube was shaken after each addition and the ^1H NMR spectra acquired at 298 K. At the end of each titration, the solution in the NMR tube was transferred into a vial and the pH was measured again confirming negligible changes ($\Delta pH < \pm 0.1$) during the experiment.

Association constants were determined by monitoring the change in chemical shift ($\Delta\delta$) for selected protons belonging to the receptor species. The $\Delta\delta$ values were analysed to give binding constants K_a using Bindfit[6], employing the 1:1 binding model for all the hosts and the guests.

2.1.1 Receptor 7 in DMSO-*d*6

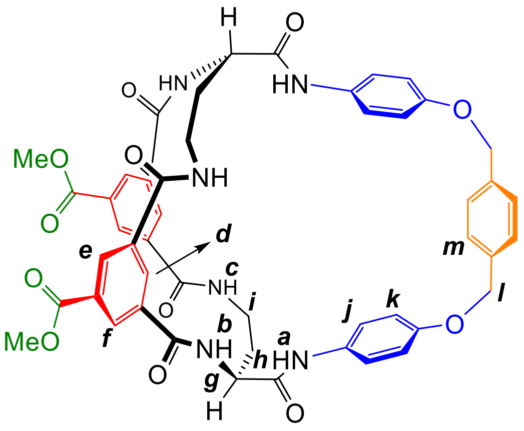

Acetate

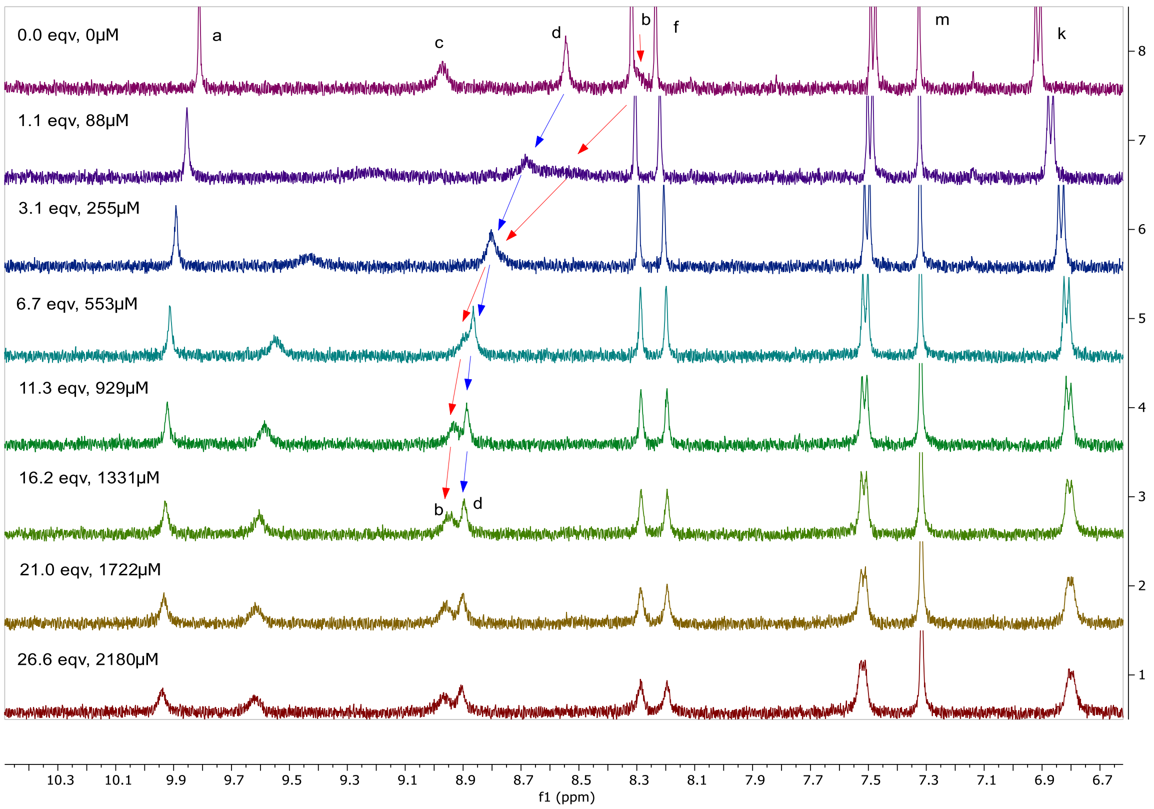

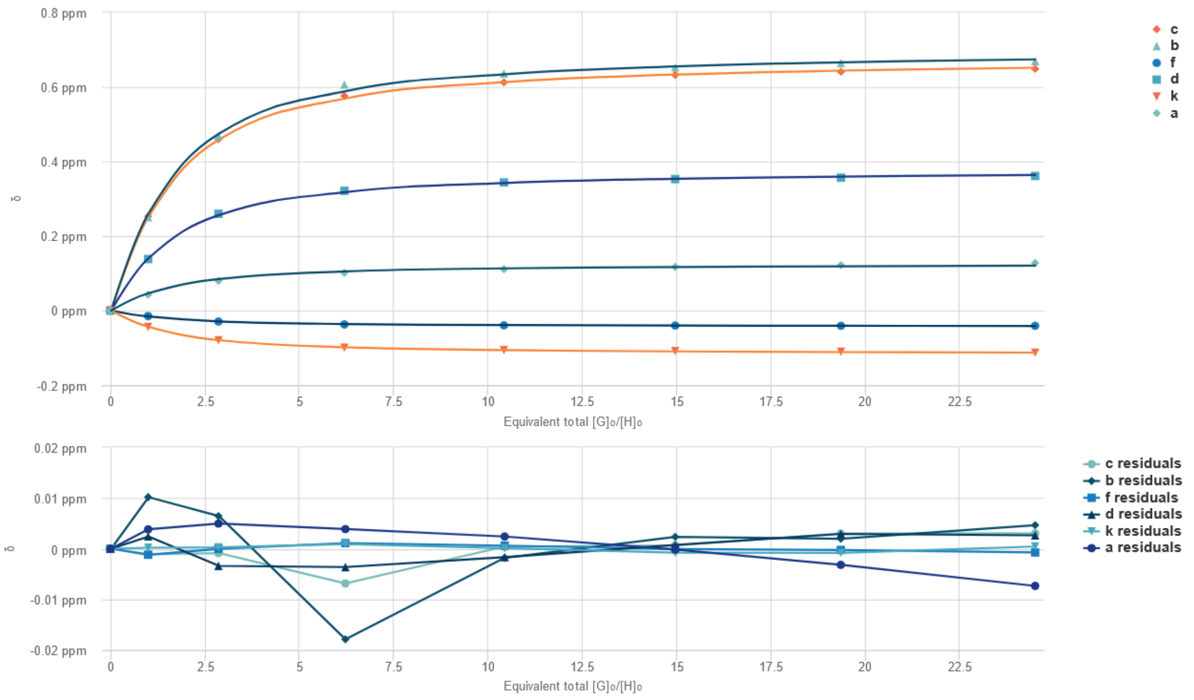

Figure S38. Top). ^{1}H NMR spectra (500 MHz, DMSO-*d*[6]) for receptor **7** (92 µM) titrated with a combined solution of tetrabutylammonium acetate (4.50 mM) and receptor **7** (92 µM). The equivalents and concentrations of guest added are listed in the graph. Bottom). Global fitting of the binding isotherms (protons a, b, c, d, f and k) from Bindfit to a 1:1 model K_a = 10626 M^{-1} (± 2.6 %). Full fitted data is available online at: http://app.supramolecular.org/bindfit/view/fa2205d2-2c4e-45d8-8cd7-3f1c4e3d4e9d

Propionate

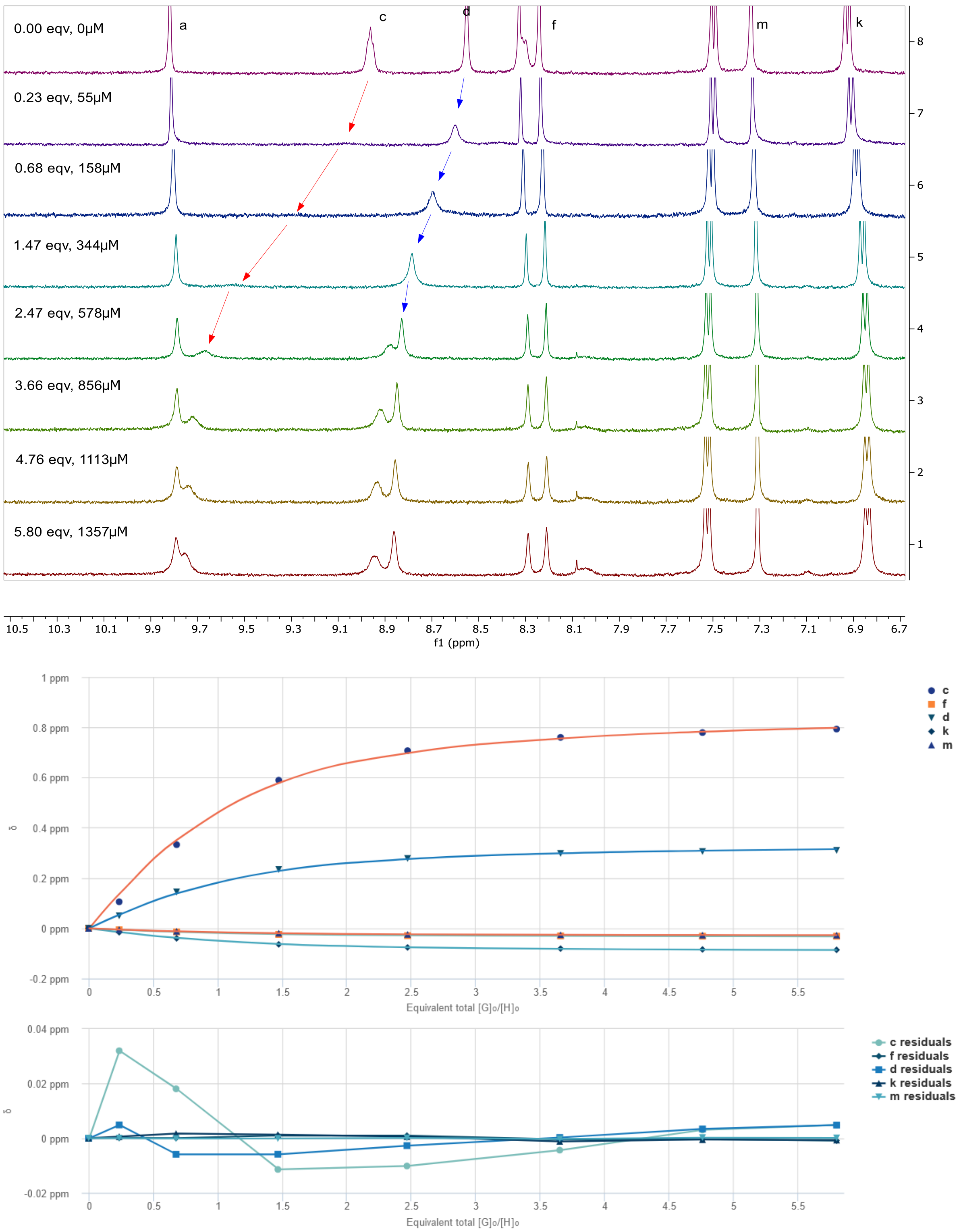

Figure S39. Top). ^{1}H NMR spectra (500 MHz, DMSO-d^6) for receptor **7** (234 µM) titrated with a combined solution of tetrabutylammonium propionate (2.80 mM) and receptor **7** (234 µM). The equivalents and concentrations of guest added are listed in the graph. Bottom). Global fitting of the binding isotherms (protons c, d, m, f and k) from Bindfit to a 1:1 model K_a = 10895 M^{-1} (± 5.3 %). Full fitted data is available online at: http://app.supramolecular.org/bindfit/view/06bc72aa-65ab-4074-a3a5-e75bf541a769

n-Butyrate

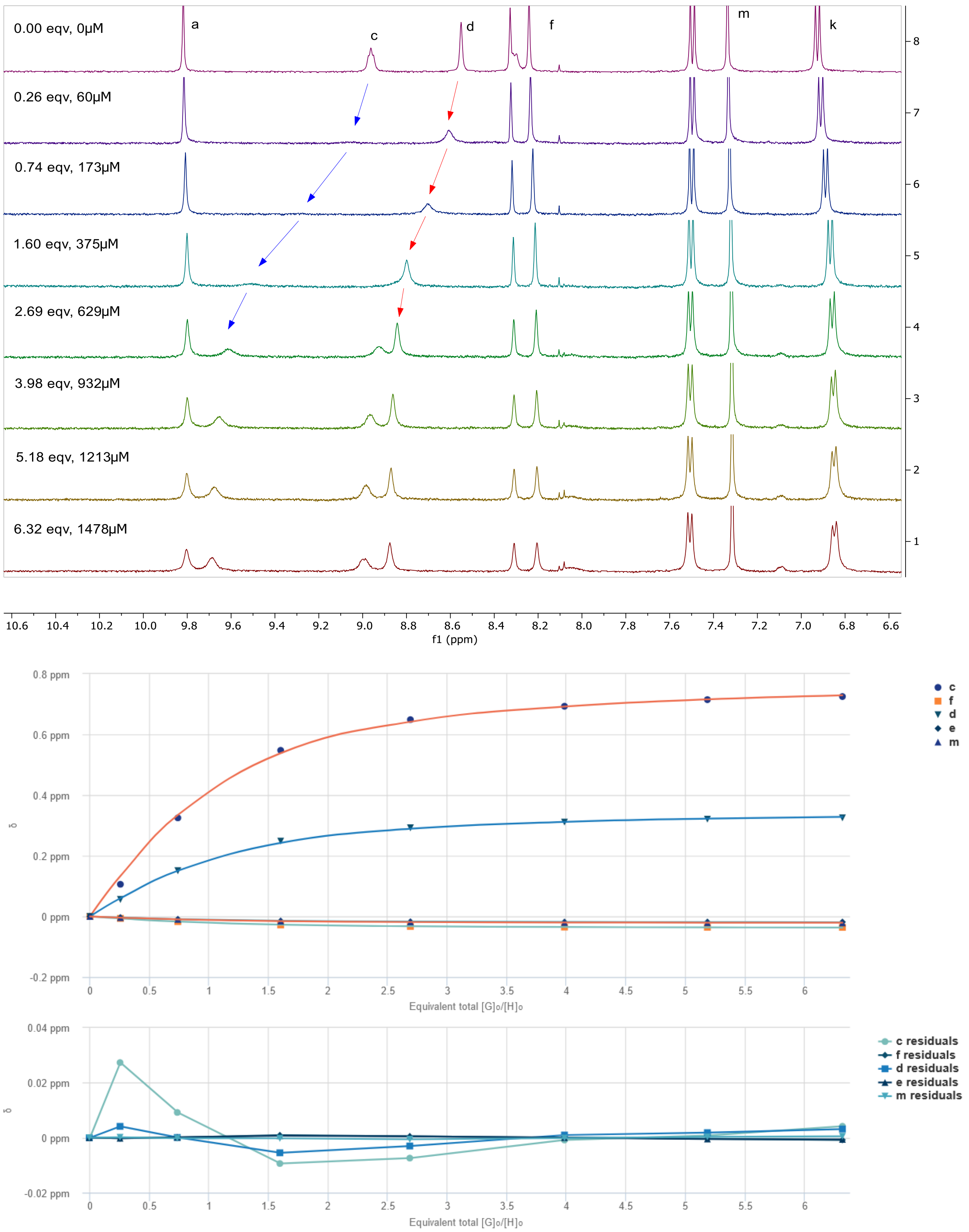

Figure S40. Top). ^{1}H NMR spectra (500 MHz, DMSO-d^6) for receptor **7** (234 µM) titrated with a combined solution of tetrabutylammonium *n*-butyrate (3.05 mM) and receptor **7** (234 µM). The equivalents and concentrations of guest added are listed in the graph. Bottom). Global fitting of the binding isotherms (protons c, d, m, f and e) from Bindfit to a 1:1 model K_a = 10135 M^{-1} (± 4.4 %). Full fitted data is available online at: http://app.supramolecular.org/bindfit/view/e304b436-4b00-4a7f-be3f-3a60b560611a

Benzoate

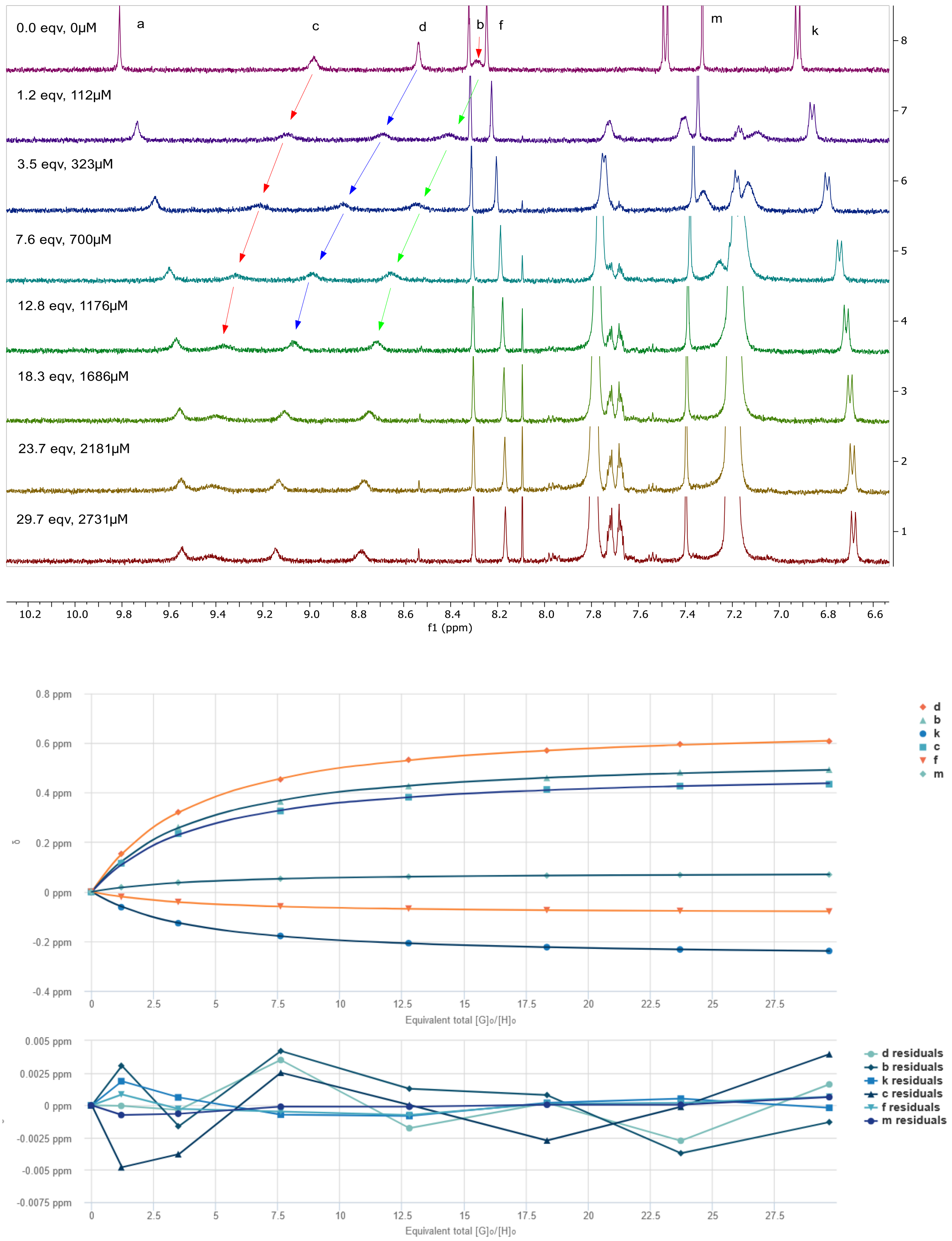

Figure S41. Top). ^{1}H NMR spectra (500 MHz, DMSO-d^6) for receptor (92 μM) titrated with a combined solution of tetrabutylammonium benzoate (5.70 mM) and receptor **7** (92 μM). The equivalents and concentrations of guest added are listed in the graph. Bottom). Global fitting of the binding isotherms (protons b, c, d, m, f and k) from Bindfit to a 1:1 model K_a = 3188 M^{-1} (± 0.9 %). Full fitted data is available online at: http://app.supramolecular.org/bindfit/view/b1da9fdd-34ce-430a-ae5e-061d64e1d94a

2.1.2 Receptor 8 in DMSO-d^6

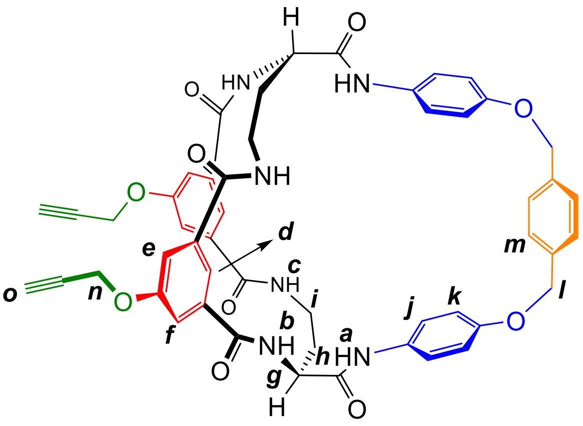

Acetate

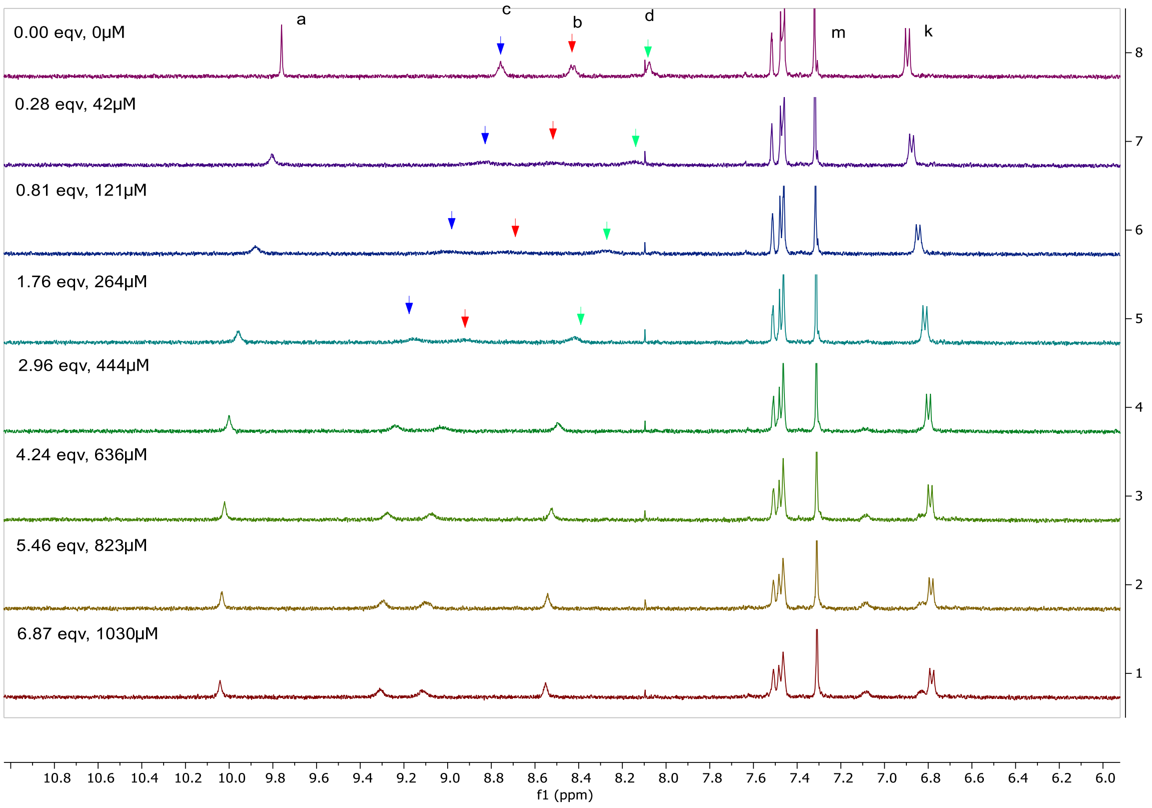

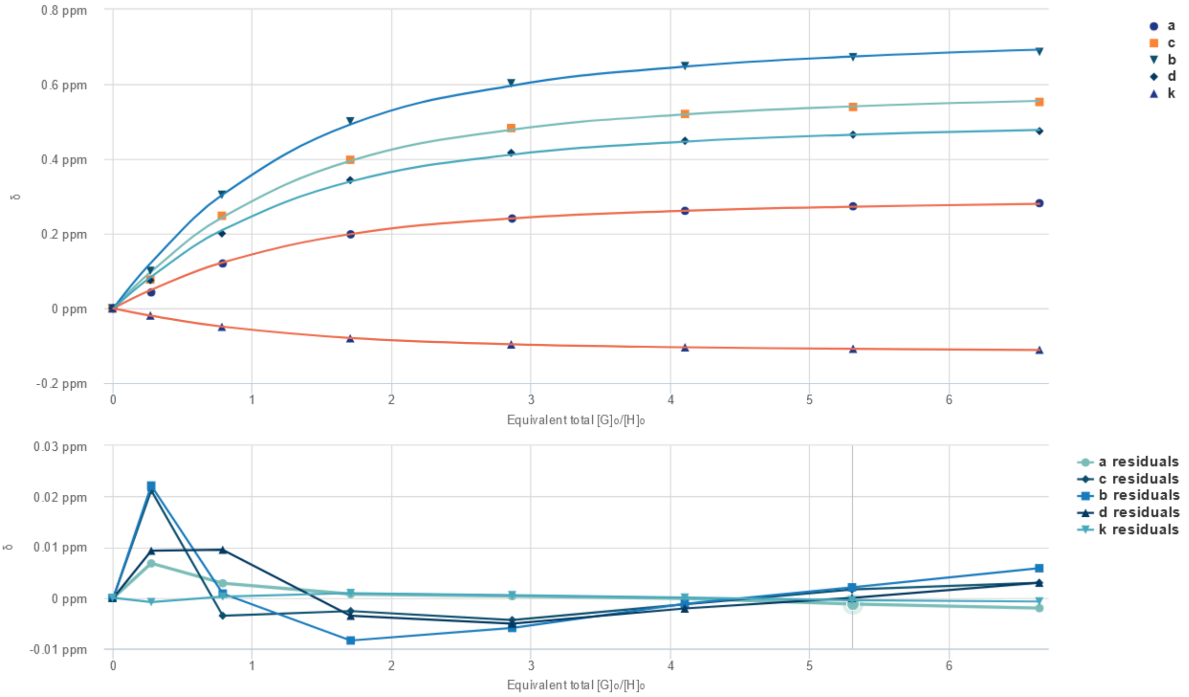

Figure S42. Top). ^{1}H NMR spectra (500 MHz, DMSO-*d*[6]) for receptor **8** (0.15 mM) titrated with a combined solution of tetrabutylammonium acetate (2.08 mM) and receptor **8** (0.15 mM). The equivalents and concentrations of guest added are listed in the graph. Bottom). Global fitting of the binding isotherms (protons a, b, c, d and k) from Bindfit to a 1:1 model K_a = 11511 M^{-1} (± 3.4 %). Full fitted data is available online at: http://app.supramolecular.org/bindfit/view/fb9d8e84-b25c-4a38-8a5c-572ef289e519

Propionate

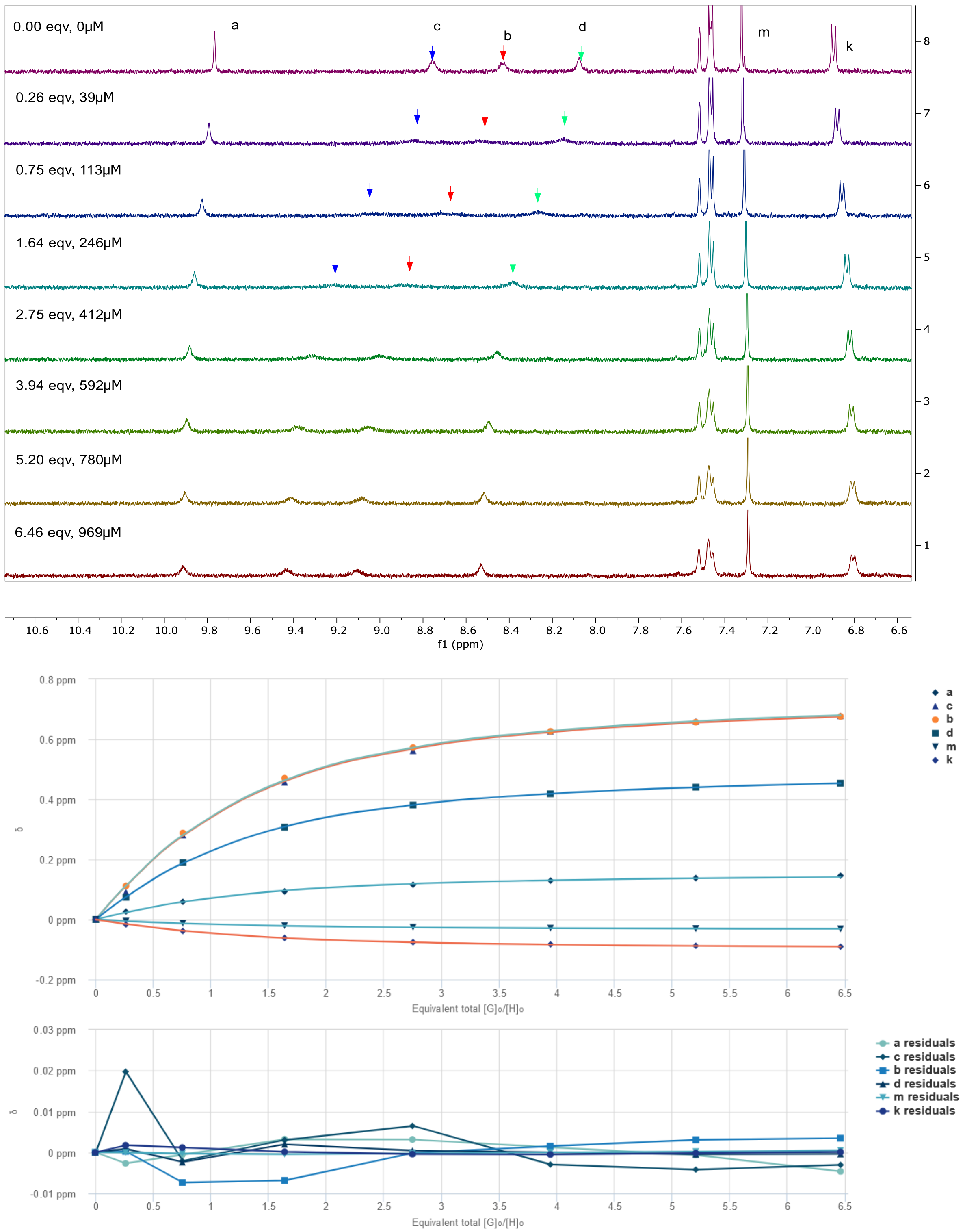

Figure S43. Top). ^{1}H NMR spectra (500 MHz, DMSO-*d*^{6}) for receptor **8** (0.15 mM) titrated with a combined solution of tetrabutylammonium propionate (2.00 mM) and receptor **8** (0.15 mM). The equivalents and concentrations of guest added are listed in the graph. Bottom). Global fitting of the binding isotherms (protons a, b, c, d, m and k) from Bindfit to a 1:1 model K_a = 10136 M^{-1} (± 1.9 %). Full fitted data is available online at: http://app.supramolecular.org/bindfit/view/42a9e11b-05b8-4d40-96d0-725ab4be46c8

n-Butyrate

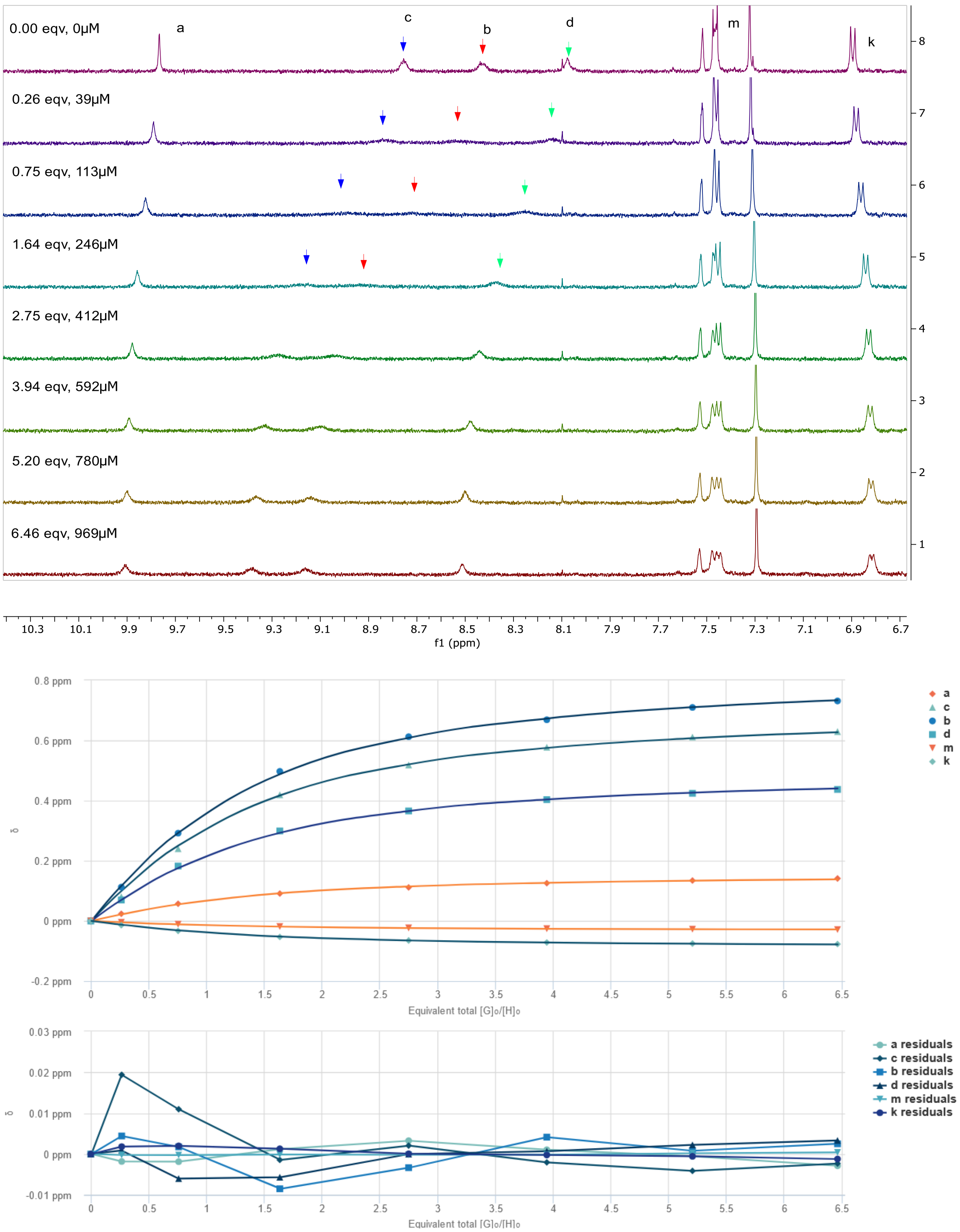

Figure S44. Top). ^{1}H NMR spectra (500 MHz, DMSO-*d*6) for receptor **8** (0.15 mM) titrated with a combined solution of tetrabutylammonium *n*-butyrate (2.00 mM) and receptor **8** (0.15 mM). The equivalents and concentrations of guest added are listed in the graph. Bottom). Global fitting of the binding isotherms (protons a, b, c, d, m and k) from Bindfit to a 1:1 model K_a = 9114 M^{-1} (± 1.9 %). Full fitted data is available online at: http://app.supramolecular.org/bindfit/view/79552418-fde3-400e-bda9-06a83d4fd626

Benzoate

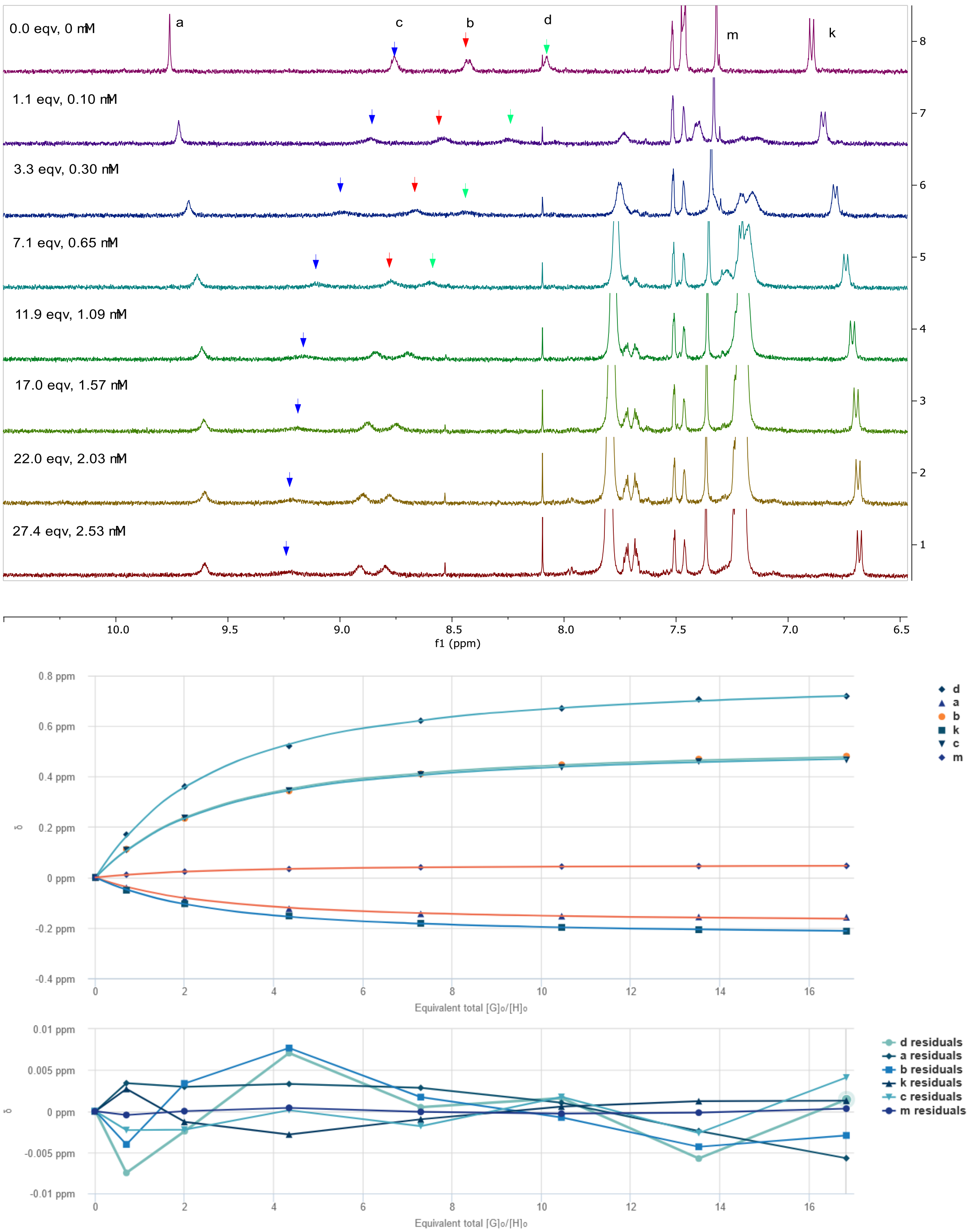

Figure S45. Top). ^{1}H NMR spectra (500 MHz, DMSO-*d*[6]) for receptor **8** (0.15 mM) titrated with a combined solution of tetrabutylammonium benzoate (15.00 mM) and receptor **8** (0.15 mM). The equivalents and concentrations of guest added are listed in the graph. Bottom). Global fitting of the binding isotherms (protons a, b, c, d, m and k) from Bindfit to a 1:1 model K_a = 3413 M^{-1} (± 1.3 %). Full fitted data is available online at: http://app.supramolecular.org/bindfit/view/a21684a9-5498-48ef-98ce-ef6942c27d65

2.1.3 Receptor 10 in DMSO-d^6

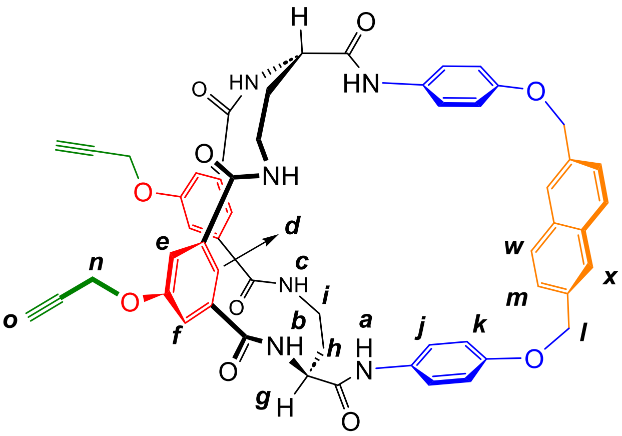

Acetate

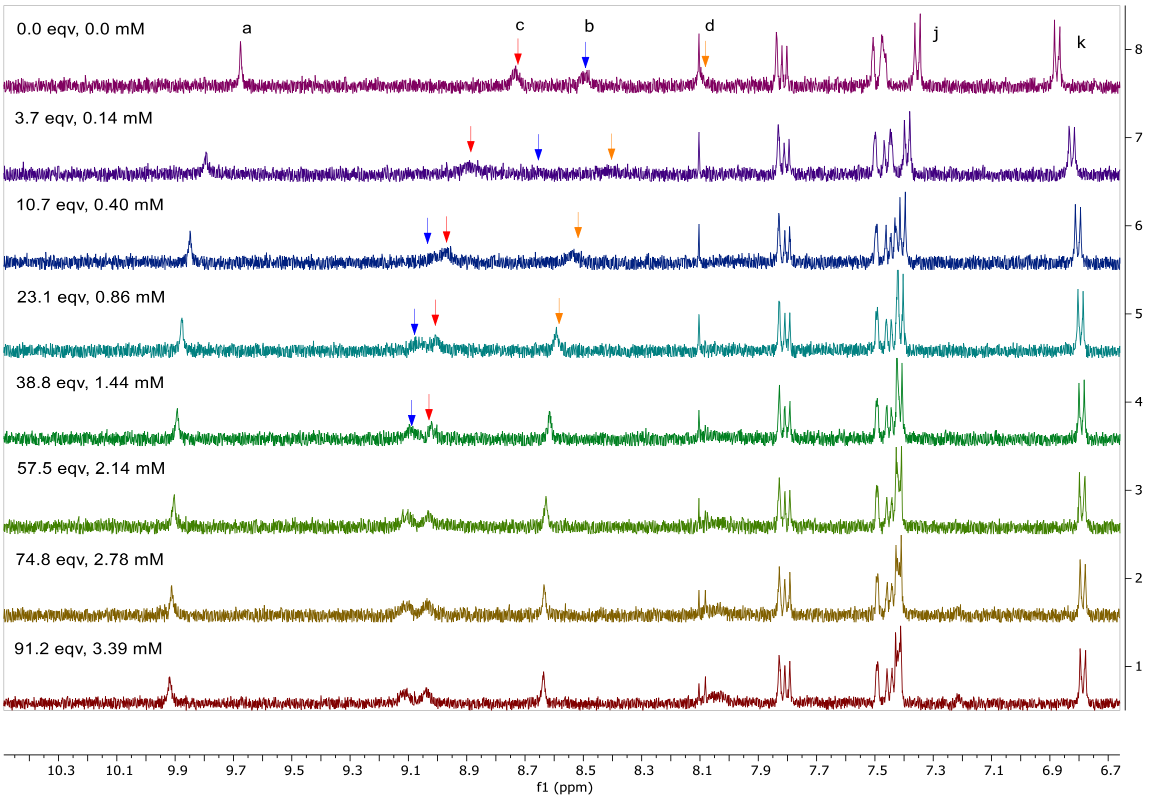

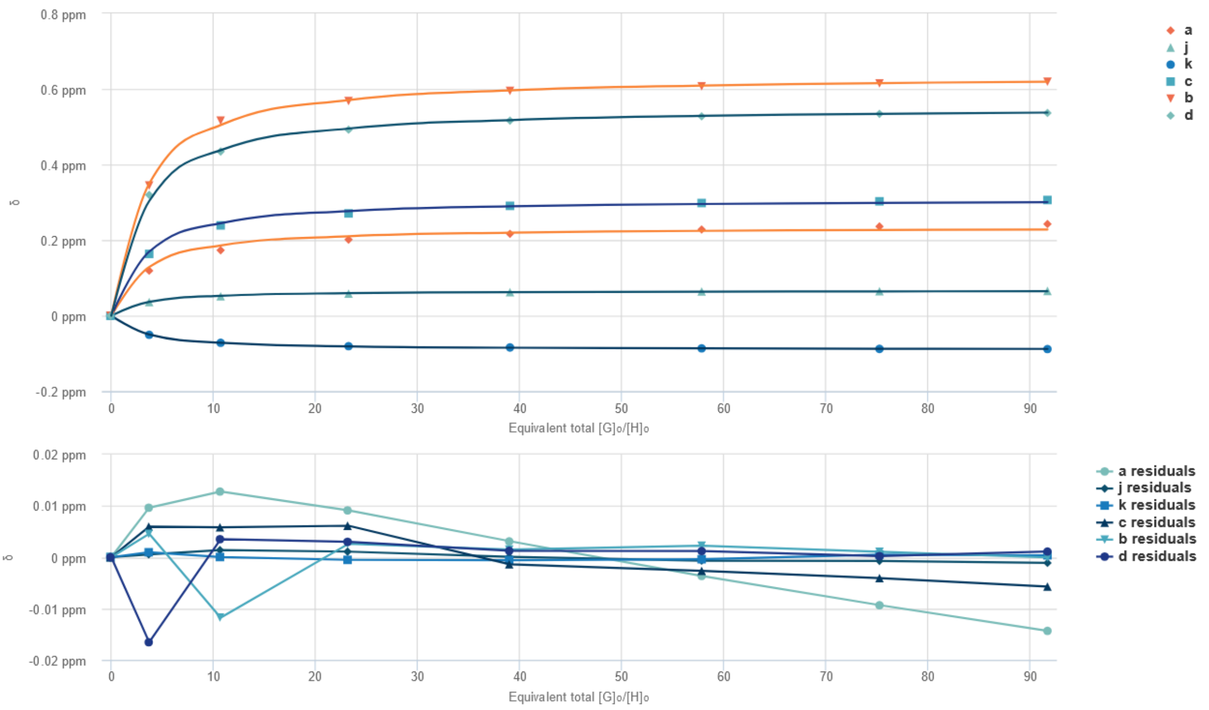

Figure S46. Top). ^{1}H NMR spectra (500 MHz, DMSO-d^6) for receptor **10** (37 µM) titrated with a combined solution of tetrabutylammonium acetate (7.00 mM) and receptor **10** (37 µM). The equivalents and concentrations of guest added are listed in the graph. Bottom). Global fitting of the binding isotherms (protons a, b, c, d, m and k) from Bindfit to a 1:1 model K_a = 10382 M^{-1} (± 4.0 %). Full fitted data is available online at: http://app.supramolecular.org/bindfit/view/2a0947fa-d49b-42f9-ba60-f83e840924c1

Benzoate

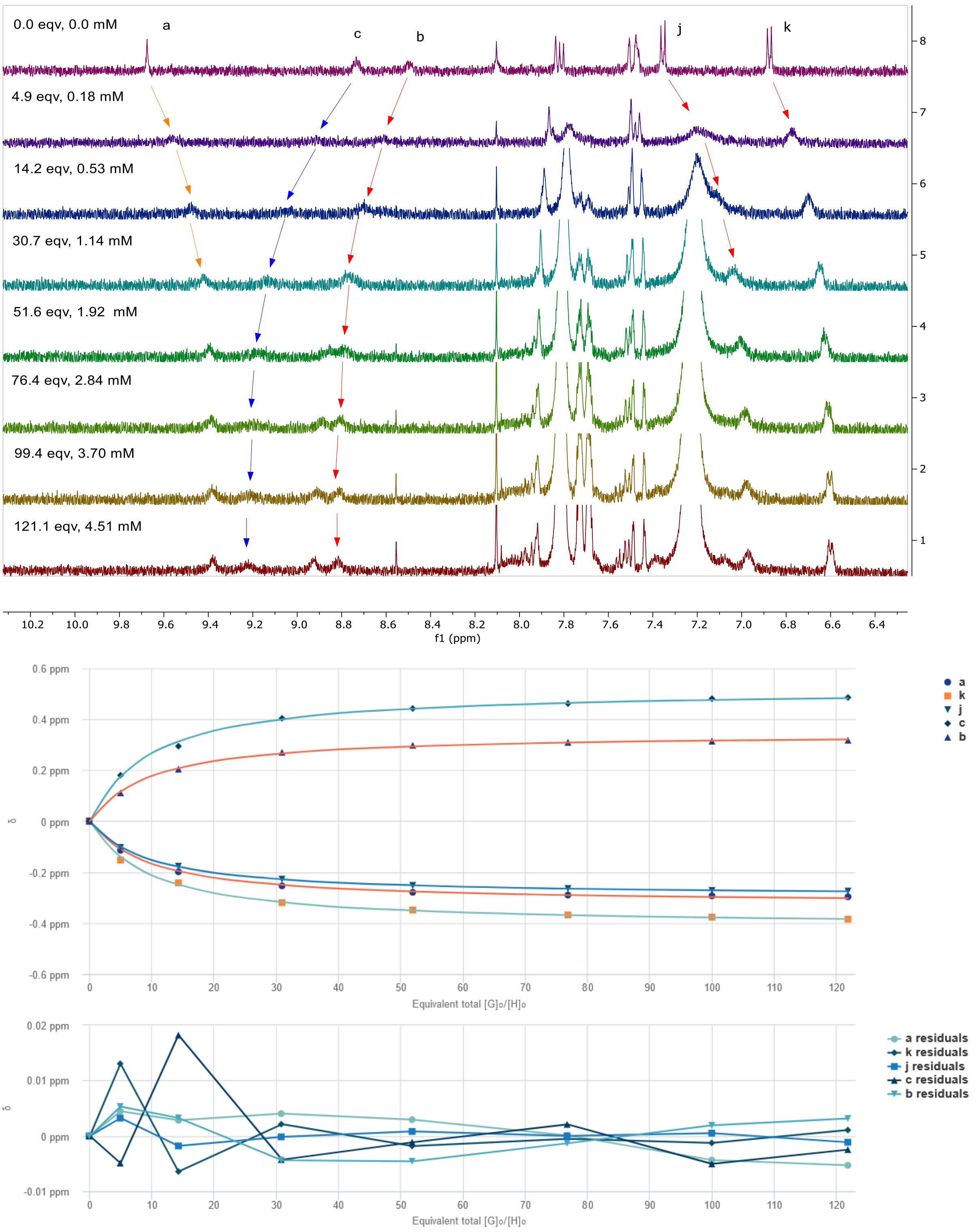

Figure S47. Top). ^{1}H NMR spectra (500 MHz, DMSO-d^6) for receptor **10** (37 μM) titrated with a combined solution of tetrabutylammonium benzoate (9.3 mM) and receptor (37 μM). The equivalents and concentrations of guest added are listed in the graph. Bottom). Global fitting of the binding isotherms (protons a, b, c, j and k) from Bindfit to a 1:1 model K_a = 3005 M^{-1} (± 2.9 %). Full fitted data is available online at: http://app.supramolecular.org/bindfit/view/0b305d9b-a2f9-40d3-aa1f-e60d2cbb3e92

2.1.4 Receptor 12 in DMSO-d^6

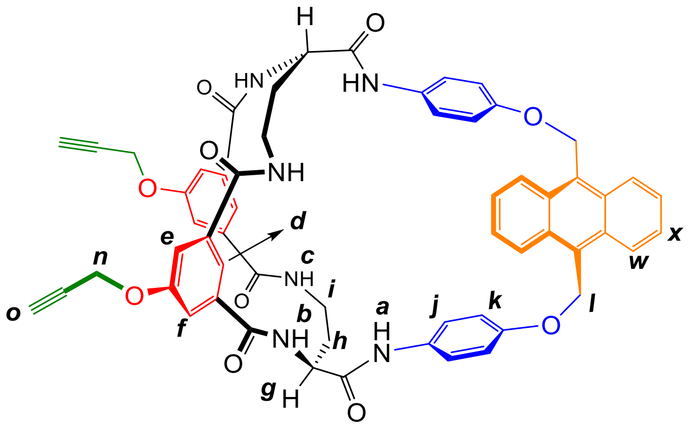

Acetate

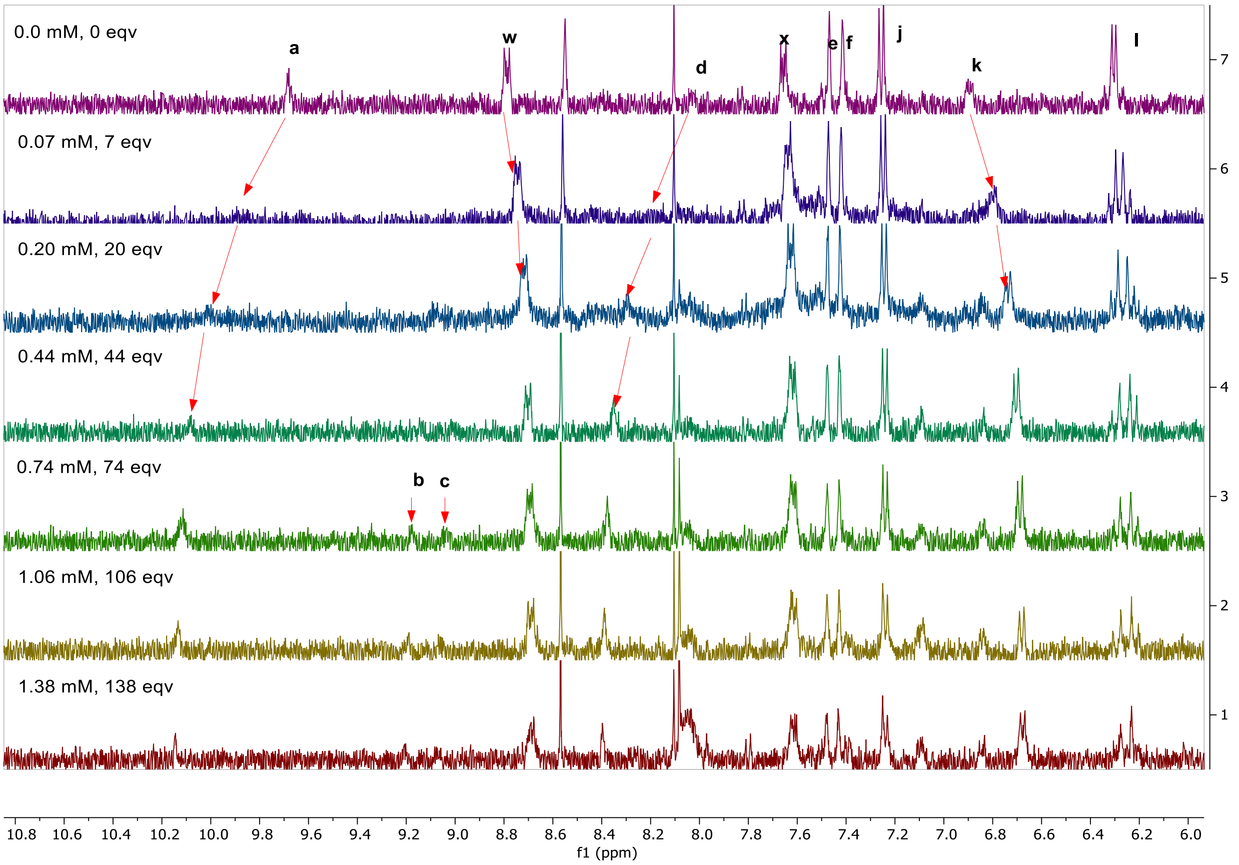

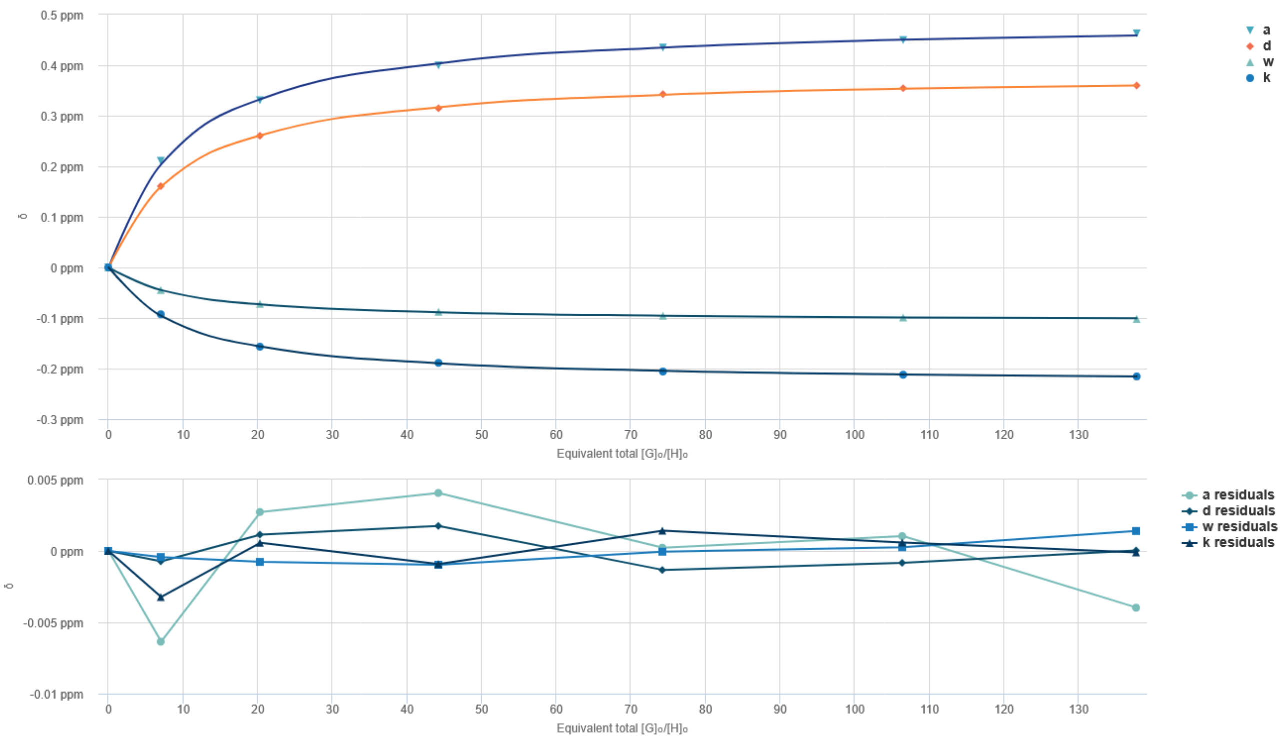

Figure S48. Top). ^{1}H NMR spectra (500 MHz, DMSO-*d*[6]) for receptor **12** (10 µM) titrated with a combined solution of tetrabutylammonium acetate (3.6 mM) and receptor **12** (10 µM). The equivalents and concentrations of guest added are listed in the graph. Bottom). Global fitting of the binding isotherms (protons a, d, w and k) from Bindfit to a 1:1 model K_a = 10762 M^{-1} (± 1.8 %). Full fitted data is available online at: http://app.supramolecular.org/bindfit/view/421fd76a-323b-469f-b43b-5f047c9ffc76

Benzoate

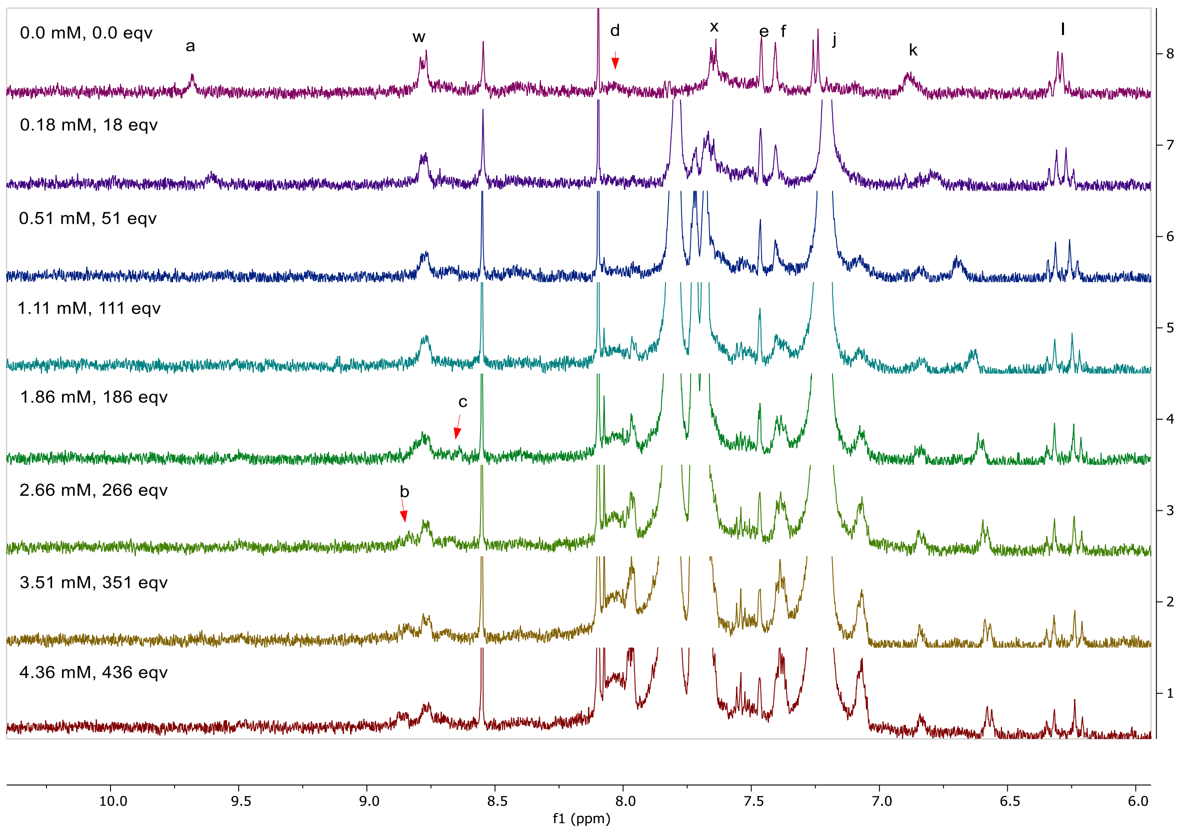

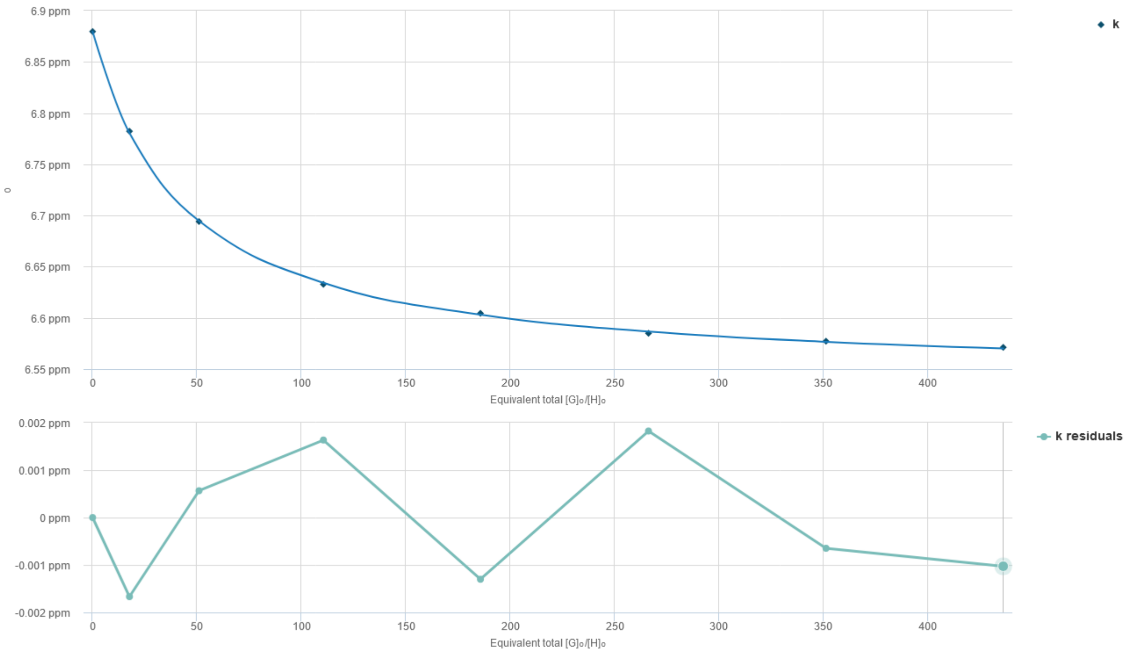

Figure S49. Top). ^{1}H NMR spectra (500 MHz, DMSO-d^{6}) for receptor **12** (10 µM) titrated with a combined solution of tetrabutylammonium benzoate (9 mM) and receptor **12** (10 µM). The equivalents and concentrations of guest added are listed in the graph. Bottom). Fitting of the binding isotherm (proton k) from Bindfit to a 1:1 model K_a = 2374 M^{-1} (± 2.6 %). Full fitted data is available online at: http://app.supramolecular.org/bindfit/view/5f0bdc25-91bc-4d62-9e43-6bf4ac4397bb

2.1.7 Receptor 9 in H_2O/D_2O

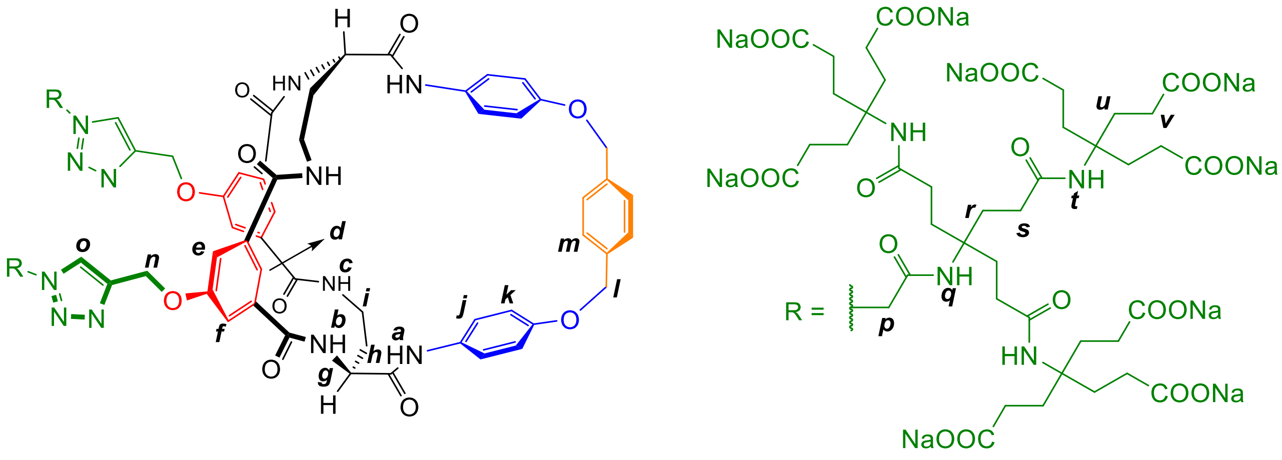

Formate

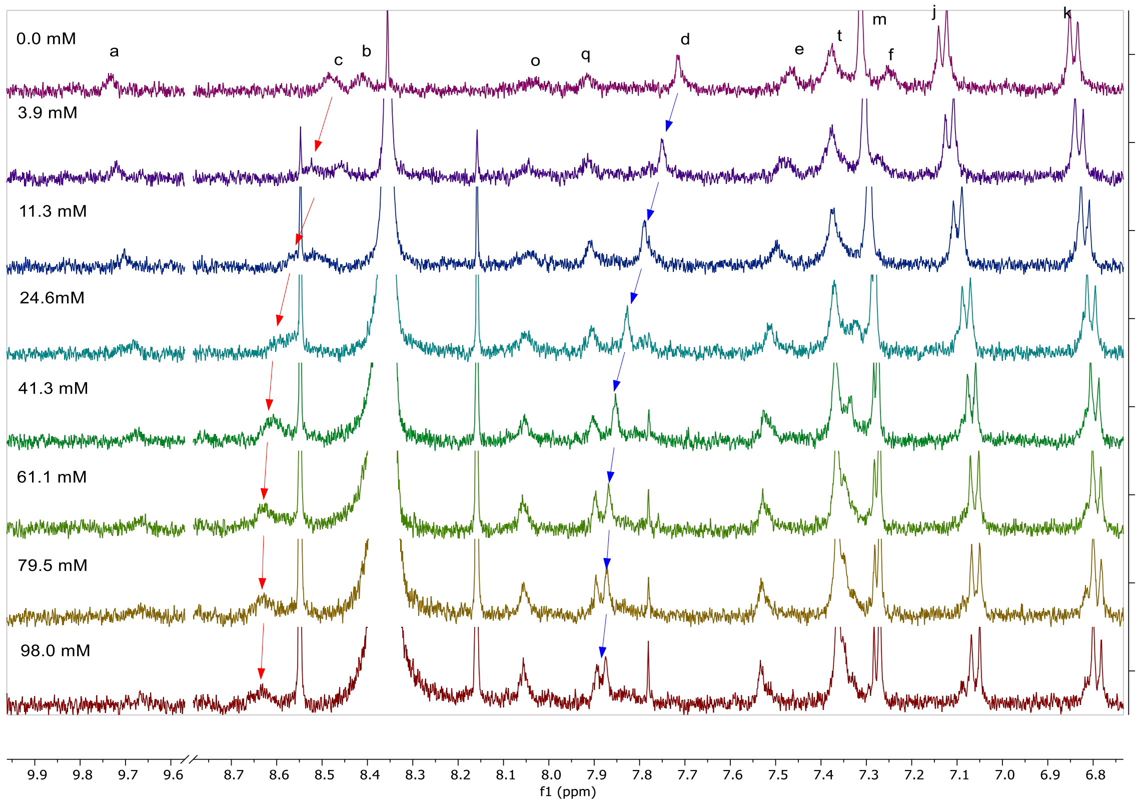

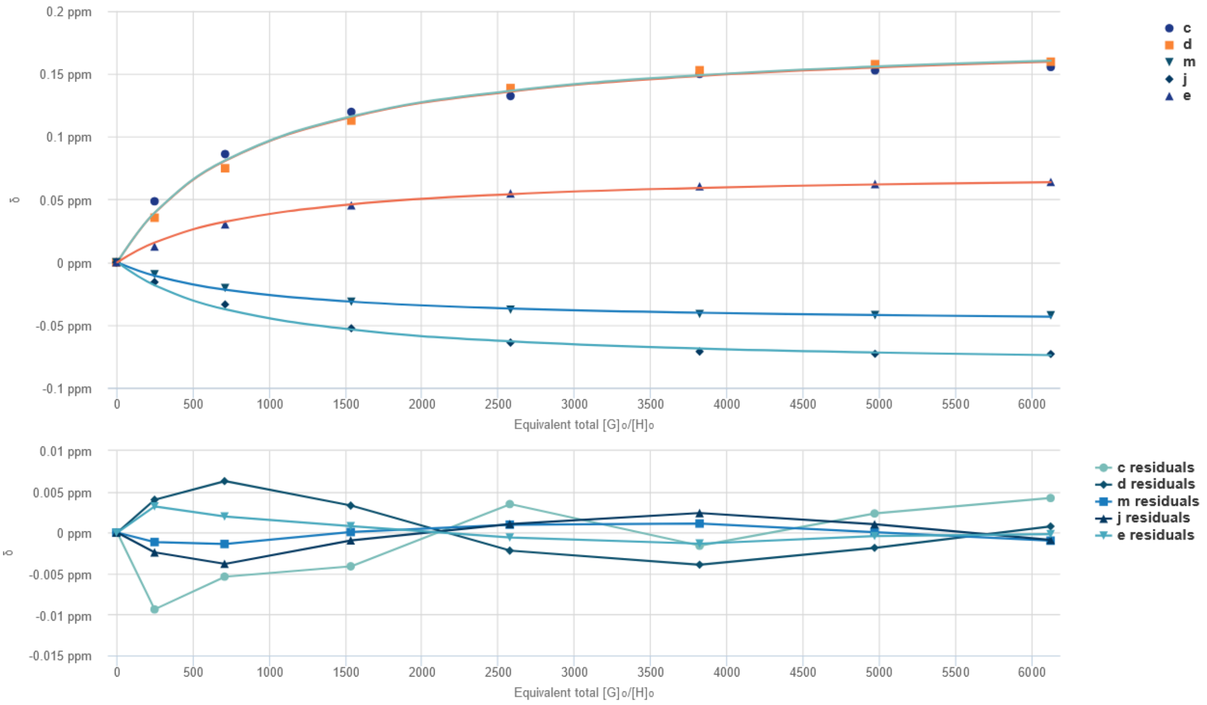

Figure S50. Top). ^{1}H NMR spectra (500 MHz, 1:9 D_2O/H_2O) for receptor **9** (16 µM) titrated with a combined solution of sodium formate (200 mM) and receptor **9** (16 µM). The concentrations of the guest added are listed in the graph. Bottom). Global fitting of the binding isotherms (protons c, d m, j and e) from Bindfit to a 1:1 model K_a = 69.8 M^{-1} (± 4.5 %). Full fitted data is available online at:
http://app.supramolecular.org/bindfit/view/c6e9ba64-9b46-45a1-a7f0-df22b7db273d

Acetate

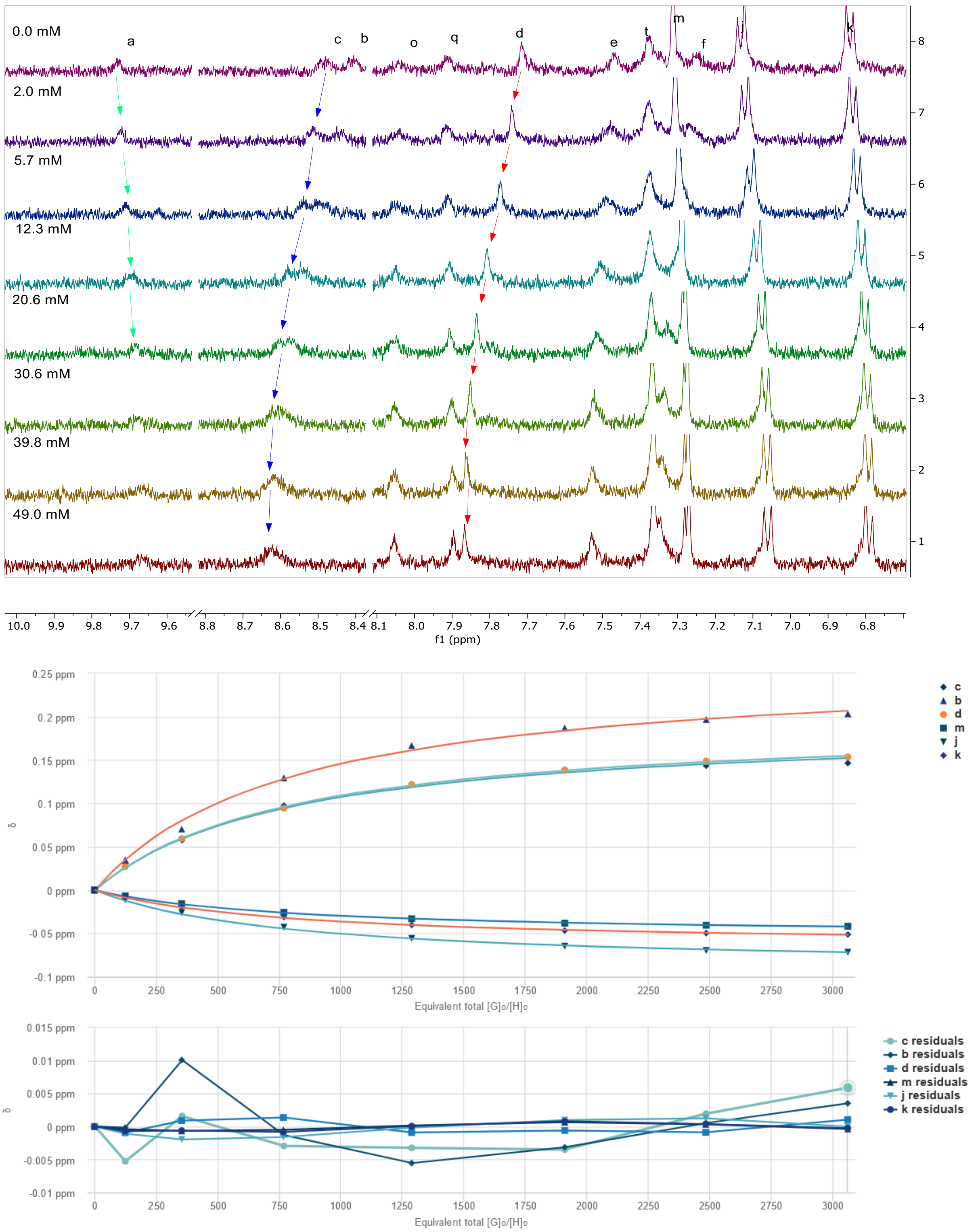

Figure S51. Top). ^{1}H NMR spectra (500 MHz, 1:9 D_2O/H_2O) for receptor **9** (16 µM) titrated with a combined solution of sodium acetate (100 mM) and receptor **9** (16 µM). The concentrations of the guest added are listed in the graph. Bottom). Global fitting of the binding isotherms (protons c, b, d m, j and k) from Bindfit to a 1:1 model K_a = 79.2 M^{-1} (± 2.5 %). Full fitted data is available online at: http://app.supramolecular.org/bindfit/view/f393de08-51d8-4889-8ec2-64e2b2ca4f24

Propionate

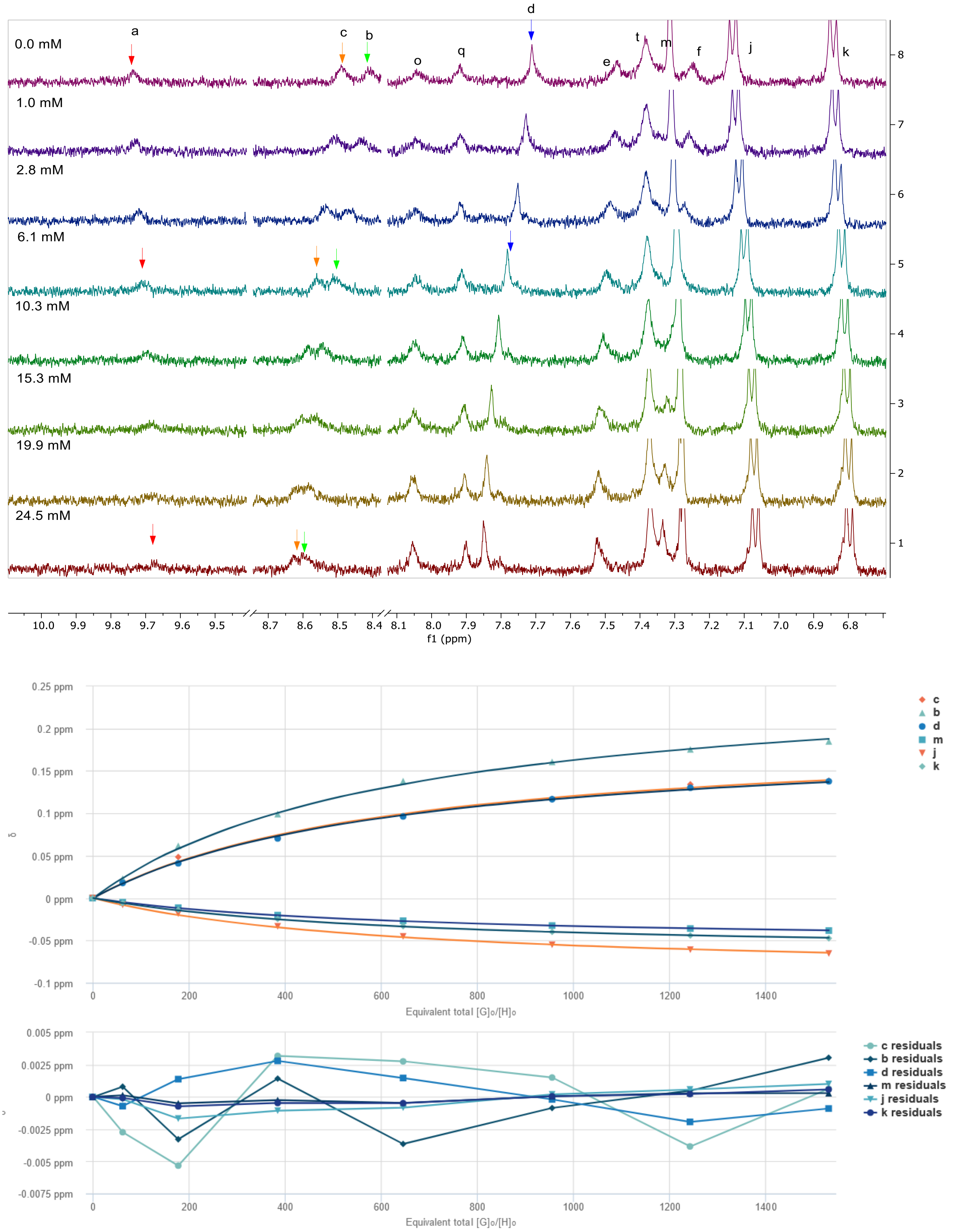

Figure S52. Top). ^{1}H NMR spectra (500 MHz, 1:9 D_2O/H_2O) for receptor **9** (16 µM) titrated with a combined solution of sodium propionate (50 mM) and receptor **9** (16 µM). The concentrations of the guest added are listed in the graph. Bottom). Global fitting of the binding isotherms (protons b, c, d m, j and k) from Bindfit to a 1:1 model K_a = 99.9 M^{-1} (± 1.7 %). Full fitted data is available online at:

http://app.supramolecular.org/bindfit/view/c6ce52d7-3e11-4de5-8f8a-7caf0c688f92

n-Butyrate

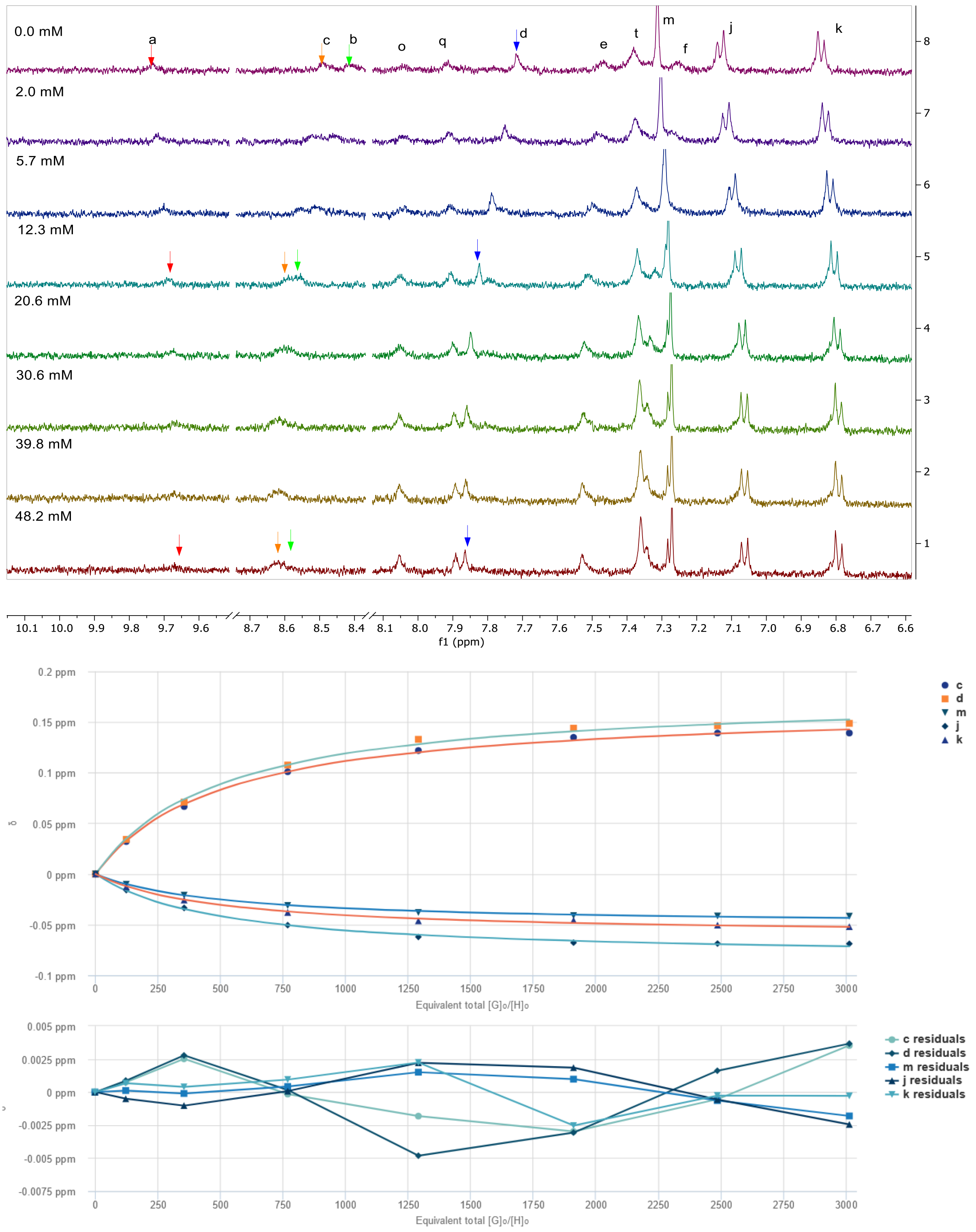

Figure S53. Top). ^{1}H NMR spectra (500 MHz, 1:9 D_2O/H_2O) for receptor **9** (16 µM) titrated with a combined solution of sodium *n*-butyrate (100 mM) and receptor **9** (16 µM). The concentrations of the guest added are listed in the graph. Bottom). Global fitting of the binding isotherms (protons c, d m, j and k) from Bindfit to a 1:1 model K_a = 125.6 M^{-1} (± 3.0 %). Full fitted data is available online at: http://app.supramolecular.org/bindfit/view/95493670-b673-4c75-9811-5eaf5bc376ea

iso-Butyrate

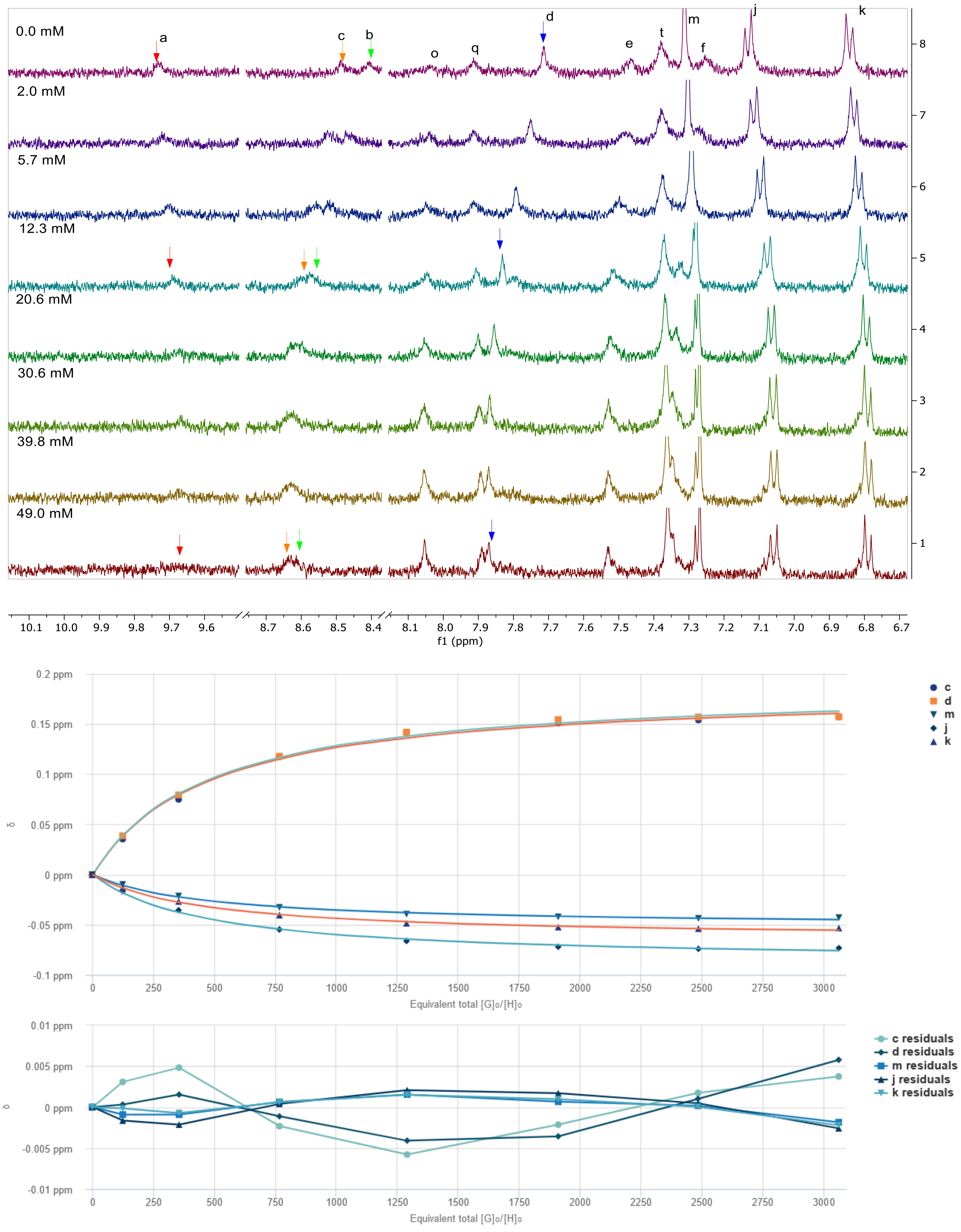

Figure S54. Top). ^{1}H NMR spectra (500 MHz, 1:9 D_2O/H_2O) for receptor **9** (16 µM) titrated with a combined solution of sodium *iso*-butyrate (100 mM) and receptor **9** (16 µM). The concentrations of the guest added are listed in the graph. Bottom). Global fitting of the binding isotherms (protons c, d m, j and k) from Bindfit to a 1:1 model K_a = 133.9 M^{-1} (± 3.6 %). Full fitted data is available online at: http://app.supramolecular.org/bindfit/view/1a739b3b-0bee-426f-b9c4-3bad3c55f959

Pivalate

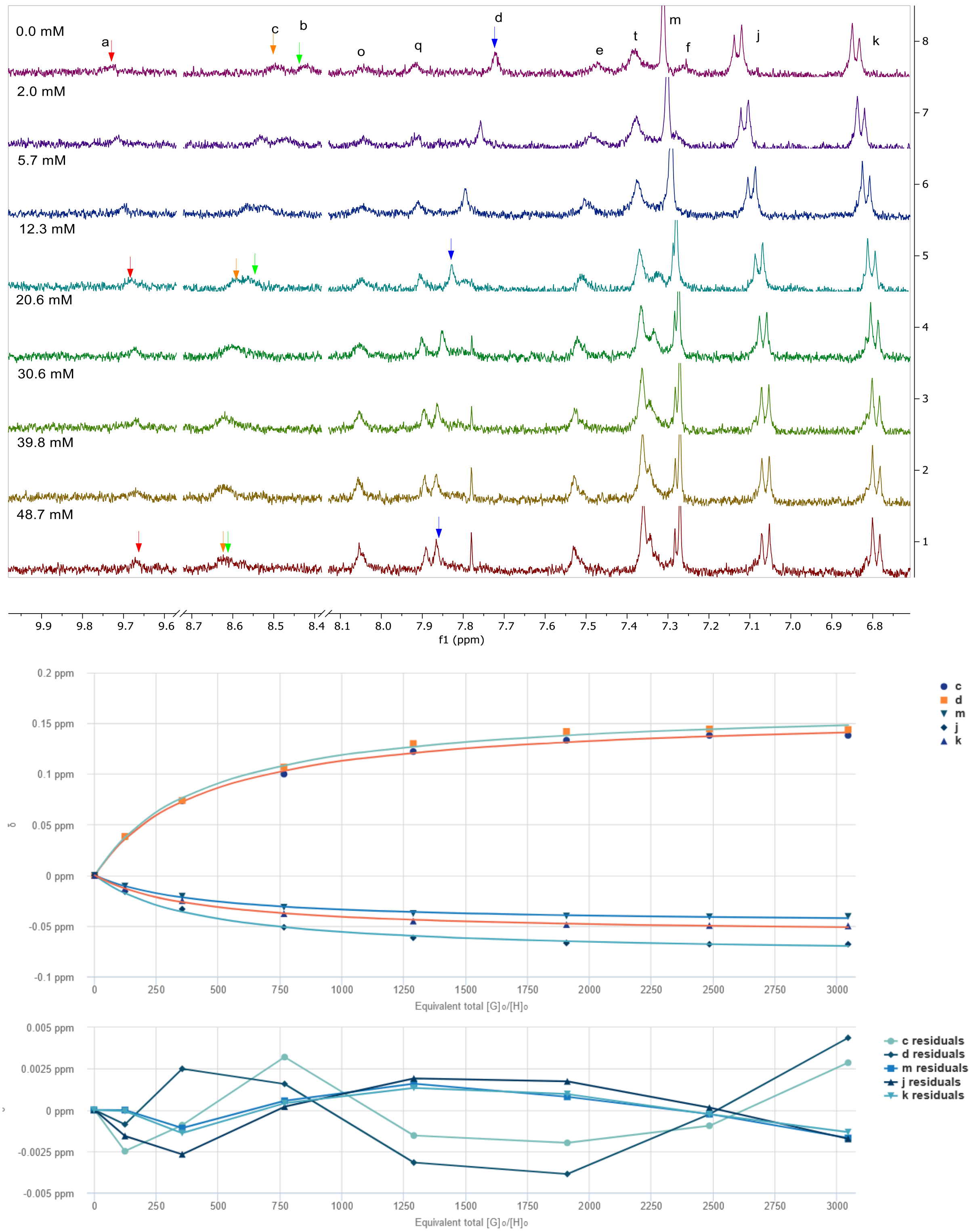

Figure S55. Top). ^{1}H NMR spectra (500 MHz, 1:9 D_2O/H_2O) for receptor **9** (16 µM) titrated with a combined solution of sodium pivalate (100 mM) and receptor **9** (16 µM). The concentrations of the guest added are listed in the graph. Bottom). Global fitting of the binding isotherms (protons c, d m, j and k) from Bindfit to a 1:1 model K_a = 145.7 M^{-1} (± 3.1 %). Full fitted data is available online at: http://app.supramolecular.org/bindfit/view/ecbce43c-6c3f-49a0-bfd3-1b8214571e0d

Benzoate-d5

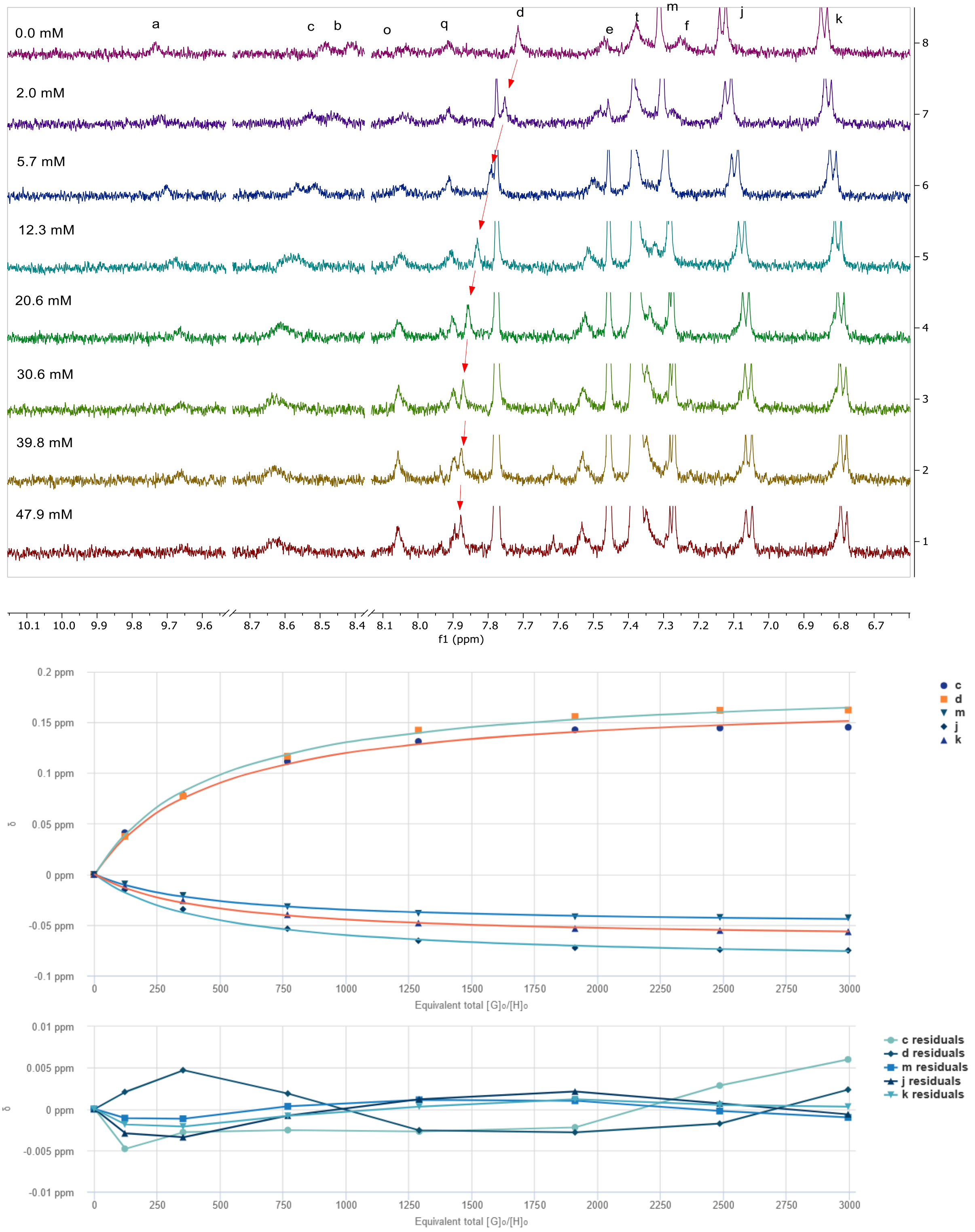

Figure S56. Top). ^{1}H NMR spectra (500 MHz, 1:9 D_2O/H_2O) for receptor **9** (16 µM) titrated with a combined solution of sodium benzoate-d5 (100 mM) and receptor **9** (16 µM). The concentrations of the guest added are listed in the graph. Bottom). Global fitting of the binding isotherms (protons c, d m, j and k) from Bindfit to a 1:1 model K_a = 134.7 M^{-1} (± 3.5 %). Full fitted data is available online at: http://app.supramolecular.org/bindfit/view/9c3e2d3d-f056-47e5-90bd-6025688ce249

D-Lactate

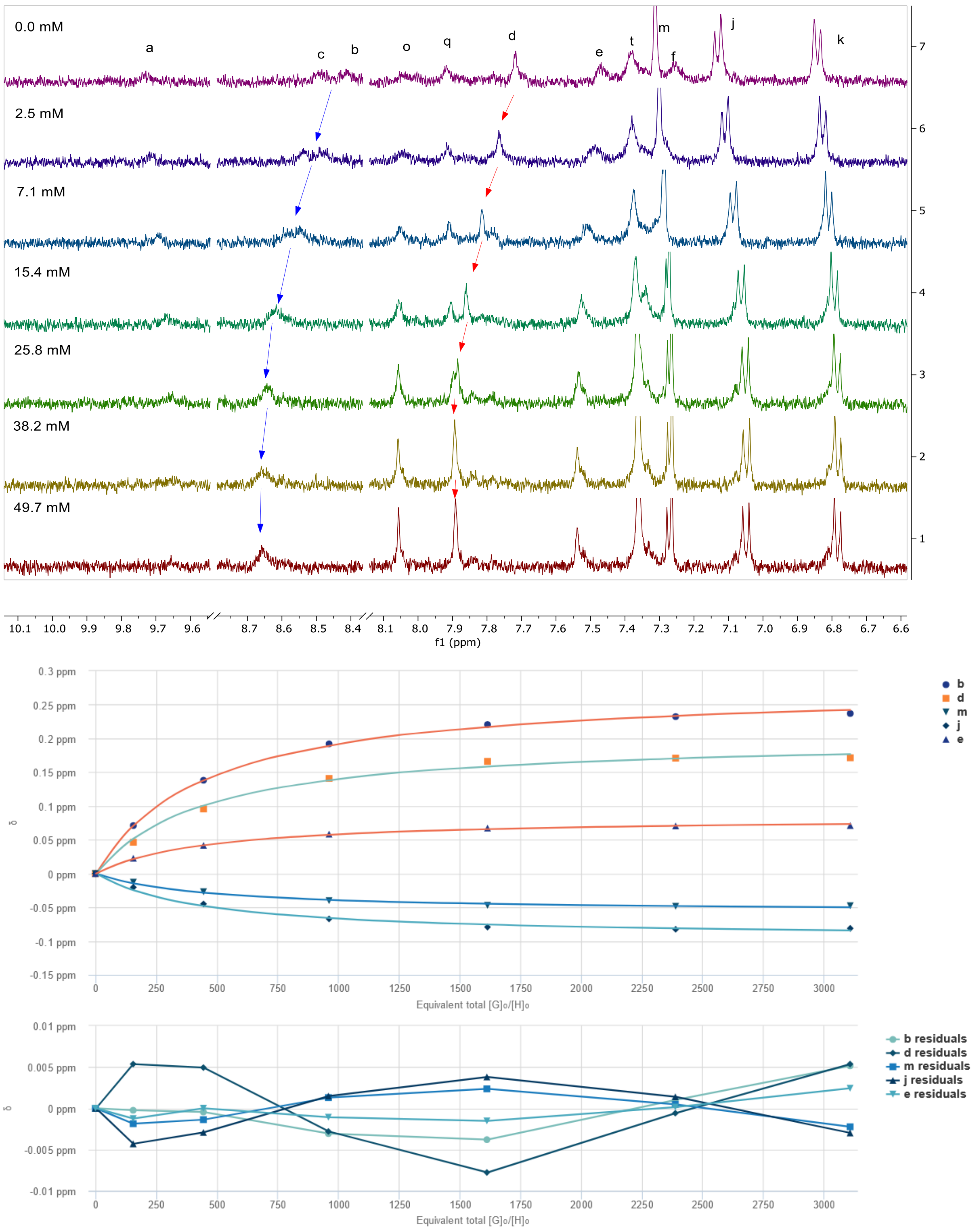

Figure S57. Top). ^{1}H NMR spectra (500 MHz, 1:9 D_2O/H_2O) for receptor **9** (16 µM) titrated with a combined solution of sodium *D*-lactate (125 mM) and receptor **9** (16 µM). The concentrations of the guest added are listed in the graph. Bottom). Global fitting of the binding isotherms (protons b, d m, j and e) from Bindfit to a 1:1 model K_a = 140.3 M^{-1} (± 3.9 %). Full fitted data is available online at:
http://app.supramolecular.org/bindfit/view/df0b7aef-ba7a-4968-84e8-a73cfd1cd489

L-Lactate

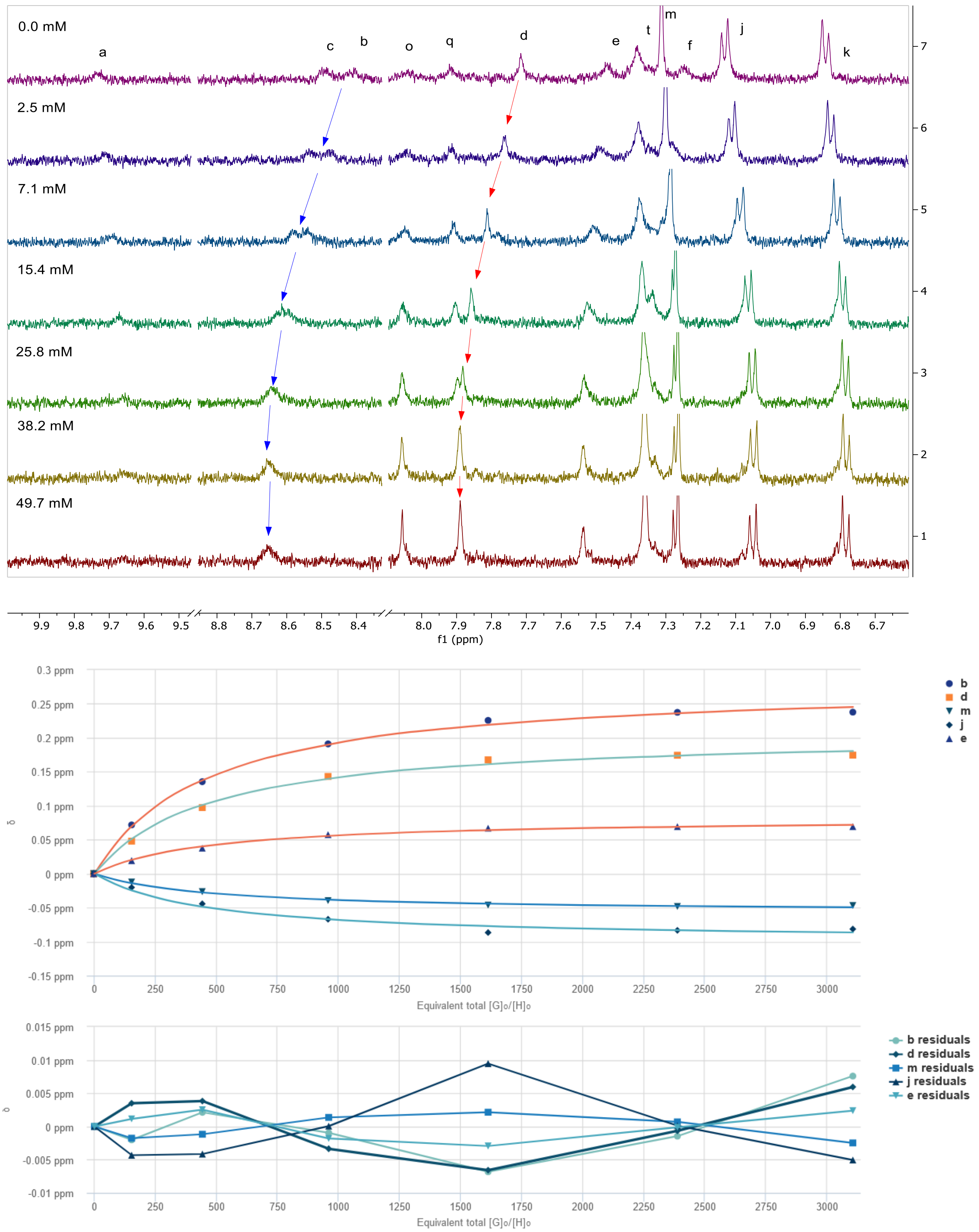

Figure S58. Top). ^{1}H NMR spectra (500 MHz, 1:9 D_2O/H_2O) for receptor **9** (16 µM) titrated with a combined solution of sodium *L*-lactate (125 mM) and receptor **9** (16 µM). The concentrations of the guest added are listed in the graph. Bottom). Global fitting of the binding isotherms (protons b, d m, j and k) from Bindfit to a 1:1 model K_a = 134.6 M^{-1} (± 4.6 %). Full fitted data is available online at: http://app.supramolecular.org/bindfit/view/5fbf313e-402d-4c43-874d-732c0aa85ca0

Chloride

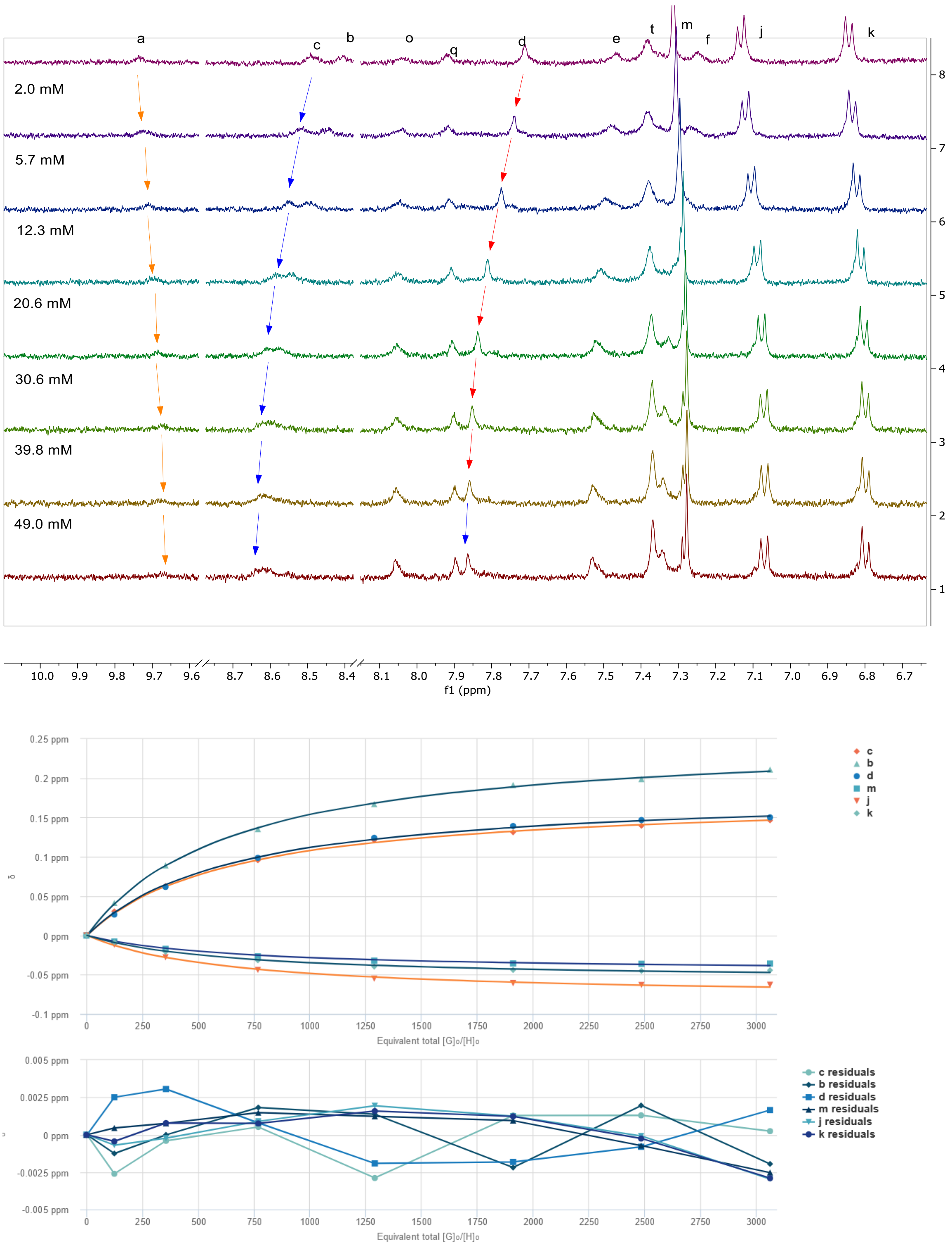

Figure S59. Top). ^{1}H NMR spectra (500 MHz, 1:9 D_2O/H_2O) for receptor **9** (16 µM) titrated with a combined solution of sodium chloride (100 mM) and receptor **9** (16 µM). The concentrations of the guest added are listed in the graph. Bottom). Global fitting of the binding isotherms (protons b, c, d m, j and k) from Bindfit to a 1:1 model K_a = 95.7 M^{-1} (± 1.7 %). Full fitted data is available online at:

http://app.supramolecular.org/bindfit/view/3ee5f977-7a55-458e-aed5-bc13d0d20464

Bromide

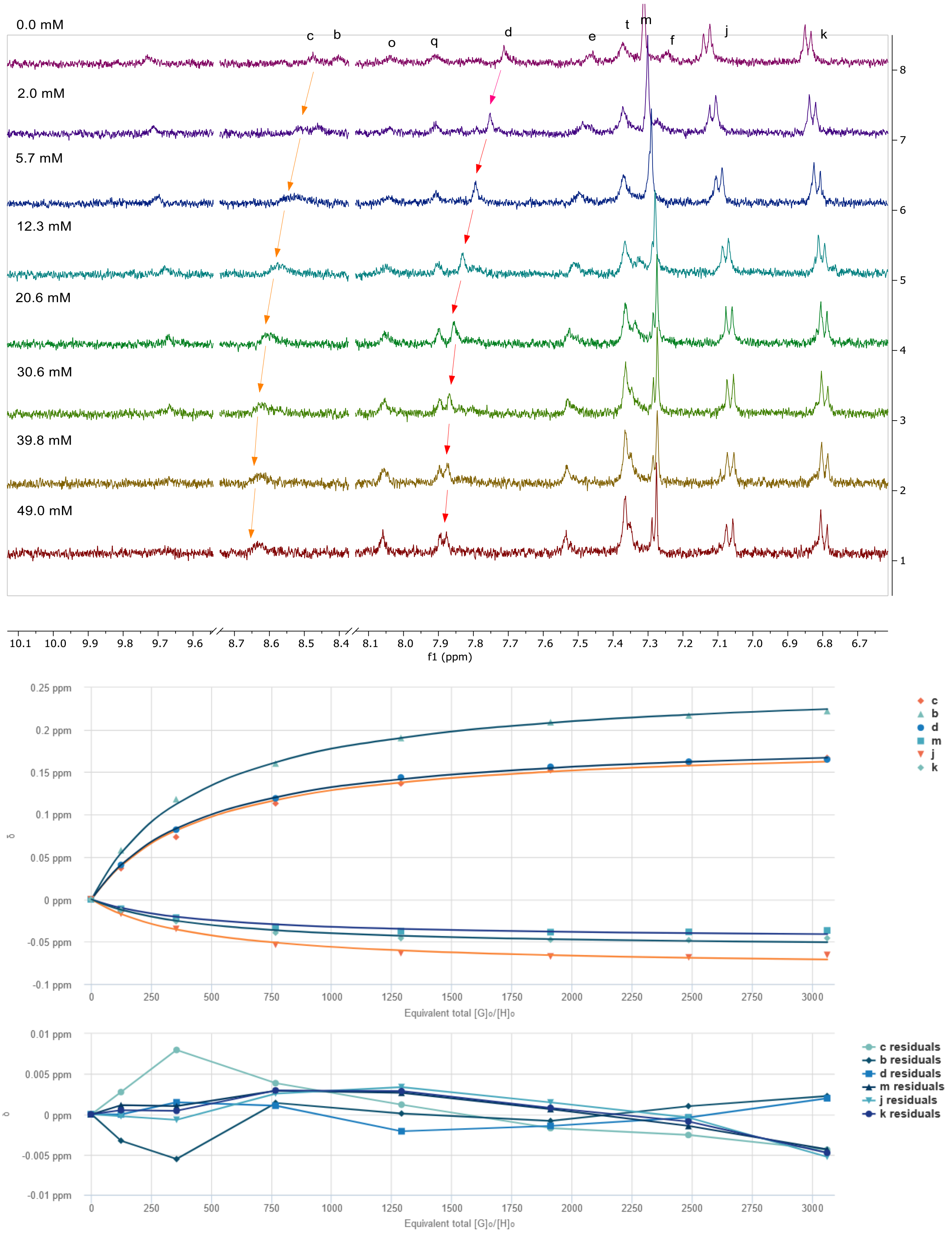

Figure S60. Top). ^{1}H NMR spectra (500 MHz, 1:9 D_2O/H_2O) for receptor **9** (16 µM) titrated with a combined solution of sodium bromide (100 mM) and receptor **9** (16 µM). The concentrations of the guest added are listed in the graph. Bottom). Global fitting of the binding isotherms (protons b, c, d m, j and k) from Bindfit to a 1:1 model K_a = 136.9 M^{-1} (± 3.0 %). Full fitted data is available online at: http://app.supramolecular.org/bindfit/view/0a09e9a1-bf1b-4576-8561-c4ef975daf34

Iodide

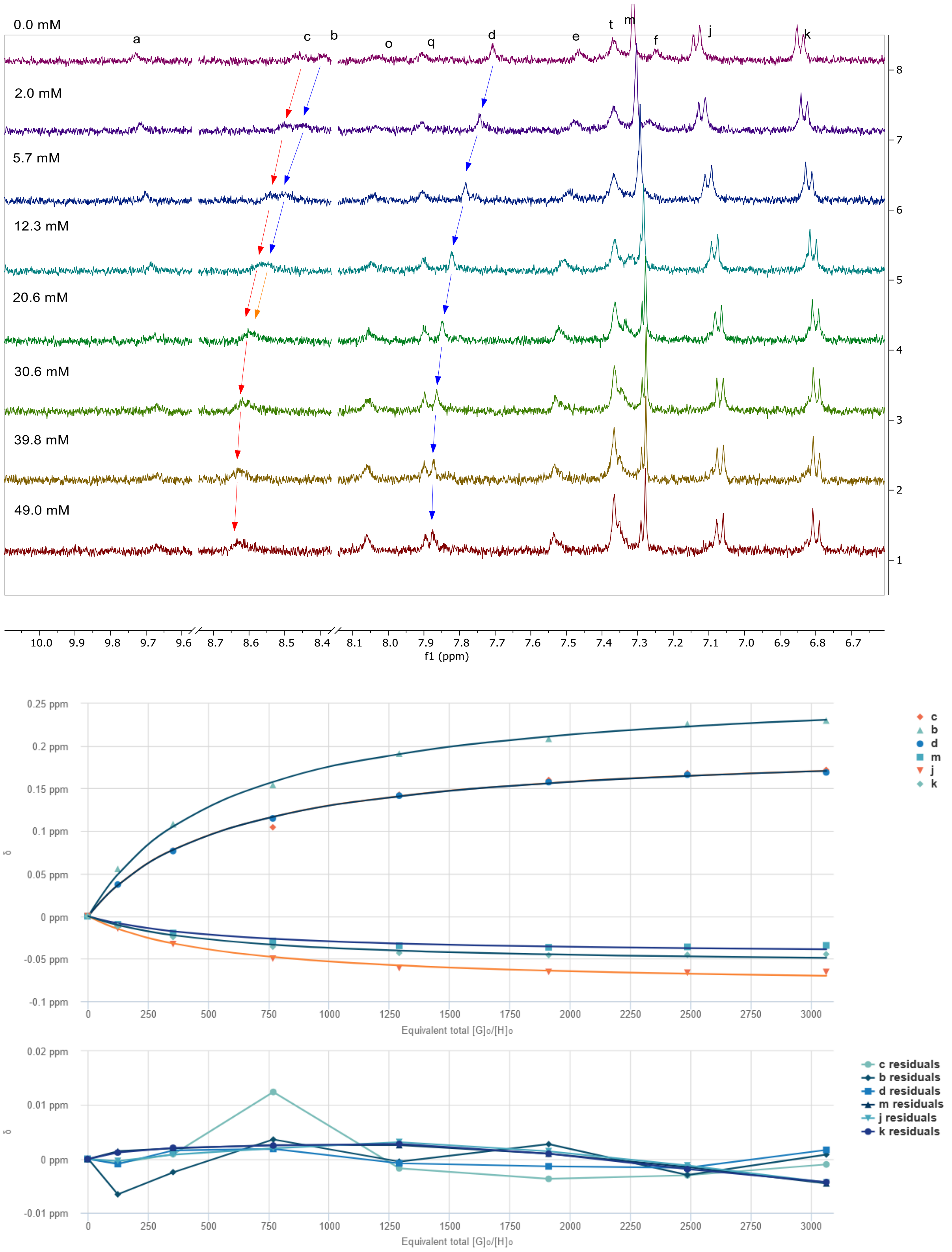

Figure S61. Top). ^{1}H NMR spectra (500 MHz, 1:9 D_2O/H_2O) for receptor **9** (16 µM) titrated with a combined solution of sodium iodide (100 mM) and receptor **9** (16 µM). The concentrations of the guest added are listed in the graph. Bottom). Global fitting of the binding isotherms (protons b, c, d m, j and k) from Bindfit to a 1:1 model K_a = 111.9 M^{-1} (± 3.1 %). Full fitted data is available online at: http://app.supramolecular.org/bindfit/view/0d1d6192-9a9a-4009-b169-181c8d89b3e4

Sulphate

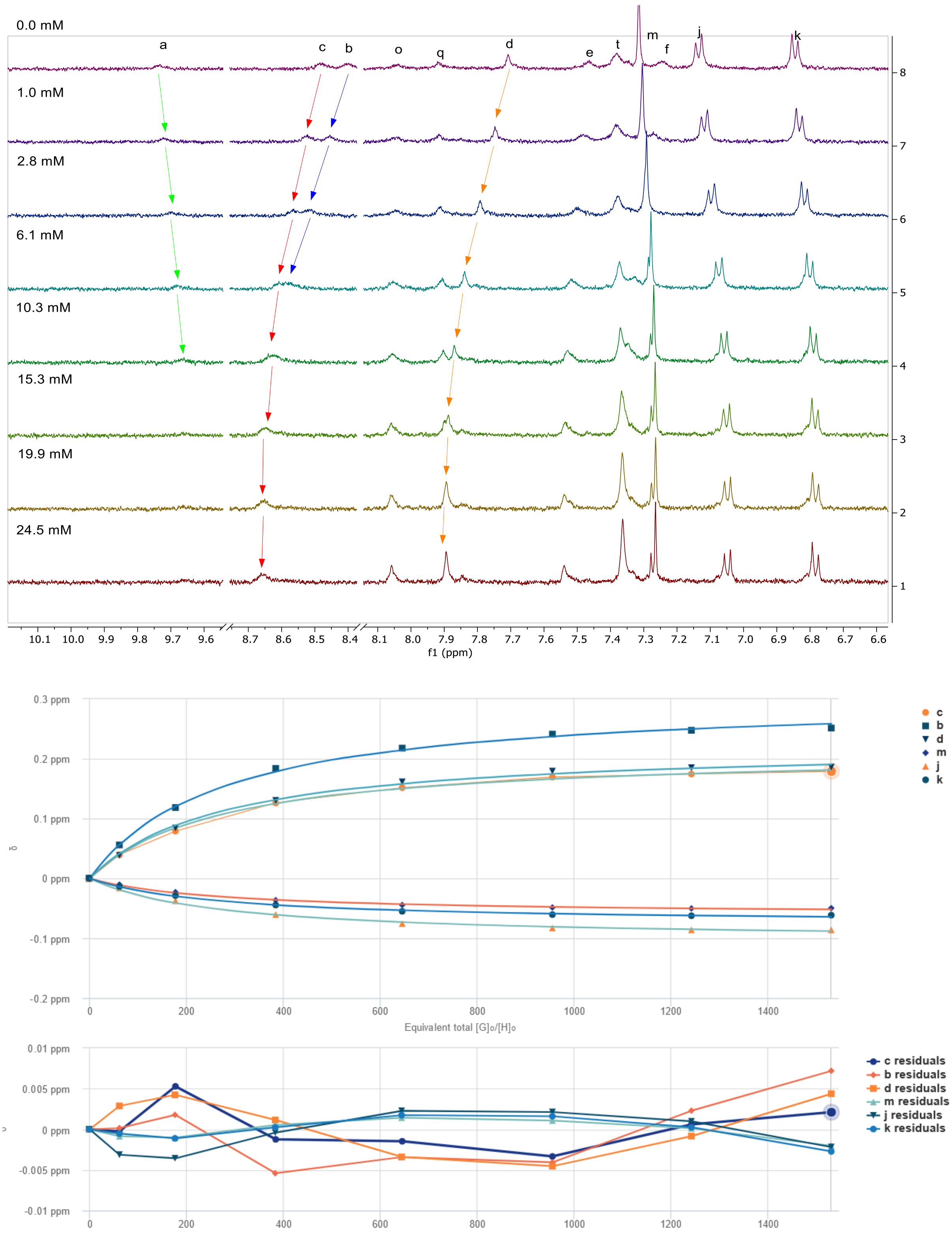

Figure S62. Top). ^{1}H NMR spectra (500 MHz, 1:9 D_2O/H_2O) for the receptor **9** (16 μM) titrated with a combined solution of sodium sulphate (50 mM) and receptor **9** (16 μM). The concentrations of the guest added are listed in the graph. Bottom). Global fitting of the binding isotherms (protons b, c, d m, j and k) from Bindfit to a 1:1 model K_a = 231.6 M^{-1} (± 2.5 %). Full fitted data is available online at:
http://app.supramolecular.org/bindfit/view/42175108-419e-451c-b091-8489ab33c0bf

Nitrate

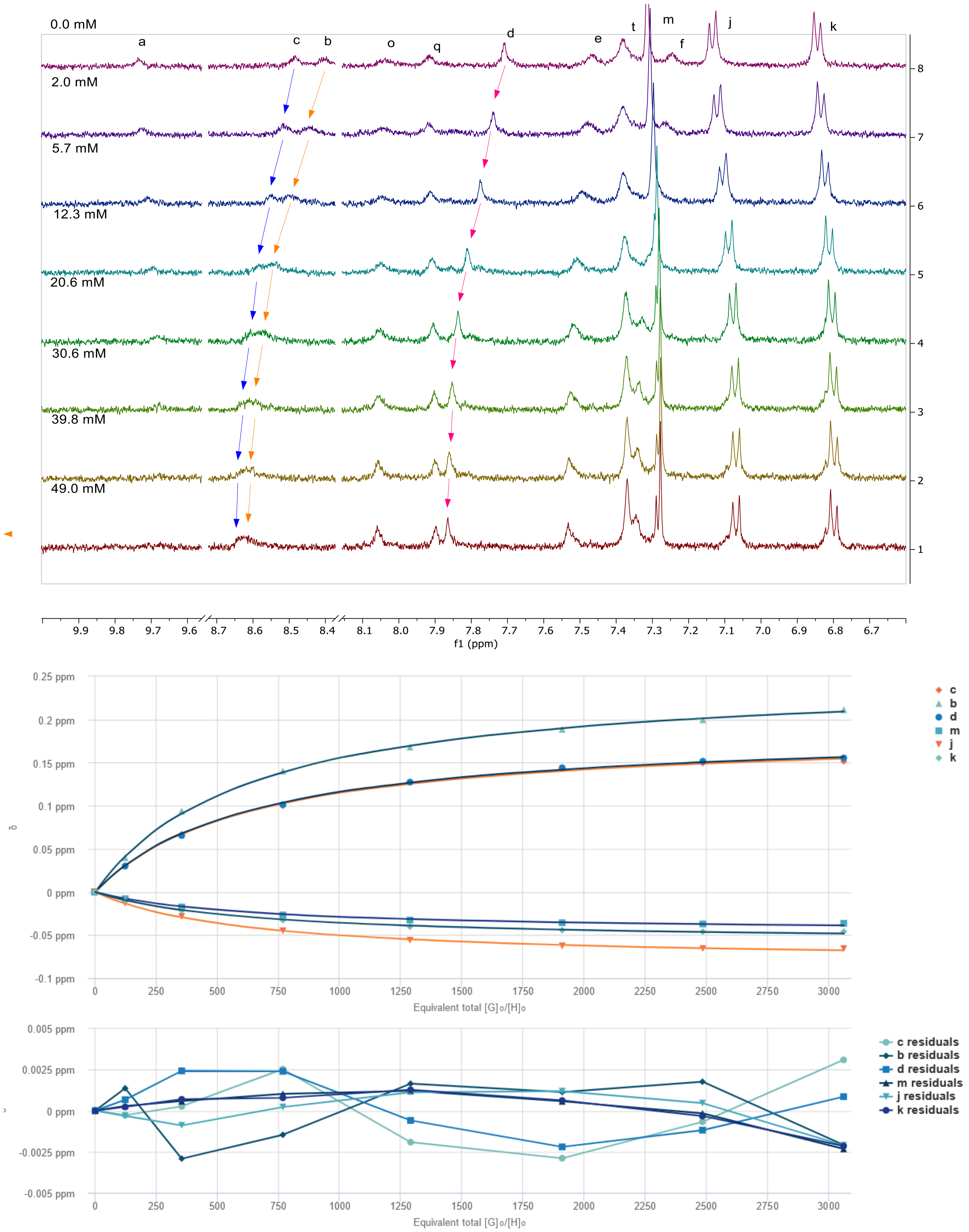

Figure S63. Top). ^{1}H NMR spectra (500 MHz, 1:9 D_2O/H_2O) for receptor **9** (16 µM) titrated with a combined solution of sodium nitrate (100 mM) and receptor **9** (16 µM). The concentrations of the guest added are listed in the graph. Bottom). Global fitting of the binding isotherms (protons b, c, d m, j and k) from Bindfit to a 1:1 model K_a = 99.6 M^{-1} (± 1.6 %). Full fitted data is available online at: http://app.supramolecular.org/bindfit/view/5a9e3819-f0ad-4f09-969c-a4f8a0216ca0

2.1.8 Receptor 11 in H_2O/D_2O

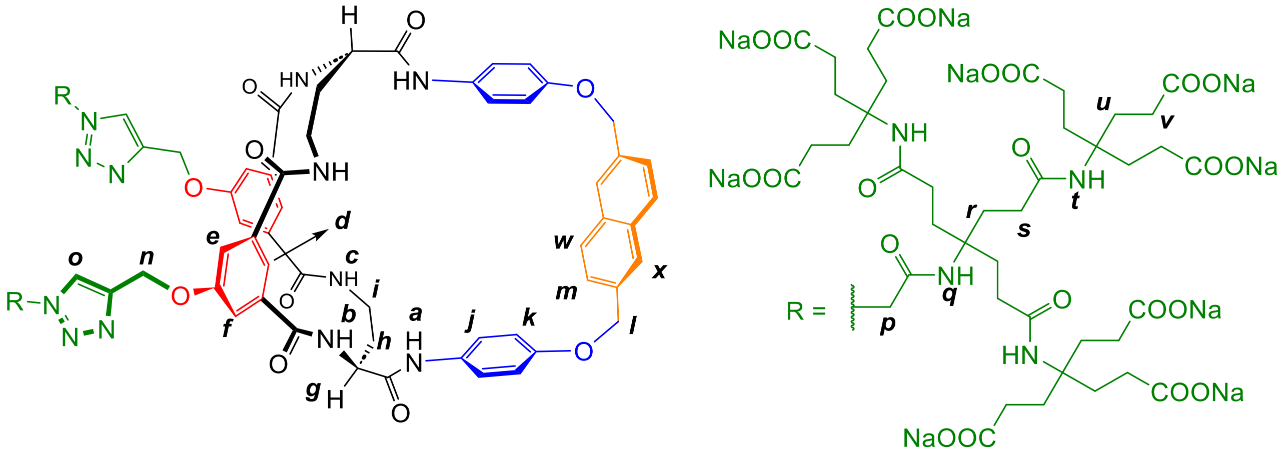

Formate

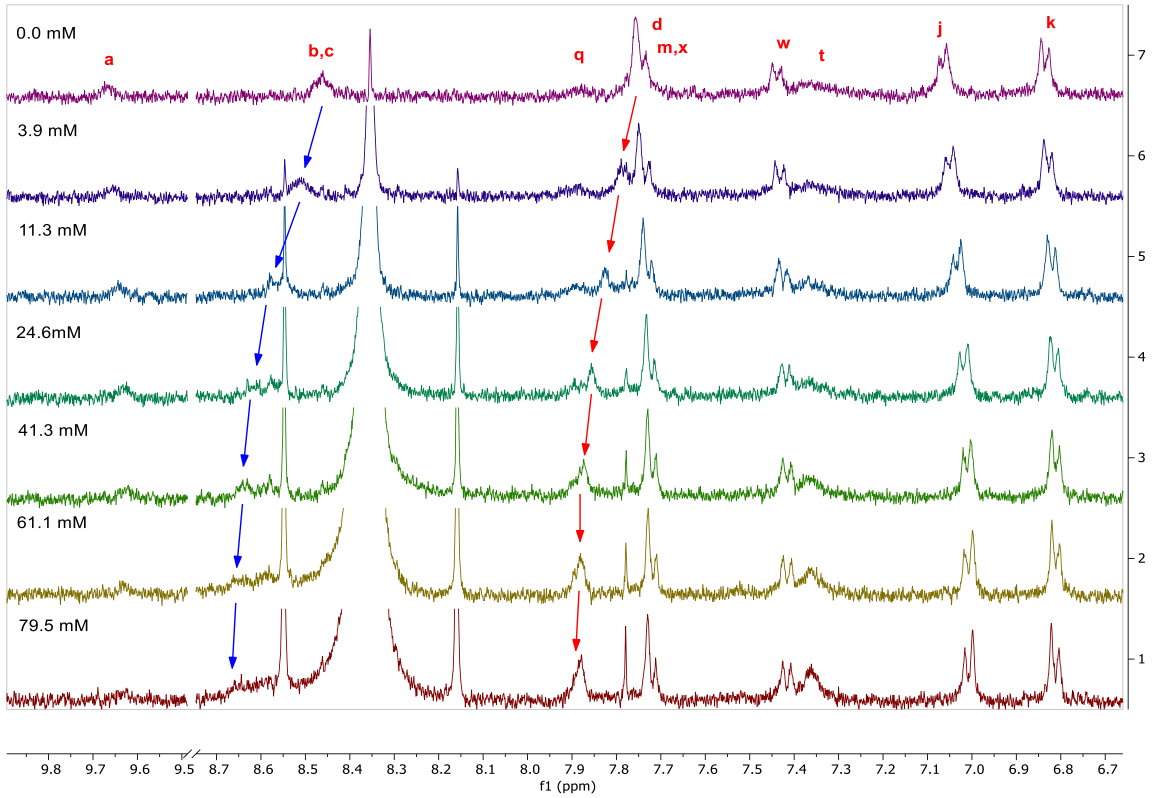

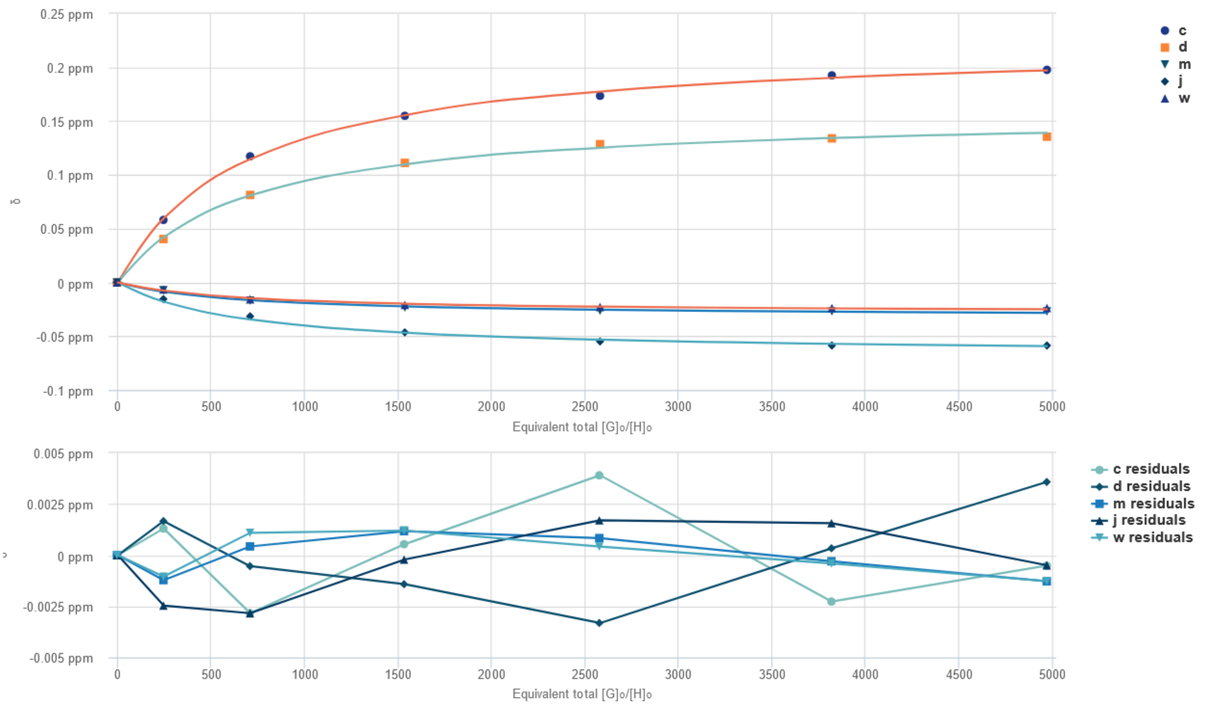

Figure S64. Top). ^{1}H NMR spectra (500 MHz, 1:9 D_2O/H_2O) for receptor **11** (16 µM) titrated with a combined solution of sodium formate (200 mM) and receptor **11** (16 µM). The concentrations of the guest added are listed in the graph. Bottom). Global fitting of the binding isotherms (protons c, d m, j and w) from Bindfit to a 1:1 model K_a = 92.6 M^{-1} (± 2.9 %). Full fitted data is available online at:

http://app.supramolecular.org/bindfit/view/b44313a1-607e-4cd5-a7e8-bce6bd56d4e7

Acetate

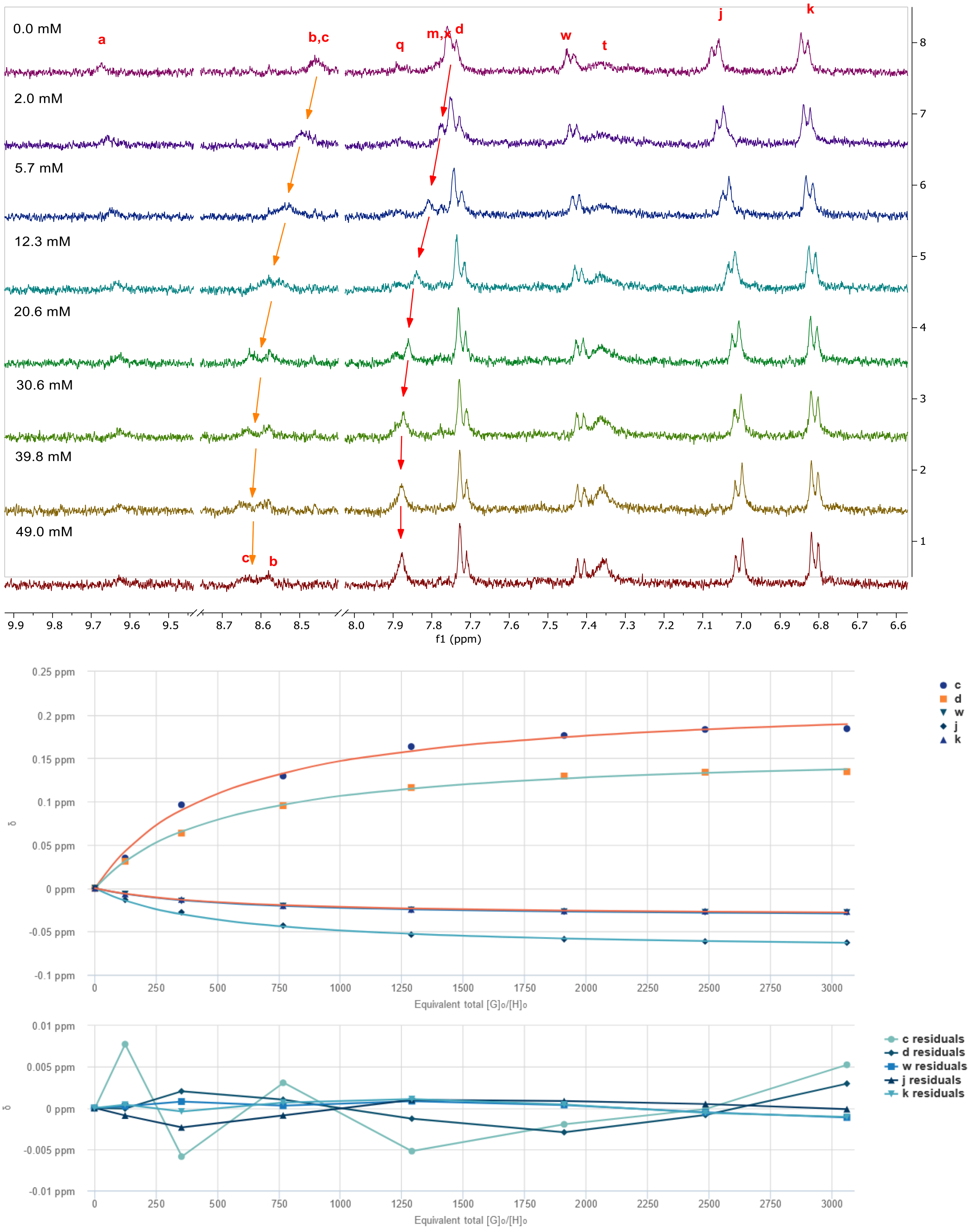

Figure S65. Top). ^{1}H NMR spectra (500 MHz, 1:9 D_2O/H_2O) for receptor **11** (16 µM) titrated with a combined solution of sodium acetate (100 mM) and receptor **11** (16 µM). The concentrations of the guest added are listed in the graph. Bottom). Global fitting of the binding isotherms (protons c, d w j and k) from Bindfit to a 1:1 model K_a = 122.5 M^{-1} (± 3.6 %). Full fitted data is available online at: http://app.supramolecular.org/bindfit/view/6d317fc4-a10f-40ad-b29d-8f0488dc5ead

Propionate

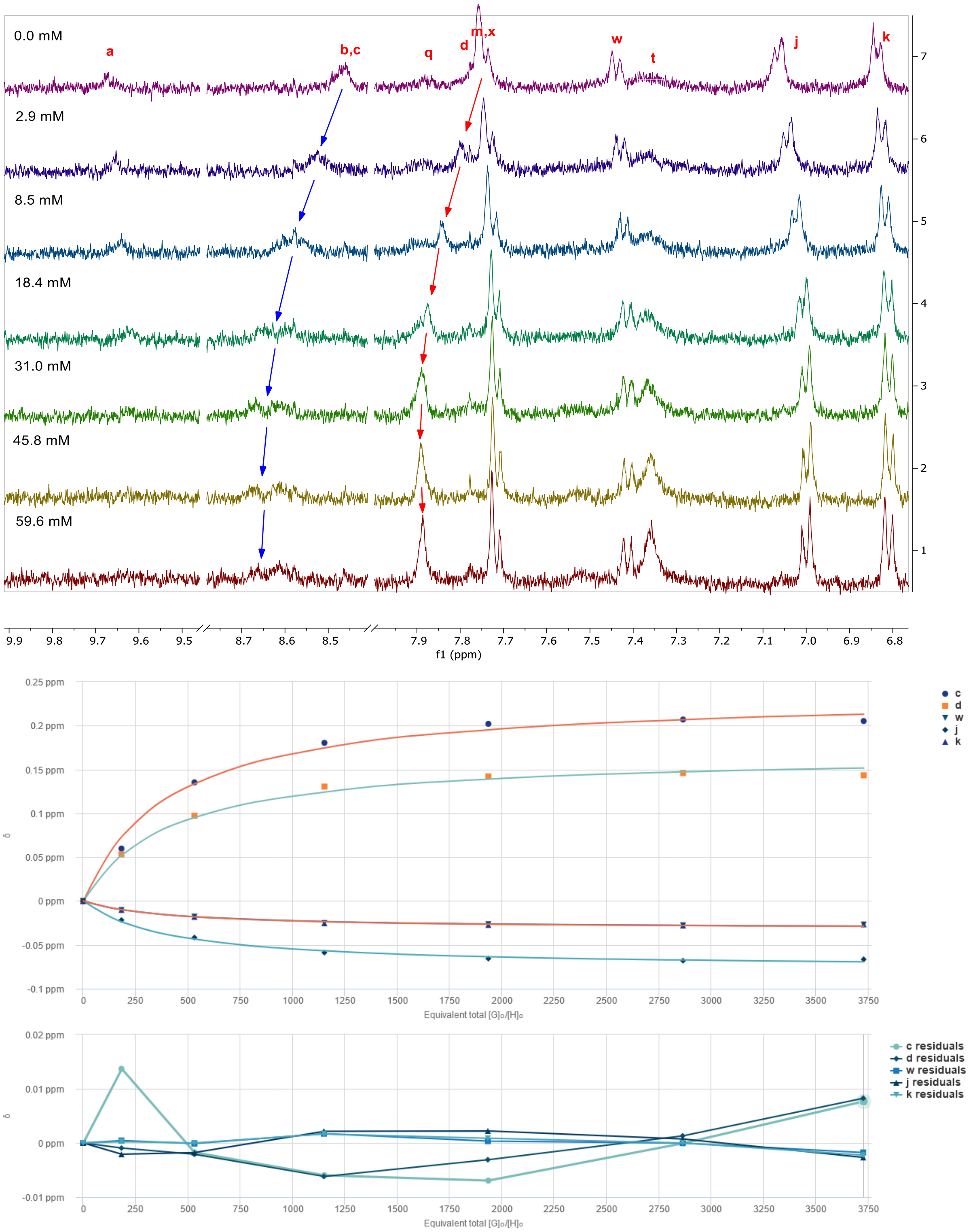

Figure S66. Top). ^{1}H NMR spectra (500 MHz, 1:9 D_2O/H_2O) for receptor **11** (16 µM) titrated with a combined solution of sodium propionate (150 mM) and receptor **11** (16 µM). The concentrations of the guest added are listed in the graph. Bottom). Global fitting of the binding isotherms (protons c, d w j and k) from Bindfit to a 1:1 model K_a = 153.6 M^{-1} (± 6.6 %). Full fitted data is available online at: http://app.supramolecular.org/bindfit/view/d83f5b8d-4899-4a59-b51b-3c1cf7a561b1

n-Butyrate

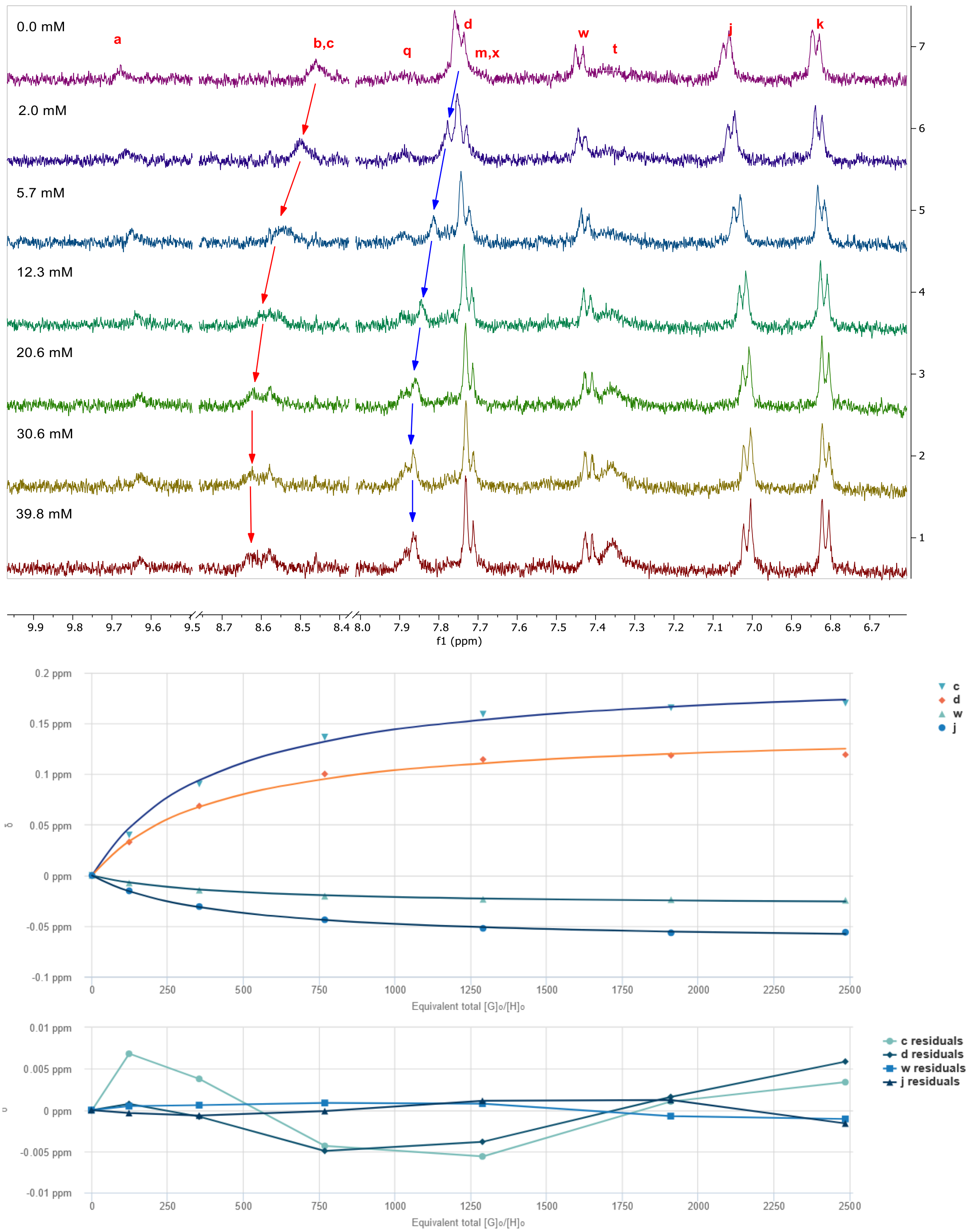

Figure S67. Top). ^{1}H NMR spectra (500 MHz, 1:9 D_2O/H_2O) for receptor **11** (16 µM) titrated with a combined solution of sodium *n*-butyrate (100 mM) and receptor **11** (16 µM). The concentrations of the guest added are listed in the graph. Bottom). Global fitting of the binding isotherms (protons c, d w and j) from Bindfit to a 1:1 model K_a = 154.2 M^{-1} (± 5.3 %). Full fitted data is available online at: http://app.supramolecular.org/bindfit/view/84155767-882c-4e54-abb5-e61287d50249

iso-Butyrate

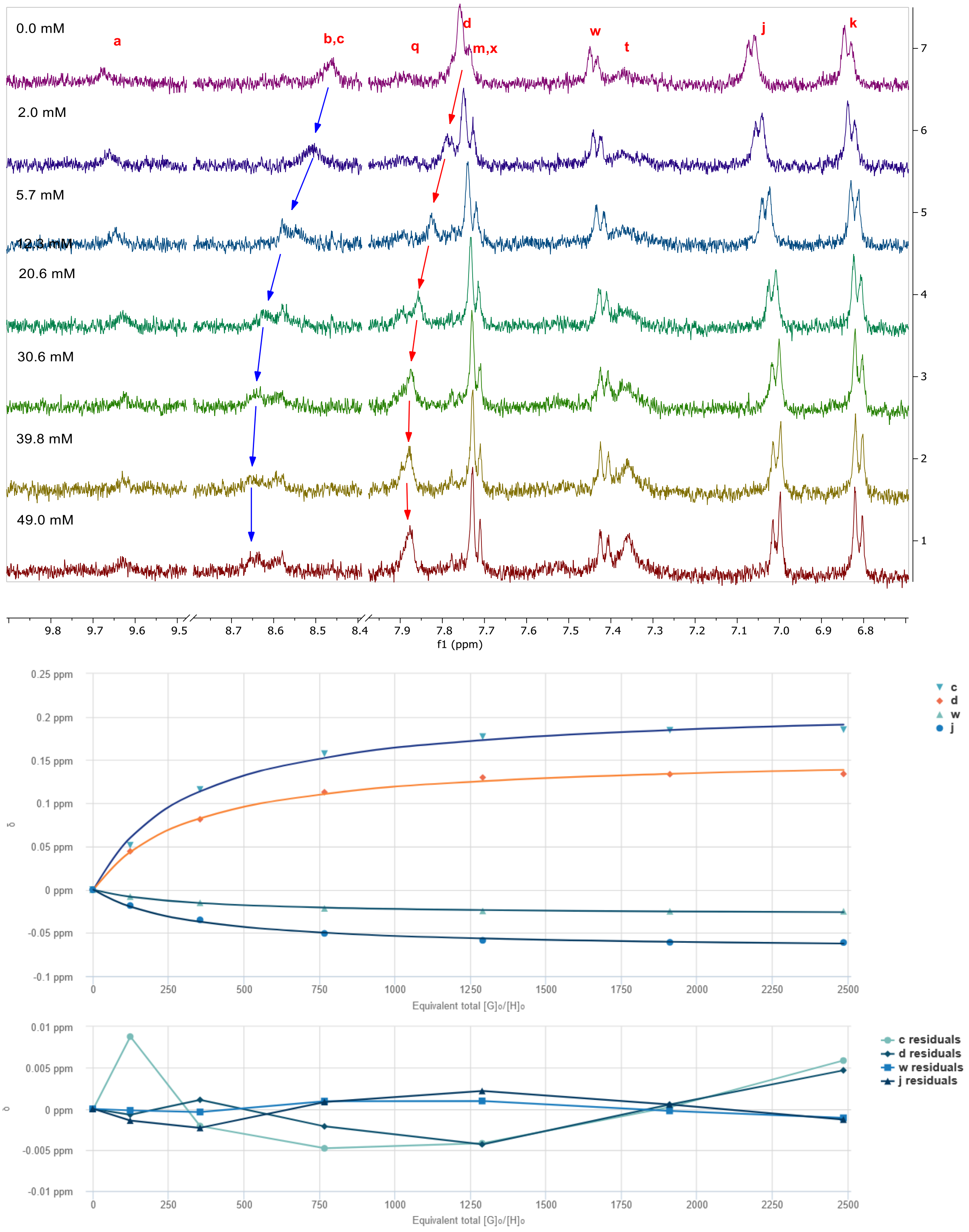

Figure S68. Top). ^1H NMR spectra (500 MHz, 1:9 D_2O/H_2O) for receptor **11** (16 µM) titrated with a combined solution of sodium *iso*-butyrate (100 mM) and receptor **11** (16 µM). The concentrations of the guest added are listed in the graph. Bottom). Global fitting of the binding isotherms (protons c, d w and j) from Bindfit to a 1:1 model K_a = 198.3 M^{-1} (± 5.4 %). Full fitted data is available online at: http://app.supramolecular.org/bindfit/view/b7fbe5aa-e0fe-4fea-b608-25723c622d5b

Pivalate

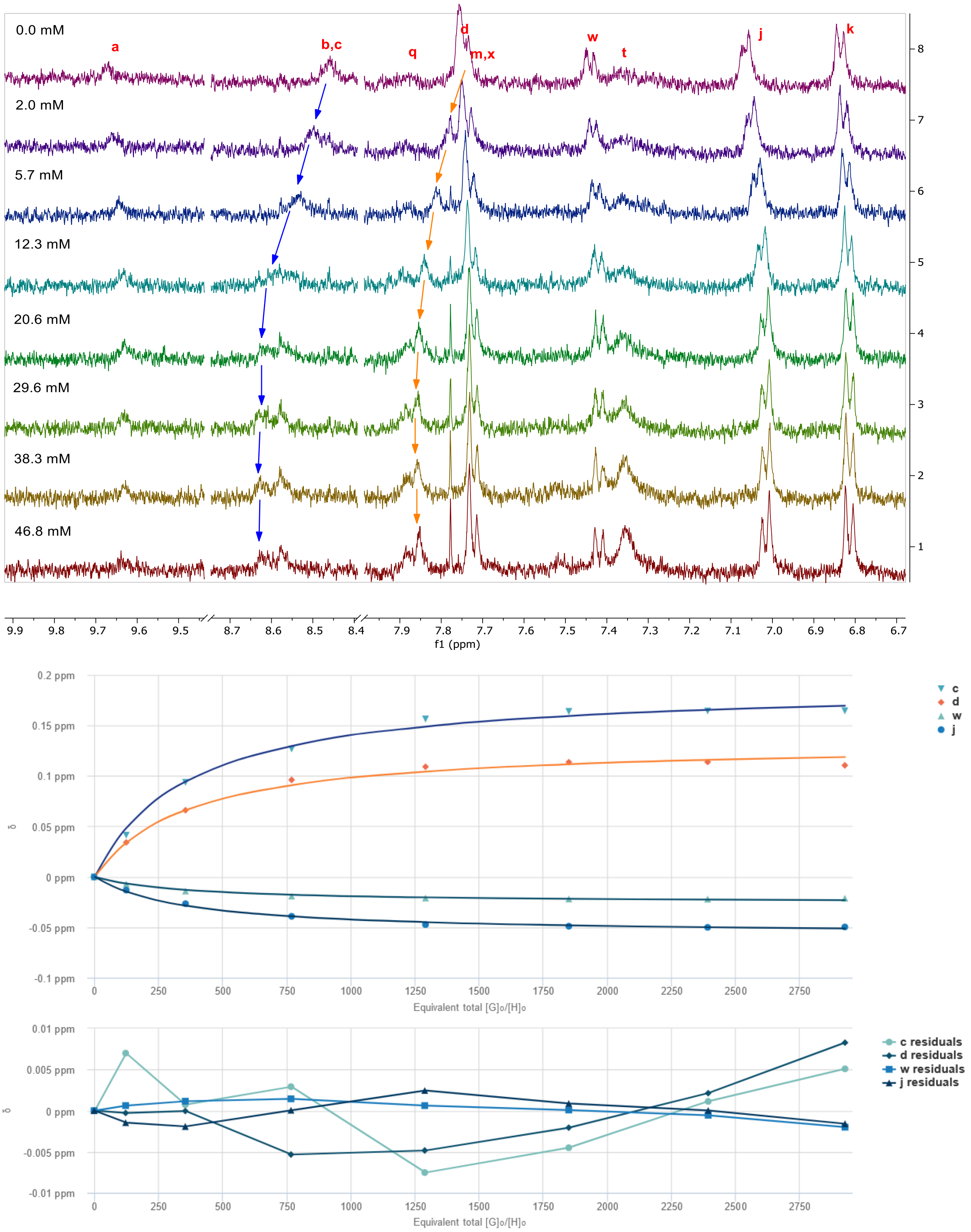

Figure S69. Top). ^{1}H NMR spectra (500 MHz, 1:9 D_2O/H_2O) for receptor **11** (16 µM) titrated with a combined solution of sodium pivalate (100 mM) and receptor **11** (16 µM). The concentrations of the guest added are listed in the graph. Bottom). Global fitting of the binding isotherms (protons c, d w and j) from Bindfit to a 1:1 model K_a = 173.6 M^{-1} (± 6.4 %). Full fitted data is available online at: http://app.supramolecular.org/bindfit/view/86083c1a-199a-474c-8b08-d92e673711ff

Benzoate-d5

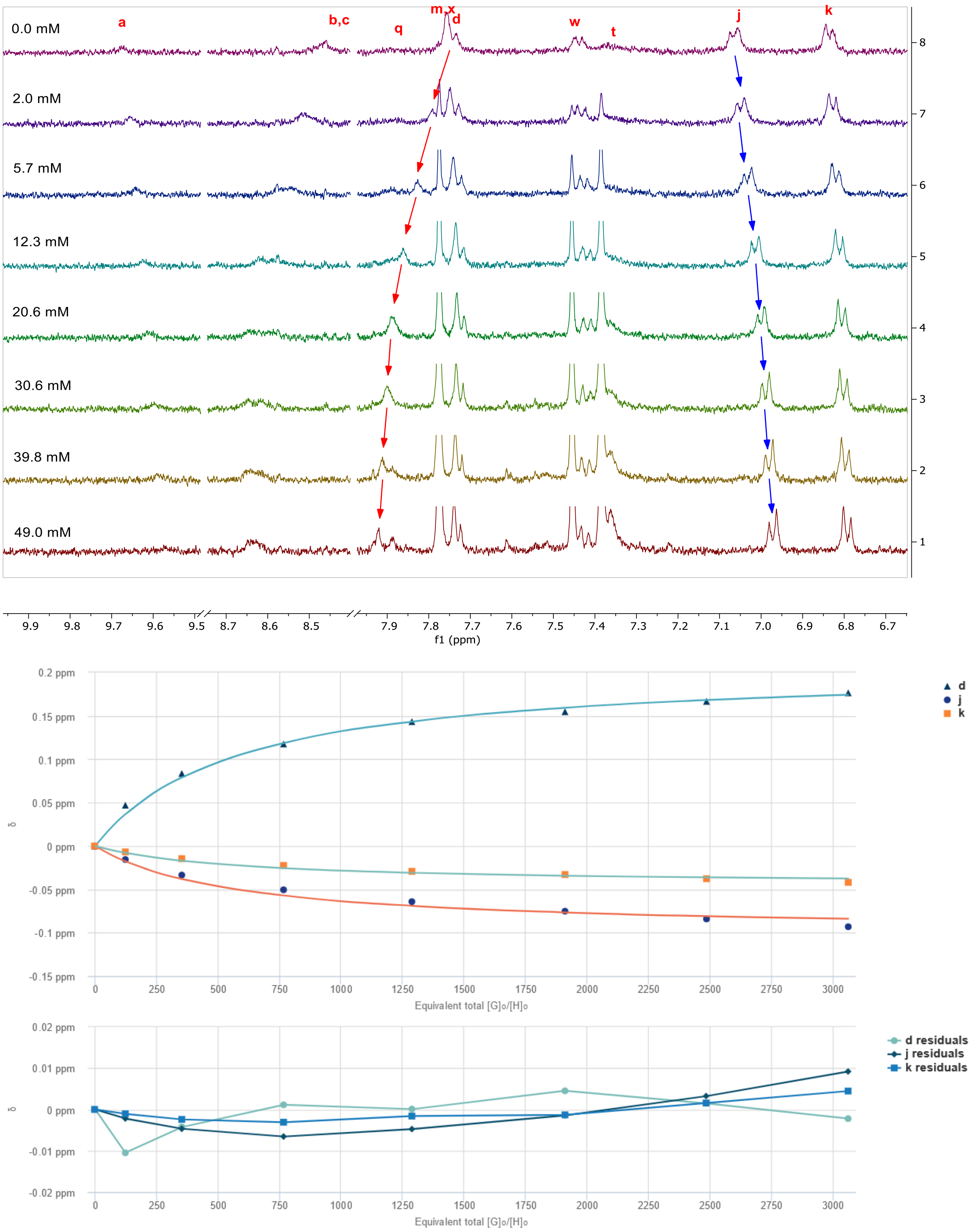

Figure S70. Top). ^{1}H NMR spectra (500 MHz, 1:9 D_2O/H_2O) for receptor **11** (16 µM) titrated with a combined solution of sodium benzoate-*d*^{5} (100 mM) and receptor **11** (16 µM). The concentrations of the guest added are listed in the graph. Bottom). Global fitting of the binding isotherms (protons d j and k) from Bindfit to a 1:1 model K_a = 109.9 M^{-1} (± 8.0 %). Full fitted data is available online at: http://app.supramolecular.org/bindfit/view/c09eb541-7067-4caf-abb8-3e44261fdaac

D-Lactate

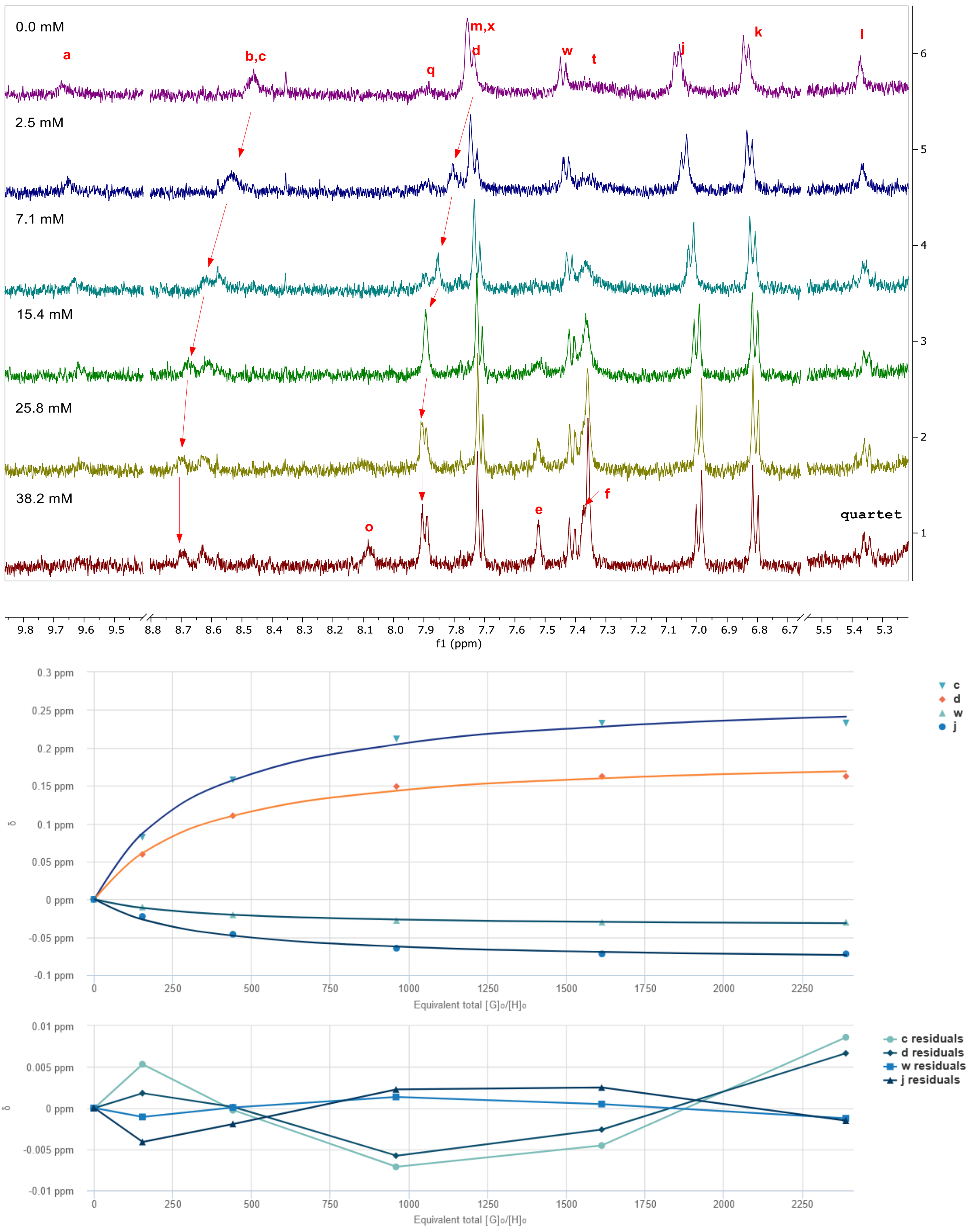

Figure S71. Top). ^{1}H NMR spectra (500 MHz, 1:9 D_2O/H_2O) for receptor **11** (16 µM) titrated with a combined solution of sodium *D*-Lactate (125 mM) and receptor **11** (16 µM). The concentrations of the guest added are listed in the graph. Bottom). Global fitting of the binding isotherms (protons c, d, j and w) from Bindfit to a 1:1 model K_a = 191.0 M^{-1} (± 5.9 %). Full fitted data is available online at: http://app.supramolecular.org/bindfit/view/789f9c03-6ded-41c0-b1b2-39db7659f57a

L-Lactate

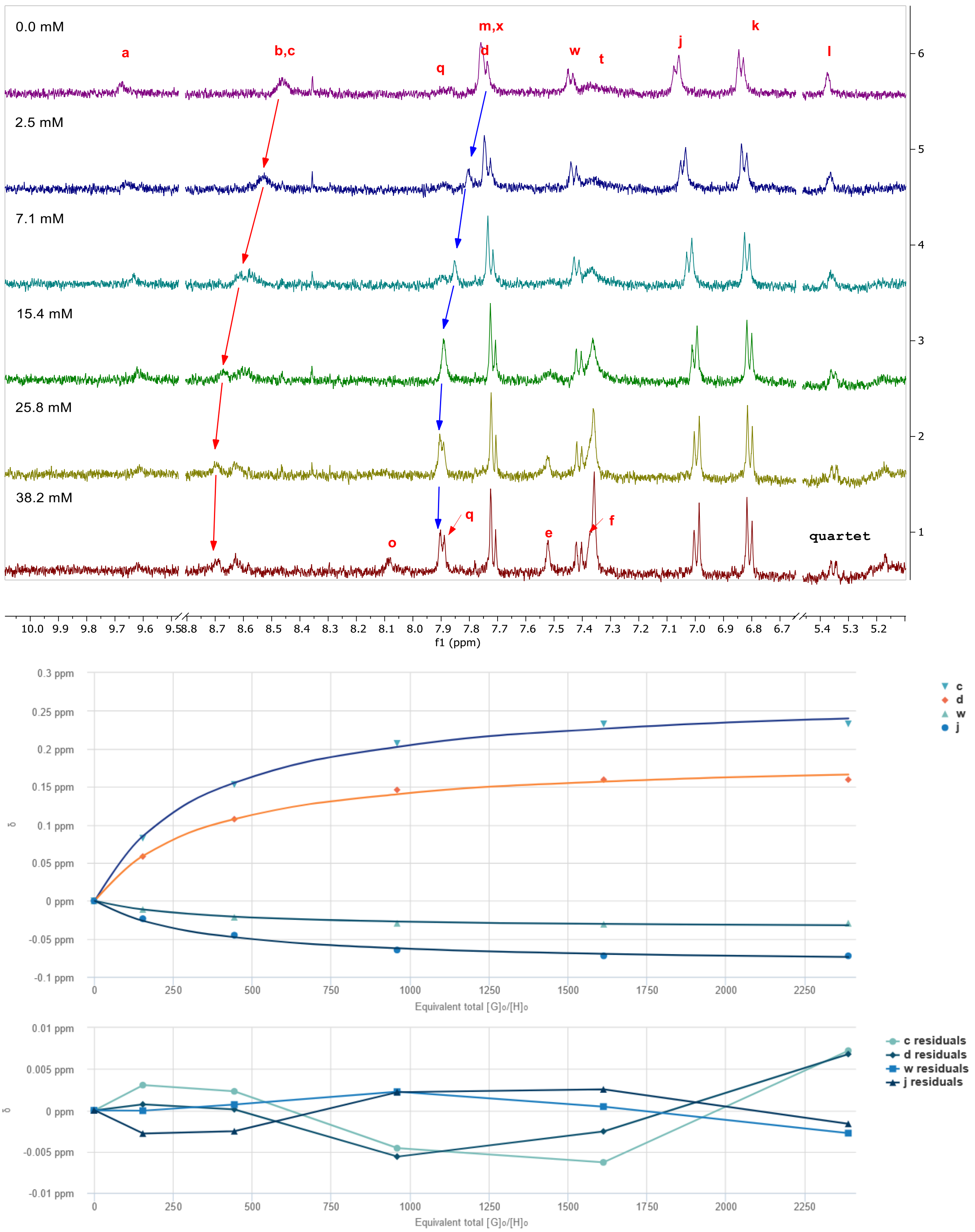

Figure S72. Top). ^{1}H NMR spectra (500 MHz, 1:9 D_2O/H_2O) for receptor **11** (16 µM) titrated with a combined solution of sodium *L*-Lactate (125 mM) and receptor **11** (16 µM). The concentrations of the guest added are listed in the graph. Bottom). Global fitting of the binding isotherms (protons c, d, j and w) from Bindfit to a 1:1 model K_a = 185.1 M^{-1} (± 5.4 %). Full fitted data is available online at: http://app.supramolecular.org/bindfit/view/68a1efb0-df4d-441c-ad4d-9532b266b2b2

Chloride

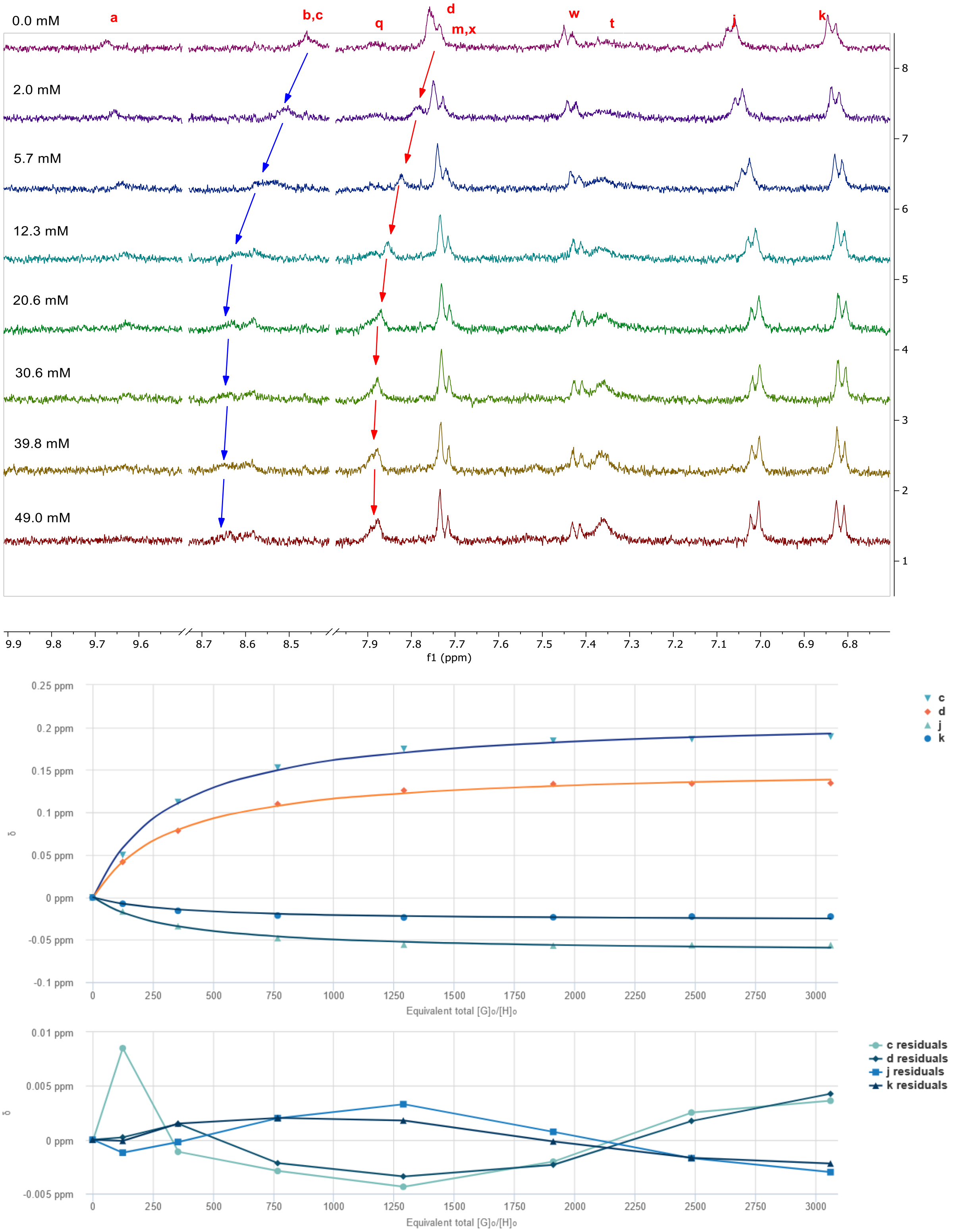

Figure S73. Top). ^{1}H NMR spectra (500 MHz, 1:9 D_2O/H_2O) for receptor **11** (16 µM) titrated with a combined solution of sodium chloride (100 mM) and receptor **11** (16 µM). The concentrations of the guest added are listed in the graph. Bottom). Global fitting of the binding isotherms (protons c, d j and k) from Bindfit to a 1:1 model K_a = 192.3 M^{-1} (± 4.8 %). Full fitted data is available online at: http://app.supramolecular.org/bindfit/view/2d09d020-f514-45e2-8b6f-b3c537d3dc46

Bromide

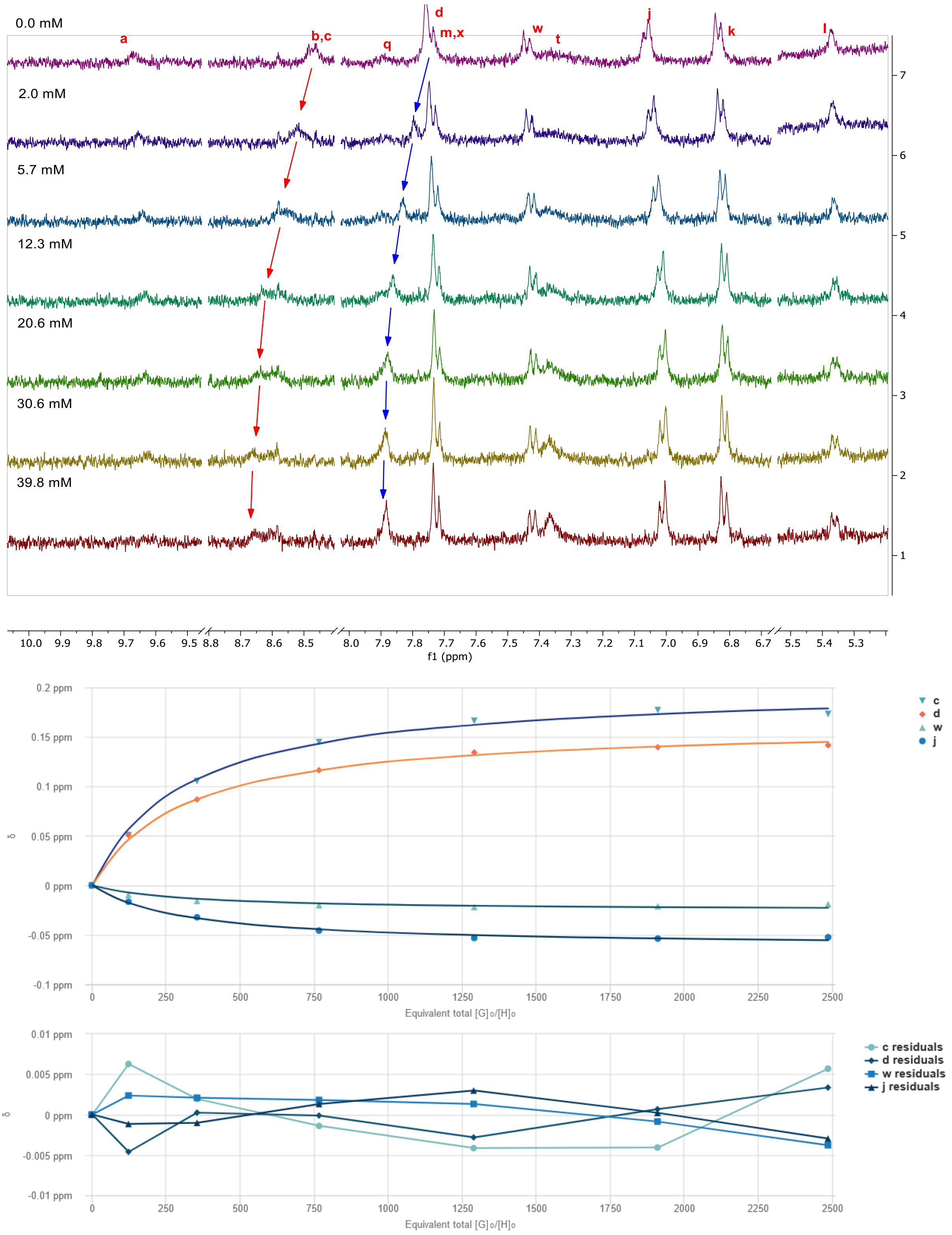

Figure S74. Top). ^{1}H NMR spectra (500 MHz, 1:9 D_2O/H_2O) for receptor **11** (16 µM) titrated with a combined solution of sodium bromide (100 mM) and receptor **11** (16 µM). The concentrations of the guest added are listed in the graph. Bottom). Global fitting of the binding isotherms (protons c, d, j and w) from Bindfit to a 1:1 model K_a = 202.6 M^{-1} (± 5.4 %). Full fitted data is available online at:
http://app.supramolecular.org/bindfit/view/d4b78b7f-641c-4f91-aacd-e401e42f2334

Iodide

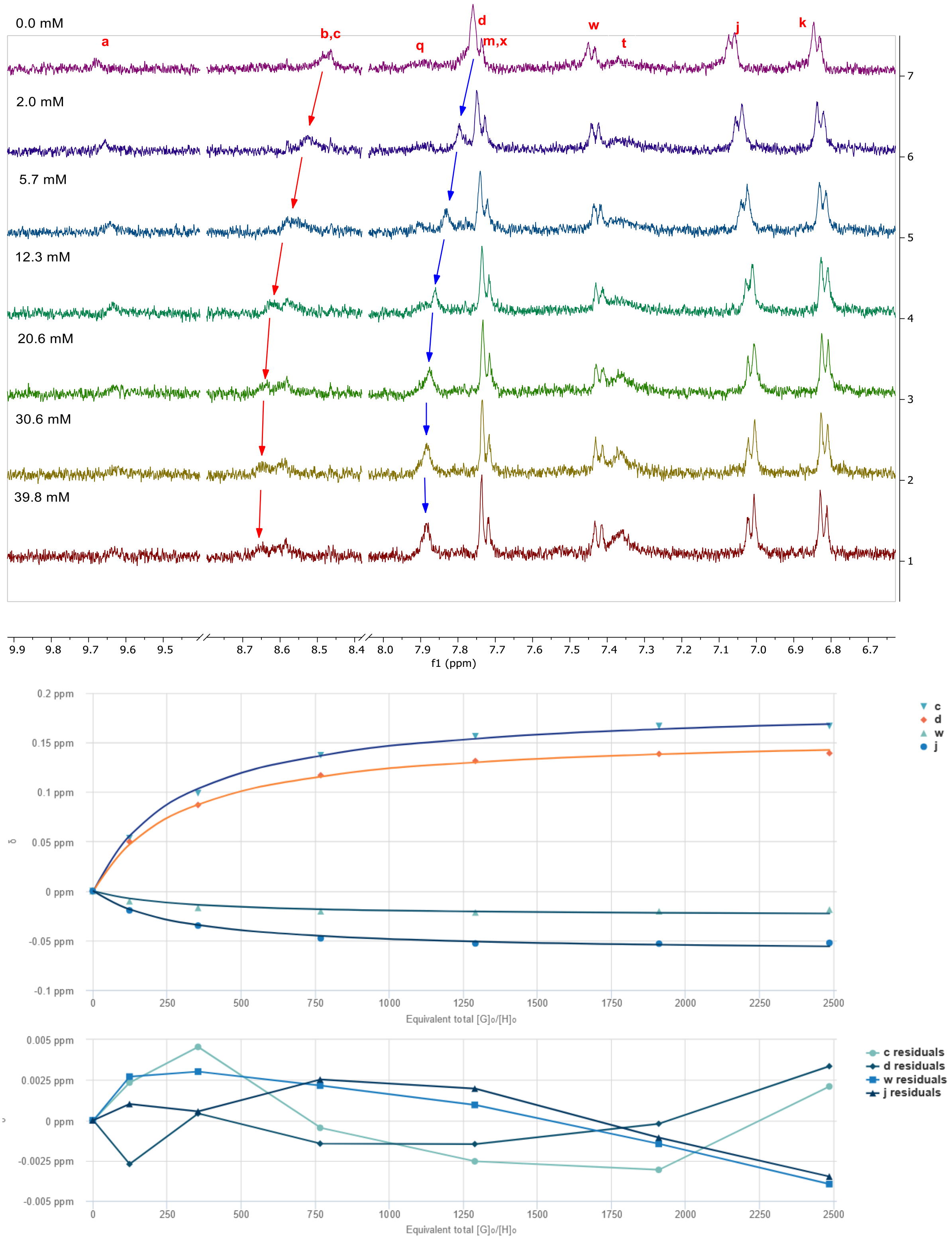

Figure S75. Top). ^{1}H NMR spectra (500 MHz, 1:9 D_2O/H_2O) for receptor **11** (16 µM) titrated with a combined solution of sodium iodide (100 mM) and receptor **11** (16 µM). The concentrations of the guest added are listed in the graph. Bottom). Global fitting of the binding isotherms (protons c, d, j and w) from Bindfit to a 1:1 model K_a = 215.1 M^{-1} (± 4.6 %). Full fitted data is available online at: http://app.supramolecular.org/bindfit/view/69346d50-c290-453d-a8c3-c480a50b62d2

2.1.9 Receptor 13 in H_2O/D_2O

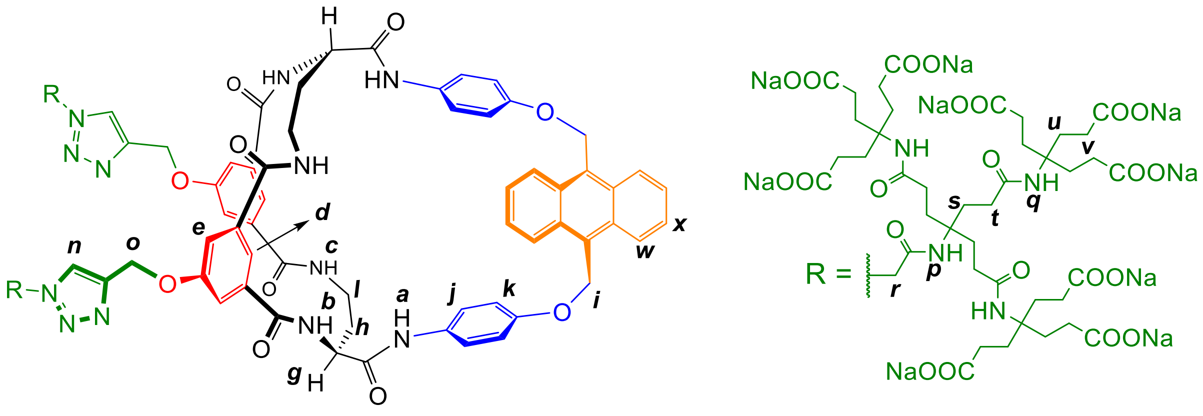

Acetate

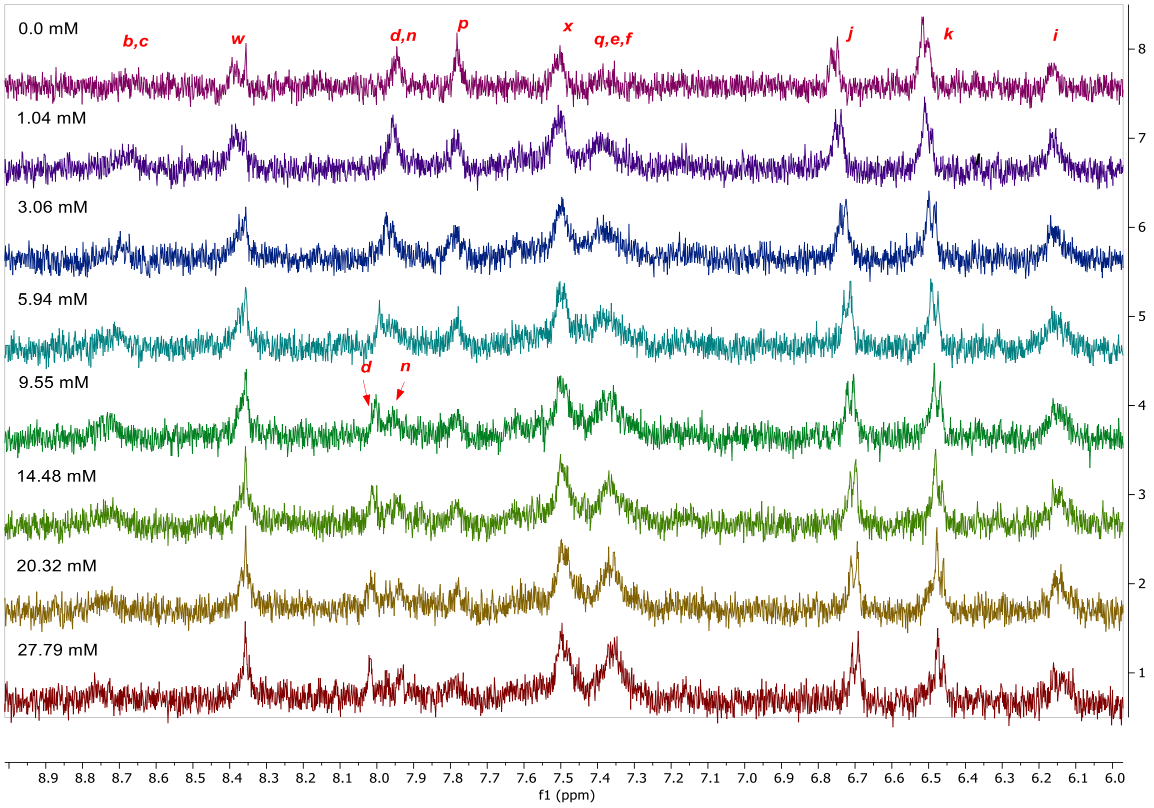

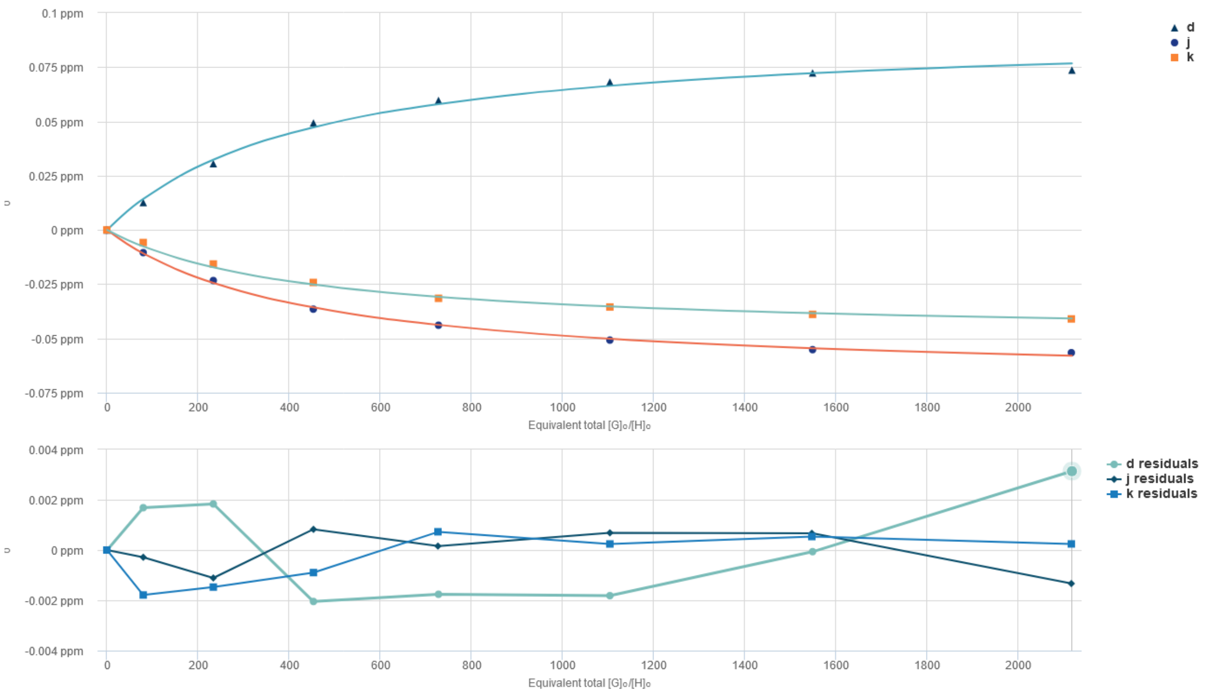

Figure S76. ^{1}H NMR spectra (500 MHz, 1:9 D_2O/H_2O) for receptor **13** (15 µM) titrated with a combined solution of sodium acetate (105 mM) and receptor **13** (15 µM). The concentrations of the guest added are listed in the graph. Bottom). Global fitting of the binding isotherms (protons d, j and k) from Bindfit to a 1:1 model K_a = 154.2 M^{-1} (± 4.5 %). Full fitted data is available online at: http://app.supramolecular.org/bindfit/view/139e1d8c-3dcd-456c-8c61-fa94dc2bda11

Propionate

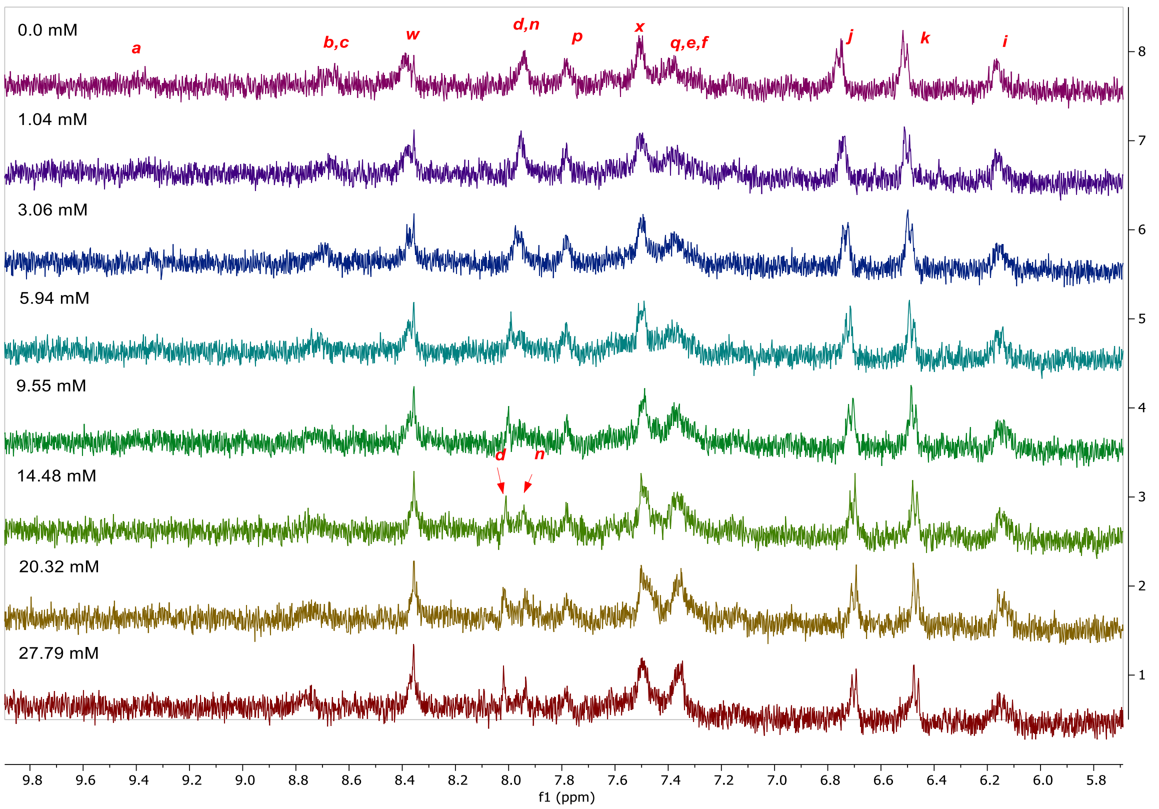

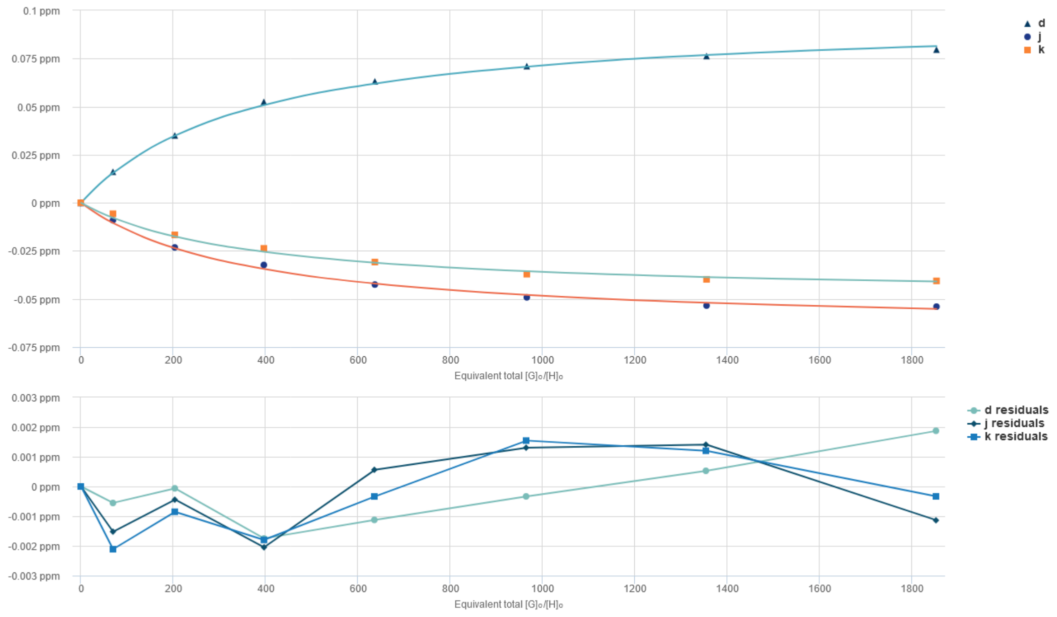

Figure S77. ^{1}H NMR spectra (500 MHz, 1:9 D_2O/H_2O) for receptor **13** (15 µM) titrated with a combined solution of sodium propionate (105 mM) and receptor **13** (15 µM). The concentrations of the guest added are listed in the graph. Bottom). Global fitting of the binding isotherms (protons d, j and k) from Bindfit to a 1:1 model K_a = 182.3 M^{-1} (± 4.1 %). Full fitted data is available online at:
http://app.supramolecular.org/bindfit/view/da5ed684-4682-45cd-becd-653790e9604e

n-butyrate

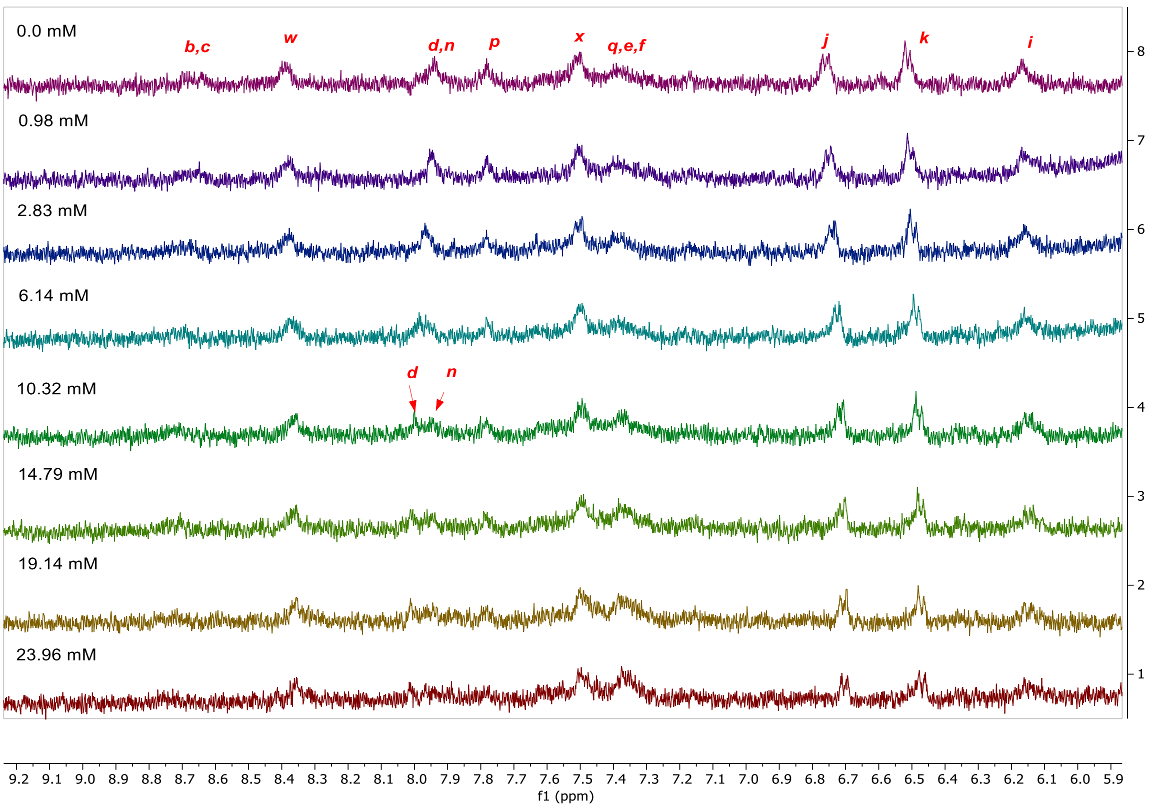

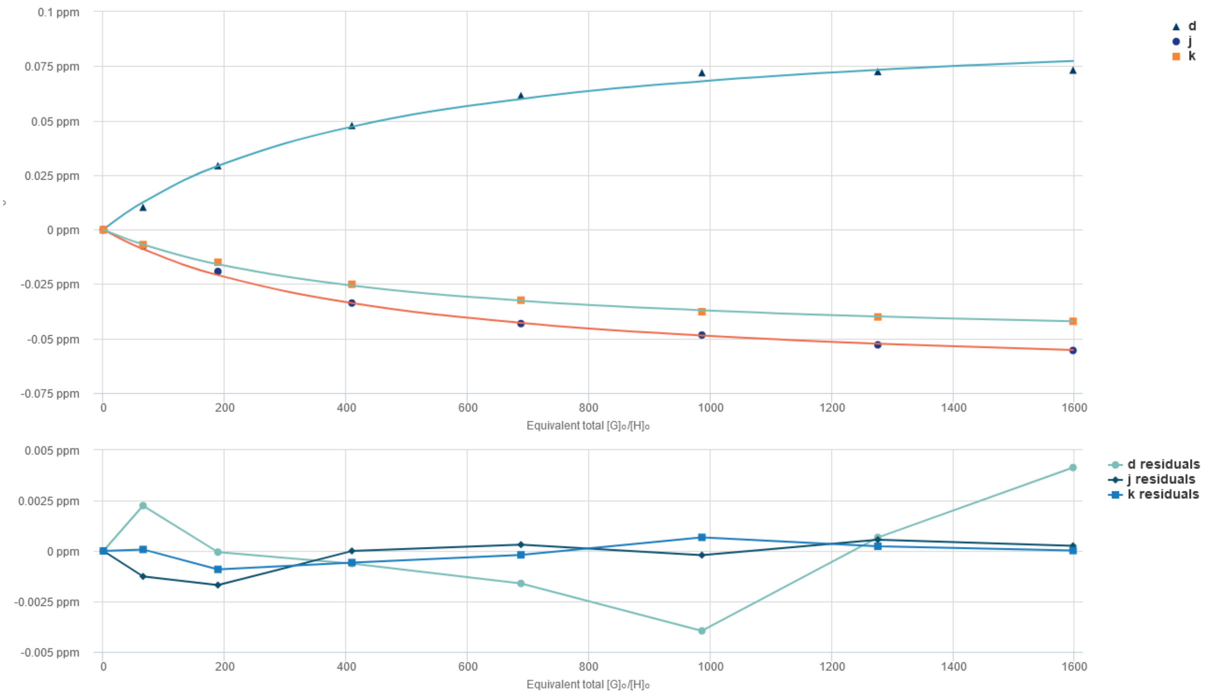

Figure S78. ^{1}H NMR spectra (500 MHz, 1:9 D_2O/H_2O) for receptor **13** (15 µM) titrated with a combined solution of sodium *n*-butyrate (50 mM) and receptor **13** (15 µM). The concentrations of the guest added are listed in the graph. Bottom). Global fitting of the binding isotherms (protons d, j and k) from Bindfit to a 1:1 model K_a = 148.0 M^{-1} (± 4.6 %). Full fitted data is available online at: http://app.supramolecular.org/bindfit/view/8d090220-f738-4264-a6ef-ec44abb7904b

iso-butyrate

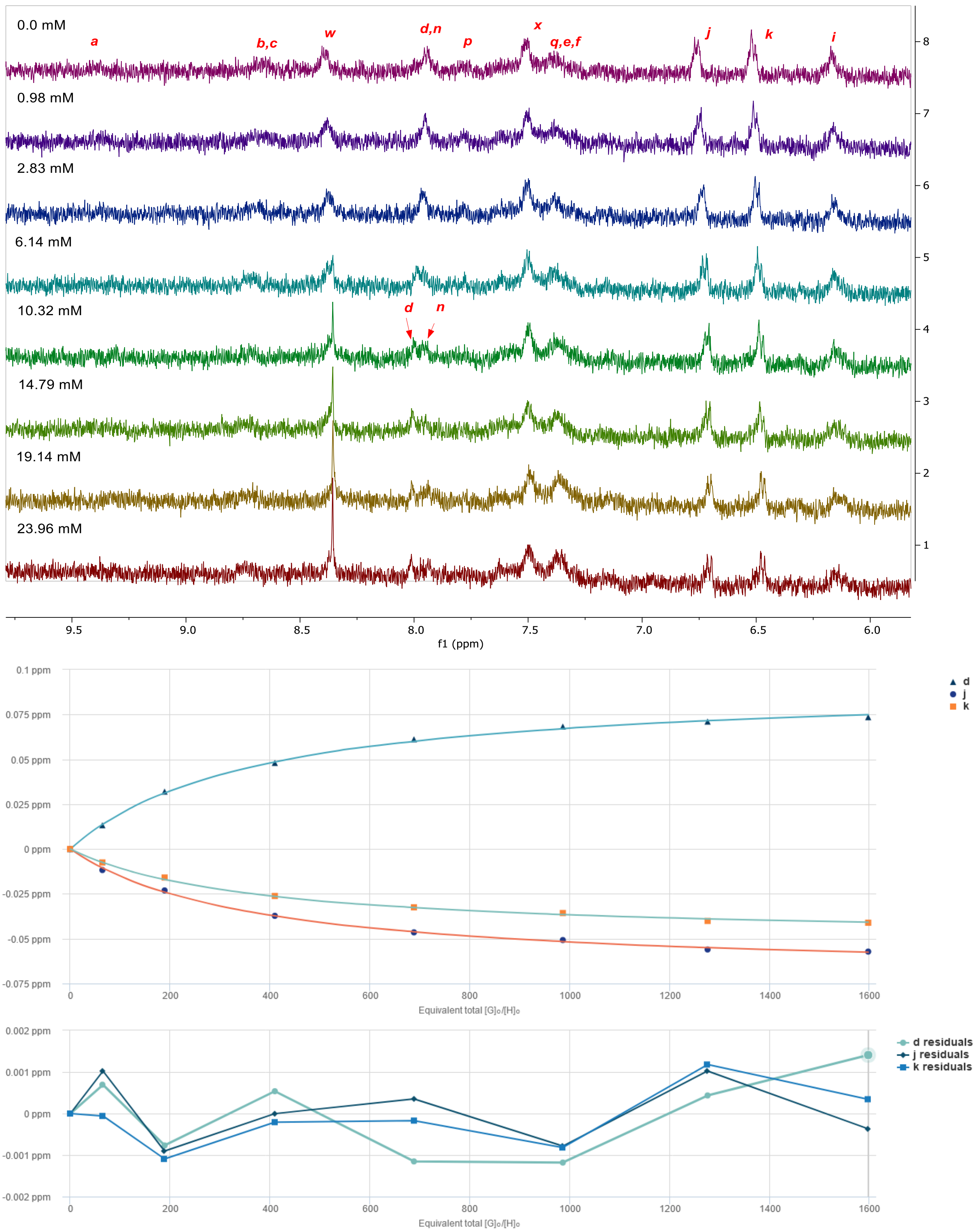

Figure S79. ^{1}H NMR spectra (500 MHz, 1:9 D_2O/H_2O) for receptor **13** (15 µM) titrated with a combined solution of sodium *iso*-butyrate (50 mM) and receptor **13** (15 µM). The concentrations of the guest added are listed in the graph. Bottom). Global fitting of the binding isotherms (protons d, j and k) from Bindfit to a 1:1 model K_a = 181.5 M^{-1} (± 2.7 %). Full fitted data is available online at:
http://app.supramolecular.org/bindfit/view/18940caa-5504-4543-a847-2b01b1bf545b

L-Lactate

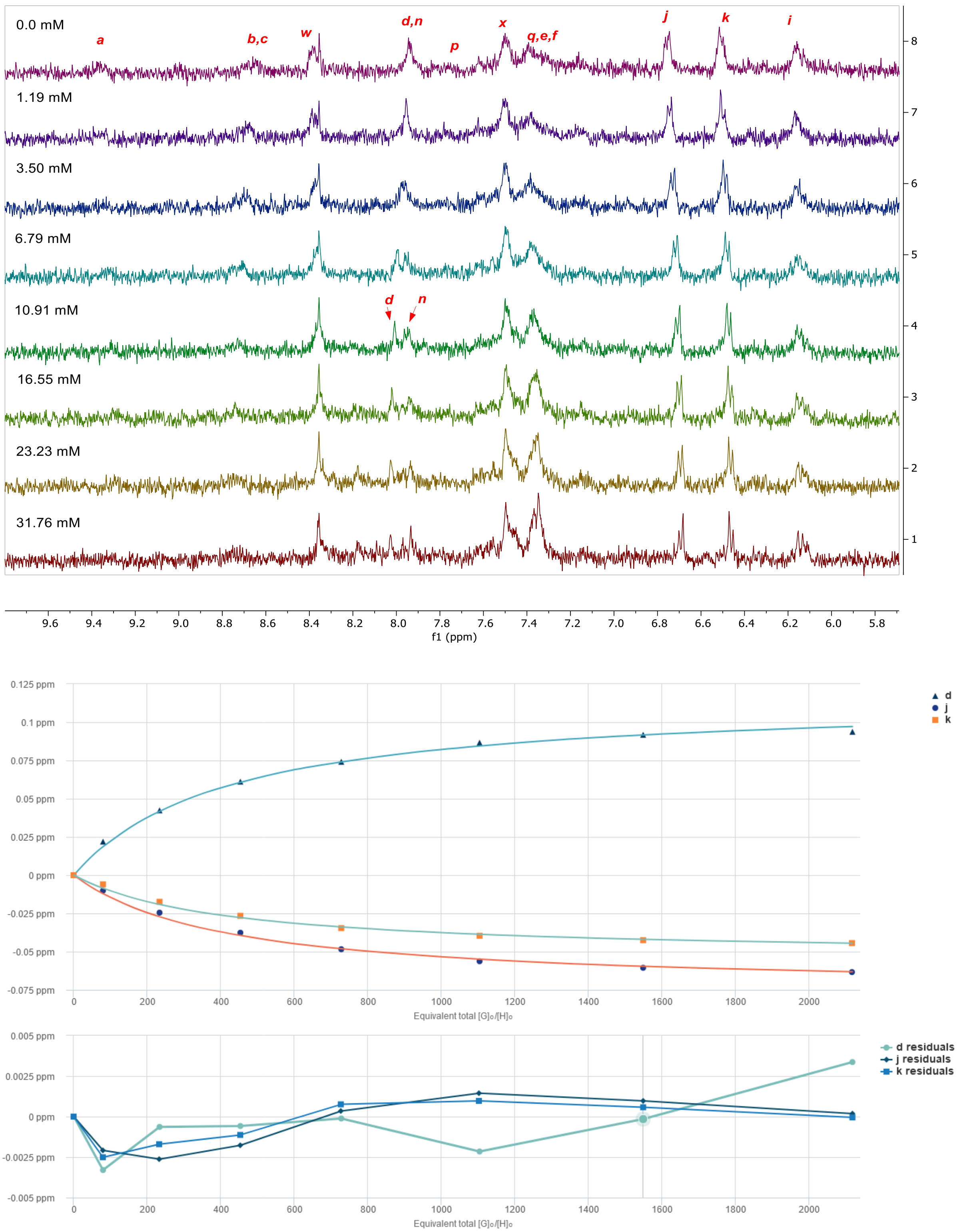

Figure S80. ^{1}H NMR spectra (500 MHz, 1:9 D_2O/H_2O) for receptor **13** (15 µM) titrated with a combined solution of sodium *L*-lactate (120 mM) and receptor **13** (15 µM). The concentrations of the guest added are listed in the graph. Bottom). Global fitting of the binding isotherms (protons d, j and k) from Bindfit to a 1:1 model K_a = 160.7 M^{-1} (± 4.7 %). Full fitted data is available online at: http://app.supramolecular.org/bindfit/view/709977cd-ad43-4fe8-b5cc-f9bf9c59222b

D-Lactate

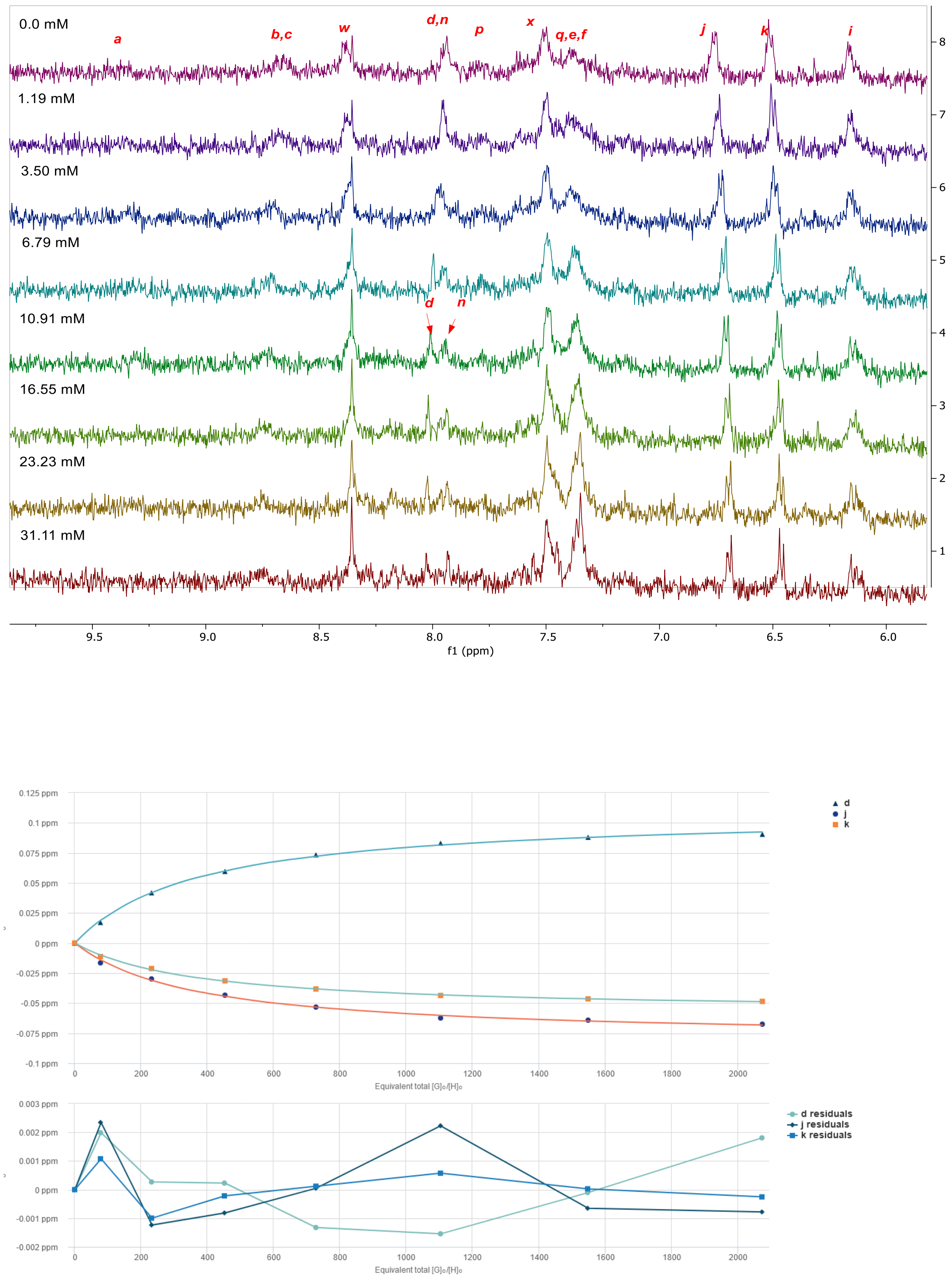

Figure S81. ^{1}H NMR spectra (500 MHz, 1:9 D_2O/H_2O) for receptor **13** (15 µM) titrated with a combined solution of sodium *D*-lactate (120 mM) and receptor **13** (15 µM). The concentrations of the guest added are listed in the graph. Bottom). Global fitting of the binding isotherms (protons d, j and k) from Bindfit to a 1:1 model K_a = 180.5 M^{-1} (± 3.4 %). Full fitted data is available online at: http://app.supramolecular.org/bindfit/view/4cad9744-938e-4ac2-ba7d-5366e678bf59

Benzoate-d^5

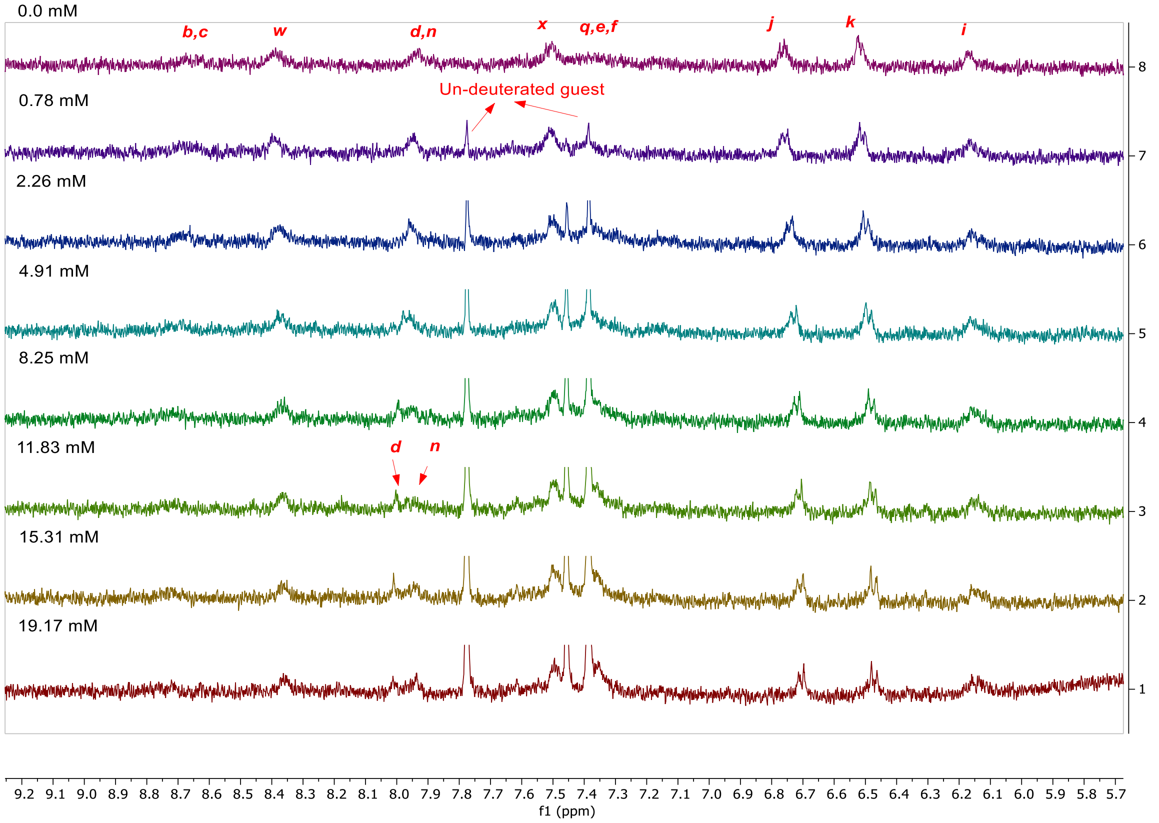

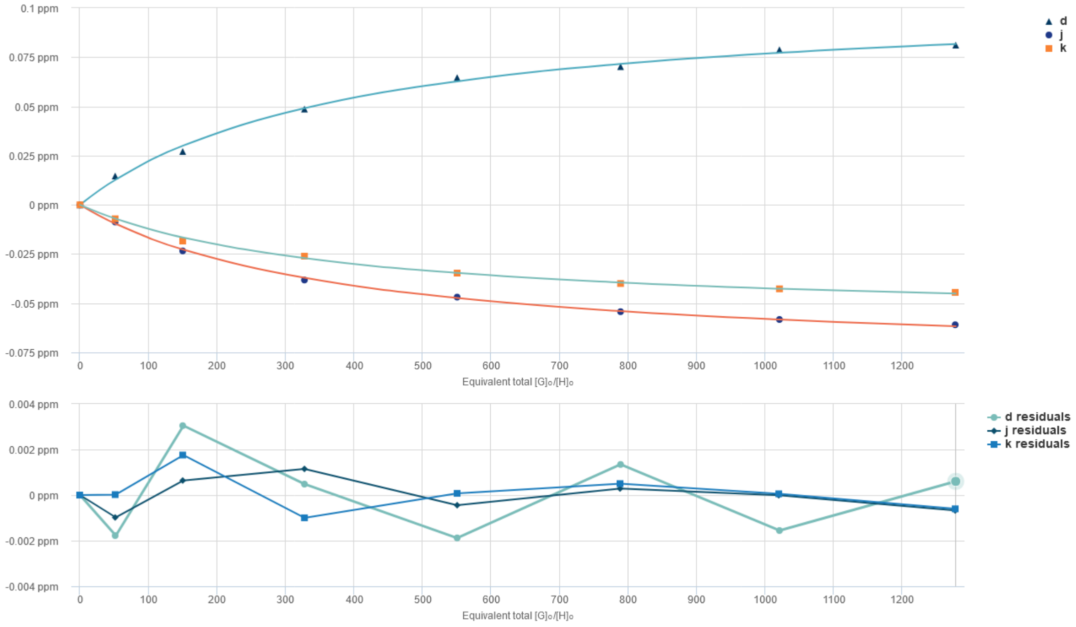

Figure S82. ^{1}H NMR spectra (500 MHz, 1:9 D_2O/H_2O) for receptor **13** (15 µM) titrated with a combined solution of sodium benzoate-d^5 (50 mM) and receptor **13** (15 µM). The concentrations of the guest added are listed in the graph. Bottom). Global fitting of the binding isotherms (protons d, j and k) from Bindfit to a 1:1 model K_a = 176.0 M^{-1} (± 3.3 %). Full fitted data is available online at:
http://app.supramolecular.org/bindfit/view/7532a1d1-a37c-475a-9e8c-94e80606c4f3

Chloride

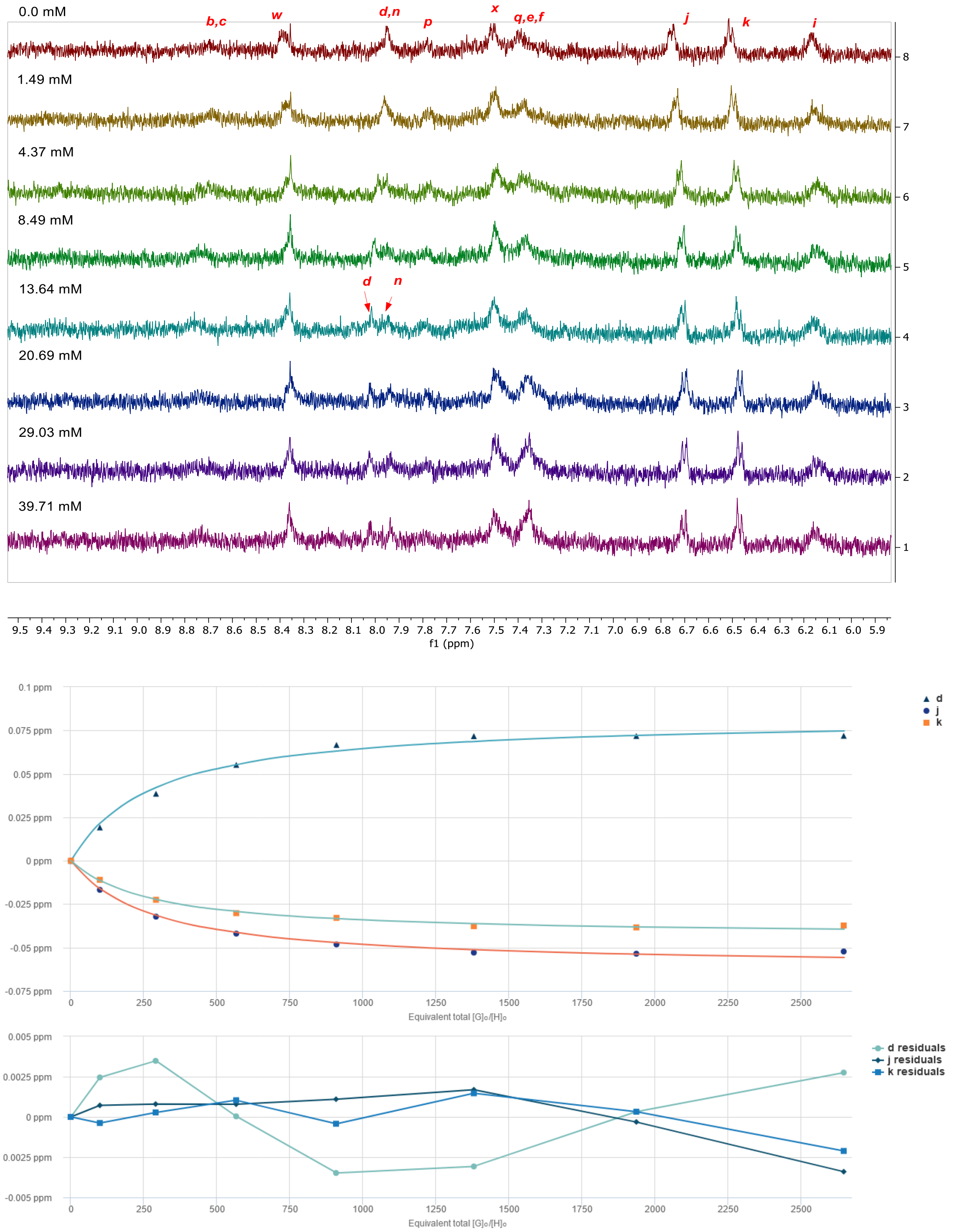

Figure S83. ^{1}H NMR spectra (500 MHz, 1:9 D_2O/H_2O) for receptor **13** (15 µM) titrated with a combined solution of sodium chloride (150 mM) and receptor **13** (15 µM). The concentrations of the guest added are listed in the graph. Bottom). Global fitting of the binding isotherms (protons d, j and k) from Bindfit to a 1:1 model K_a = 238.6 M^{-1} (± 7.8 %). Full fitted data is available online at: http://app.supramolecular.org/bindfit/view/778fae19-e4ce-497b-a931-4bdaa9f58599

Iodide

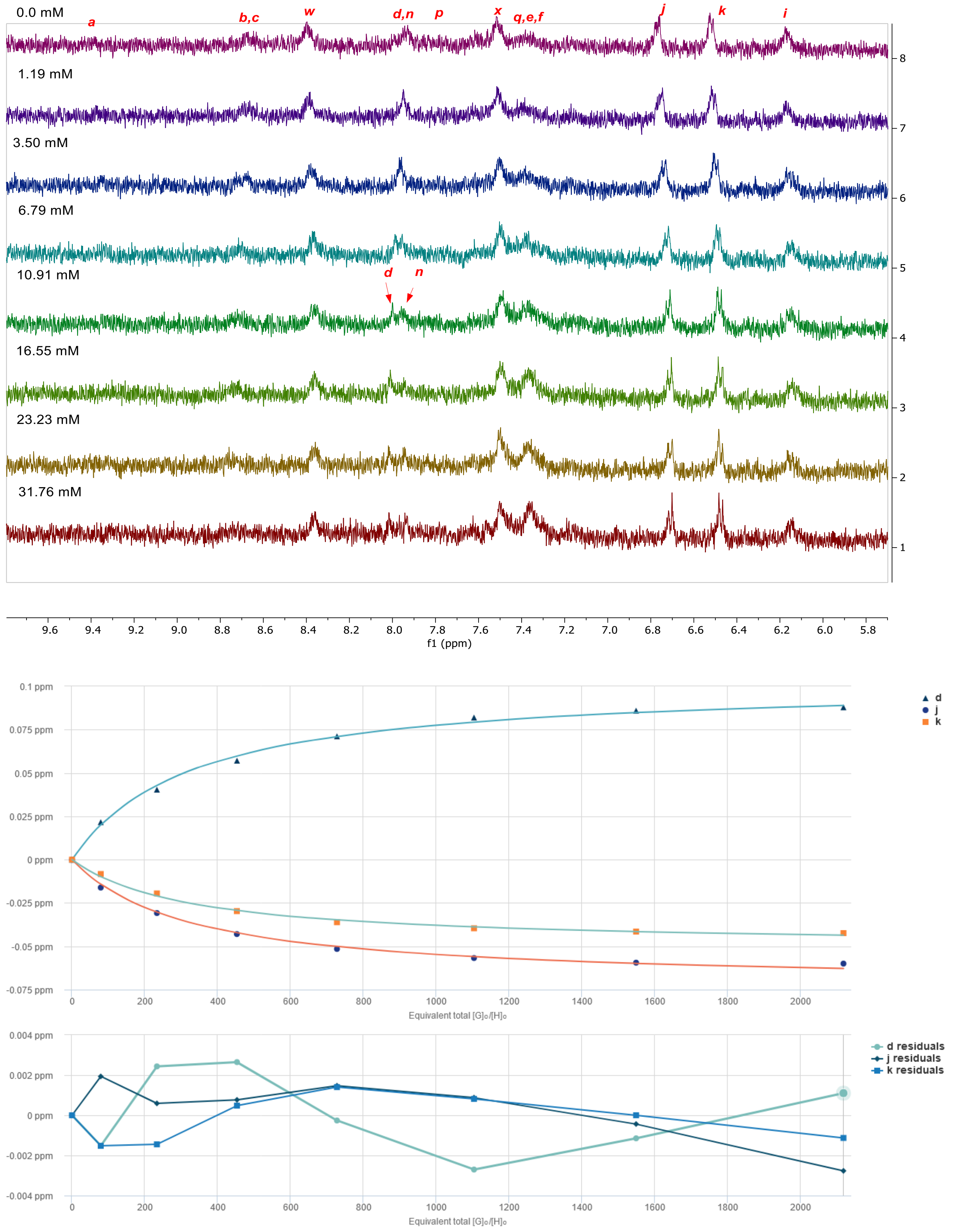

Figure S84. ^{1}H NMR spectra (500 MHz, 1:9 D_2O/H_2O) for receptor **13** (15 µM) titrated with a combined solution of sodium iodide (120 mM) and receptor **13** (15 µM). The concentrations of the guest added are listed in the graph. Bottom). Global fitting of the binding isotherms (protons d, j and k) from Bindfit to a 1:1 model K_a = 205.2 M^{-1} (± 4.9 %). Full fitted data is available online at: http://app.supramolecular.org/bindfit/view/0a9b69be-c4f6-45cc-ab6c-e718477b2b9e

Sulphate

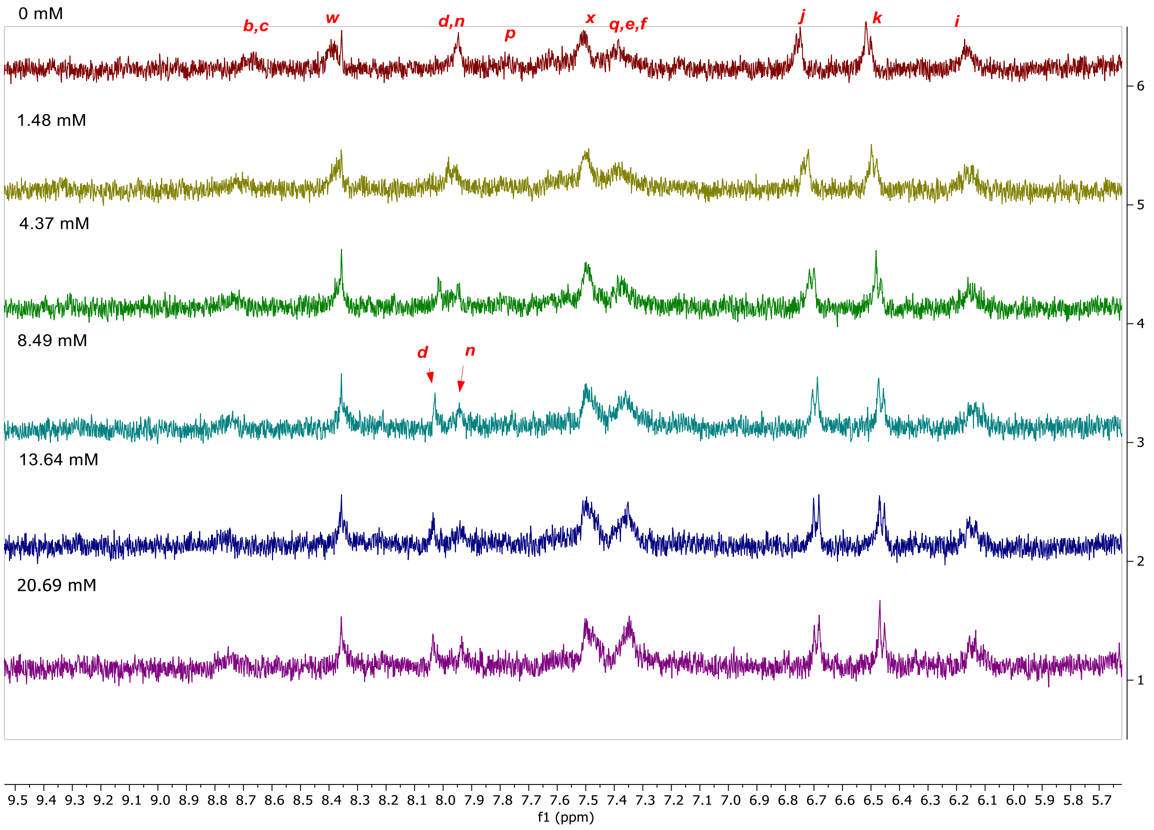

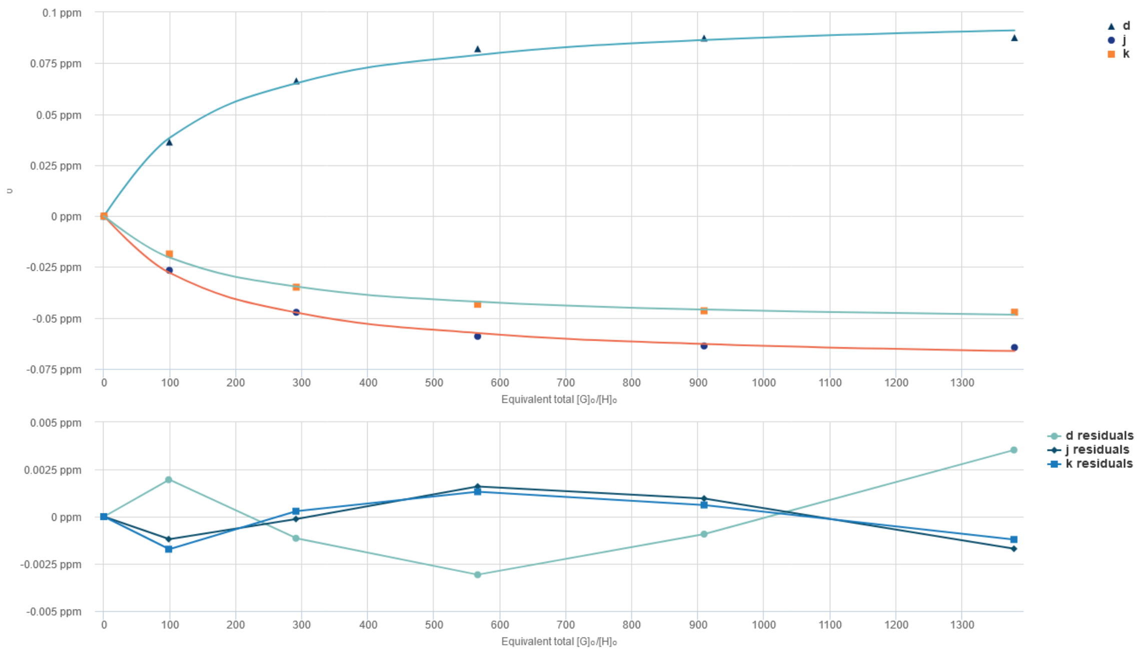

Figure S85. ^{1}H NMR spectra (500 MHz, 1:9 D_2O/H_2O) for receptor **13** (15 µM) titrated with a combined solution of sodium sulphate (150 mM) and receptor **13** (15 µM). The concentrations of the guest added are listed in the graph. Bottom). Global fitting of the binding isotherms (protons d, j and k) from Bindfit to a 1:1 model K_a = 405.7 M^{-1} (± 7.0 %). Full fitted data is available online at: http://app.supramolecular.org/bindfit/view/770a8ea3-71dc-475b-9c86-7e90098a6553

2.2 Fluorescence titrations

Fluorescence titrations were performed on a Horiba Fluoromax spectrofluorometer in quartz cuvettes (3 mL, 10 mm path length). The temperature was controlled at 298 K by a Julabo F12-ED Refrigerated/Heating Circulator. Slit width for both excitation and emission = 4 nm.

Before each binding experiment, two solutions for titration were prepared using the near-neutral stock solutions (H_2O). The first consisted of 2500 μL of host solution at a concentration below the association threshold. The second solution consisted of 500 μL of host, at the same concentration as the first solution, mixed with guest. Both solutions were made in vials, and the near-neutral pH was confirmed (ranging from 7.30 to 7.65 for different host and host-guest solutions). During the titration, the host solution (500 μL) was transferred to a quartz cuvette and aliquots of host-guest mixture were added precisely using Gilson pipettes. This method holds the receptor concentration constant while the guest concentration was increased, with the pH staying near-neutral. After each addition the cuvette was stirred for 3 minutes, left to settle for 30 seconds before the spectrum was recorded at 298 K. At the end of each titration, the solution in the cuvette was measured again confirming negligible changes (ΔpH < ± 0.1) during the experiment.

Association constants were determined by monitoring the change in fluorescence intensity (F) at the specific wavelength. The ΔF values were analysed to give binding constants K_a using Bindfit,[6] employing the 1:1 binding model for all the hosts and the guests.

2.2.1 Photophysical Properties of receptors

Receptor 11

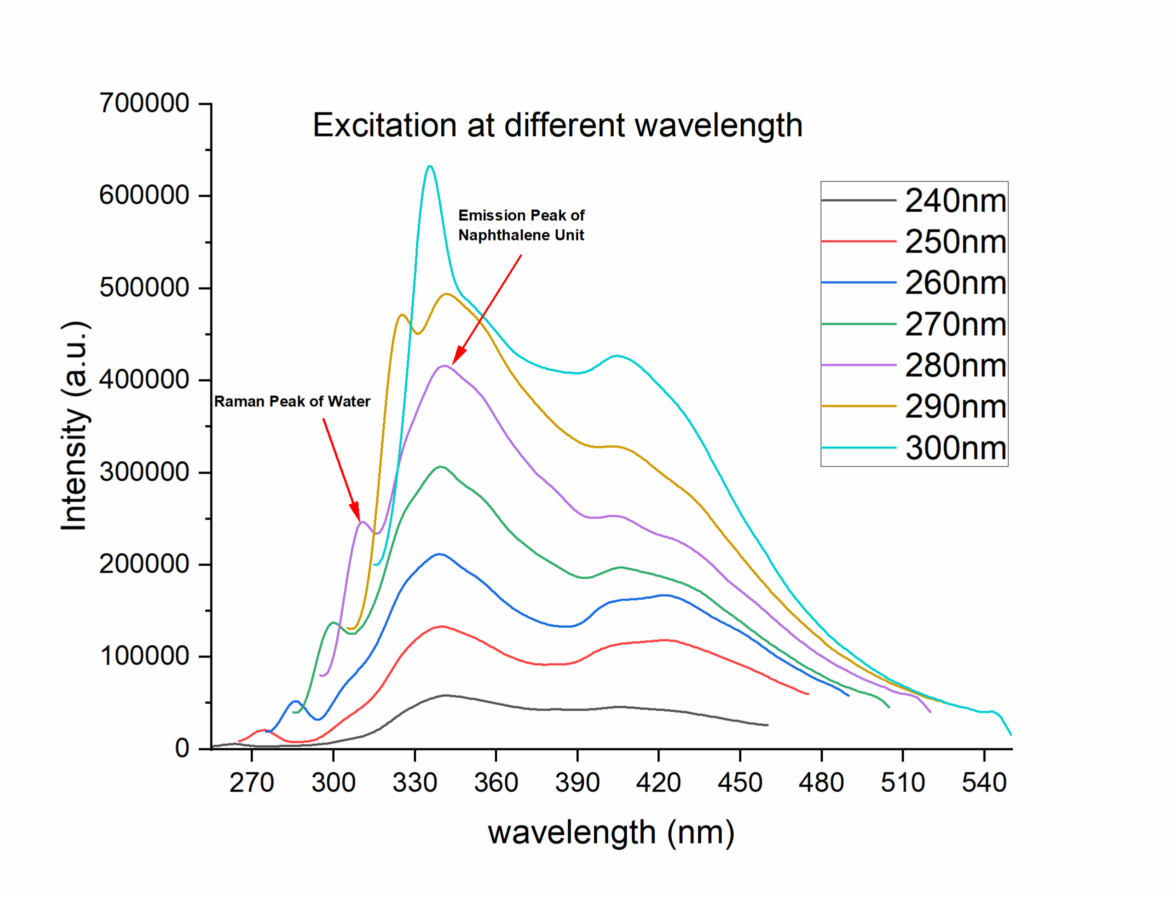

Figure S86. The fluorescence spectra of receptor **11** (10 μM) excited at different wavelengths from 240 nm to 300 nm. Three major peaks from left to right were assigned to water Raman peak, emission peak and excimer peak, respectively.

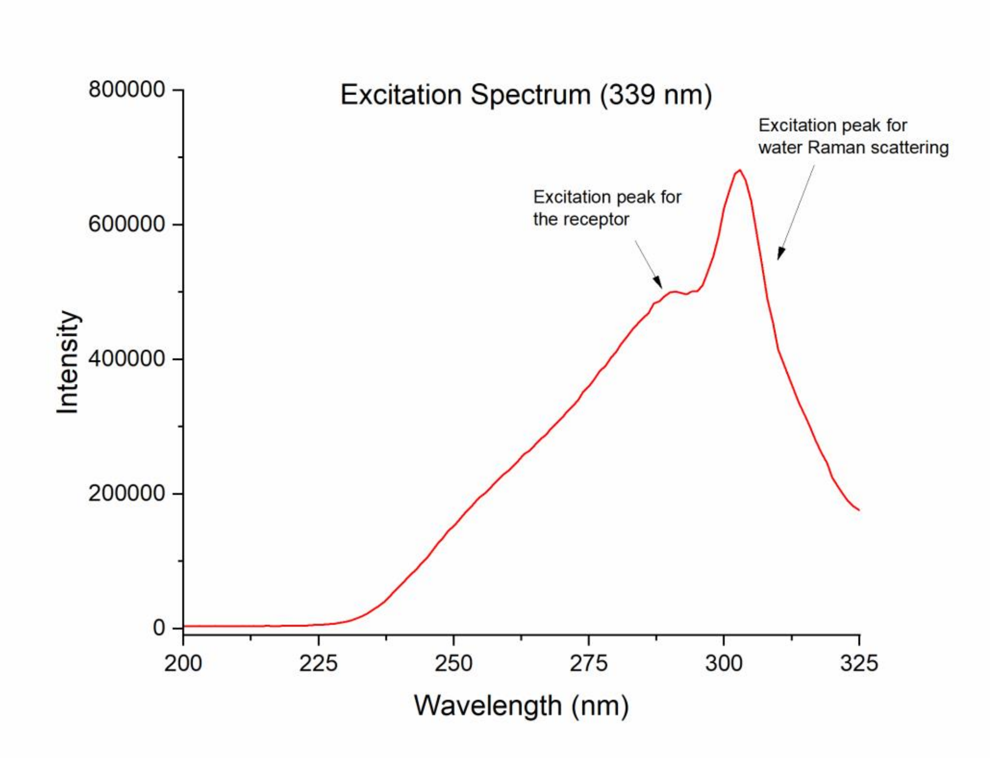

Figure S87. The excitation spectrum (emission measured at 339 nm) of receptor **11** (10 μM).

Receptor 13

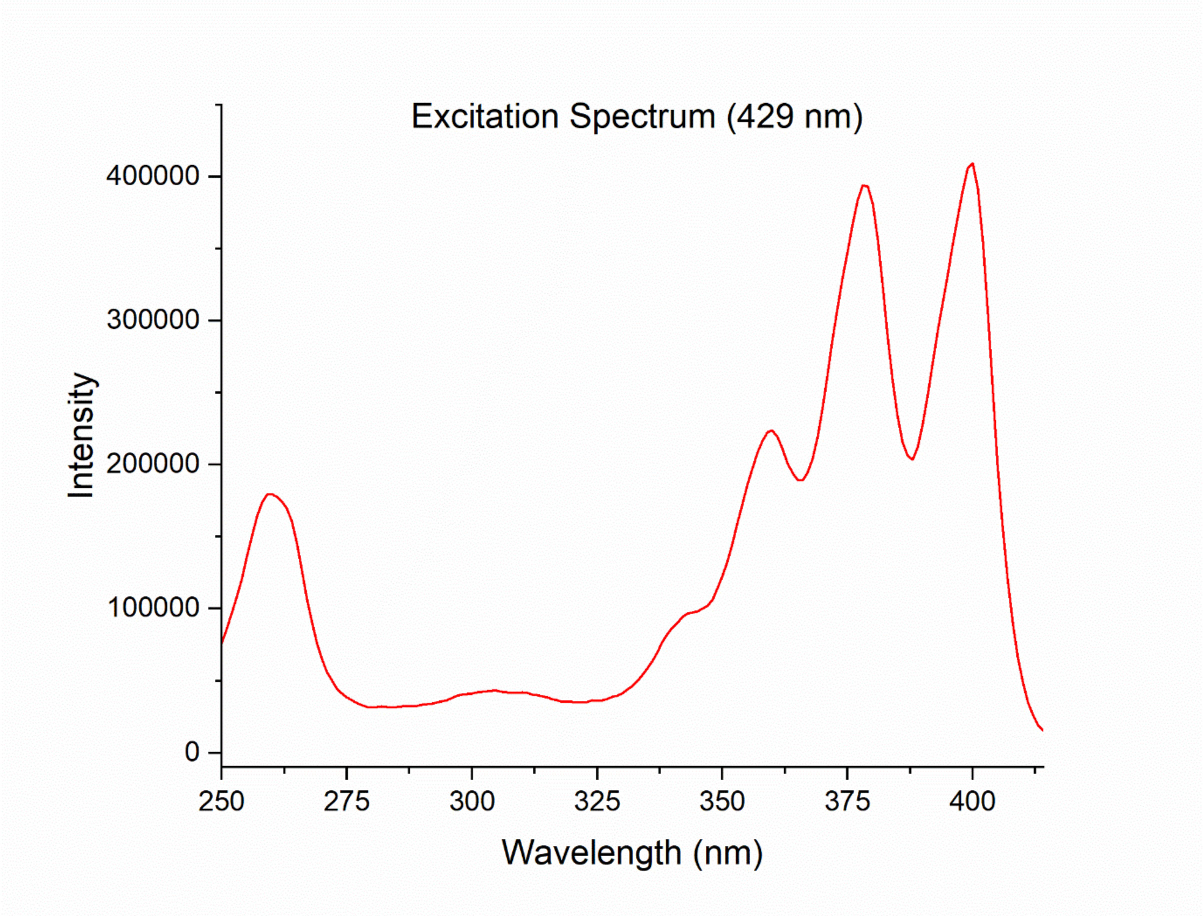

Figure S88. The excitation spectrum (emission measured at 339 nm) of receptor **13** (10 μM).

2.2.2 Fluorescence titrations

Receptor 11 + Acetate

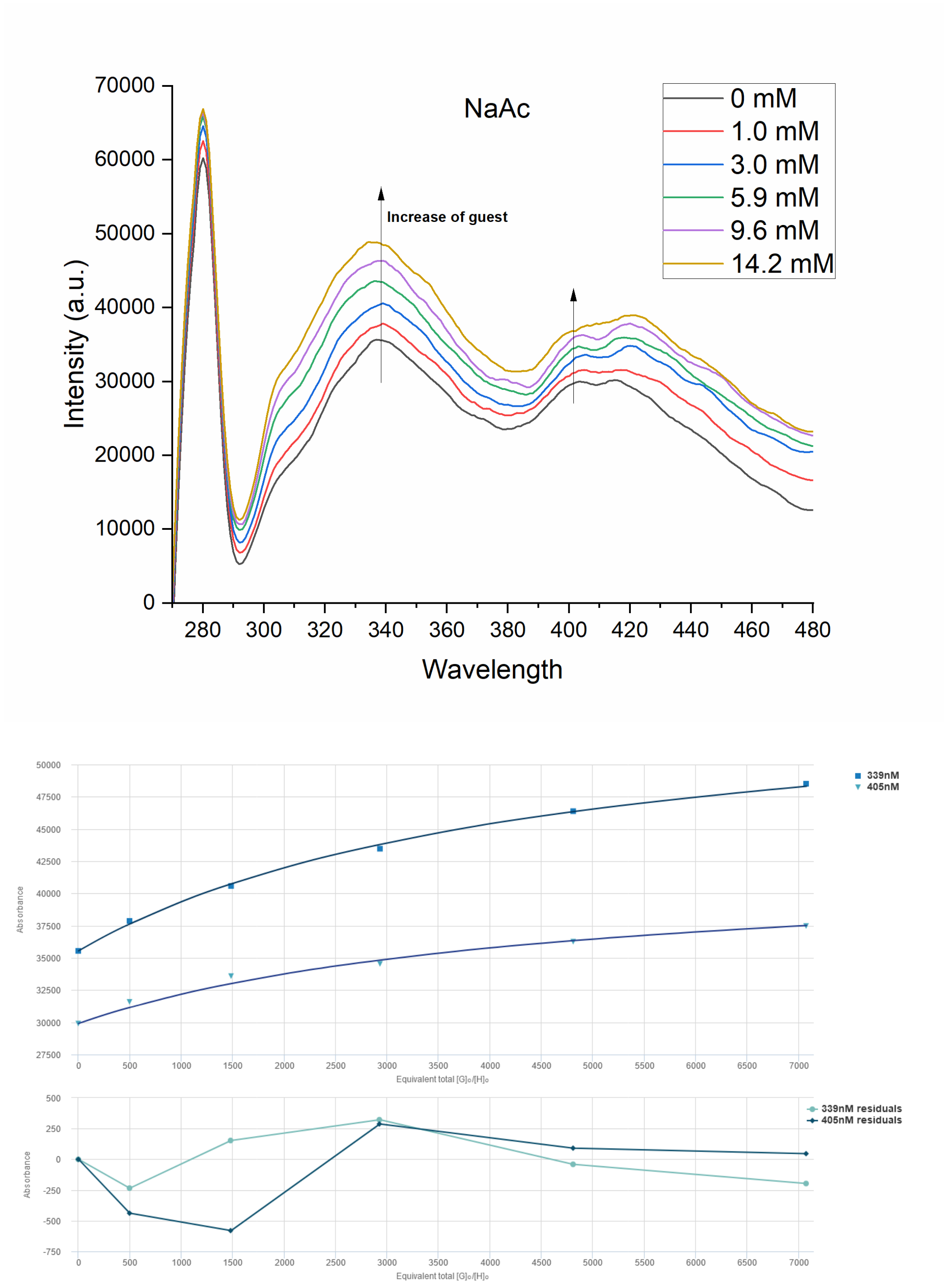

Figure S89. Top). Fluorescence titration of receptor **11** (2 μM) titrated with a combined solution of sodium acetate (250 mM) and receptor **11** (2 μM) in H_2O. (Excitation wavelength = 255 nm) Bottom). Global fitting of the binding isotherms (339 nm and 405 nm) from Bindfit to a 1:1 model K_a = 101.5 M^{-1} (± 3.0 %). Full fitted data is available online at:

http://app.supramolecular.org/bindfit/view/a8b918c2-8c78-4db4-bbbb-6554c2d1b4af

Receptor 11 + Propionate

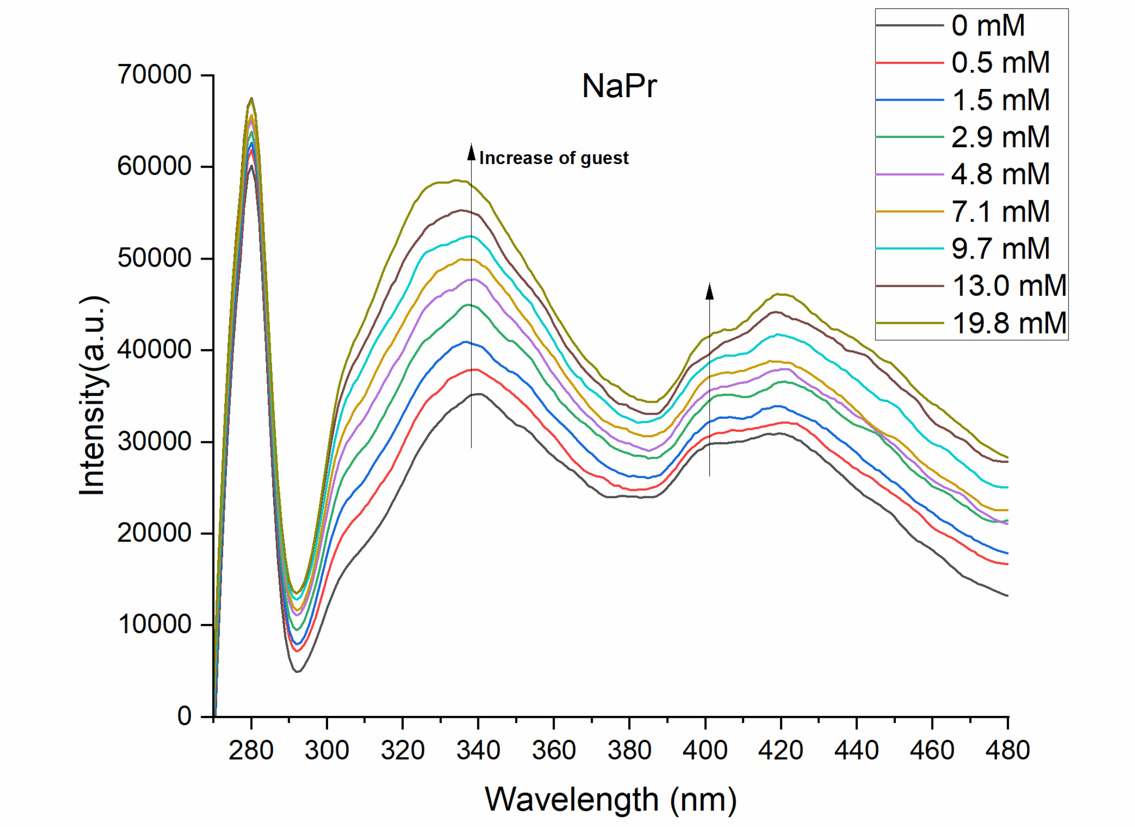

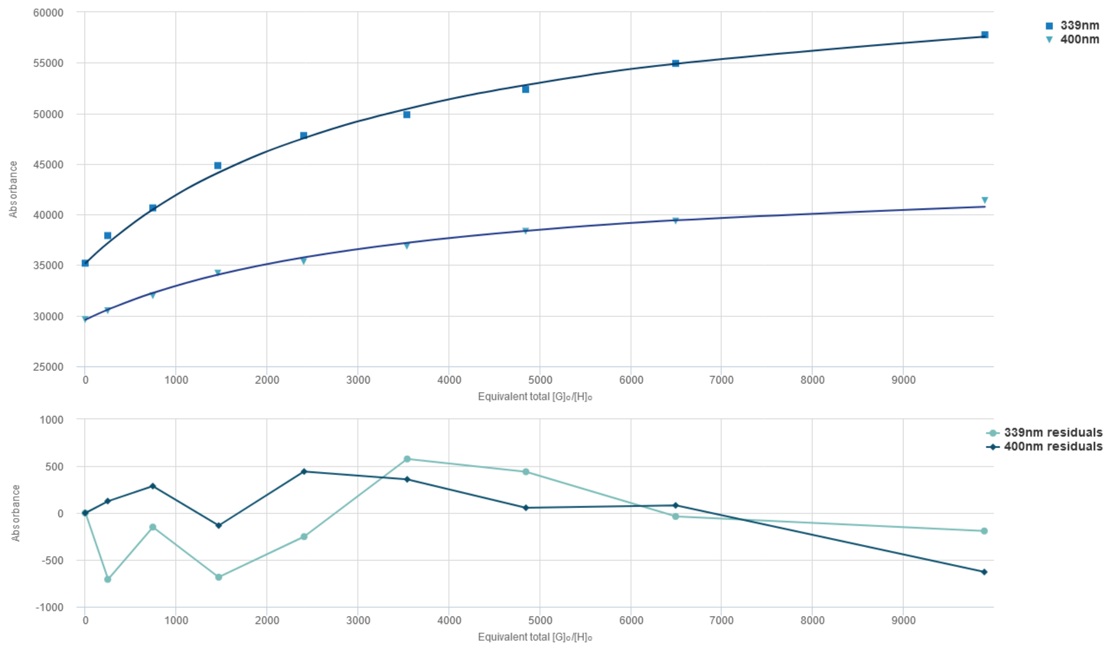

Figure S90. Top). Fluorescence titration of receptor **11** (2 µM) titrated with a combined solution of sodium propionate (125 mM) and receptor **11** (2 µM) in H_2O. (Excitation wavelength = 255 nm) Bottom). Global fitting of the binding isotherms (339 nm and 400 nm) from Bindfit to a 1:1 model K_a = 143.8 M^{-1} (± 4.6 %). Full fitted data is available online at:

http://app.supramolecular.org/bindfit/view/f5cdc2d6-1a2f-4717-8ab3-0776238436b9

Receptor 11 + L-Lactate

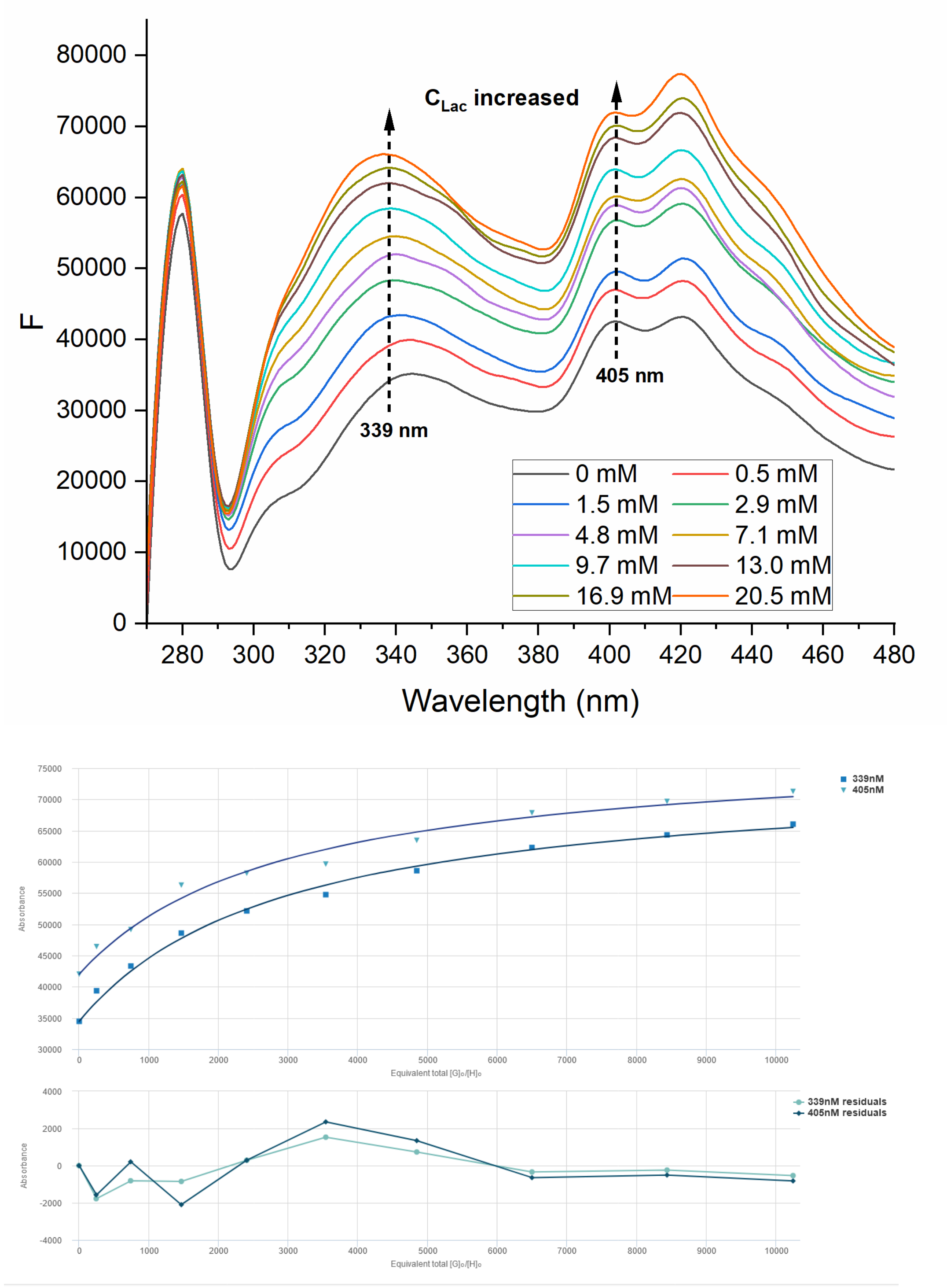

Figure S91. Top). Fluorescence titration of receptor **11** (2 µM) titrated with a combined solution of sodium *L*-lactate (125 mM) and receptor **11** (2 µM) in H_2O. (Excitation wavelength = 255 nm) Bottom). Global fitting of the binding isotherms (339 nm and 405 nm) from Bindfit to a 1:1 model K_a = 170.0 M^{-1} (± 7.8 %). Full fitted data is available online at:

http://app.supramolecular.org/bindfit/view/7778952d-9df8-48ad-b331-c74912a09509

Receptor 11 + Chloride

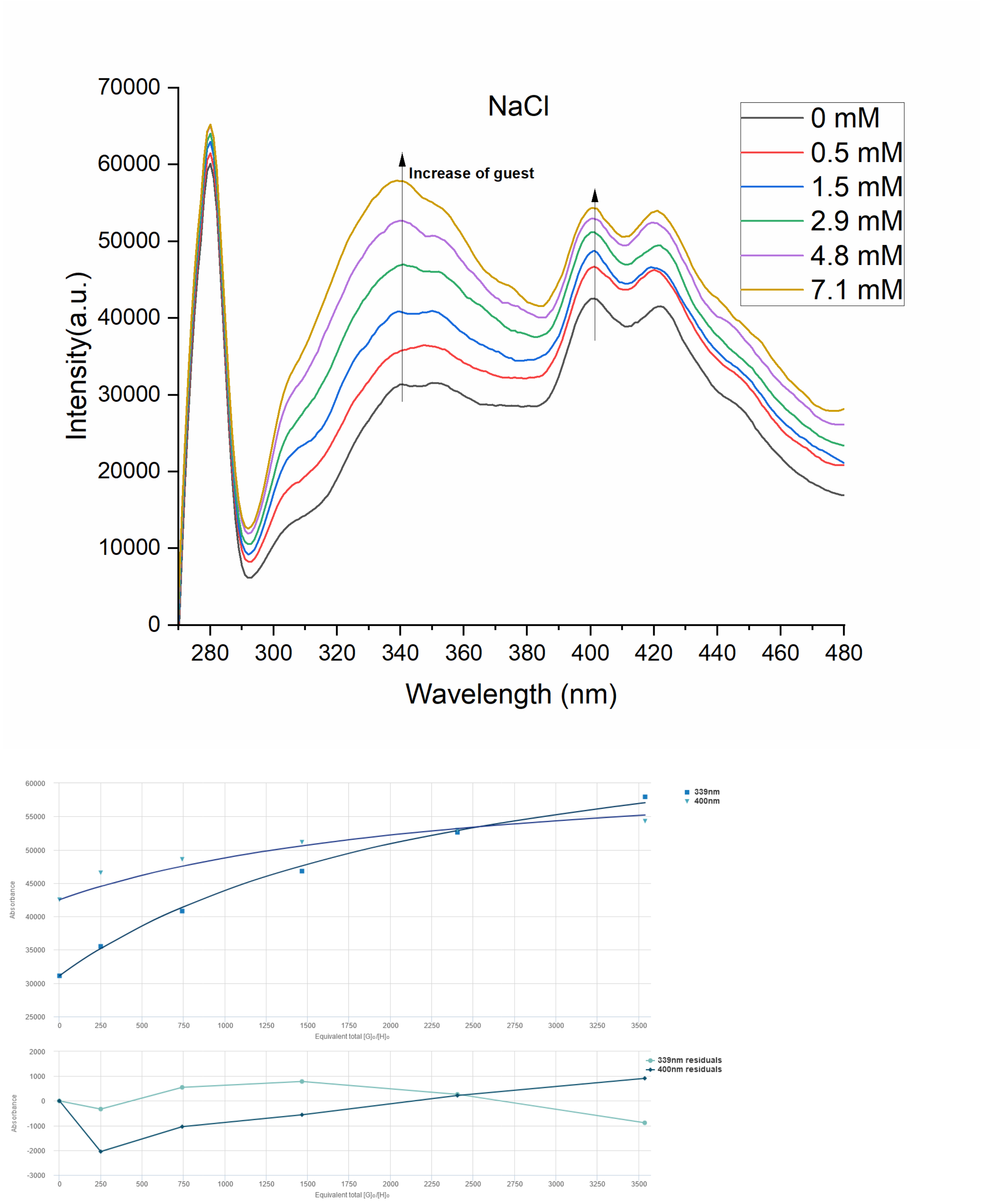

Figure S92. Top). Fluorescence titration of receptor **11** (2 µM) titrated with a combined solution of sodium chloride (125 mM) and receptor **11** (2 µM) in H_2O. (Excitation wavelength = 255 nm) Bottom). Global fitting of the binding isotherms (339 nm and 400 nm) from Bindfit to a 1:1 model K_a = 207.1 M^{-1} (± 10.3 %). Full fitted data is available online at:

http://app.supramolecular.org/bindfit/view/8eb904c4-702a-47f0-990a-9793890f930a

Receptor 13 + L-Lactate

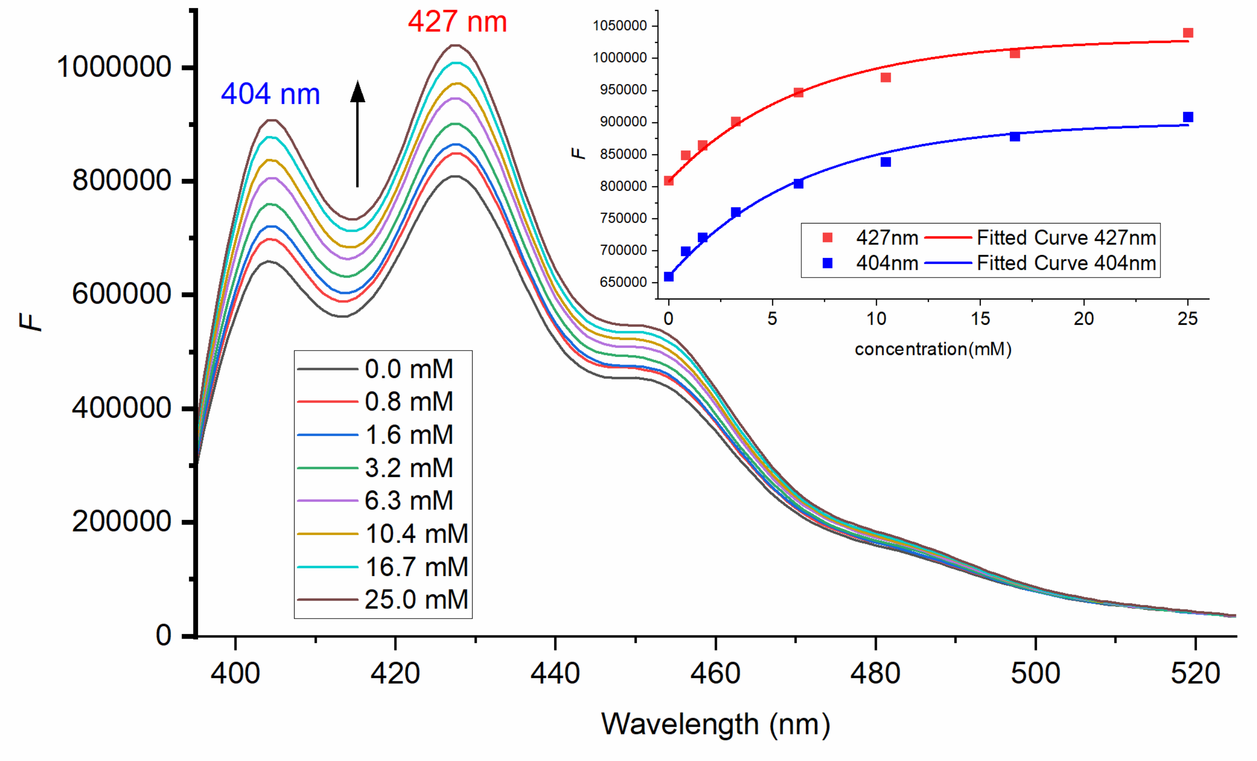

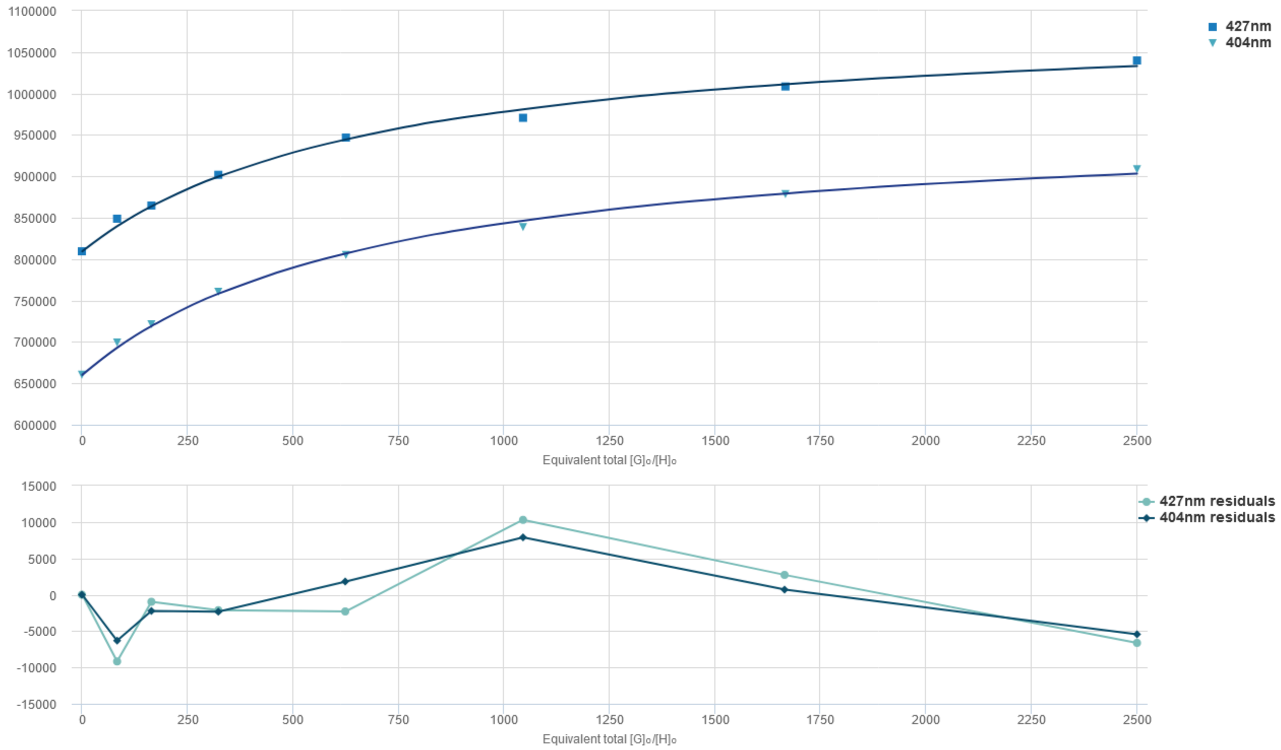

Figure S93. Top). Fluorescence titration of receptor **13** (10 µM) titrated with a combined solution of sodium *L*-Lactate (100 mM) and receptor **13** (10 µM) in H_2O. (Excitation wavelength = 377 nm) Bottom). Global fitting of the binding isotherms (404 nm and 427 nm) from Bindfit to a 1:1 model K_a = 142.7 M^{-1} (± 5.6 %). Full fitted data is available online at:

http://app.supramolecular.org/bindfit/view/62b650f4-2173-412c-874b-3b08fe2380b4

2.3 ITC titrations

Experimental methods: Isothermal Titration MicroCalorimetry (ITC) experiments were performed on a MicroCal iTC200 microcalorimeter. ITC experiments were carried out at 298 K. Host solutions were prepared by dissolving a weighed amount of solid receptors in HPLC-grade water, the pH was checked to be near-neutral (pH = 7.25). For carboxylate guests (acetate and propionate), the guest stock solution was then prepared by dissolving sodium carboxylate salt in water and adjusting pH to match the host solution as closely as possible (pH = 7.20 – 7.35) by adding the conjugate acid. Other guest (NaCl and Na_2SO_4) stock solutions were prepared by dissolving the compound in water and confirming the neutrality of pH. Guest stock solutions were filtered through WhatmanGD/X disposable filter devices, pore size 0.45 µm. Aliquots (typically 1.0 µL) of guest solution were added to the host solution and the evolution of heat was followed as a function of time. Heats of dilution were measured by injecting the same guest solution into HPLC-grade water using identical conditions.

Data processing: ITC outputs for heat of dilution of substrates and the corresponding binding experiments, as well as analysis curves, are included in Figures S92 – 97.

For every addition, the heat of dilution was subtracted from the heat of binding using MicroCal software (MicroCal iTC200 Analysis Add-On Software Package (v7.20) for ORIGIN 7.0). This gave an XY matrix of heat vs. total guest concentration. Obviously bad data was deleted. The data was then analysed using two methods. (1) The matrix was taken to 'One Set of Sites' fitting (N = 1) on MicroCal software to give K_a, ΔG, ΔH and ΔS (Boxes in part *c* of Figures S92 - 97). (2) The matrix was then imported into a specially written Excel programme to fit the data to a 1:1 binding model to give a K_a. ΔG can be derived from K_a and thus ΔS can be derived from ΔH and ΔG using common thermodynamic equations (part *d* of Figures S92 - 97).

The choice of concentrations: Previous experiments demonstrated the difficulties of conducting ITC below the monomeric concentration of this types of receptors. Due to the low host concentration and low binding constants, the subtracted heat change caused by binding was small and overwhelmed by the error of control experiments. In order to generate analysable data, the host concentration chosen in this study was 200 µM which is above the monomeric concentration. At this host concentration, the measurable heat change after subtracting the blank was distinct enough to be plotted with good correlation coefficients (*r*).

Receptor 9 + Chloride

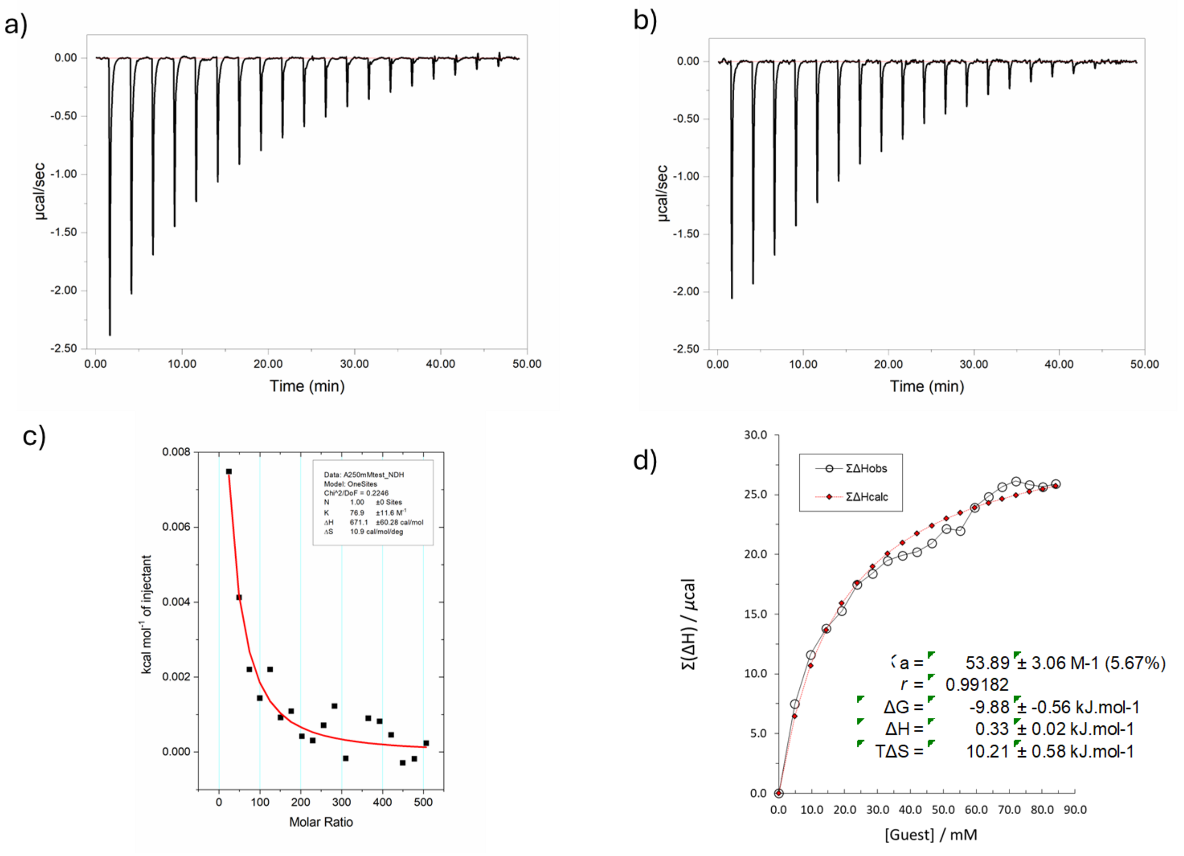

Figure S94. ITC binding results for receptor **9** (200 µM) titrated with sodium chloride (500 mM) in H_2O, in which: a) shows the blank run (addition of substrate into the medium); b) shows the titration (substrate into receptor **9**); c) shows the plotted change in enthalpy vs molar ratio. (K_a = 76.9 ± 11.6 M^{-1}) fitted by Microcal Software; d) shows the fit calculated using an Excel spreadsheet (K_a = 53.9 ± 3.1 M^{-1}).

Receptor 9 + Acetate

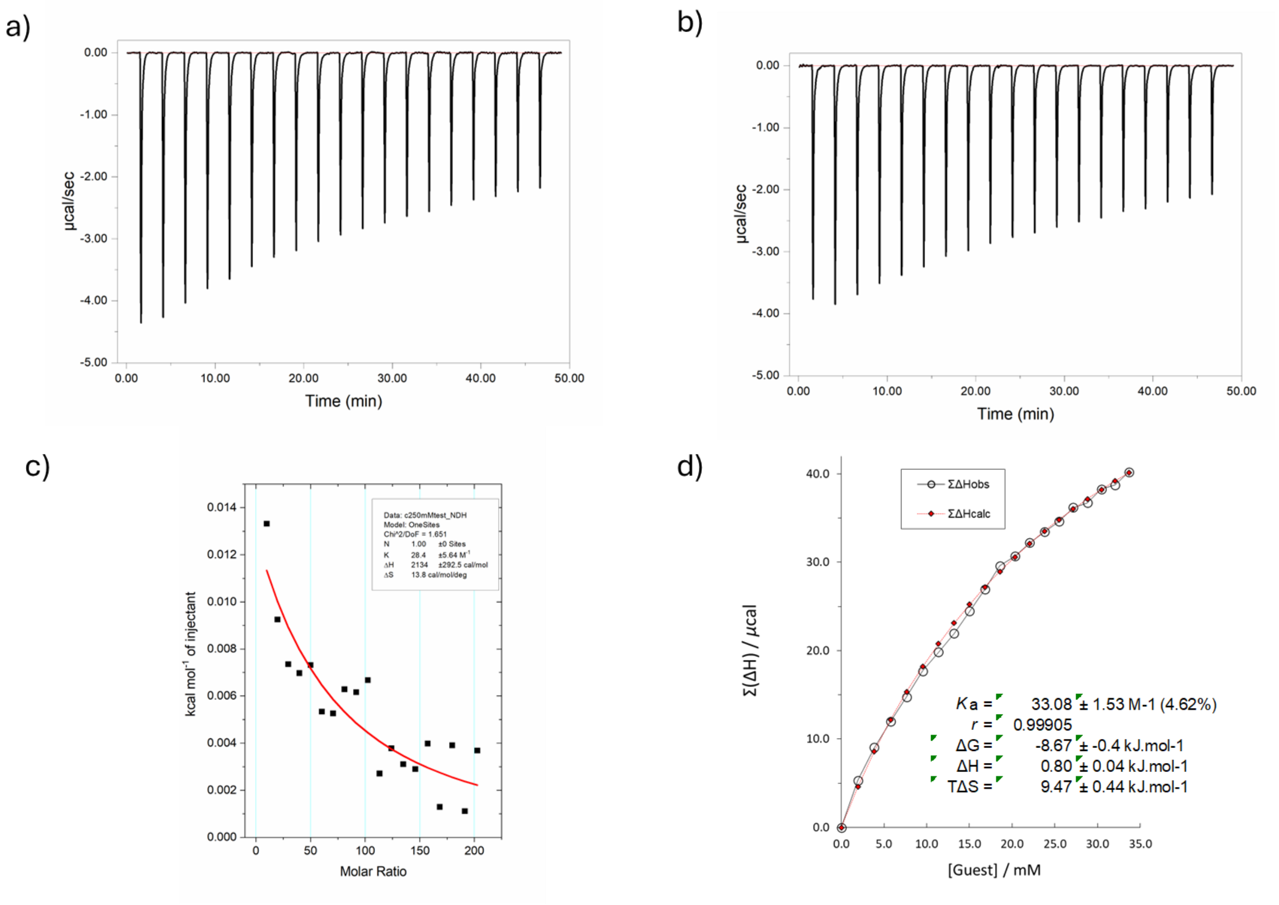

Figure S95. ITC binding results for receptor **9** (200 µM) titrated with sodium acetate (200 mM) in H_2O, in which: a) shows the blank run (addition of substrate into the medium); b) shows the titration (substrate into receptor **9**); c) shows the plotted change in enthalpy vs molar ratio. (K_a = 28.4 ± 5.6 M^{-1}) fitted by Microcal Software; d) shows the fit calculated using an Excel spreadsheet (K_a = 33.1 ± 1.5 M^{-1}).

Receptor 9 + n-Butyrate

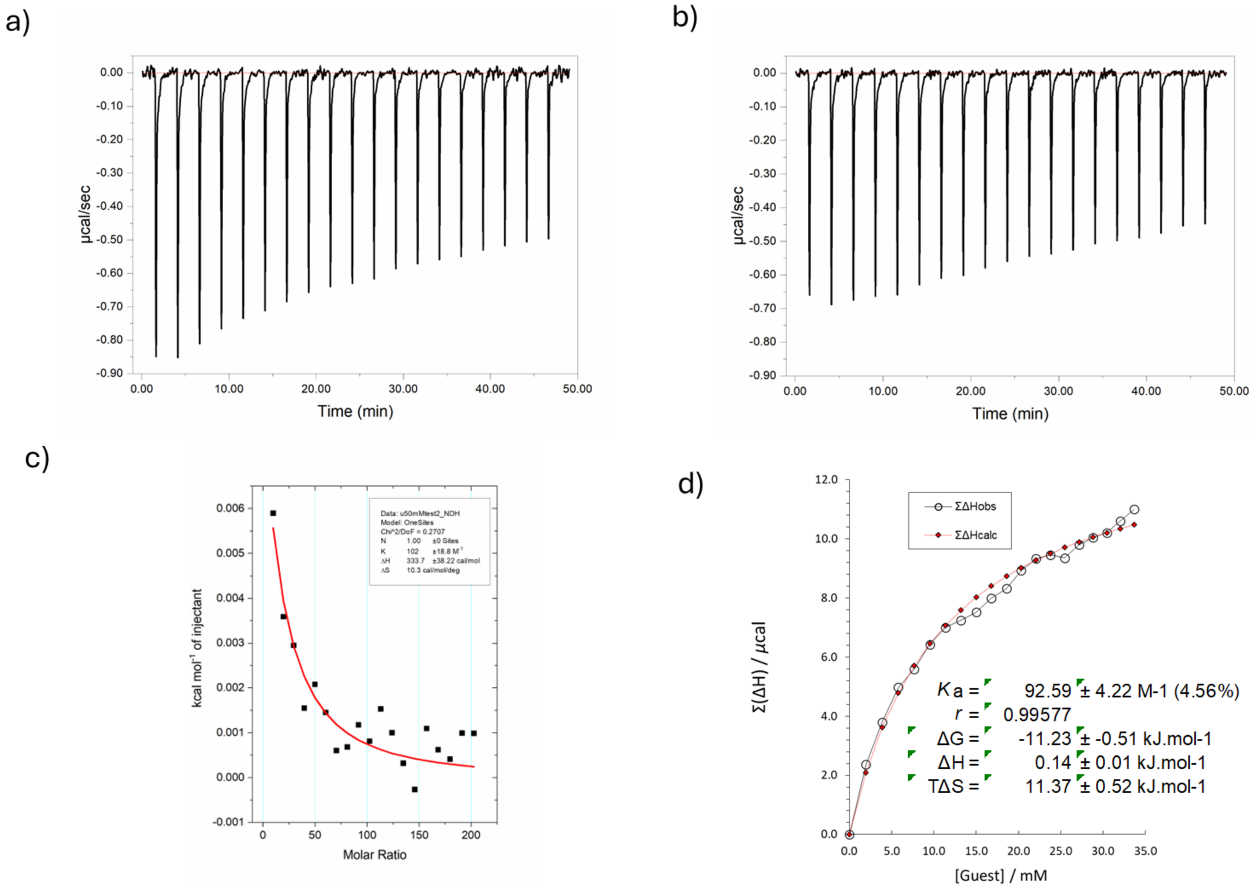

Figure S96. ITC binding results for receptor **9** (200 μM) titrated with sodium n-butyrate (200 mM) in H_2O, in which: a) shows the blank run (addition of substrate into the medium); b) shows the titration (substrate into receptor **9**); c) shows the plotted change in enthalpy vs molar ratio. (K_a = 102 ± 18.8 M^{-1}) fitted by Microcal Software; d) shows the fit calculated using an Excel spreadsheet (K_a = 92.3 ± 4.2 M^{-1}).

Receptor 11 + Chloride

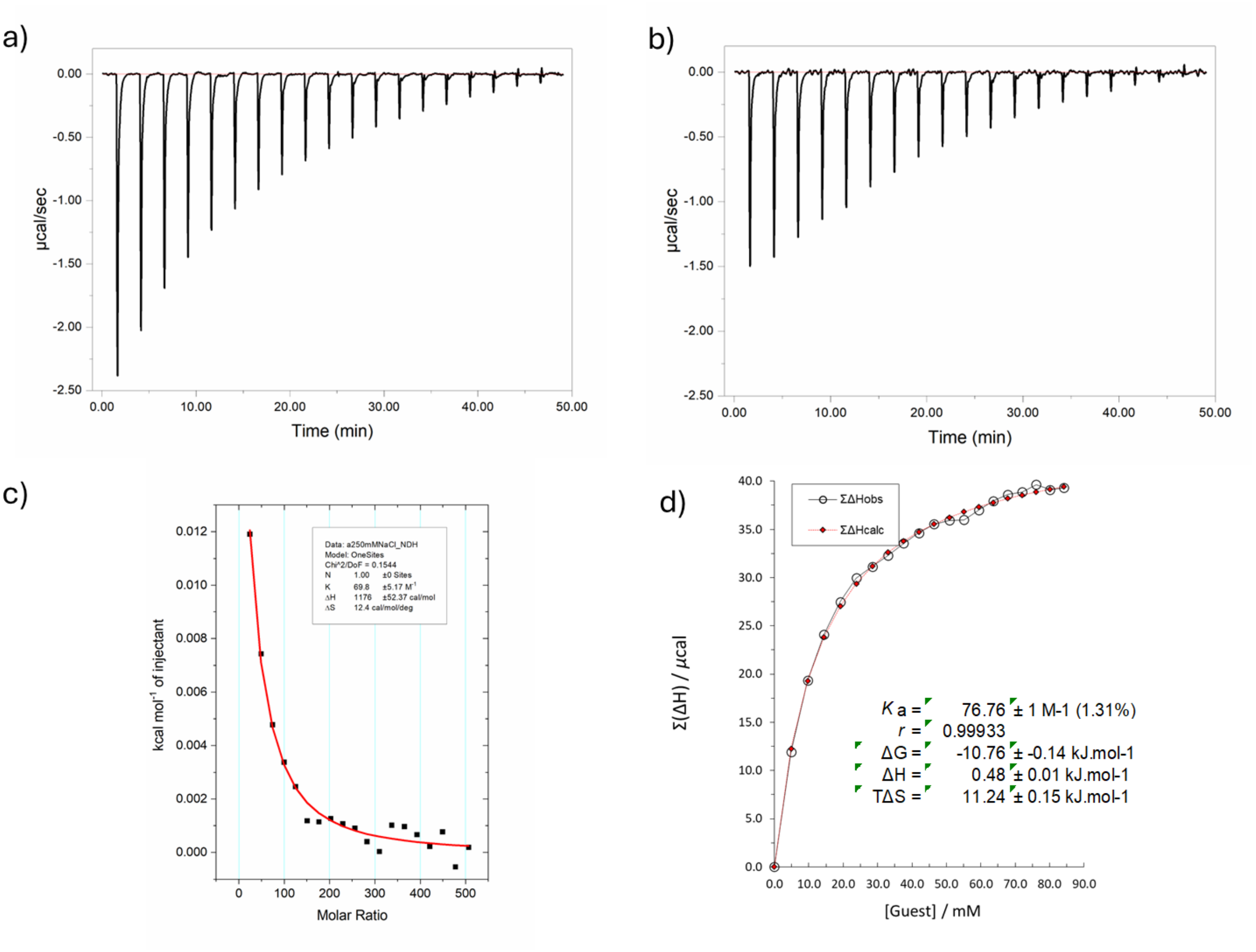

Figure S97. ITC binding results for receptor **11** (200 μM) titrated with sodium chloride (500 mM) in H_2O, in which: a) shows the blank run (addition of substrate into the medium); b) shows the titration (substrate into receptor **11**); c) shows the plotted change in enthalpy vs molar ratio. (K_a = 69.8 ± 5.2 M^{-1}) fitted by Microcal Software; d) shows the fit calculated using an Excel spreadsheet (K_a = 76.8 ± 1.0 M^{-1}).

Receptor 11 + Acetate

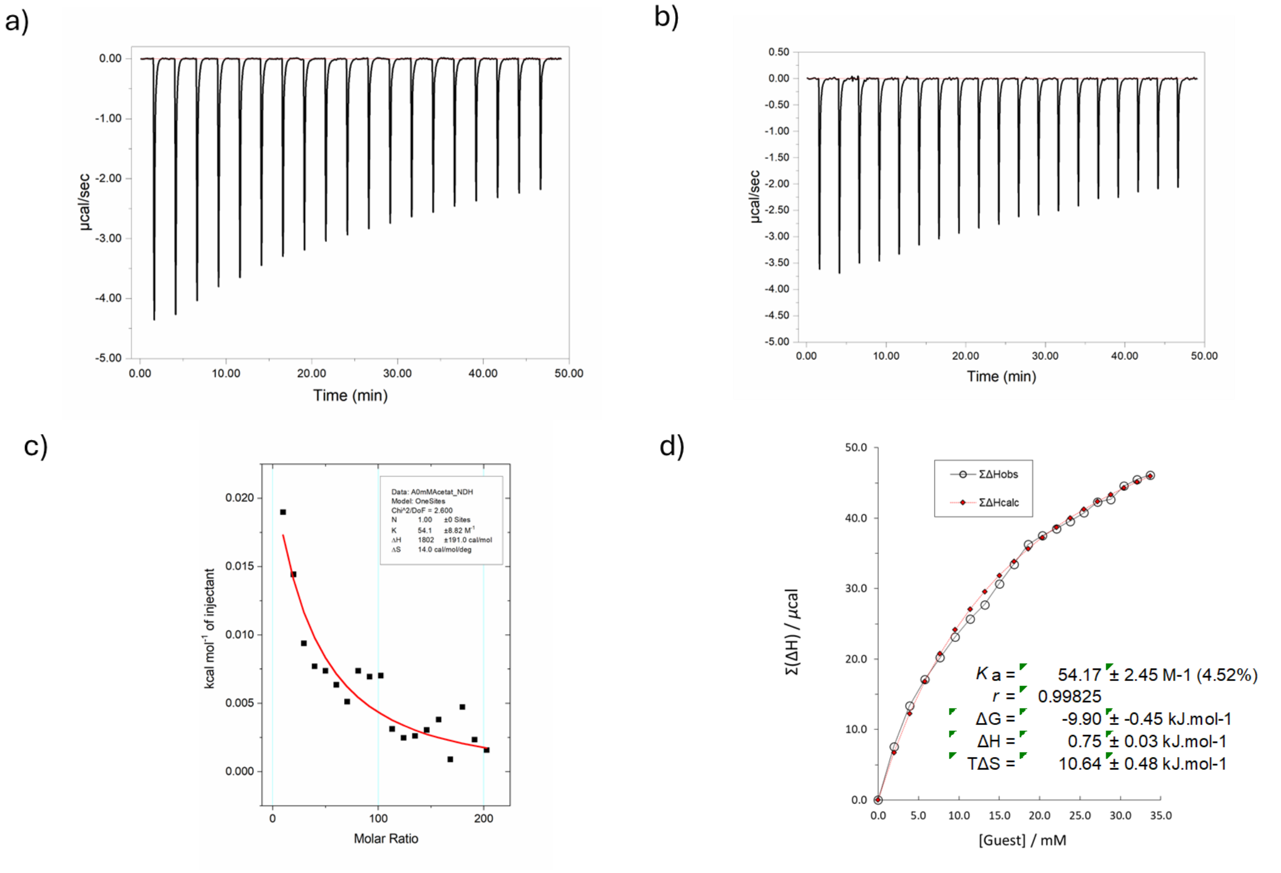

Figure S98. ITC binding results for receptor **11** (200 μM) titrated with sodium acetate (200 mM) in H_2O, in which: a) shows the blank run (addition of substrate into the medium); b) shows the titration (substrate into receptor **11**); c) shows the plotted change in enthalpy vs molar ratio. (K_a = 54.1 ± 8.8 M^{-1}) fitted by Microcal Software; d) shows the fit calculated using an Excel spreadsheet (K_a = 54.2 ± 2.5 M^{-1}).

Receptor 11 + n-Butyrate

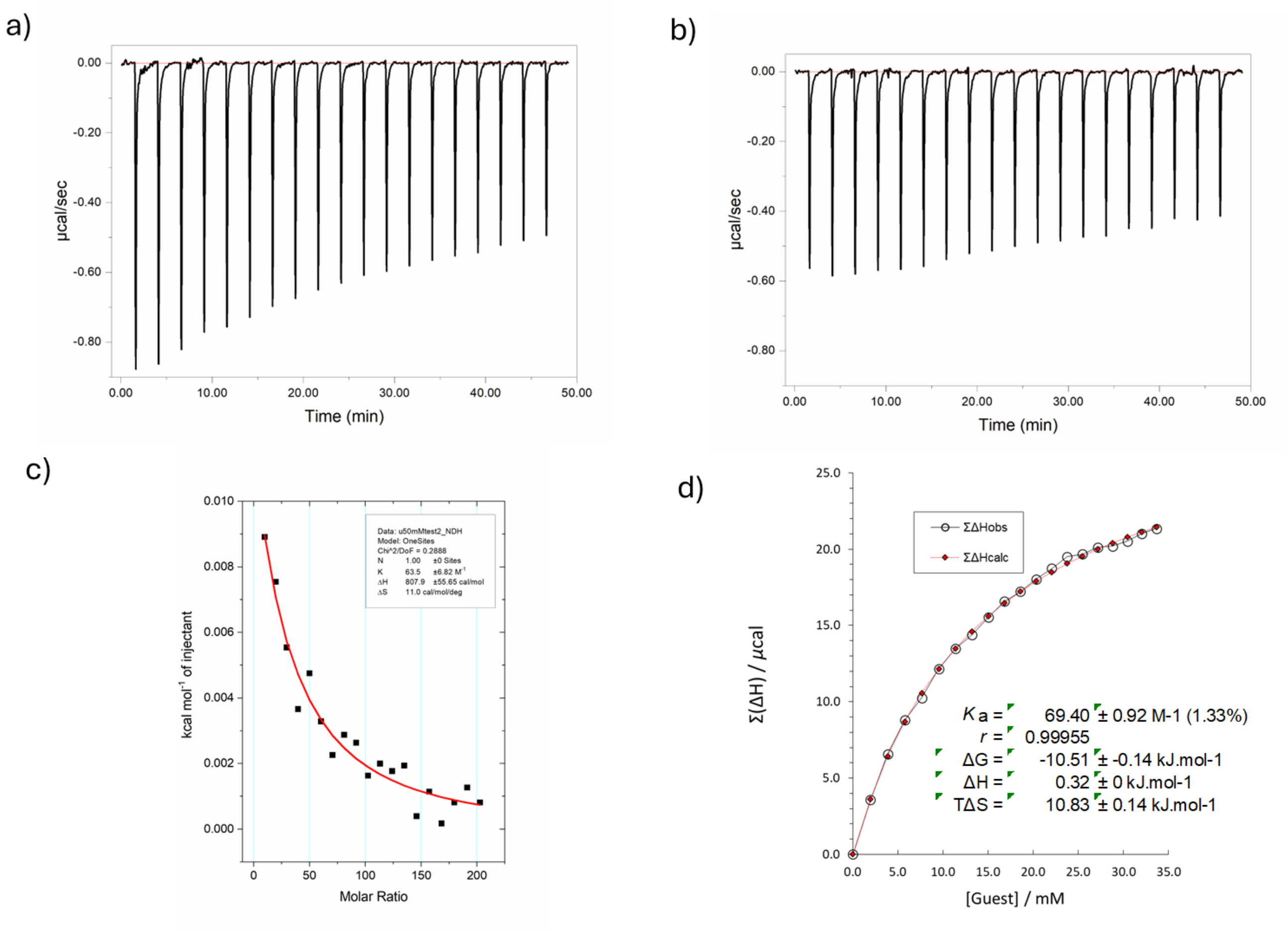

Figure S99. ITC binding results for receptor **11** (200 µM) titrated with sodium *n*-butyrate (200 mM) in H_2O, in which: a) shows the blank run (addition of substrate into the medium); b) shows the titration (substrate into receptor **11**); c) shows the plotted change in enthalpy vs molar ratio. (K_a = 63.5 ± 6.8 M^{-1}) fitted by Microcal Software; d) shows the fit calculated using an Excel spreadsheet (K_a = 69.4 ± 0.9 M^{-1}).

2.4 NMR Structural Studies of Hosts and Complexes

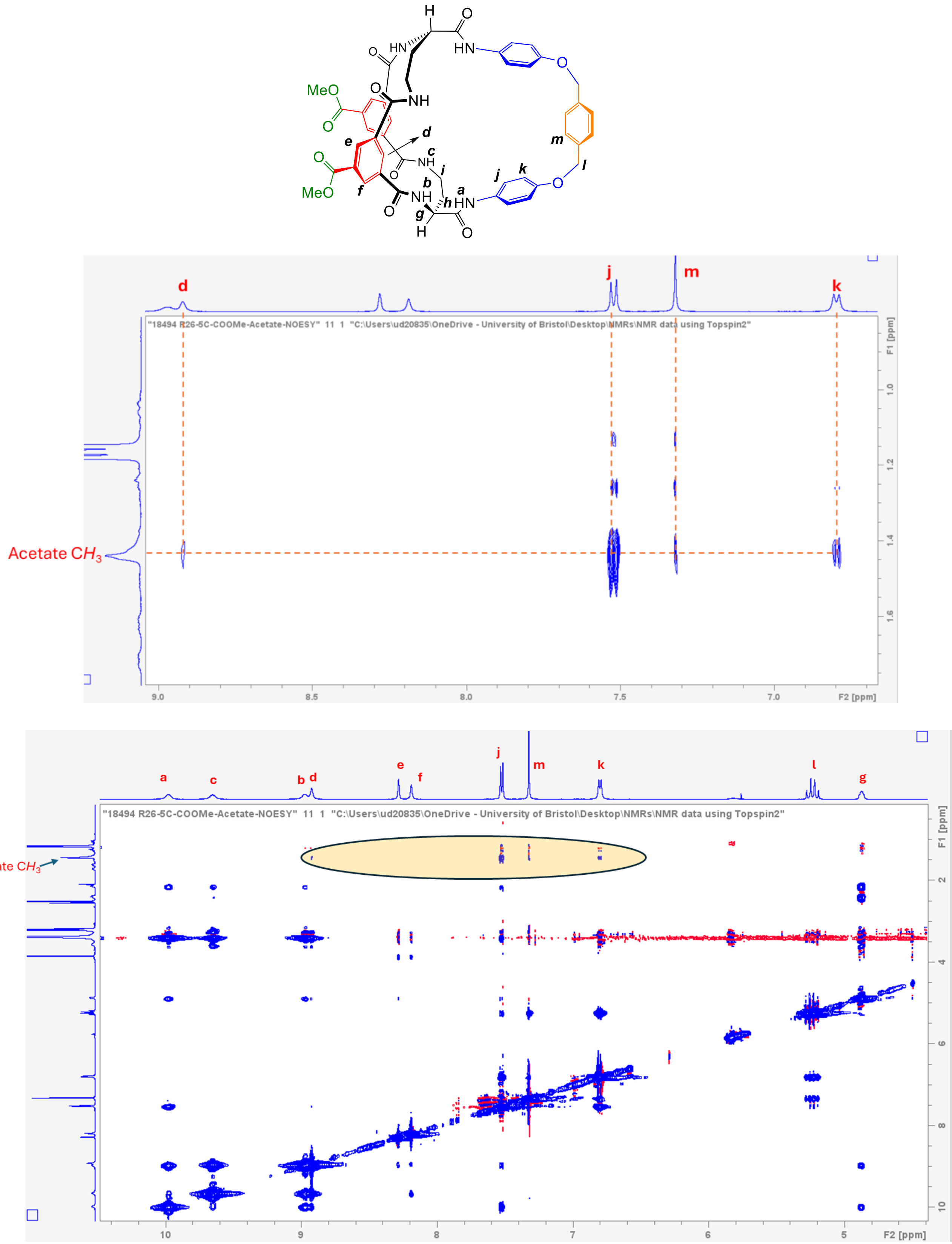

Figure S100. 2D NOESY NMR spectrum (600 MHz, 350 ms mixing time) of **7** (1.5 mM) and TBA Acetate (5 mM) complex in DMSO-d^6.

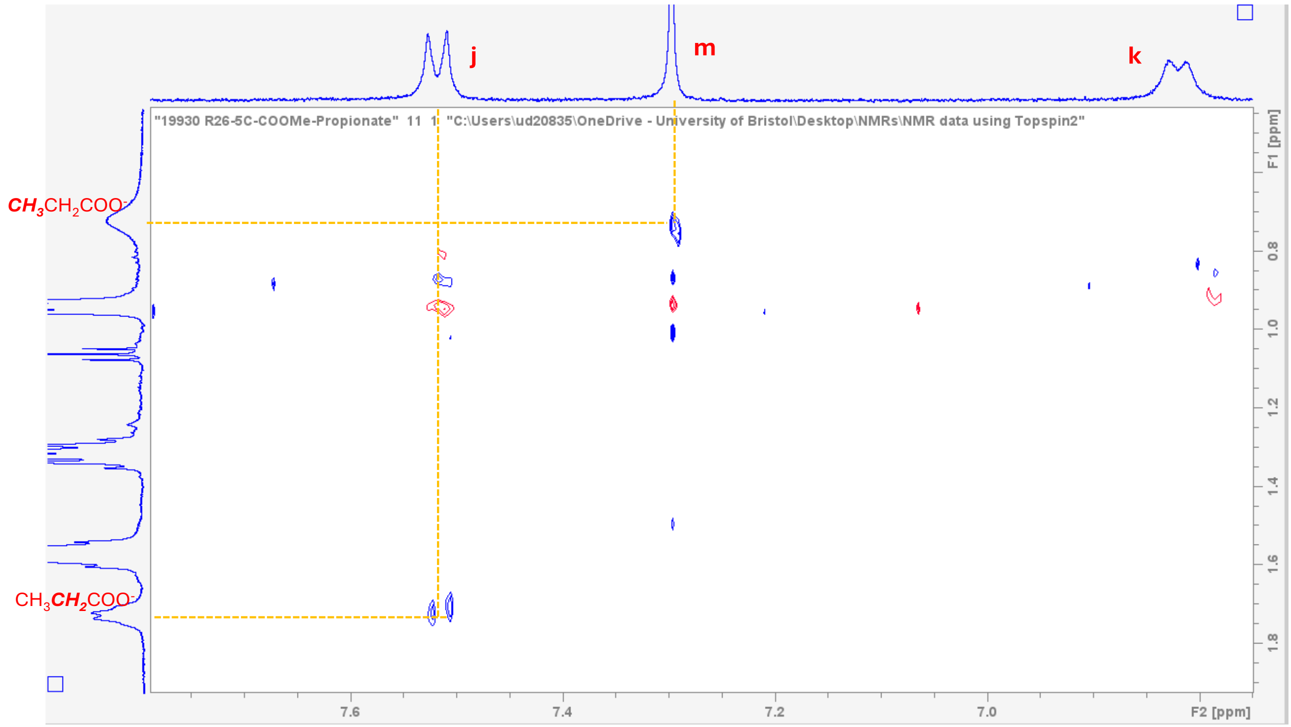

Figure S101. 2D NOESY NMR spectrum (500 MHz, 350 ms mixing time) of receptor **7** (2.5 mM) and TBA propionate (10 mM) complex in DMSO-*d*6.

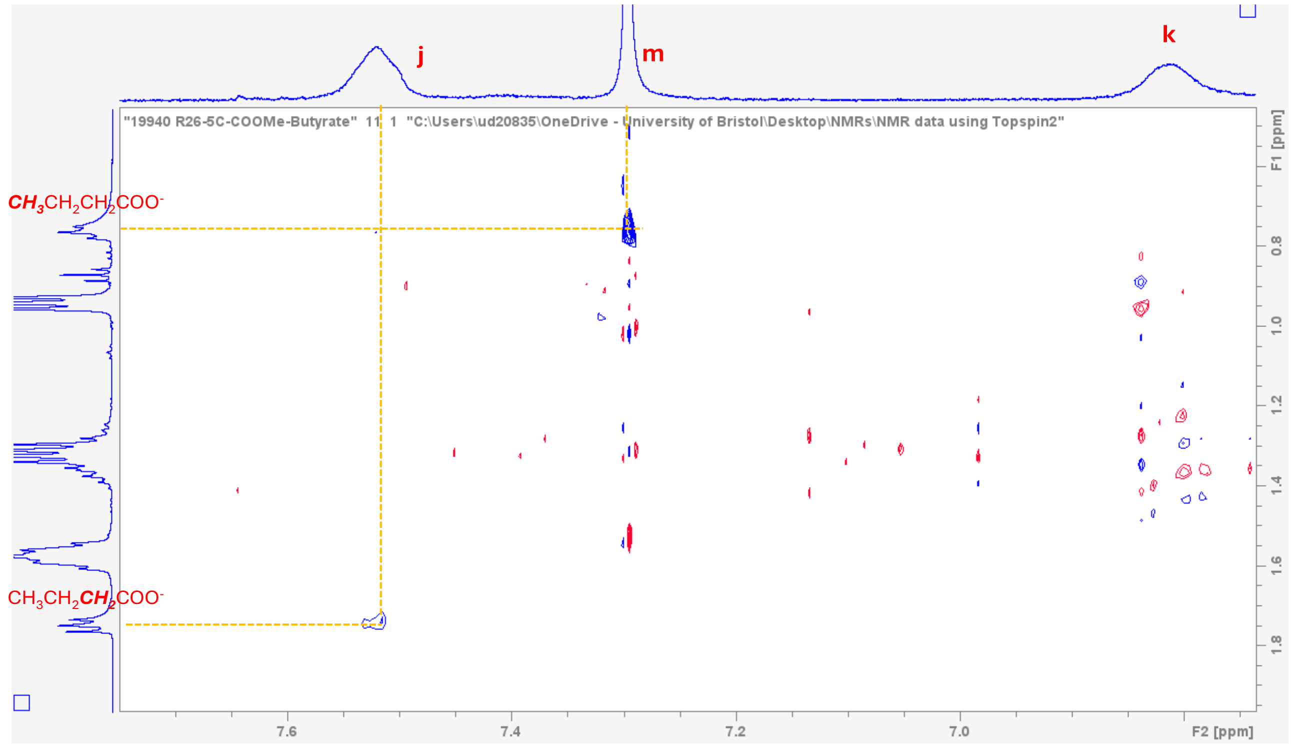

Figure S102. 2D NOESY NMR spectrum (500 MHz, 350 ms mixing time) of receptor **7** (2.5 mM) and TBA butyrate (10 mM) complex in DMSO-*d*6.

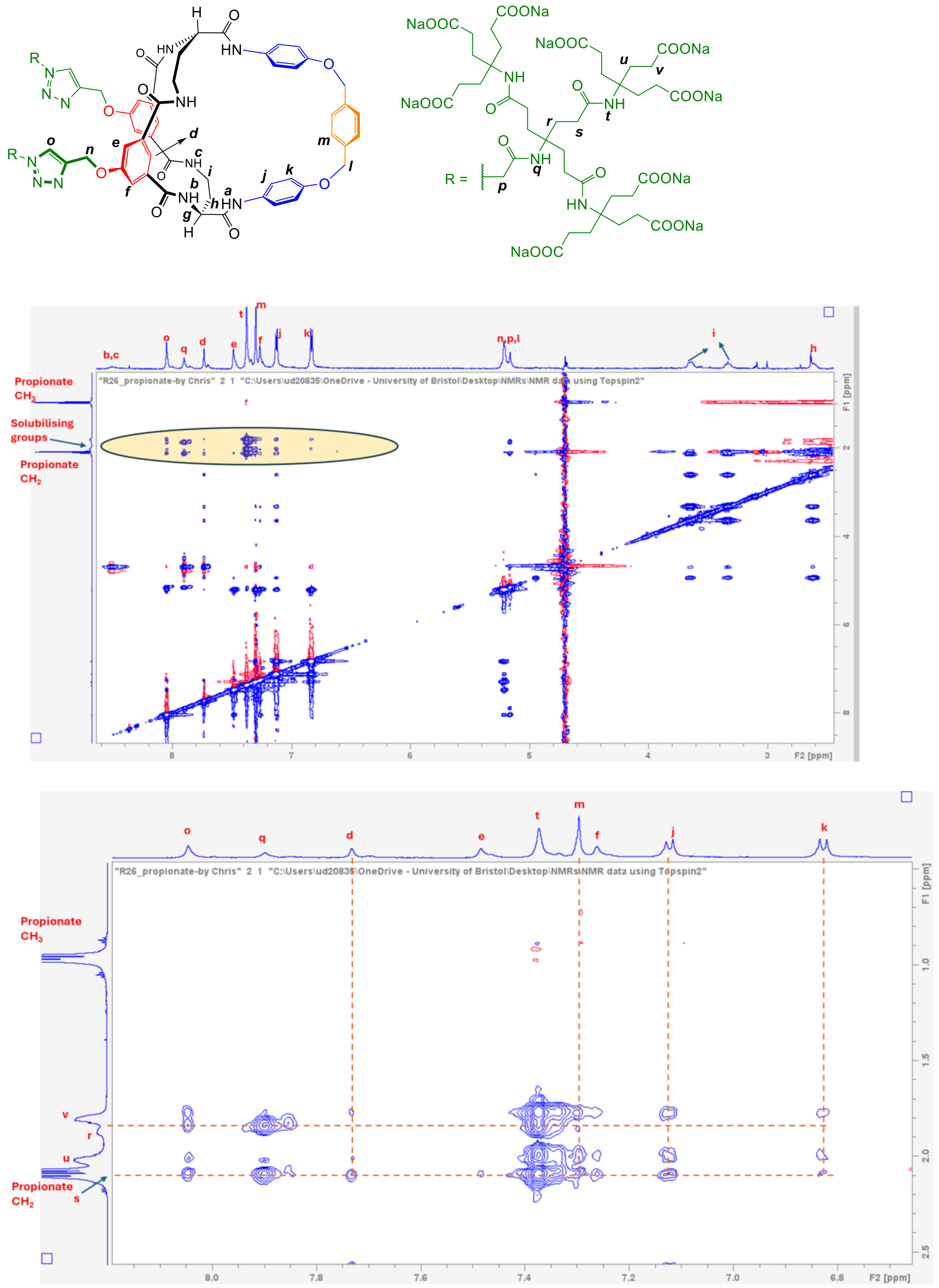

Figure S103. 2D NOESY NMR spectrum (700 MHz, 450 ms mixing time) of receptor **9** (1.0 mM) and sodium propionate (100 mM) in 1:9 D_2O/H_2O. Proton *s* and propionate CH_2 both come at 2.1 ppm. There is a medium strength cross-peak between this chemical shift and proton *j* at 7.12 ppm. However there is no cross peak between *j* and proton *r* (1.85 ppm), so one would not expect a cross-peak for *j*-*s*. We conclude that the cross-peak at *j*-2.1 ppm is to propionate CH_2, supporting the hypothesis that the carboxylate sits within the bicyclic binding site.

3. Molecular Modelling

Modelling studies employed Maestro Version 14.3, with Batchmin V14.7 for energy minimisation. The calculations employed the OPLS4 force field with aqueous GB/SA solvation. Details of Monte Carlo Molecular Mechanics (MCMM) searches are given in the figure captions.

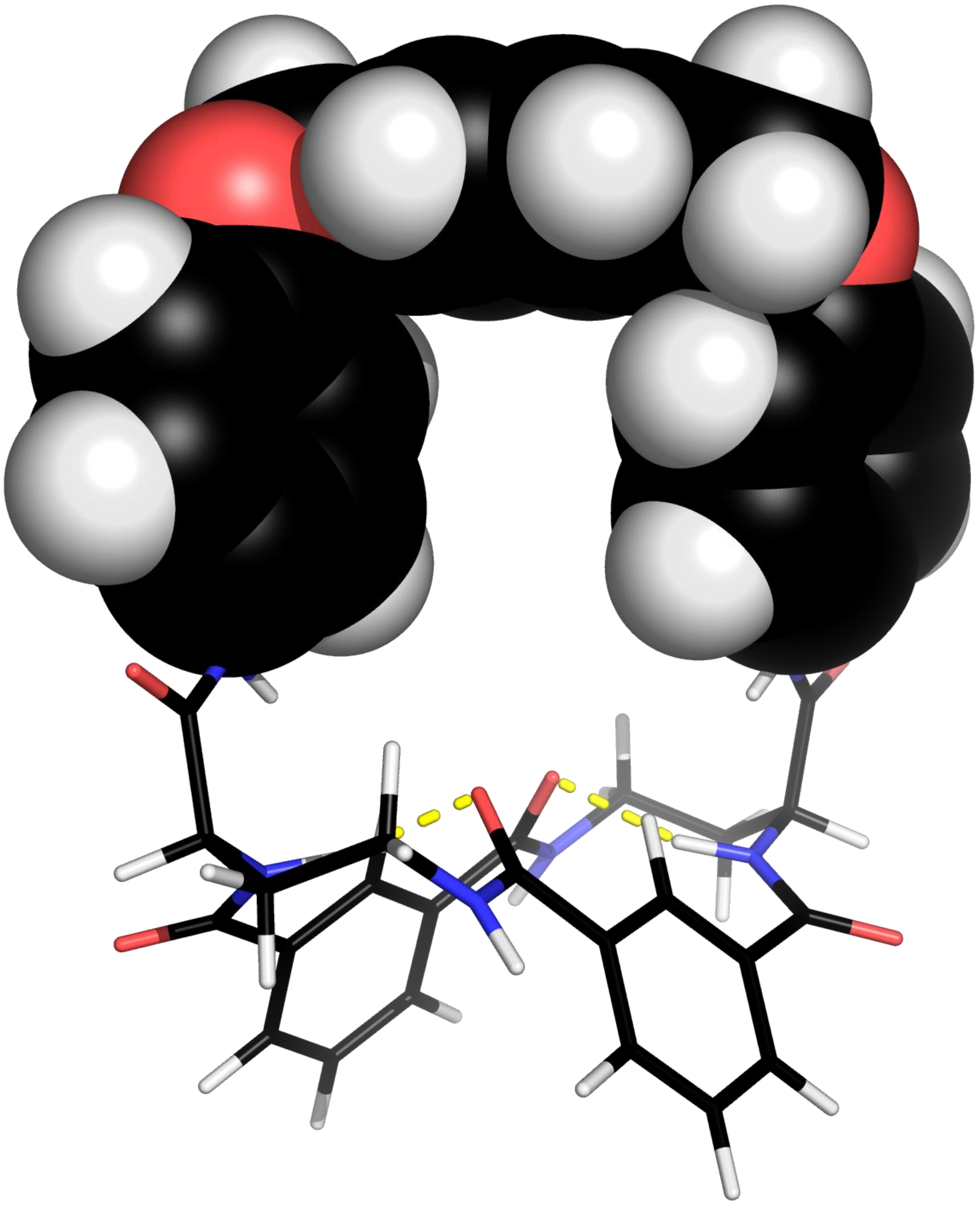

Figure S104. Lowest energy conformation from an MCMM search (5000 steps, aqueous solvation) on the bicyclic core of **7**, **8** and **9** (Fig. 2, external groups X replaced by H). One bond in the tetractam ring and one in the bridge are allowed to break and re-form during the search, so that rotations about the other bonds are able to take place. In the absence of anionic guest, the tetralactam unit forms two intramolecular H-bonds. All conformations found in the search possessed open cavities with hydrophobic clefts.

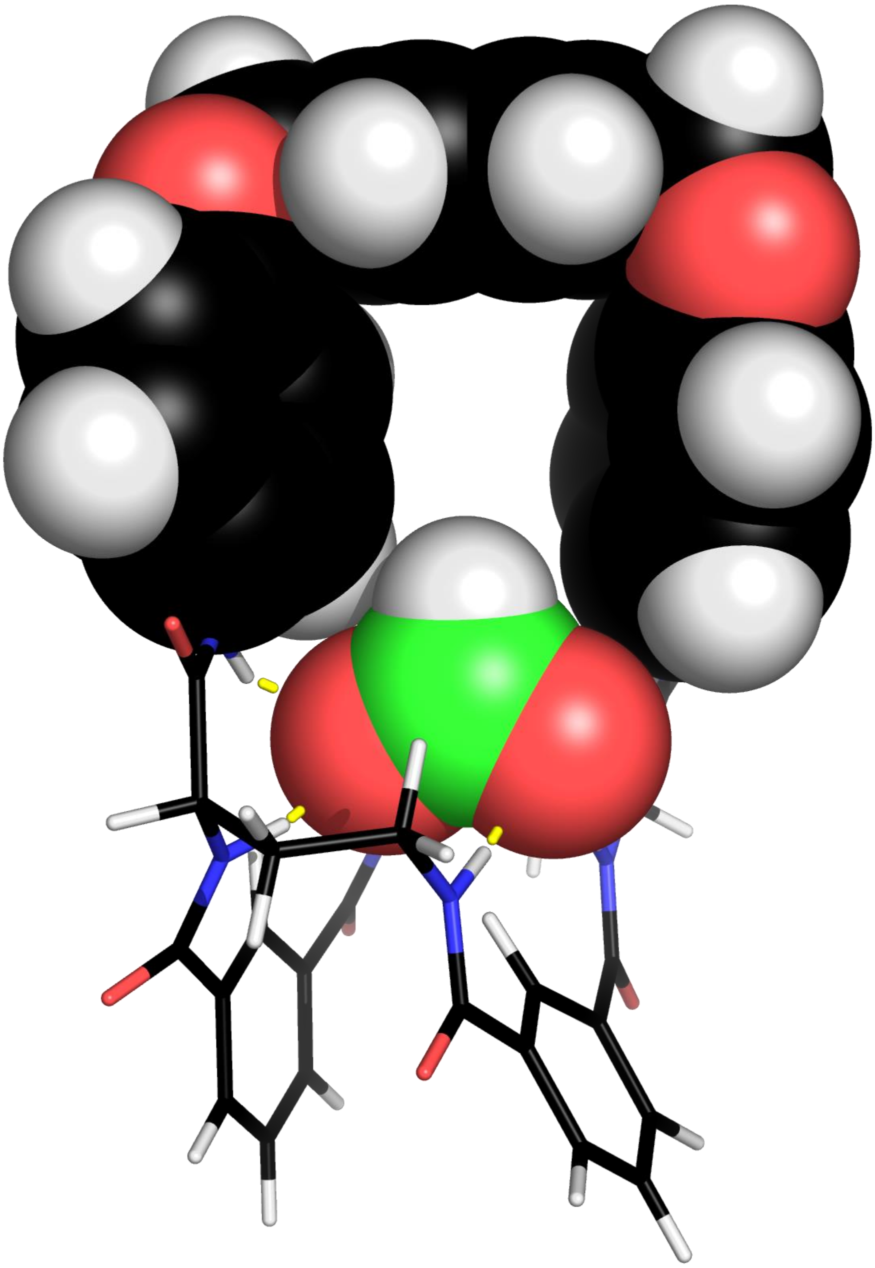

Figure S105. Lowest energy conformation from an MCMM search (1000 steps) on the bicyclic core of **7**, **8** and **9**, performed as for S102 but in the presence of formate anion. The formate stays bound and drives the adoption of an “all-NH-in” conformation, forming 6 H-bonds with the receptor.

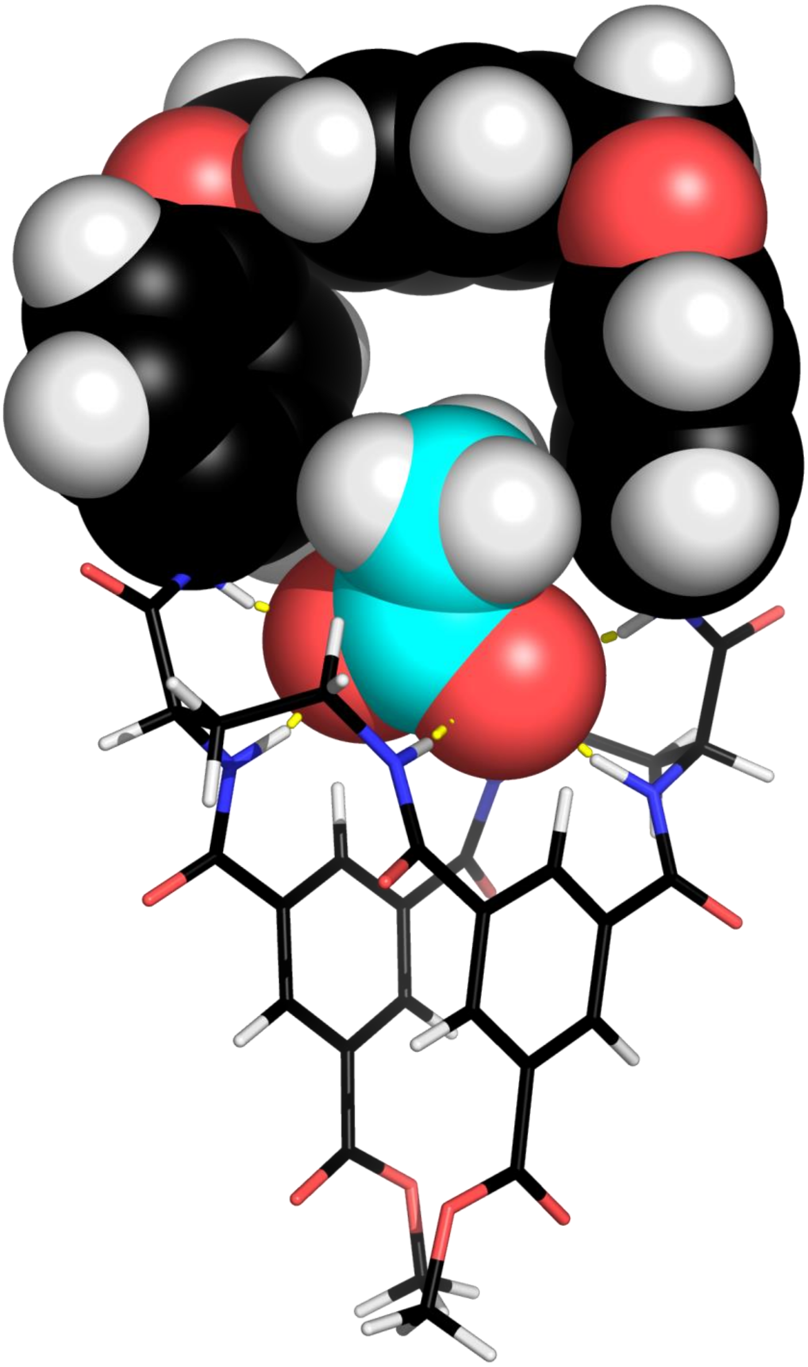

Figure S106. Lowest energy conformation from an MCMM search of the complex between receptor **7** and acetate. The acetate anion was allowed to translate and rotate while the receptor conformation was derived from Fig. S103 and kept constant.

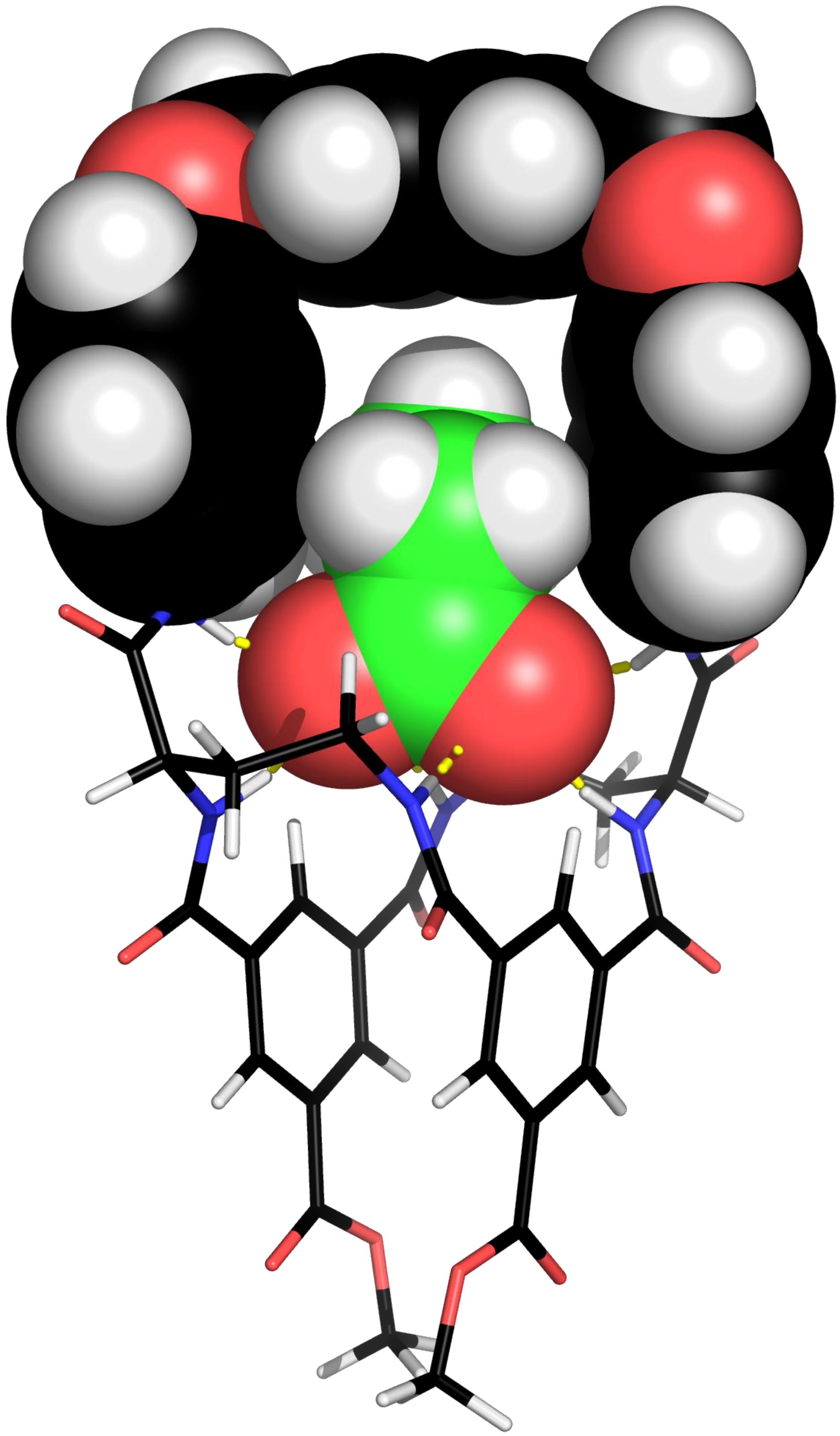

Figure S107. Energy-minimised structure of receptor **7** + propionate. This structure is one of many low energy conformations found in an MCMM search, and was chosen to illustrate the relative sizes of substrate and cavity.

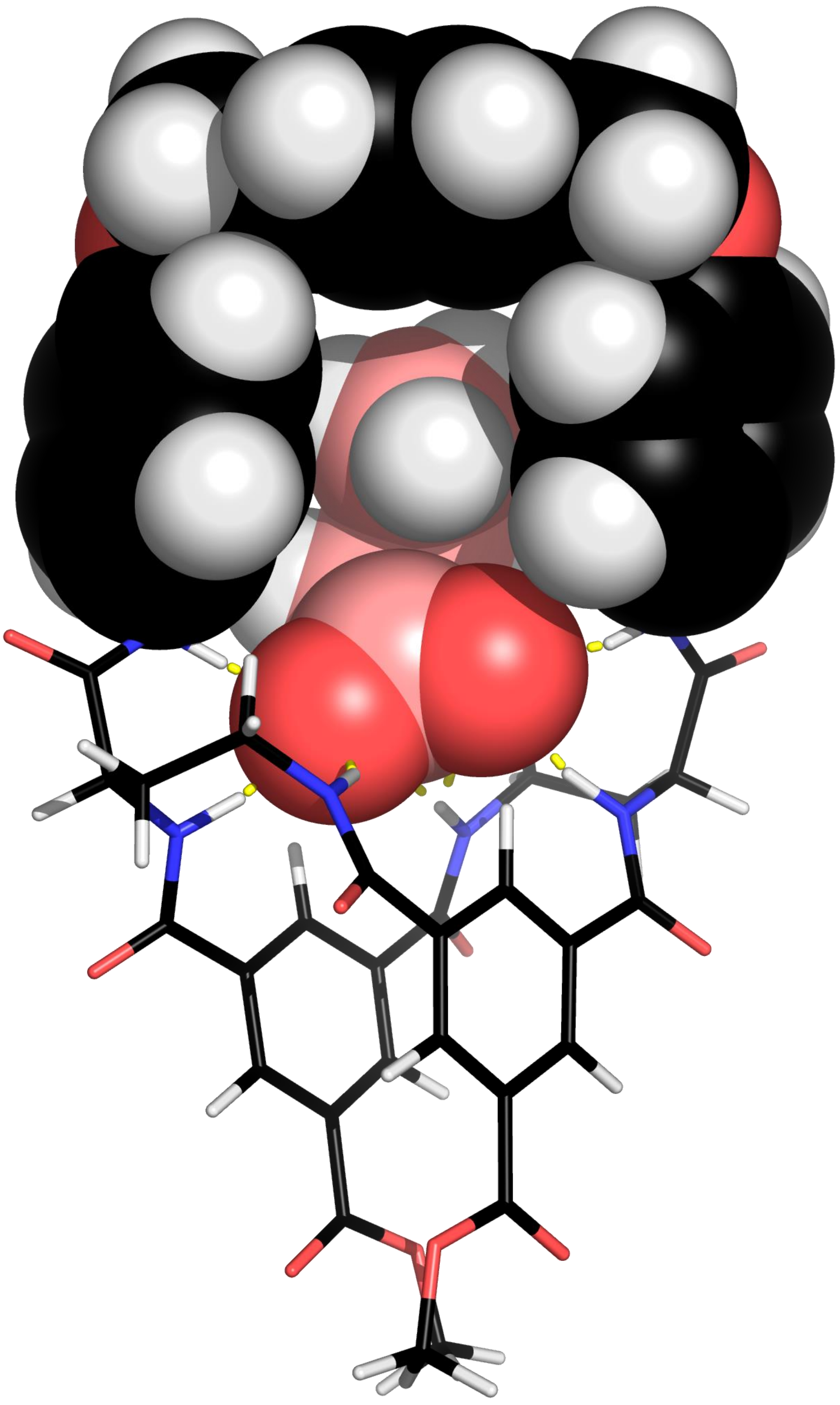

Figure S108. Energy-minimised structure of receptor **7** + butyrate. This structure is one of many low energy conformations found in an MCMM search, and was chosen to illustrate the relative sizes of substrate and cavity.

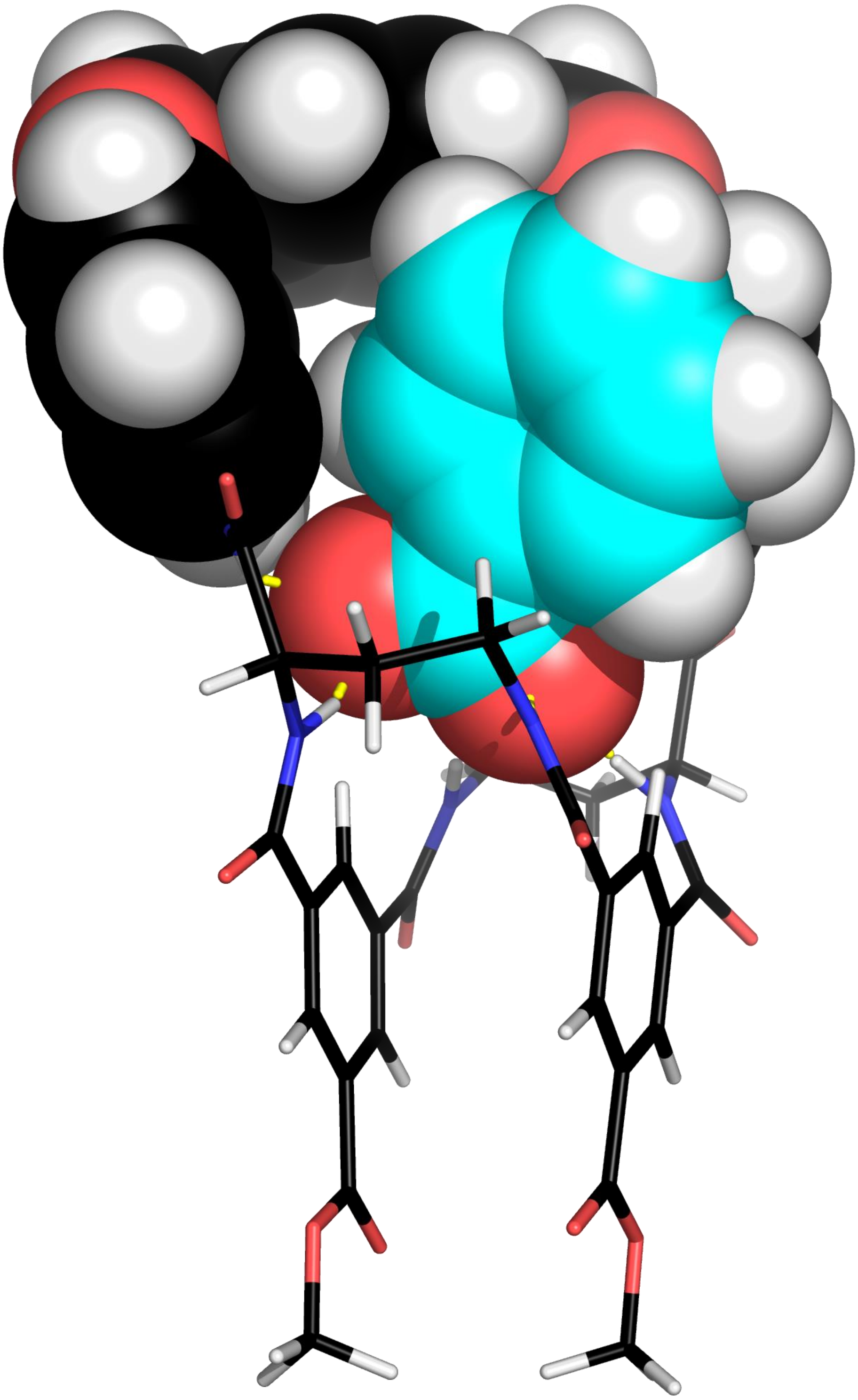

Figure S109. Energy-minimised structure of receptor **7** + benzoate. The benzoate can form 6 hydrogen bonds with the receptor but the phenyl group is too large to enter the cavity.

References

1 M. D. Weingarten, K. Sekanina and W. C. Still, Enantioselective resolving resins from a combinatorial library. Kinetic resolution of cyclic amino acid derivatives, *J. Am. Chem. Soc.*, 1998, **120**, 9112-9113.

2 A. Haider, Z. Akhter, F. Jabeen, N. K. Janjua and M. Bolte, Synthesis, structure and DNA binding studies of 1,4-bis((4-nitrophenoxy)methyl)benzene and its reduction derivative, *J. Mol. Struct.*, 2011, **994**, 242-247.

3 X. D. Ren, A. J. Flint, D. Austin and A. P. Davis, Polyanionic Receptors for Carboxylates in Water, *Angew. Chem., Int. Ed.*, 2024, e202413505.

4 A. A. J. Aquino, I. Borges, R. Nieman, A. Köhn and H. Lischka, Intermolecular interactions and charge transfer transitions in aromatic hydrocarbon–tetracyanoethylene complexes, *Phys. Chem. Chem. Phys.*, 2014, **16**, 20586-20597.

5 R. Bruckner and R. Huisgen, Diels-Alder reactions with 2,2-bis(trifluoromethyl)ethylene-1,1-dicarbonitrile as dienophile, *Tetrahedron Lett.*, 1994, **35**, 3285-3288.

6 http://supramolecular.org
